# Radical Activation of N–H and O–H Bonds at Bismuth(II)

**DOI:** 10.1021/jacs.2c05882

**Published:** 2022-09-02

**Authors:** Xiuxiu Yang, Edward J. Reijerse, Kalishankar Bhattacharyya, Markus Leutzsch, Markus Kochius, Nils Nöthling, Julia Busch, Alexander Schnegg, Alexander A. Auer, Josep Cornella

**Affiliations:** † Max-Planck-Institut für Kohlenforschung, Kaiser-Wilhelm-Platz 1, 45470 Mülheim an der Ruhr, Germany; ‡ Max-Planck-Institut für Chemische Energiekonversion, Stiftstrasse 34-36, 45470 Mülheim an der Ruhr, Germany

## Abstract

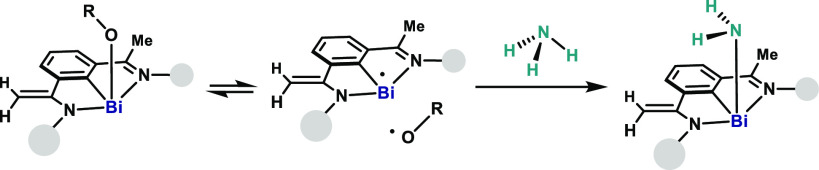

The development of unconventional strategies for the activation of ammonia (NH_3_) and water (H_2_O) is of capital importance for the advancement of sustainable chemical strategies. Herein we provide the synthesis and characterization of a radical equilibrium complex based on bismuth featuring an extremely weak Bi–O bond, which permits the in situ generation of reactive Bi(II) species. The ensuing organobismuth(II) engages with various amines and alcohols and exerts an unprecedented effect onto the X–H bond, leading to low BDFE_X–H_. As a result, radical activation of various N–H and O–H bonds—including ammonia and water—occurs in seconds at room temperature, delivering well-defined Bi(III)-amido and -alkoxy complexes. Moreover, we demonstrate that the resulting Bi(III)–N complexes engage in a unique reactivity pattern with the triad of H^+^, H^–^, and H^•^ sources, thus providing alternative pathways for main group chemistry.

## Introduction

**Figure 1:**
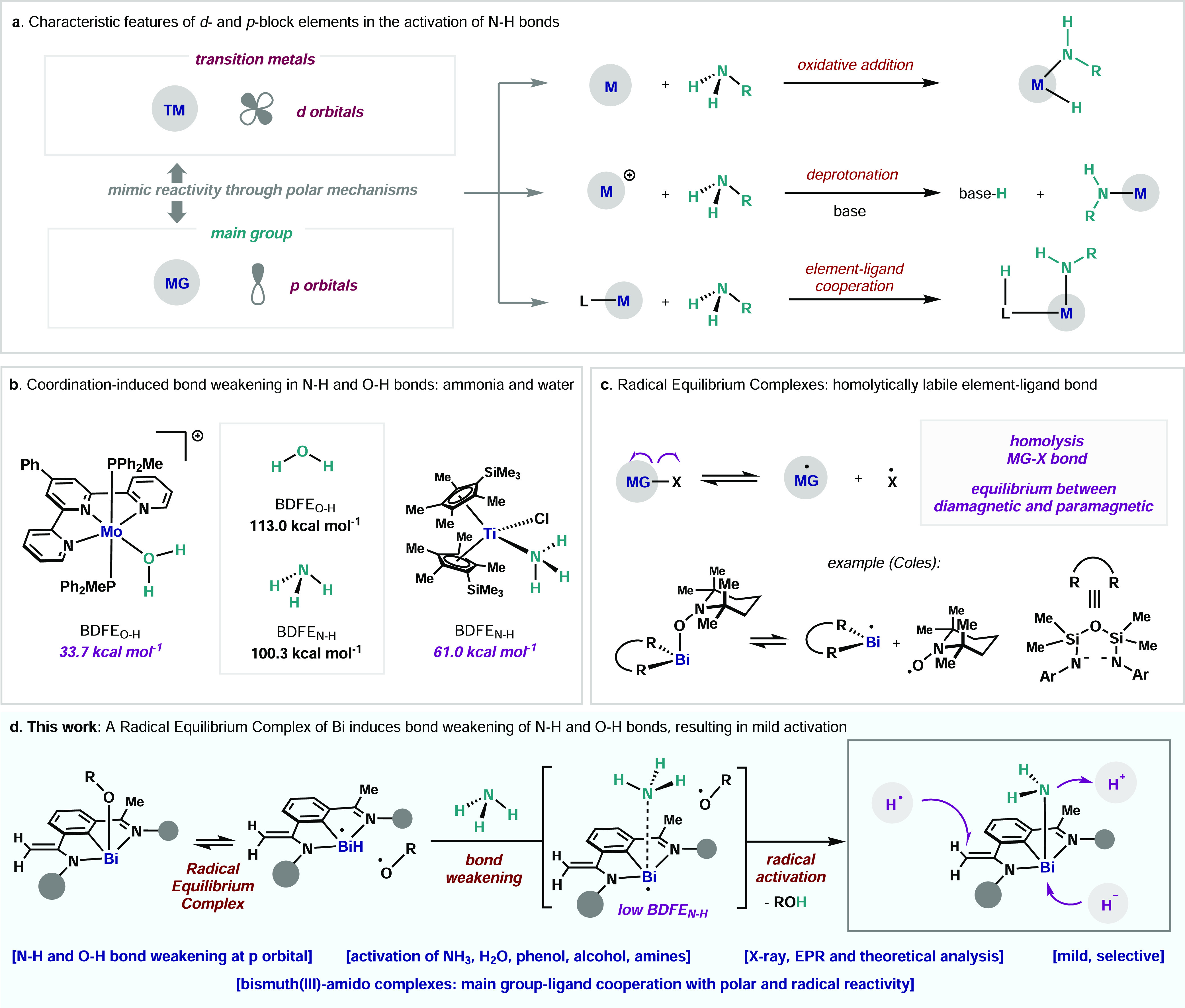
Overview of N–H and O–H activation modes. (a) State-of-the-art modes for X–H activation by transition metals and main group elements; example of amines. TM, transition metal; MG, main group element. (b) Bond weakening of N–H and O–H by coordinating to transition metal complexes. (c) Top: reversible homolysis of MG–X single bond in radical equilibrium complex (REC). Bottom: an example of a Bi REC (right).^20^ (d) This work: activation of N–H and O–H by a Bi–O REC complex, and reactivity of the new Bi–amido compounds. TM: transition metal; MG: main group element.

Compounds bearing N–H and O–H functionalities are prevalent motifs in both the natural and the synthetic world. Among them, ammonia (NH_3_) and water (H_2_O) occupy the most prominent positions; indeed, they have been identified as energy units or economic building blocks en route to high-value compounds.^1,2^ However, chemical manipulation of N–H and O–H bonds is nontrivial, as a result of the high bond dissociation free energy (BDFE), e.g., BDFE_O–H_ in H_2_O = 113.0 kcal·mol^–1^; BDFE_N–H_ in NH_3_ = 100.3 kcal·mol^–1^.^3^ Indeed, the majority of the approaches toward X–H cleavage focus on polar pathways; for example, both *d*- and *p*-block elements undergo oxidative addition^4^ or deprotonation through metal–ligand cooperation^4,5^ using the two electrons of the respective *d*- and *p*-orbitals (Figure 1a). More recently, the activation of the X–H bonds through radical pathways has become feasible, albeit comparatively fewer examples are known. Although *s*-block elements can activate X–H bond through single-electron transfer (SET),^6^ milder strategies capitalizing on the concept of coordination-induced bond weakening have recently arisen (Figure 1b).^7^ Examples of this reactivity are found in biology,^8^ catalysis,^9^ coordination chemistry,^7a^ or ammonia synthesis.^10^ Yet, such reactivity is largely dominated by transition metals, and examples dealing with main group elements remain rare.^11,12^ For example, a (corrolato)germanium-TEMPO complex (group 14) has been reported to activate N–H and O–H bonds under visible light.^11,13^ Without irradiation, low yields were obtained at higher temperatures and extended reaction times. In group 13, boron-containing compounds have been shown to lower the BDFE of E–H, including H_2_O and NH_3_.^11b−11e^ Despite these examples, complexes based on group 15 elements that enable selective, fast, and mild radical activation of O–H and N–H bonds through coordination-induced bond weakening properties are rare.

As the heaviest stable element,^14^ the electronic structure of bismuth (Bi) is strongly influenced by relativistic effects, thus decreasing the energies of its 6*s* and 6*p* orbitals. Consequences of these unique electronic features are the well-known inert-pair effect^15^ or strong Lewis acidity.^16^ In certain cases, homolysis of LBi–X bonds becomes feasible due to the preferential stability of the LBi radical over the ionized heterolysis product LBi^+^.^17^ In principle, if the LBi· and X· generated from homolysis are stable, it is possible that this complex exists in both diamagnetic and paramagnetic form: a radical equilibrium complex (REC) (Figure 1c).^18^ Examples of Bi REC are scarce, and the few reported homolysis cases of the Bi–X bond are mainly irreversible, due to the high reactivity of the ensuing Bi radical species.^19^ Fundamental studies on a Bi–Mo catalyst for the SOHIO process conducted by Hanna suggested that Bi(II) intermediates—formed after thermal homolysis of the Bi(III) bearing bulky phenolates—could be responsible for the formation of allyl radicals from propene.^19c^ Similar reactivity with bulky phenolate anions was later observed by Evans in a unique C–H bismuthylation of phenols.^19a^ In 2018, Coles demonstrated that the Bi(III)–O bond in a R_2_Bi–OTEMP compound is in equilibrium with the corresponding R_2_Bi(II) and TEMPO·.^20^ Collectively, these precedents pointed to a facile thermal Bi–O bond scission of bulky oxy-type anions that can stabilize O centered radicals.^19d,19g^ Yet, the origin and factors that influence this process still remain unclear, and investigations on such unusual chemical properties would be desirable. Herein we report on the synthesis, reactivity, and structural characterization of a Bi REC, whose Bi–O homolyzes reversibly at room temperature without the need of irradiation (Figure 1d). We demonstrate that such a complex permits fast and mild activation of ammonia and water—among other alcohols and amines—resulting in well-defined Bi(III) amido and alkoxy compounds. We suggest that upon coordination to Bi(II), amines and alcohols undergo X–H bond weakening, thus permitting their facile radical activations. In addition, we propose that the novel pincer-based Bi(III)–NR_2_ compounds show reactivity with a triad of H^+^, H^–^, and H^•^ sources.

## Results and Discussion

**Figure 2:**
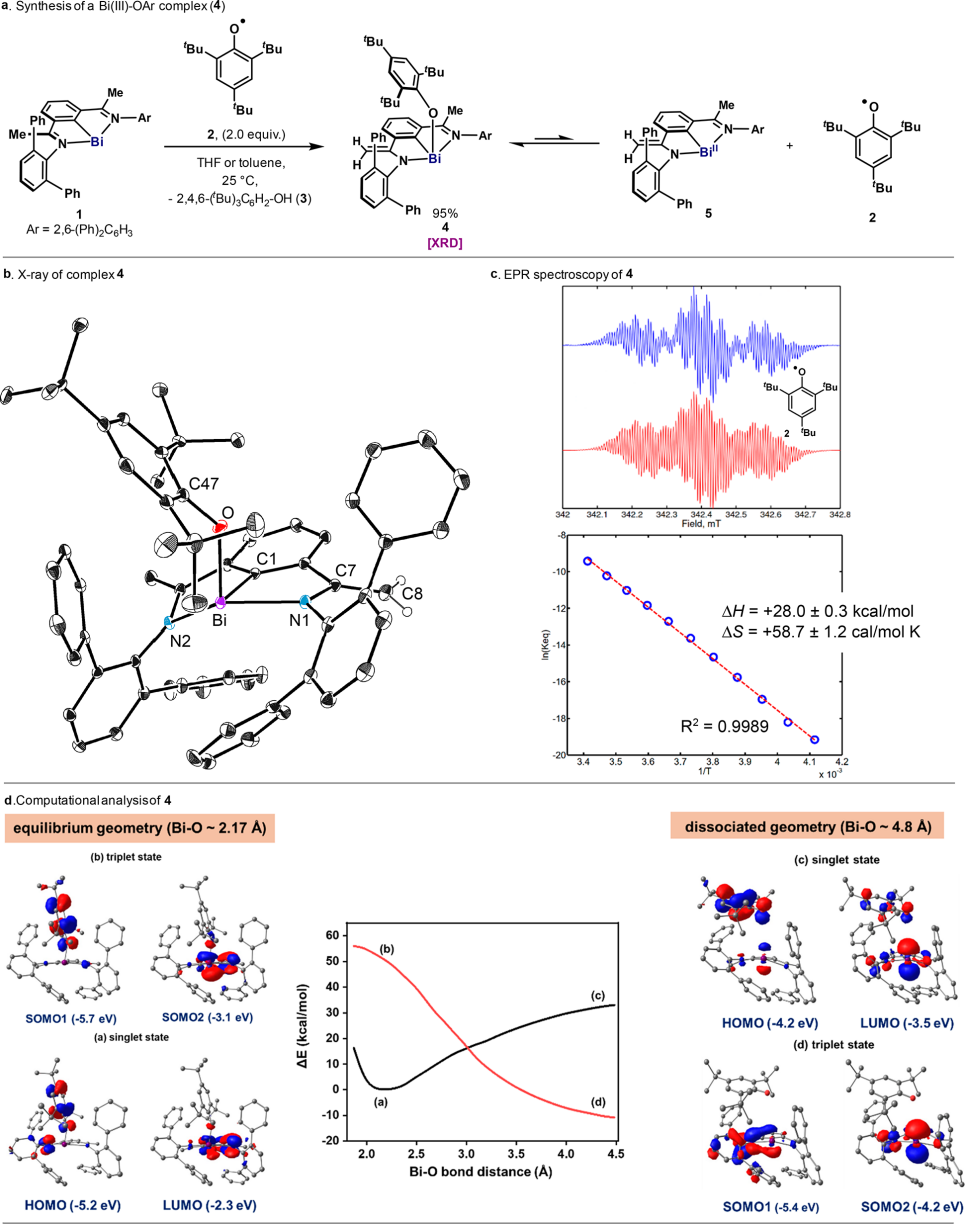
Synthesis and characterization of a bismuth radical equilibrium complex. (a) Synthesis of complex **4**. (b) Solid-state structure of **4**, illustrated using 30% probability ellipsoids except hydrogen atoms. Solvents, hydrogen atoms, and disordered parts have been omitted for clarity, except those on C8. (c) Top: (blue line) EPR spectrum of complex **4** (after dissociation) at 25 °C, showing the presence of **2**; (red line) spectral simulation of **2**. Parameters: *g* = 2.00854, 2×^1^H-A_iso_ = 4.76 MHz, 9×^1^H-A_iso_ = 1.04 MHz, 18×^1^H-A_iso_ = 0.2 MHz. Bottom: van’t Hoff plot of **4** in PhMe between −30 and 20 °C. (d) Computational analysis of the Bi–O bond cleavage: potential energy profiles of the Bi–O bond dissociation of **4** at (ZORA) PBE0-D3/Def2-TZVP (SMD:Toluene) level of theory. Black and red color denote singlet (heterolytic bond cleavage) and triplet (homolytic bond cleavage) potential energy surface, respectively. Frontier molecular orbitals both in singlet (a,c) and triplet states (b,d) are plotted at equilibrium (left panel) and dissociated (right panel) geometries.

Reaction of *N*,*C*,*N* organobismuth(I) **1** with 2.0 equiv of alkoxide radical **2** in THF led to the isolation of **4** in 95% yield as an orange solid, with concomitant formation of **3** (Figure 2a). Single crystal XRD reveals **4** as a monomeric structure and a 4-fold coordinated Bi center (Figure 2b, and SI). The bond distances of C7–C8 (1.355(6) Å) and C7–N1 (1.379(5) Å) clearly indicate a C=C double bond and a C–N single bond, respectively. The angles between Bi and the distinct three anionic ligands (C1, N1, O) vary from 75.48(13)° to 95.07(12)°, with a sum of angles up to 256.2°, pointing to a major contribution of the 6*p* Bi orbitals in the Bi–X bonds (X = C1, N1, O).^21^ Importantly, the Bi–O distance (2.178(3) Å) and the angle of C47–O–Bi (136.2(3)°) are larger than the closely related BiCl(O-2,4,6-^*t*^Bu_3_C_6_H_2_)_2_ (Bi–O: 2.091(3) and 2.094(3) Å; C–Bi–O: 123.8(2) and 118.0(3)°).^22^ This implies a poor overlap between the lone pair in the *sp*^2^ hybridized orbital of the O atom and the diffuse *p* orbital of the Bi center, indicating a weak Bi–O bond. EPR analysis of **4** at room temperature resulted in the clear detection of the signal for the known radical **2** (Figure 2c, top),^23^ which could be characterized with high resolution. Due to the relatively high temperature (>100 K) for the Bi–O bond homolytic cleavage, only **2** was detected, as the EPR signal for Bi(II) (**5**) is assumed to be too broad to be detectable, because of a fast relaxation caused by large spin–orbit coupling.^19f^ When **4** (13.08 mM) was subjected to successive cycles of temperature changes within the range of 243–293 K, the concentration of **2** remained constant at a given temperature, supporting the reversibility of the homolytic cleavage (see SI). The thermodynamic parameters of the equilibrium in PhMe (Δ*H* = +28.0 ± 0.3 kcal·mol^–1^ and Δ*S* = +58.7 ± 1.2 cal·mol^–1^·K^–1^) are consistent with a dissociative mechanism (Figure 2d, bottom).^19f^ Importantly, the large contribution of the entropy compensates for the unfavorable enthalpy and results in Δ*G* = +10.5 ± 0.67 kcal·mol^–1^ at 298 K between the diamagnetic and the paramagnetic species. Computed singlet and triplet bond dissociation potential energy profiles of **4** at the PBE0/Def2-TZVP (ZORA)^24^ level of theory are shown in Figure 2d. Upon elongation of the Bi–O bond, the triplet state crosses the singlet state at around ∼3.1 Å, indicating that splitting into two radical species is energetically favorable by 37.2 kcal·mol^–1^. Spin density analysis indicates a considerable spin polarization on the Bi center when the Bi–O bond is elongated to 2.3 Å (see SI). Orbital analysis of the singlet state for **4** shows that the HOMO is predominantly located on the alkoxide ligand and the LUMO on the *N*,*C*,*N* ligand and neighboring Bi. The Bi–O cleavage is essentially completed at ca. 4.5 Å. Importantly, values of Δ*H* = +25.0 kcal·mol^–1^ and Δ*S* = +62.1 cal·mol^–1^·K^–1^ for the Bi–O scission are in good agreement with the experimental thermodynamic data obtained by EPR. The considerable entropic contribution is attributed to high translational and rotational entropy components resulting in a rather small computed Δ*G* = +6.5 kcal·mol^–1^ (Δ*G*_exp_ = +10.5 ± 0.67 kcal·mol^–1^). In comparison, the heterolytic bond cleavage of the Bi–O bond, is highly endergonic with Δ*G* = +43.7 kcal·mol^–1^, supporting the energetic preference for the formation of radical **2** and **5**. It is important to highlight that the weak Bi–O bond is a consequence of the relativistic effect of Bi, which combined with the stability of the Bi(II) by the pincer framework, the stability of radical **2**, and the large entropic gain, results in a mild reversible homolytic cleavage.

**Figure 3:**
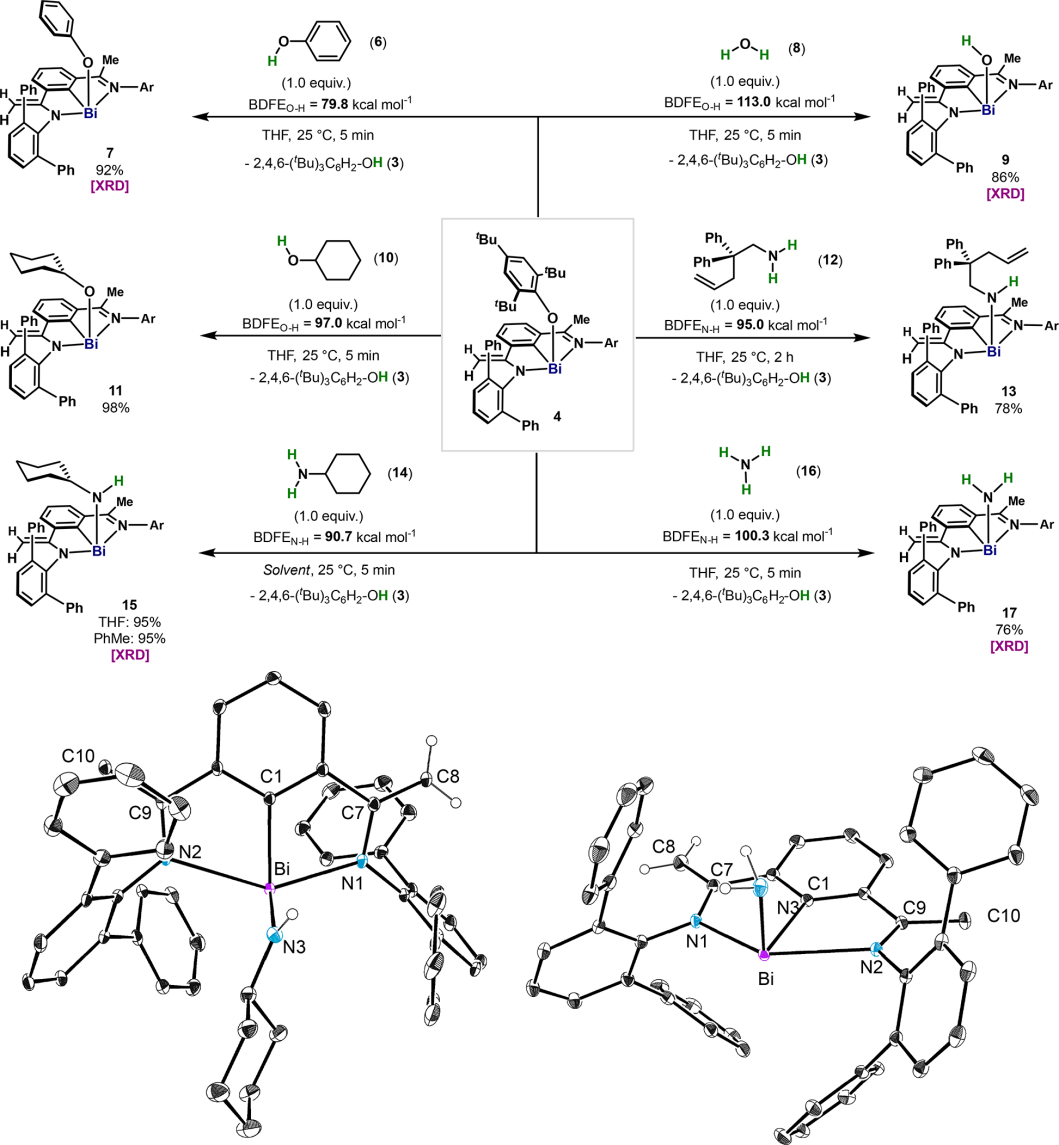
Activation of O–H and N–H bonds: synthesis of **7**, **9**, **11**, **13**, **15**, **17** (top), and solid-state structure of **15** (bottom, left) and **17** (bottom, right), illustrated using 30% probability ellipsoids except hydrogen atoms. Solvents, hydrogen atoms, except those on C8 and N3 in **15** and **17**, and disordered parts have been omitted for clarity. All yields are of isolated pure material.

The BDFE of X–H on a ligand is influenced by the oxidation potential at the metal center and the p*K*_a_ value of X–H.^3^ Therefore, putative coordination to Bi(II) would increase the population of the antibonding orbitals, making LBi(II)–X–H a strong reductant.^13^ Hence, the Bi(II)/Bi(III) redox couple presents itself as a good candidate for coordination-induced bond weakening. When **4** was mixed with 1.0 equiv of phenol (**6**, BDFE_O–H_ = 79.8 kcal·mol^–1^),^3^**7** was formed quickly and obtained in a 92% isolated yield (Figure 3). Interestingly, the reaction with 1.0 equiv of H_2_O (BDFE_O–H_ = 113.0 kcal·mol^–1^) led to rapid conversion to the corresponding hydroxy bismuth **9** (86%), which has recently been characterized in the context of N_2_O activation.^25^ Cyclohexanol afforded the corresponding bismuth alkoxide **11** in 98% yield. Similarly, when primary α-monoalkyl (**12**, BDFE_N–H_ = 95.0 kcal·mol^–1^) and α-dialkyl amines (**14**, BDFE_N–H_ = 90.7 kcal·mol^–1^) were mixed with **4**, the corresponding bismuth amides were obtained in 78% (**13**) and 95% (**15**) yields, respectively. Similar yields were observed in apolar nonprotic solvents, as shown for the 95% yield of **15** in PhMe. Finally, when **4** was mixed with 1.0 equiv of dry ammonia, **17** was isolated in 76% yield. It is important to mention that Bi(III) complexes bound to a free NH_2_ group are rare,^26^ and therefore, **17** represents a unique example of such a pnictogen–pnictogen bond. In all cases, solid-state structures reveal the bismuth center to be 4-fold coordinated, and residing in a distorted plane formed by the imine, amido and phenyl ring (see SI). The −OH, −OPh, −NHCy, and −NH_2_ groups in **7**, **9**, **15**, and **17** are perpendicular to this plane, and they localize on either side. The bond distances of C7–C8 and C7–N1 clearly indicate that the C=C double bonds and C–N single bonds are preserved. It is important to mention that no EPR signal was detected from **7**, **9**, **11**, **13**, **15**, and **17** in toluene at various temperatures. Moreover, the reaction of **7** with CyOH (**10**) produced <5% of **11**, thus highlighting the unique reactivity of **4**.

**Scheme 1:**
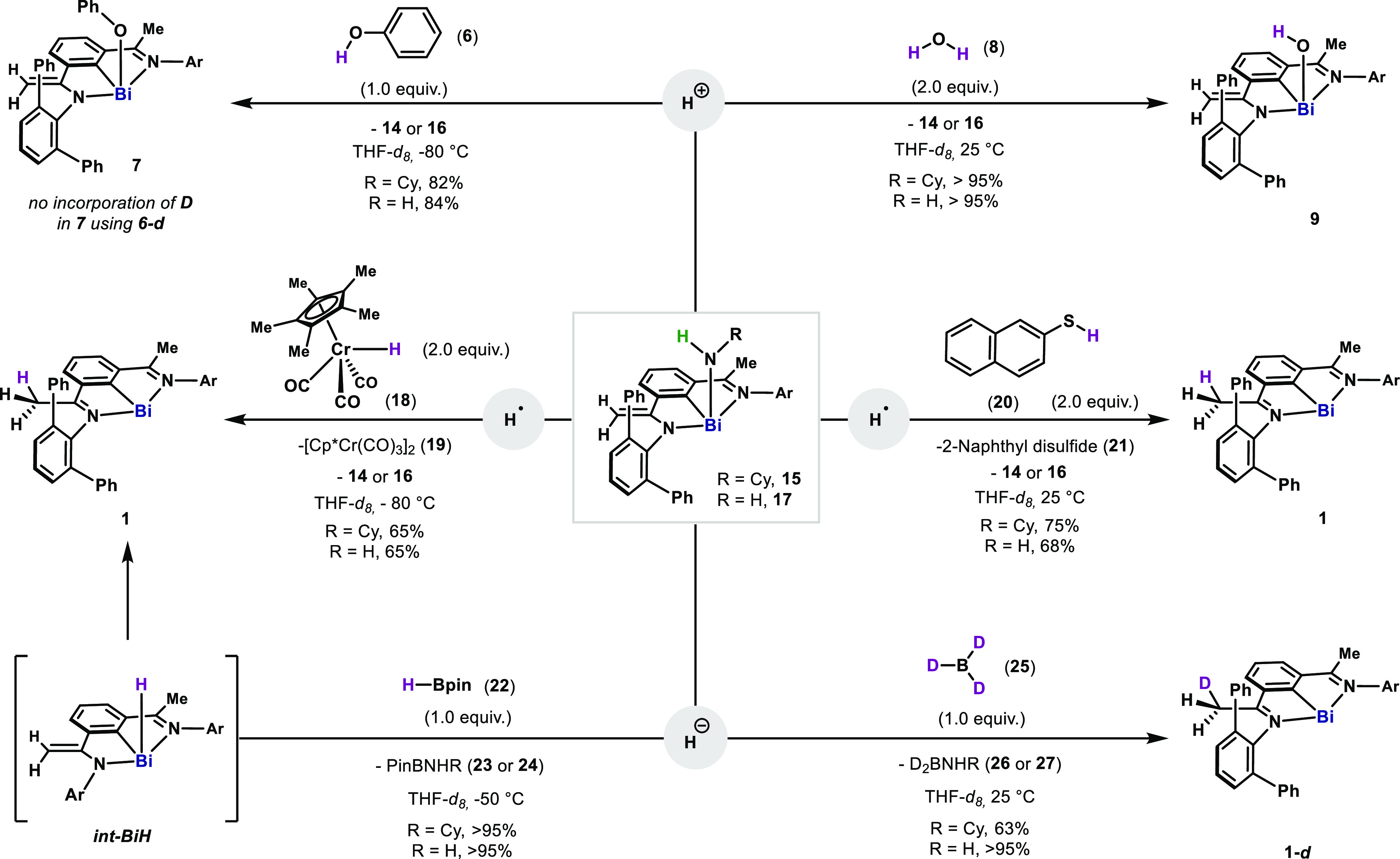
Reactivity of Bismuth(III) Amido Complexes **15** and **17** with H^+^, H^–^, and H^•^ Sources Ar = 2,6-diphenylphenyl.

Generally, main group elements beyond group 14 have a reduced tendency to form stable complexes with NH_3_,^5b^ and therefore represent excellent candidates for NH_3_ activation and direct conversion to added-value chemicals beyond the stable MG–NH_2_ compounds.^27^ In order to explore their reactivity, **15** and **17** were mixed with various X–H sources (Scheme 1). Initially, the basicity of the Bi–NH_2_ and Bi–NHCy bond was confirmed by the immediate reaction at −80 °C with **6**, leading to **7** and **14**/**16**. Such basicity is also demonstrated by the reaction with H_2_O, leading quantitatively to **9**. When **6** was replaced by **6-***d*, no deuteration of the ligand was observed, pointing to reactivity occurring solely at the Bi center. Additionally, when **15** and **17** were mixed with a chromium hydride (**18**) with aweak Cr–H bond (BDFE_Cr–H_ = 53.0 kcal·mol^–1^),^28^ reduction to **1** rapidly occurred at −80 °C, with no intermediates detected. Concomitantly, Cr–Cr dimer **19** and **14**/**16** resulted, which point to a radical reaction of **15** and **17** with a weak H^•^ source.^19d,19e^**1** was also produced selectively when **15** and **17** were mixed with 2 equiv of 2-naphthalenethiol (**20**) (BDFE_S–H_ = 75.9 kcal·mol^–1^, see SI), which formed 2-naphthyl disulfide (**21**) and **14**/**16**.^29^ Interestingly, when **15** and **17** were mixed with HBpin, an alternative hydride source with a much larger BDFE_B–H_ = 108.6 kcal·mol^–1^,^30^ reduction to **1** occurred with the formation of **23**/**24**. In this case, a Bi(III)–H (*int-BiH*) could be detected at −50 °C, featuring the characteristic signal in the ^1^H NMR at +26.0 ppm.^31^*Int-BiH* slowly converted into **1** at −50 °C, with the migration of the H atom to the methylene backbone. Incorporation of one deuterium in the methyl groups on the backbone using DBpin further confirmed such migration (see SI). Moreover, reduction of **15** and **17** to **1-***d* could also be accomplished using BD_3_. Collectively, these bismuth-amido complexes feature chemically noninnocent reactivity (element–ligand cooperation),^32^ as well as reactivity toward radical species.

**Figure 4:**
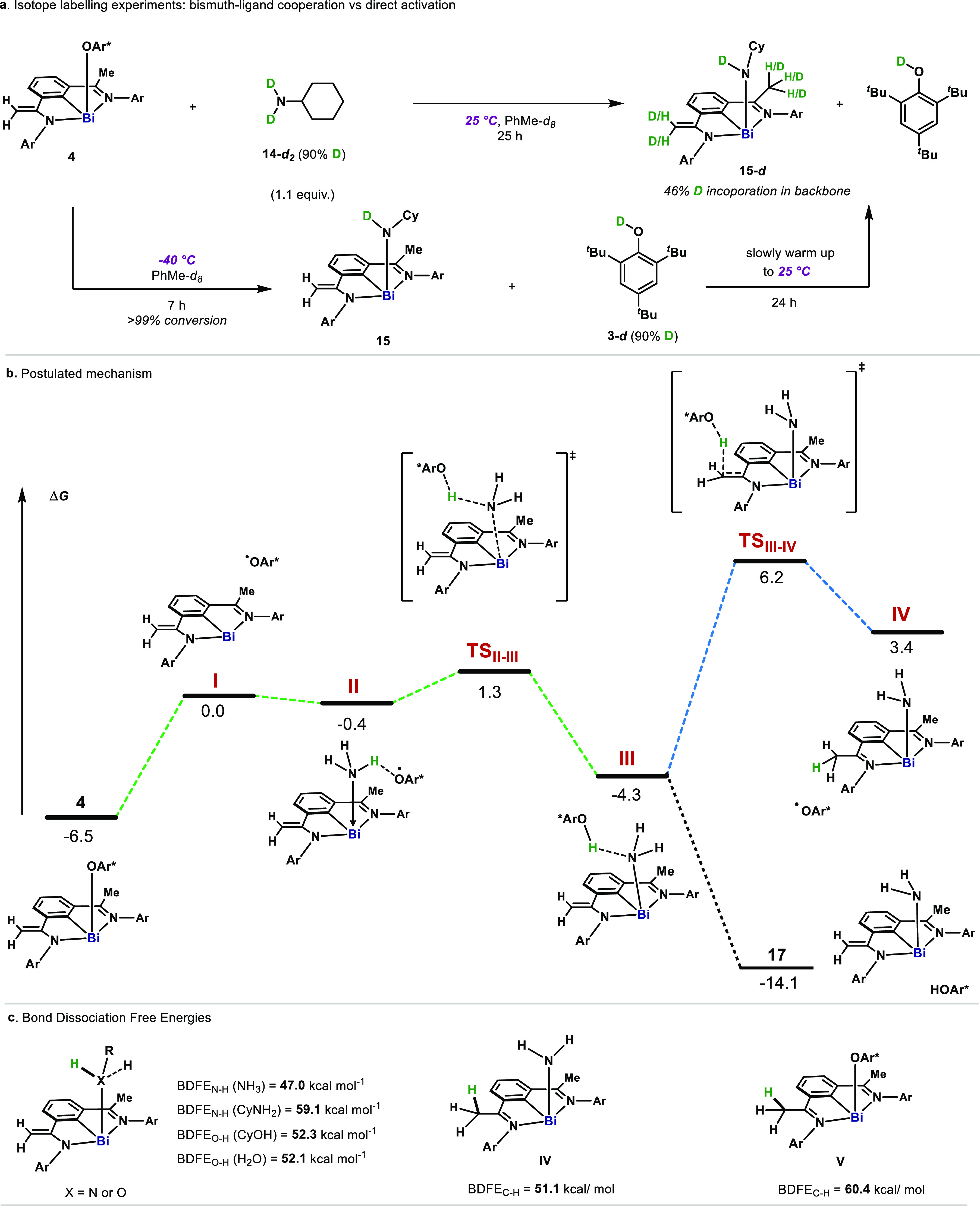
Mechanistic investigations. (a) Deuterium labeling experiments at various temperatures. (b) Computational analysis of the mechanism of the radical activation of N–H bond in ammonia. Computed free energy (Δ*G*, in kcal·mol^–1^) profile for the N–H bond cleavage of NH_3_ by 5/OAr pair. Relative free energies (in kcal·mol^–1^) are computed based on (ZORA) PBE0-D3/Def2-TZVP (SMD:Toluene) single point energies, and gas-phase free energies corrections at 298.15 K obtained at the (ZORA) BP86-D3/Def2-TZVP level of theory. (c) Calculated BDFE of N–H and O–H bonds after coordination with Bi(II). OAr* = 2,4,6-(^*t*^Bu)_3_C_6_H_2_O.

As shown in Scheme 1, element–ligand cooperation through the methylene unit is feasible during radical and hydride processes involving H. To evaluate whether similar reactivity is involved in the activation of N–H bonds, we carried out the activation of deuterated cyclohexylamine (**14-***d*_2_, 90% D) using **4**. As shown in Figure 4a, both methyl and methylene moieties resulted in an enrichment of deuterium (46% D) after 25 h at 25 °C. However, when the reaction was carried out at −40 °C and monitored by NMR, no obvious incorporation of D in the backbone was detected after complete conversion to **15**. Only upon warming the reaction mixture to 25 °C, a clear exchange of H for D in the CH_3_ and CH_2_ could be detected. These experiments confirm the following: (1) the absence of H/D exchange on the ligand by **14-***d*_2_ during X–H activation; and (2) that ligand noninnocent reactivity with **3-***d* is triggered at higher temperatures from amido complex **15**. Figure 4b contains the computed free energy profile for the N–H bond activation step (green-dotted line). Based on the combined experimental evidence and the computational analysis, it is proposed that upon reversible homolysis of the Bi–O bond in **4**, NH_3_ coordinates to the Bi(II) radical through the semioccupied 6*p*_*z*_ orbital to generate **II**. HAT from **II** to OAr radical (**2**) proceeds with a very low energy barrier (**TS**^**II–III**^, Δ*G* = +1.3 kcal·mol^–1^), resulting in **III** (Δ*G* = −4.3 kcal·mol^–1^). The low energy barrier associated with **TS**^**II–III**^ is the result of the remarkably low BDFE_N–H_ = 47.0 kcal·mol^–1^ calculated for the N–H bond once coordinated to the Bi(II) center. Such a coordination-induced bond weakening effect of the Bi(II) was also observed for H_2_O, CyNH_2_, and CyOH, with BDFE_X–H_ = 52.1, 59.1, and 52.3 kcal·mol^–1^, respectively (Figure 4c, left). Without hydrogen bonding with HOAr* (**3**), **17** is significantly lower in energy (−14.1 kcal mol^–1^), permitting its isolation. The hydrogen exchange observed experimentally at the vinylic C–H bonds after the N–H activation was also computationally evaluated (Figure 4b, blue-dotted line). The computed barrier for the radical hydrogen exchange between **III** and **IV** raises to Δ*G*^‡^ = +10.5 kcal·mol^–1^, due to the energetic mismatch between **IV** (BDFE_C–H_ = +51.1 kcal·mol^–1^) and **2** (BDFE_O–H_ = +76.8 kcal·mol^–1^)^3^ (Figure 4c, middle). Upon single electron transfer (SET) between **1** and **2**, the Me C–H bond in the backbone in **V** also undergoes bond-weakening (Figure 4c, right, BDFE_C–H_ = +60.4 kcal·mol^–1^),^33^ resulting in feasible H-abstraction by **2** en route to starting complex **4** (Figure 2a). The small energy difference between **4** and **III** indicates that NH_3_ activation might be reversible,^5b^ which was confirmed by the incorporation of deuterium in the CH_2_ groups of **4** in the presence of ND_3_ (see SI). Finally, alternative pathways such as direct HAT from **2** to NH_3_ without the involvement of Bi, or reaction between **4** and NH_3_ through heterolytic bond cleavage, were discarded due to high energy transition states obtained in the free energy profile (>40 kcal·mol^–1^, see SI).

## Conclusions

In this article, we disclose the design, synthesis, and reactivity of a bismuth REC (**4**), featuring a weak Bi–O bond. The facile homolysis at room temperature leads to a highly reactive Bi(II) species (**5**) with unusual chemical properties. Under mild conditions, compound **4** is able to perform a rapid and selective activation of amines and alcohols—including ammonia and water—resulting in exclusive alkoxy- and amido-bismuth(III) complexes. A combined experimental and computational analysis of the system suggests that upon homolysis, coordination of the lone pair in X–H to **5** occurs, resulting in a dramatic reduction of the BDFE_X–H_, which enables its cleavage by the phenoxy radical. Reactivity studies of the novel Bi(III)–NHR resulted in engagement with the triad of proton, hydride, or radical hydrogen sources, a rather unique feature for main group elements. Although Bi(III)–NHR have shown reactivity involving the ligand framework, deuteration experiments and kinetic analysis indicate that no element–ligand cooperation occurs during the activation, in agreement with the mechanistic hypothesis from a computational analysis. Properties such as coordination-induced bond weakening at bismuth combined with the rich reactivity pattern offered by the Bi(III)-amido complexes (at metal and ligand) provide a platform for further exploration in the area of bismuth radical catalysis.

## Supporting Information Available

Experimental procedures and analytical data (^1^H, ^13^C, ^15^N NMR, HRMS, and crystallographic data) for all new compounds (PDF)
