# Supplementary material for: Radical Activation of N–H and O–H Bonds at Bismuth(II)
Source: J Am Chem Soc. 2022 Sep 2;144(36):16535–44. doi: 10.1021/jacs.2c05882 (PMC9479083; doi:10.1021/jacs.2c05882)
Supplement: Supplementary file 1 — ja2c05882_si_001.pdf [file ja2c05882_si_001.pdf]

Radical Activation of N–H and O–H bonds at Bismuth(II)

Xiuxiu Yang[1], Edward J. Reijerse‡[2], Kalishankar Bhattacharyya‡[1], Markus Leutzsch[1], Markus Kochius[1], Nils Nöthling[1], Julia Busch[1], Alexander Schnegg[2], Alexander A. Auer[1], Josep Cornella[1]

[1]Max-Planck-Institut für Kohlenforschung, Kaiser-Wilhelm-Platz 1, 45470, Mülheim an der Ruhr, Germany.

[2]Max Planck Institute for Chemical Energy Conversion, Stiftstrasse 34-36, D-45470, Mülheim an der Ruhr, Germany.

cornella@kofo.mpg.de

Contents

1 General methods 4

2 Proposed mechanisms for the generation of 4 5

3 Preparation of bismuth complexes 6

3.1 Preparation of **4** 6

3.2 Preparation of **7** 8

3.3 Preparation of **9** 10

3.4 Preparation of **11** 12

3.5 Preparation of **13** 14

3.6 Preparation of **15** 16

3.7 Preparation of **17** 19

4 Reactivity of 15 and 17 towards H^+, H·, and H^- 23

4.1 The reaction of **15** and **17** with PhOH 23

4.2 The reaction of **15** and **17** with H_2O 23

4.3 The reaction of **15** and **17** with HBpin 24

4.4 The reaction of **15** and **17** with BD_3 27

4.5 The reaction of **15** and **17** with $Cp^*CrH(CO)_3$ 28

4.6 The reaction of **15** and **17** with 2-naphthalenethiol 28

5 Isotope labeling experiments 30

5.1 The reaction of **4** with ND_3 in C_6D_6 30

5.2 The reaction of **4** with ND_3 in C_6H_6 32

5.3 The reaction of **4** with ND_3 (< 1 eq.) in C_6H_6 33

5.4 The kinetic reaction of **4** with $CyND_2$ at -40 °C 34

6 EPR measurements 37

7 Computational Setup 43

7.1 Input files for DFT calculations 43

7.2 Bi-O bond dissociation free energy of complex **4** 44

7.3 Bi-O bond dissociation free energy of complex **7** and **17** 46

7.4 Spin density plot 47

7.5 Bond dissociation free energies (BDFE) values 48

7.6 Computational studies of N-H bond activation 49
7.6.1 Results of the mechanistic pathway for H/D exchange: coordination followed by direct hydrogen transfer to OAr radical pathway 50
7.6.2 Mechanism of ammonia coordination and metal-pincer ligand cooperation pathway 51
7.6.3 Mechanism of ammonia coordination and heterolytic cleavage of N-H bond pathway 52
7.6.4 Mechanism of N-H bond cleavage by complex **4** pathway 53
8 Crystallographic data 54
8.1 Single crystal structure analysis of **4** 55
8.2 Single crystal structure analysis of **7** 69
8.3 Single crystal structure analysis of **15** 80
8.4 Single crystal structure analysis of **17** 92
9 NMR spectra of bismuth compounds 105
10 Coordinates from Geometry Optimizations 135
11 References 187

1 General methods

All experiments were performed under argon atmosphere using standard Schlenk and glovebox techniques. THF, *n*-pentane, benzene, THF-d_8, C_6D_6 were degassed and distilled from sodium/benzophenone and stored with 4Å molecular sieves under argon. Toluene-d_8 was degassed and distilled from sodium and stored with 4Å molecular sieves under argon. H_2O was degassed before use. PhOH was sublimed under vacuum and stored under argon. CyOH was distilled from Na, and stored with 4Å molecular sieves under argon. 2,2-diphenyl-4-penten-1-amine was stored with 4Å molecular sieves under argon. $CyNH_2$ was dried with $LiAlH_4$, distilled, and stored with 4 Å molecular sieves under argon. NH_3 was generated in-situ in a two-chamber system [1]. **1** [2], 2,4,6-tri-*tert*-butylphenoxyl radical [3], O-d-2,4,6-tri-*tert*-butylphenol [4], PhOD [5], $CyND_2$ [6], and Cp*CrH(CO)$_3$ [7] were prepared by published methods.

NMR spectra were recorded on a Bruker Avance IIIHD nanobay 300 MHz, or an AvanceNeo 600 MHz NMR spectrometer with respect to ^{1}H. Low temperature NMR spectra were recorded on a Bruker 500 MHz NMR spectrometer with respect to ^{1}H. The spectra were measured at 298K unless noted otherwise. The AvanceNeo 600 MHz NMR spectrometer was equipped with a BBO cryoprobe. Chemical shifts are reported in ppm relative to TMS and residual chemical shifts of the solvent as the secondary standard (for ^{1}H and ^{13}C). Chemical shifts assignments were established using high resolution 2D NMR spectra (^{1}H, ^{13}C-*edited*-HSQC, ^{1}H, ^{13}C-HMBC, ^{1}H-^{1}H-COSY, ^{1}H-^{1}H-EASY-ROESY). ^{15}N chemical shifts were extracted from cross-peaks observed in ^{1}H-^{15}N HMBC experiments. ^{15}N chemical shifts are reported relative to $MeNO_2$ ($\delta(^{15}N) = 0$ ppm) and were referenced with their Ξ -value = 10.136 767% relative to the ^{1}H NMR spectrum using the *xiref* command in Bruker Topspin 4.0.6 [8]. Coupling constants (*J*) are given in Hertz (Hz) as absolute values. The multiplicity of the signals is indicated as s, d, t, or m for singlets, doublets, triplets, or multiplets, respectively.

Single crystals suitable for X-ray diffraction were protected with polyisobutylene oil in a glovebox then transferred to the goniometer of an Apex II, KappaCCD, or Proteum diffractometer. The structures were solved and refined using Olex 2 and SHELXL programs.

The HRMS analyses were performed using a Thermo Scientific 12 T LTQ-FT mass spectrometer equipped with an APCI source.

2 Proposed mechanisms for the generation of 4

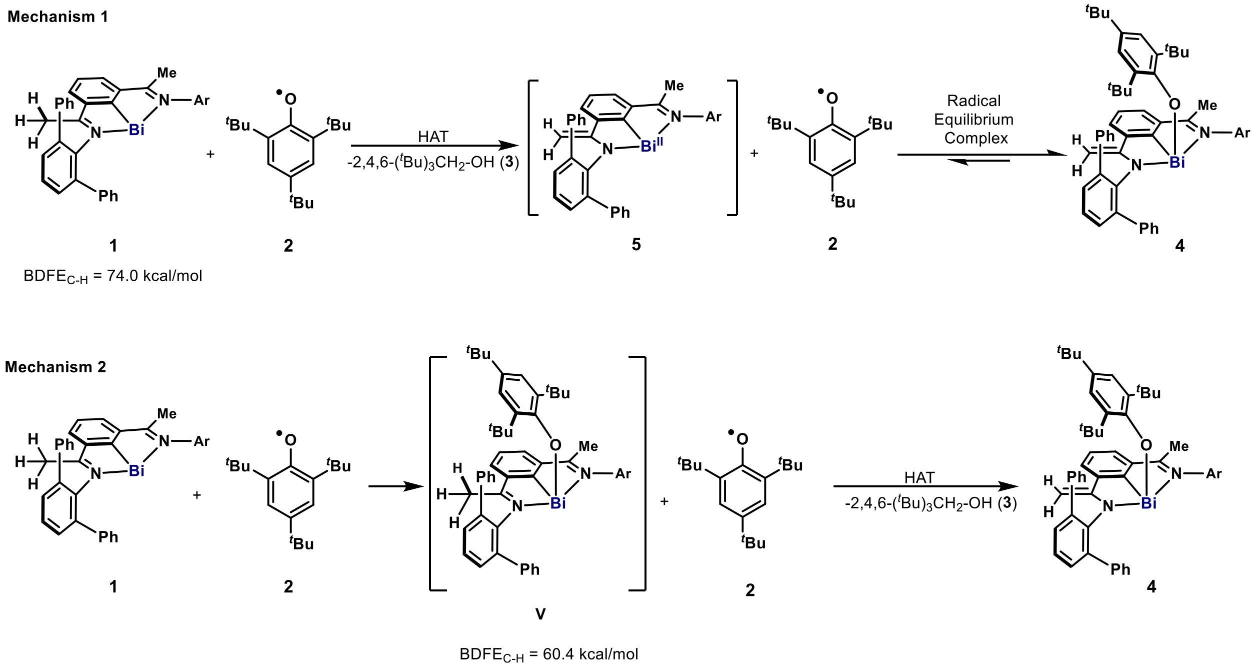

Scheme S1. Proposed mechanisms for the generation of **4**. Mechanism 1 (top): Hydrogen atom abstraction (HAT) from the methyl group of **1** by **2** first, generating **5**, which combines with **2** forming **4**. Mechanism 2 (bottom): **1** was oxidized by **2** first, forming **V**, then one hydrogen atom from the methyl groups of **V** was abstracted by **2**, producing **4**.

The BDFEs of C–H bond in **1** and **V** are 74.0 and 60.4 kcal/mol, respectively. Both of them are smaller than the BDFE of O–H in **3**; hence, 2,4,6-tri-*tert*-butylphenoxyl radical (**2**) can abstract one H atom from either of them. **5** could combine with **2** forming **4**, which indicates that **2** can oxidize bismuth +2 to +3. So **2** should also be able to oxidize bismuth from oxidation state +1 to +2. Thus, mechanism 1 and mechanism 2 are both feasible in the formation of **4**.

3 Preparation of bismuth complexes

3.1 Preparation of **4**

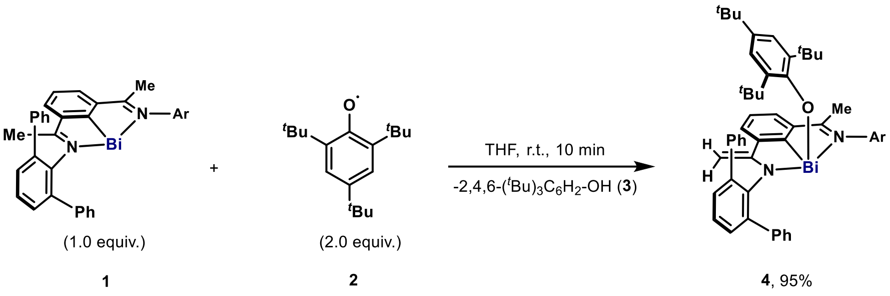

To a solution of **1** (20 mg, 0.024 mmol) in THF (2 mL) was slowly added 2,4,6-tri-*tert*-butylphenoxyl radical, **2**, (12.7 mg, 0.048 mmol) under Ar. The initial brown solution turned quickly to orange. The mixture was stirred for 10 min at 25 °C. The orange solution was then filtered and concentrated (~ 0.5 mL). After layering with *n*-pentane at –24 °C, an orange powder was obtained, which was washed with *n*-pentane and dried under an Ar stream. Yield: 26 mg, 95%.

Note: The complex should be always stored at –24 °C in the dark. It decomposes to unidentified solids in THF after storage at 25 °C overnight. The compound is visible light-sensitive in THF-d_8.

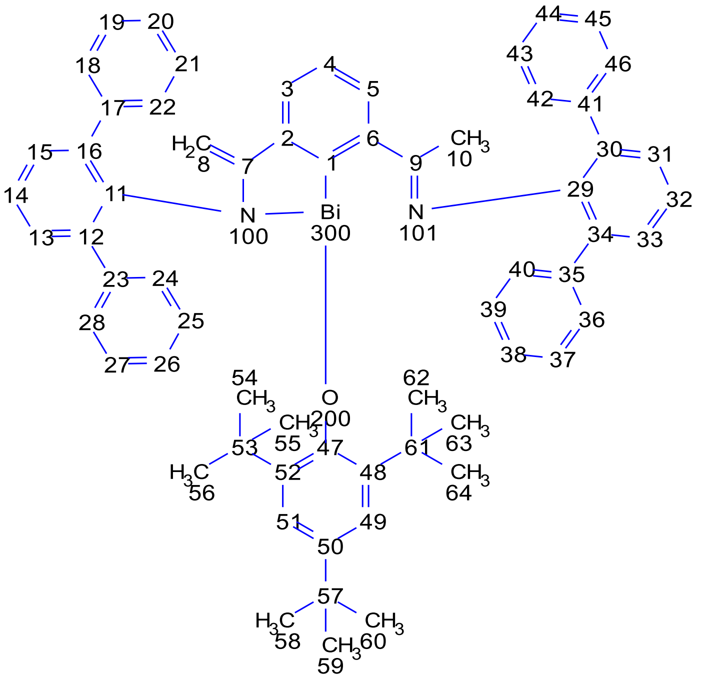

^{1}H NMR - (300 MHz, THF-d_8) δ 7.96 (d, J = 7.9 Hz, 1H, H^{3}), 7.76 (d, J = 9.8 Hz, 2H, $H^{18,22}$), 7.56 (d, J = 8.5 Hz, 1H, H^{5}), 7.41 (dd, J = 7.7 Hz, 1.7 Hz, 1H, H^{15}), 7.38 (dd, J = 7.1, 2.0 Hz, 1H, H^{33}), 7.35 (dd, J = 7.6, 1.7 Hz, 1H, H^{13}), 7.32 (t, J = 7.7 Hz, 1H, H^{4}), 7.31 – 7.28 (m, 1H, H^{32}), 7.27 (dd, J = 7.7, 2.1 Hz, 1H, H^{31}), 7.21 (t, J = 7.6 Hz, 1H, H^{14}), 7.18 (m, 2H, $H^{24,28}$), 7.13 - 7.12 (m, 2H, $H^{49,51}$), 7.10 – 7.09 (m, 2H, $H^{36,40}$), 7.07 – 7.0 (m, 7H, $H^{25,27,37,38,39,42,46}$), 6.90 (d, J = 7.5 Hz, 3H, $H^{19,20,21}$), 6.77 (d, J = 8.8 Hz, 2H, H^{26}), 6.70 (t, J = 7.9 Hz, 1H, H^{44}), 6.61 (t, J = 7.9 Hz, 2H, $H^{43,45}$), 4.05 (s, 1H, $H^{8'}$), 3.47 (d, J = 0.9 Hz, 1H, $H^{8''}$), 1.82 (s, 3H, H^{10}), 1.30 (s, 9H, $H^{58,59,60}$), 0.95 (s, 9H, $H^{62,63,64}$), 0.91 (s, 9H, $H^{54,55,56}$).

^{13}C NMR - (151 MHz, THF-d_8) δ 214.9 (C^{1}), 176.1 (C^{9}), 166.5 (C^{7}), 158.9 (C^{47}), 156.4 (C^{2}), 149.2 (C^{6}), 146.5 (C^{11}), 145.2 (C^{29}), 142.3 (C^{23}), 142.0 (C^{17}), 141.8 (C^{52}), 141.5 (C^{35}), 140.7 (C^{41}), 140.4 (C^{48}), 140.2 (C^{16}), 140.0 (C^{12}), 138.9 (C^{50}), 134.3 (C^{30}), 134.2 (C^{34}), 132.4 (C^{15}), 131.7 (C^{3}), 131.4 (C^{31}), 130.9 (C^{33}), 130.7 ($C^{36,40}$), 130.2 (C^{5}), 130.2 (C^{13}), 129.6 ($C^{25,27}$), 129.5 ($C^{24,28}$), 129.4 ($C^{18,22}$), 129.4 ($C^{42,46}$), 129.1 ($C^{43,45}$), 129.0 ($C^{37,39}$), 128.8 (C^{44}), 128.7 (C^{4}), 128.5 ($C^{19,21}$), 127.9 (C^{26}), 127.4 (C^{38}), 126.8 (C^{20}), 125.5 (C^{32}), 125.2 (C^{14}), 122.4 (C^{49}), 121.9 (C^{51}), 90.1 (C^{8}), 35.6 (C^{53}), 35.6 (C^{61}), 34.8 (C^{57}), 33.6 ($C^{62,63,64}$), 32.5 ($C^{58,59,60}$), 32.1 ($C^{54,55,56}$), 20.6 (C^{10}).

^{15}N NMR - (60.8 MHz, THF-d_8, F1 projection with ^{15}N Signals extracted from the ^{15}N-HMBC) δ -63.54 (N^{101}), -194.2 (N^{100}).

APCI HRMS calc'd for $C_{46}H_{34}BiN_2^+$ 823.25258; found: 823.25145.

APCI HRMS calc'd for $C_{18}H_{29}O^-$ 261.22184; found: 261.22210.

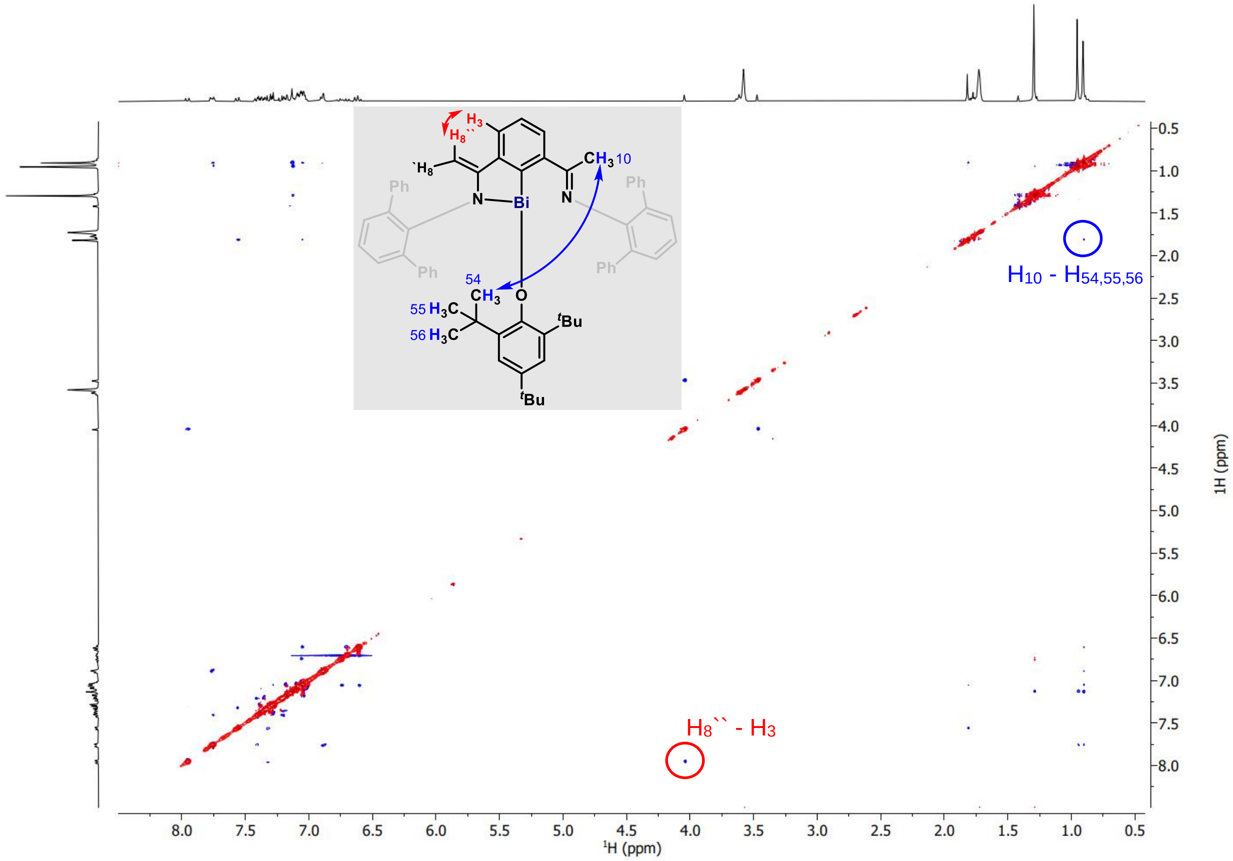

Fig. S1. Relevant ROE-correlations obtained from the 2D EASY-ROESY NMR spectrum of **4** in THF-d_8.

3.2 Preparation of **7**

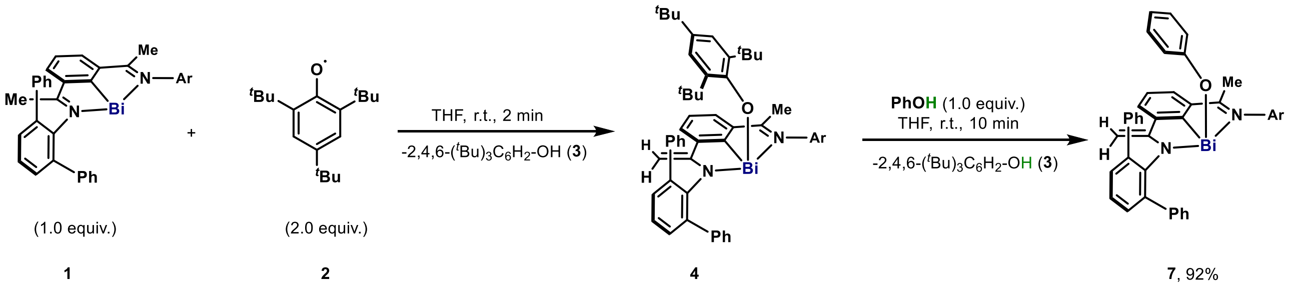

To a solution of **1** (20 mg, 0.024 mmol) in THF (2 mL) was slowly added 2,4,6-tri-*tert*-butylphenoxyl radical, **2**, (12.7 mg, 0.048 mmol) under Ar. The initial brown solution turned quickly to orange. Then PhOH (2.3 mg, 0.024 mmol) was added to the mixture. The mixture was stirred for 10 min at 25 °C. The yellow solution was then filtered and concentrated (~ 1 mL). After layering with *n*-pentane at –24 °C, yellow crystals, which are suitable for X-ray diffraction study, were obtained. The crystals were washed with *n*-pentane and dried under an Ar stream. Yield: 22 mg, 92%.

Note: **7** can be isolated with similar yield from **4** and PhOH. Due to the instability of **4**, the synthesis usually start from **1** and PhOH. The complex was stored at –24 °C in the dark. It is visible light-sensitive in THF-d_8.

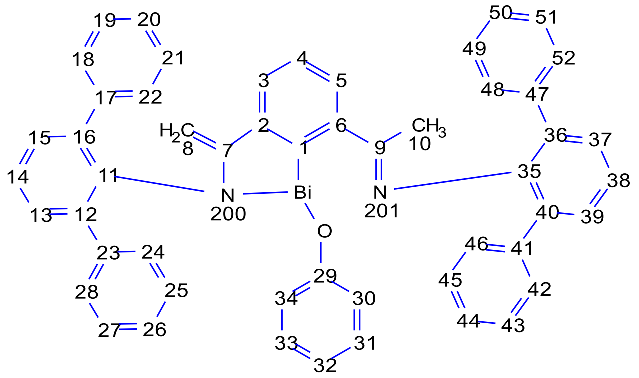

^{1}H NMR - (600 MHz, THF-d_8) δ 7.92 (dd, J = 8.0, 0.9 Hz, 1H, H^{3}), 7.80 – 7.74 (m, 5H, $H^{5,18,22,42,46}$), 7.46 (dd, J = 7.6, 1.6 Hz, 1H, H^{39}), 7.37 (t, J = 7.6 Hz, 1H, H^{38}), 7.35 - 7.28 (m, 4H, $H^{4,15,26,37}$), 7.27 (dd, J = 7.7, 1.7 Hz, 1H, H^{13}), 7.24 – 7.15 (m, 8H, $H^{14,24,25,27,28,43,45,50}$), 7.14 – 7.11 (m, 1H, H^{44}), 7.09 (t, J = 7.7 Hz, 2H, $H^{49,51}$), 7.01 – 6.92 (m, 3H, $H^{19,20,21}$), 6.90 – 6.86 (m, 2H, $H^{48,52}$), 6.69 (m, 2H, $H^{31,33}$), 6.44 (tt, J = 7.3, 1.1 Hz, 1H, H^{32}), 5.70 – 5.55 (m, 2H, $H^{30,34}$), 4.04 (s, 1H, $H^{8'}$), 3.36 (s, 1H, $H^{8''}$), 1.91 (s, 3H, H^{10}).

^{13}C NMR - (151 MHz, THF-d_8) δ 202.4 (C^{1}), 174.3 (C^{9}), 163.2 (C^{29}), 162.3 (C^{7}), 152.7 (C^{2}), 146.8 (C^{11}), 144.7 (C^{6}), 144.2 (C^{35}), 143.3 (C^{17}), 142.1 (C^{23}), 141.0 (C^{41}), 141.0 (C^{16}), 140.5 (C^{47}), 139.5 (C^{12}), 135.4 (C^{36}), 135.3 (C^{40}), 132.3 (C^{15}), 131.7 (C^{3}), 131.4 (C^{39}), 130.8 ($C^{42,46}$), 130.6 (C^{5}), 130.5 (C^{13}), 130.2 ($C^{48,52}$), 130.1 (C^{37}), 129.7 ($C^{24,28}$), 129.5 ($C^{18,22}$), 129.4 ($C^{4,25,27}$), 129.1 ($C^{49,51}$), 129.0 ($C^{31,33}$), 129.0 (C^{50}), 128.9 ($C^{43,45}$), 128.5 (C^{26}), 127.8 ($C^{19,21}$), 127.4 (C^{44}), 126.3 (C^{38}), 126.2 (C^{20}), 124.4 (C^{14}), 121.6 ($C^{30,34}$), 119.6 (C^{32}), 87.0 (C^{8}), 19.3 (C^{10}).

^{15}N NMR - (60.8 MHz, THF-d_8, F1 projection with ^{15}N Signals extracted from the ^{15}N-HMBC) δ -83.5 (N^{201}), -211.8 (N^{200}).

APCI HRMS calc'd for $C_{52}H_{39}BiN_2O$ [M]$^+$ 916.28662; found: 916.28607.

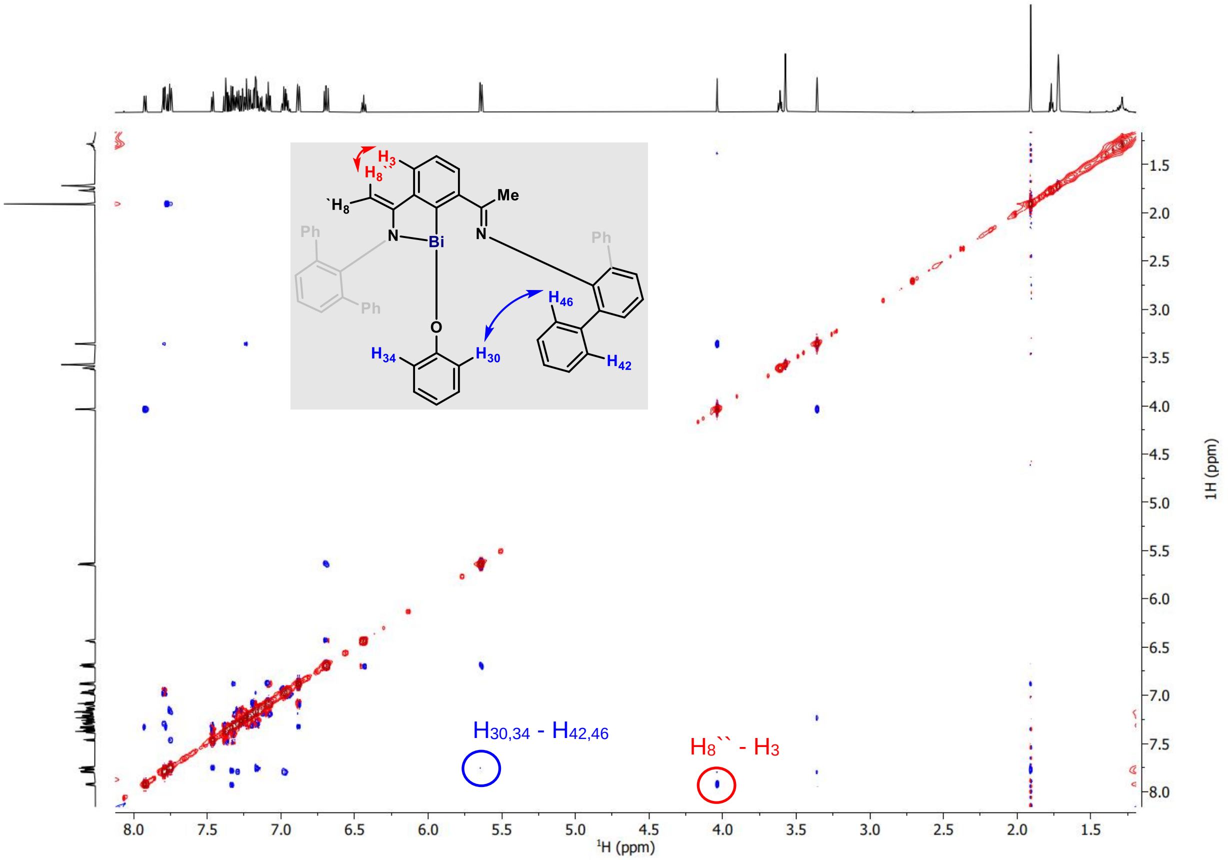

Fig. S2. Relevant ROE-correlations obtained from the 2D EASY-ROESY NMR spectrum of **7** in THF-d_8.

3.3 Preparation of **9**

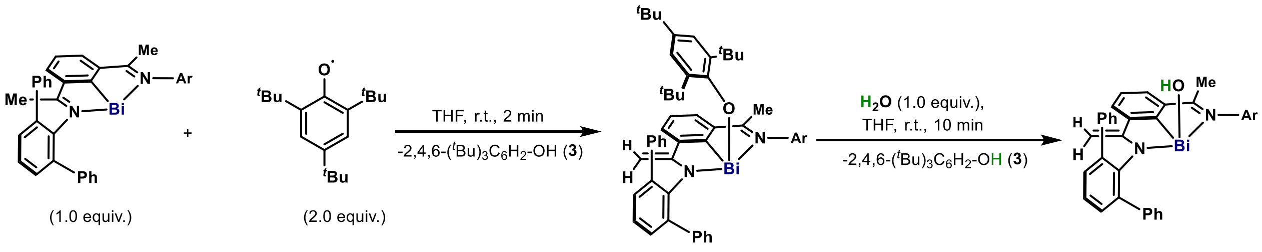

1 2 4 **9**, 86%

To a solution of **1** (20 mg, 0.024 mmol) in THF (2 mL) was added 2,4,6-tri-*tert*-butylphenoxyl radical, **2**, (12.7 mg, 0.048 mmol) slowly under Ar. The initial brown solution turned quickly to orange. Then H_2O (0.22 µL, 0.025 mmol) was added to the mixture. The mixture was stirred for 10 min at 25 °C. The yellow solution was then filtered and concentrated (~ 1 mL). After layering with *n*-pentane at -24 °C, yellow crystals were obtained. The crystals were washed with *n*-pentane and dried under an Ar stream. Yield: 19 mg, 86%.

Note: **9** can be isolated with similar yield from **4** and H_2O. Due to the instability of **4**, the synthesis usually start from **1** and H_2O. The complex was stored at 25 °C under Ar. It is visible light-sensitive in THF-d_8.

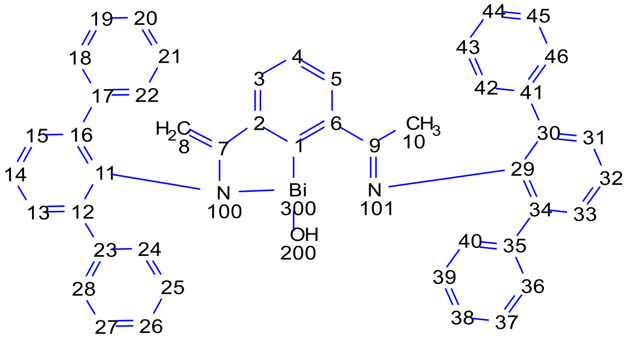

^{1}H NMR - (600 MHz, THF-d_8) δ 7.82 (d, J = 8.0 Hz, 1H, H^{3}), 7.73 – 7.62 (m, 5H, $H^{5,24,28,36,40}$), 7.38 (dd, J = 7.5, 1.7 Hz, 1H, H^{33}), 7.34 (dd, J = 7.5, 1.7 Hz, 1H, H^{31}), 7.29 (dd, J = 14.8, 7.3 Hz, 1H, H^{32}), 7.28 – 7.20 (m, 8H, $H^{4,13,15,18,22,43,44,45}$), 7.17 – 7.12 (m, 4H, $H^{14,19,20,21}$), 7.10 – 7.05 (m, 8H, $H^{25,26,27,37,38,39,42,46}$), 4.14 (s, 1H, $H^{8'}$), 3.26 (s, 1H, $H^{8''}$), 1.86 (s, 3H, H^{10}), 0.92 (s, 1H, OH^{200}).

^{13}C NMR - (151 MHz, THF-d_8) δ 194.9 (C^{1}), 173.7 (C^{9}), 163.8 (C^{7}), 152.9 (C^{2}), 147.0 (C^{11}), 144.3 (C^{6}), 144.1 (C^{29}), 143.0 (C^{23}), 142.7 (C^{17}), 142.3 (C^{12}), 141.0 (C^{16}), 140.8 (C^{41}), 140.6 (C^{35}), 135.6 (C^{34}), 135.2 (C^{30}), 131.7 (C^{3}), 131.4 (C^{13}), 131.1 (C^{33}), 130.9 ($C^{36, 40}$), 130.7 (C^{15}), 130.4 ($C^{5,42,46}$), 130.1 (C^{31}), 130.0 ($C^{24,28}$), 129.4 ($C^{18,22}$), 129.0 ($C^{43,45}$), 128.8 ($C^{37,39}$), 128.7 (C^{44}), 128.5 ($C^{19,21}$), 128.5 (C^{4}), 128.2 ($C^{25,27}$), 127.5 (C^{20}), 127.3 (C^{38}), 126.9 (C^{26}), 126.0 (C^{32}), 124.5 (C^{14}), 84.6 (C^{8}), 19.1 (C^{10}).

^{15}N NMR - (60.8 MHz, THF-d_8, F1 projection with ^{15}N Signals extracted from the ^{15}N-HMBC) δ -84.1(N^{101}), -225.6 (N^{100}).

APCI HRMS calc'd for $C_{46}H_{35}BiN_2O^+$ $[M]^+$: 840.25532; found: 840.25413.

3.4 Preparation of **11**

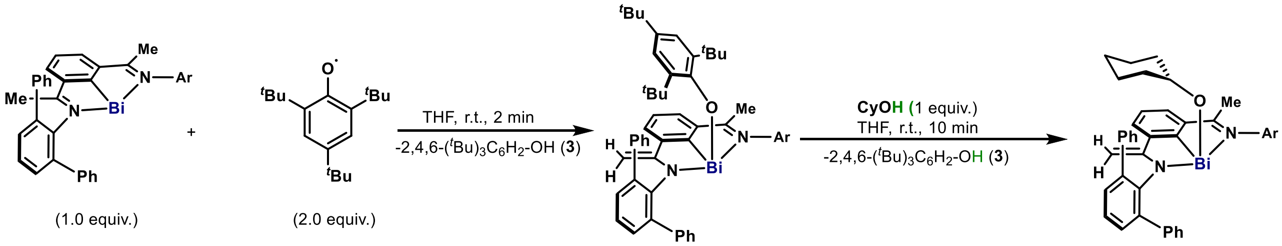

1 **2** **4** **11**, 99%

To a solution of **1** (20 mg, 0.024 mmol) in THF (2 mL) was added 2,4,6-tri-*tert*-butylphenoxyl radical, **2**, (12.7 mg, 0.048 mmol) slowly under Ar. The initial brown solution turned quickly to orange. Then CyOH (2.4 mg, 0.024 mmol) was added to the mixture. The mixture was stirred for 10 min at 25 °C. The yellow solution was then filtered and concentrated (~ 1 mL). After layering with *n*-pentane at -24 °C, yellow crystals were obtained. The crystals were washed with *n*-pentane and dried under an Ar stream. Yield: 24 mg, 99%.

Note: **11** can be isolated with similar yield from **4** and CyOH. Due to the instability of **4**, the synthesis usually start from **1** and CyOH. The complex was stored at -24 °C in the dark. It is visible light-sensitive in THF-d_8.

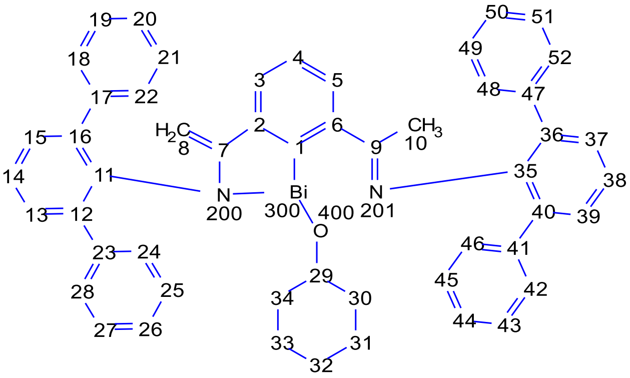

^{1}H NMR - (600 MHz, THF-d_8) δ 7.87-7.85 (m, 2H, $H^{18,22}$), 7.80– 7.79 (m, 2H, $H^{48,52}$), 7.75 (dd, J = 8.1 Hz, J = 0.9 Hz, 1H, H^{3}), 7.65 (dd, J = 7.5 Hz, 0.8 Hz, 1H, H^{5}), 7.42 (dd, J = 7.5 Hz, 1.6 Hz, 1H, H^{37}), 7.38 – 7.32 (m, 5H, $H^{24,26,28,38,39}$), 7.27 – 7.23 (m, 5H, $H^{4,13,25,27,44}$), 7.20 – 7.12 (m, 6H, $H^{15,43,45,49,50,51}$), 7.09 – 7.05 (m, 3H, $H^{14,42,46}$), 6.96 – 6.93 (m, 3H, $H^{19,20,21}$), 3.93 (s, 1H, $H^{8'}$), 3.55 (m, 1H, H^{29}), 3.26 (d, 1H, $H^{8''}$), 1.83 (s, 3H, H^{10}), 1.29 (m, 2H, $H^{31,33}$), 1.18 – 1.12 (m, 3H, $H^{30,32,34'}$), 0.83 – 0.78 (m, 3H, $H^{31',32',33}$), 0.66 – 0.62 (m, 2H, $H^{30',34}$).

^{13}C NMR - (151 MHz, THF-d_8) δ 195.1 (C^1), 173.6 (C^9), 161.3 (C^7), 152.3 (C^2), 147.2 (C^{11}), 144.3 (C^6), 144.1 (C^{35}), 143.5 (C^{17}), 142.4 (C^{23}), 140.9 (C^{47}), 140.8 (C^{16}), 140.6 (C^{41}), 139.3 (C^{12}), 135.3 (C^{40}), 135.1 (C^{36}), 132.0 (C^{15}), 131.2 ($C^{48,52}$), 131.1 (C^3), 131.1 (C^{37}), 130.1 ($C^{42,46}$), 130.0 (C^5), 130.0 (C^{13}), 129.8 (C^{39}), 129.7 ($C^{24,28}$), 129.6 ($C^{18,22}$), 129.3 ($C^{25,27}$), 129.2 ($C^{43,45}$), 128.9 (C^{44}), 128.8 (C^4), 128.8 ($C^{49,51}$), 128.4 (C^{26}), 127.7 ($C^{19,21}$), 127.3 (C^{50}), 126.1 (C^{38}), 126.1 (C^{20}), 123.8 (C^{14}), 84.6 (C^8), 74.6 (C^{29}), 38.9 (C^{34}), 38.8 (C^{30}), 26.7 (C^{32}), 24.9 ($C^{31,33}$), 19.1 (C^{10}).

^{15}N NMR - (60.8 MHz, THF-d_8, F1 projection with ^{15}N Signals extracted from the ^{15}N-HMBC) δ -84.6 (N^{201}), -225.5 (N^{200}).

APCI HRMS calc'd for $C_{52}H_{45}BiN_2O$ $[M]^+$ 922.33357; found: 922.33304.

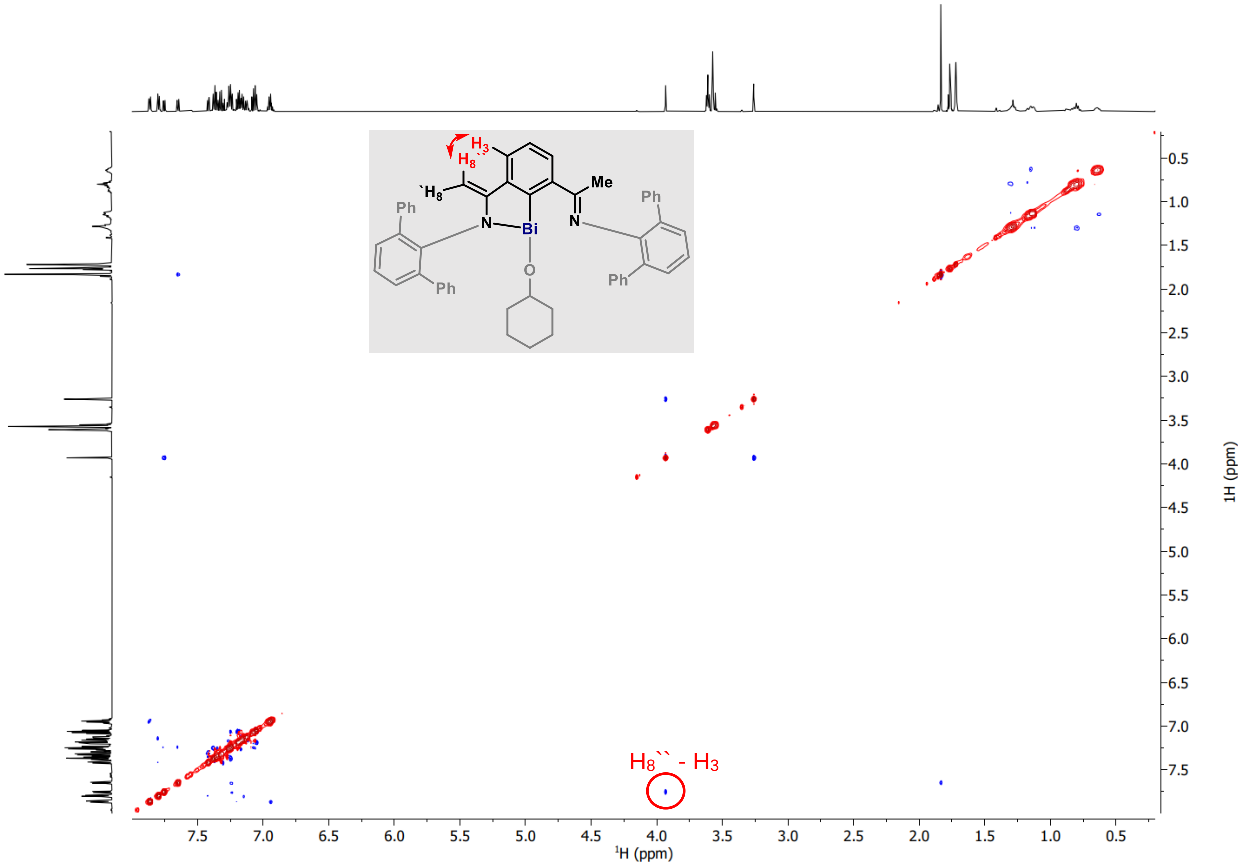

Fig. S3. Important ROE-correlations obtained from the 2D EASY-ROESY NMR spectrum of **11** in THF-d_8.

3.5 Preparation of **13**

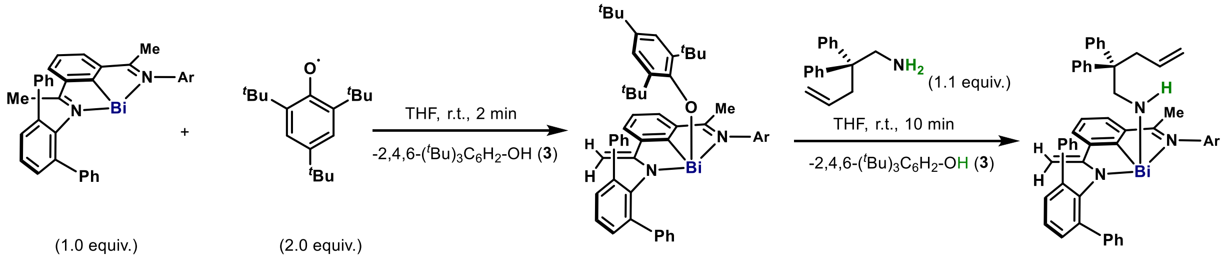

1 **2** **4** **13**, 78 %

To a solution of **1** (20 mg, 0.024 mmol) in THF (2 mL) was added 2,4,6-tri-*tert*-butylphenoxyl radical, **2**, (12.7 mg, 0.048 mmol) slowly under Ar. The initial brown solution turned quickly to orange. Then 2,2-diphenyl-4-penton-1-amine (6.3 mg, 0.026 mmol) was added to the mixture. The mixture was stirred for 10 min at 25 °C. The yellow solution was then filtered and concentrated (~ 0.5 mL). After layering with *n*-pentane at -24 °C, a yellow solid was obtained. The solid was washed with *n*-pentane and dried under an Ar stream. Yield: 20 mg, 78%.

Note: **13** can be isolated with similar yield from **4** and 2,2-diphenyl-4-penton-1-amine. Due to the instability of **4**, the synthesis usually start from **1** and 2,2-diphenyl-4-penton-1-amine. The complex was stored at -24 °C in the dark. It is extremely moisture sensitive, and visible light sensitive in THF-d_8. Due to the formation of **1** as a side product, excess of amine is needed for the full conversion of **4**. Excess of amine crystalized out together with **13**.

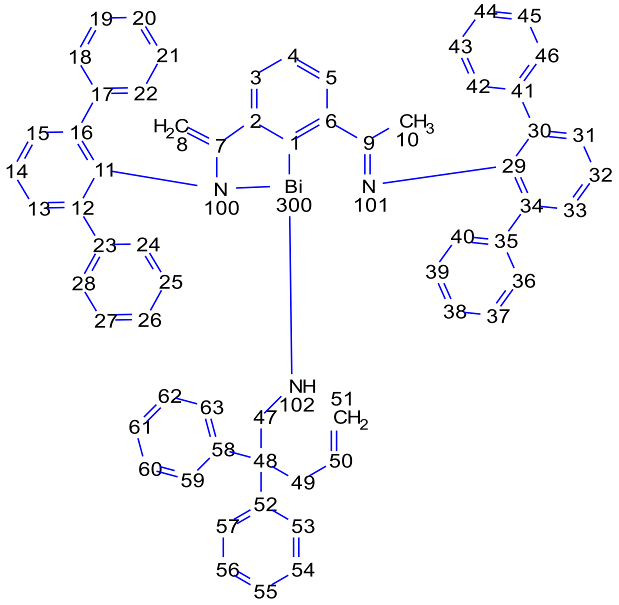

^{1}H NMR - (600 MHz, THF-d_8) δ 7.68 (dd, J = 8.1 Hz, 0.9 Hz, 1H, H^{3}), 7.53 – 7.52 (m, 3H, $H^{5,18,22}$), 7.50 – 7.47 (m, 1H, H^{31}), 7.44 – 7.44 (m, 2H, $H^{32,33}$), 7.38 – 7.36 (m, 2H, $H^{24,28}$), 7.31 – 7.24 (m, 6H, $H^{13,15,26,38,42,46}$), 7.22 – 7.19 (m, 4H, $H^{4,14,25,27}$), 7.16 – 7.13 (m, 2H, $H^{37,39}$), 7.09 – 7.07 (m, 1H, H^{44}), 7.06 – 7.04 (m, 2H, $H^{36,40}$), 7.01 – 6.98 (m, 2H, $H^{43,45}$), 6.97 – 6.94 (m, 1H, H^{20}), 6.91 – 6.85 (m, 8H, $H^{19,21,54,55,56,60,61,62}$), 6.62 – 6.60 (m, 4H, $H^{53,57,59,63}$), 5.05 – 4.97 (m, 1H, H^{50}), 4.71 – 4.66 (m, 1H, H^{51}), 4.44 (ddt, J = 17.1 Hz, 2.6 Hz, 1.3 Hz, 1H, $H^{51`}$), 3.93 (s, 1H, $H^{8´}$), 3.39 (dd, $J$ = 12.4 Hz, 8.1 Hz, 1H, $H^{47}$), 3.33 (dd, $J$ = 12.4, 9.9 Hz, 1H, $H^{47`}$), 3.13 (s, 1H, $H^{8´´}$), 2.69 – 2.64 (m, 1H, H^{49}), 2.56 (dd, J = 12.9 Hz, 6.8 Hz, 1H, $H^{49`}$), 2.33 (dd, J = 9.9 Hz, 8.1 Hz, 1H, NH^{102}), 1.81 (s, 3H, H^{10}).

^{13}C NMR - (151 MHz, THF-d_8) δ 186.0 (C^{1}), 173.9 (C^{9}), 161.3 (C^{7}), 152.3 (C^{2}), 148.1 (C^{52}), 147.9 (C^{58}), 147.5 (C^{11}), 145.3 (C^{29}), 144.5 (C^{6}), 143.3 (C^{17}), 142.3 (C^{23}), 141.6 (C^{16}), 140.8 (C^{41}), 140.4 (C^{35}), 140.0 (C^{12}), 136.2 (C^{50}), 135.5 (C^{34}), 134.5 (C^{30}), 132.0 (C^{15}), 131.5 (C^{31}), 131.0 (C^{13}), 130.9 (C^{3}), 130.7 (C^{33}), 130.3 ($C^{42,46}$), 130.2 ($C^{36,40}$), 130.0 (C^{5}), 129.7 ($C^{24,28}$), 129.5 ($C^{18,22}$), 129.2 (C^{38}), 129.2 ($C^{25,27}$), 129.0 ($C^{37,39}$), 128.9 ($C^{43,45}$), 128.8 ($C^{59,63}$), 128.8 ($C^{53,57}$), 128. (C^{4}), 128.3 ($C^{60,62}$), 128.2 ($C^{54,56}$), 128.2 (C^{26}), 127.7 ($C^{19,21}$), 127.2 (C^{44}), 126.2 (C^{20}), 126.1 (C^{32}), 125.9 (C^{55}), 125.9 (C^{61}), 124.0 (C^{14}), 117.5 (C^{51}), 83.7 (C^{8}), 54.0 (C^{48}), 53.6 (C^{47}), 40.5 (C^{49}), 19.6 (C^{10}).

^{15}N NMR - (60.8 MHz, THF-d_8, F1 projection with ^{15}N Signals extracted from the ^{15}N-HMBC) δ -84.5 (N^{101}), -243.9 (N^{100}), -321.8 (N^{102}).

APCI HRMS calc'd for $C_{63}H_{52}BiN_3$ 1059.39651; found: 1059.39597.

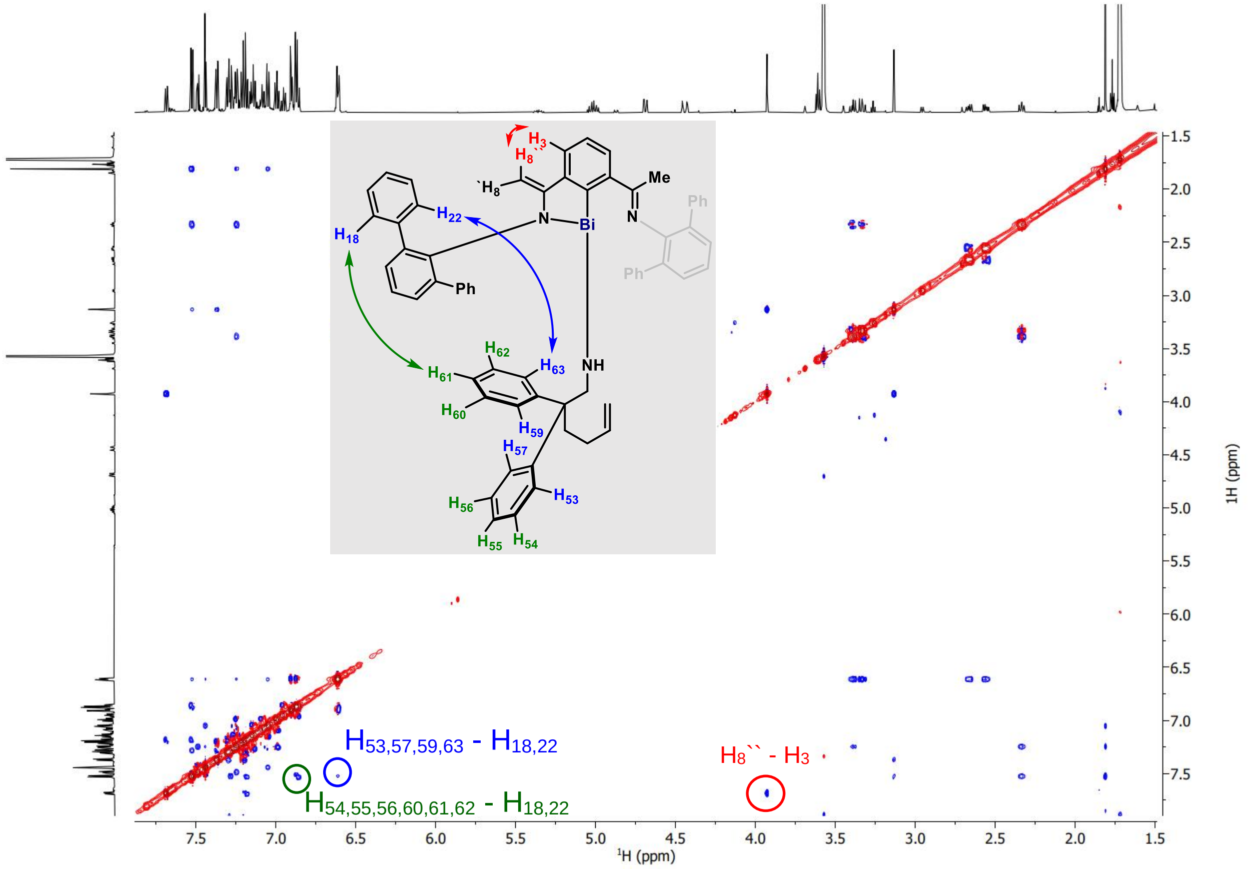

Fig. S4. Relevant ROE-correlations obtained from the 2D EASY-ROESY NMR spectrum of **13** in THF-d_8.

3.6 Preparation of **15**

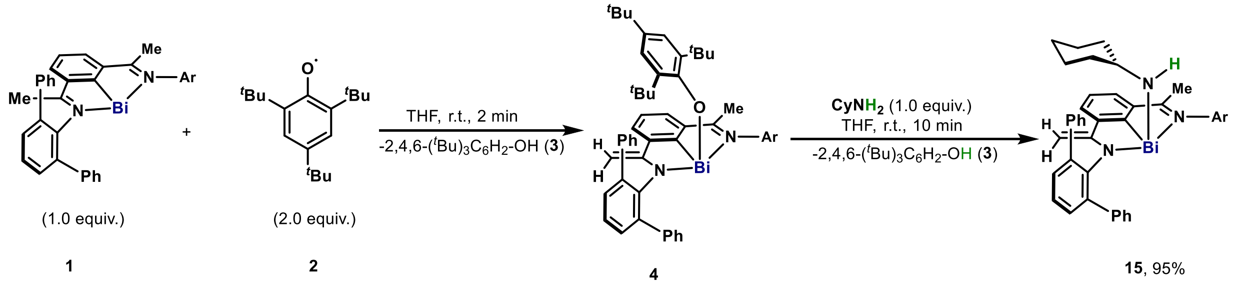

To a solution of **1** (20 mg, 0.024 mmol) in THF (2 mL) was added 2,4,6-tri-*tert*-butylphenoxyl radical, **2**, (12.7 mg, 0.048 mmol) slowly under Ar. The initial brown solution turned quickly to orange. Then $CyNH_2$

(2.4 mg, 0.024 mmol) was added to the mixture. The mixture was stirred for 10 min at 25 °C. The yellow solution was then filtered and concentrated (~ 0.5 mL). After layering with *n*-pentane at -24 °C, yellow crystals, which are suitable for X-ray diffraction study, were obtained. The crystals were washed with *n*-pentane and dried under an Ar stream. Yield: 22 mg, 95%.

Note: **15** can be isolated with similar yield from **4** and $CyNH_2$. Due to the instability of **4**, the synthesis usually start from **1** and $CyNH_2$. The complex was stored at -24°C in the dark. It is extremely moisture sensitive, and visible light sensitive in THF-d_8.

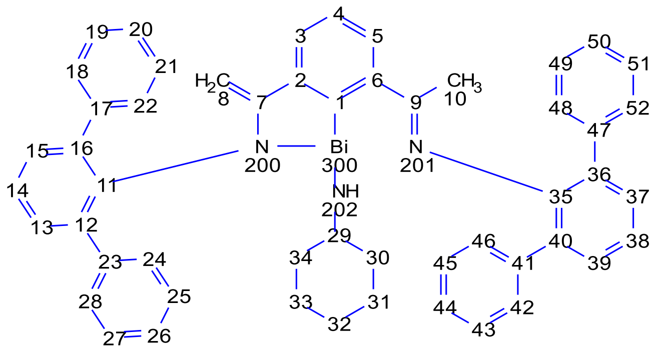

^{1}H NMR - (600 MHz, THF-d_8) δ 7.80 (d, J = 7.1 Hz, 1H, H^{3}), 7.77 – 7.75 (m, 2H, $H^{42,46}$), 7.72 – 7.70 (m, 2H, $H^{18,22}$), 7.56 (dd, J = 7.6, 0.8 Hz, 1H, H^{5}), 7.38 (dd, J = 7.5, 1.6 Hz, 1H, H^{39}), 7.34 (dd, J = 4.2, 1.8 Hz, 1H, H^{37}), 7.33 (dd, J = 3.9, 1.8 Hz, 1H, H^{15}), 7.30 – 7.25 (m, 3H, $H^{4,38,50}$), 7.25 – 7.20 (m, 4H, $H^{24,28,49,51}$), 7.17 – 7.09 (m, 10H, $H^{13,14,19,20,21,25,26,27,48,52}$), 7.07 – 7.06 (m, 3H, $H^{43,44,45}$), 4.24 (s, 1H, $H^{8'}$), 3.20 (s, 1H, $H^{8''}$), 2.42 (tdt, J = 10.1, 6.0, 3.7 Hz, 1H, H^{29}), 1.80 (s, 3H, H^{10}), 1.40 – 1.21 (m, 4H, $H^{31,32,33,34}$), 1.09 (d, J = 6.0 Hz, 1H, H^{202}), 0.98 (m, 1H, H^{30}), 0.91 - 0.84 (m, 1H, $H^{31`}$), 0.81 – 0.77 (m, 1H, $H^{33`}$), 0.70 – 0.65 (m, 1H, $H^{32`}$), 0.38 – 0.31 (m, 1H, $H^{30`}$), 0.25 – 0.19 (m, 1H, $H^{34`}$).

^{13}C NMR - (151 MHz, THF-d_8) δ 182.8 (C^{1}), 173.1 (C^{9}), 163.9 (C^{7}), 152.5 (C^{2}), 147.7 (C^{11}), 144.5 (C^{6}), 144.2 (C^{35}), 142.9 (C^{17}), 142.6 (C^{23}), 142.2 (C^{12}), 142.1 (C^{16}), 141.0 (C^{47}), 140.8 (C^{41}), 135.7 (C^{36}), 134.9 (C^{40}), 131.8 (C^{15}), 131.2 (C^{13}), 131.2 (C^{39}), 131.1 ($C^{42,46}$), 131.1 (C^{3}), 130.4 ($C^{48,52}$), 130.2 (C^{5}), 130.1 (C^{37}), 129.9 ($C^{18,22}$), 129.7 ($C^{24,28}$), 129.1 ($C^{49,51}$), 128.8 (C^{50}), 128.7 ($C^{43,45}$), 128.4 ($C^{19,21}$), 128.2 ($C^{25,27}$), 128.2 (C^{4}), 127.3 ($C^{20,44}$), 127.2 (C^{26}), 126.0 (C^{38}), 124.4 (C^{14}), 83.6 (C^{8}), 55.5 (C^{29}), 41.6 (C^{30}), 39.1 (C^{34}), 26.6 (C^{32}), 26.4 (C^{33}), 26.2(C^{31}), 19.5 (C^{10}).

^{15}N NMR - (60.8 MHz, THF-d_8, F1 projection with ^{15}N Signals extracted from the ^{15}N-HMBC) δ -84.6 (N^{201}), -250.9 (N^{200}), -295.6 (N^{202}).

APCI HRMS calc'd for $C_{46}H_{34}BiN_2^+$ 823.25258; found: 823.25182. CyNH$^-$ was lost in the HRMS measurement.

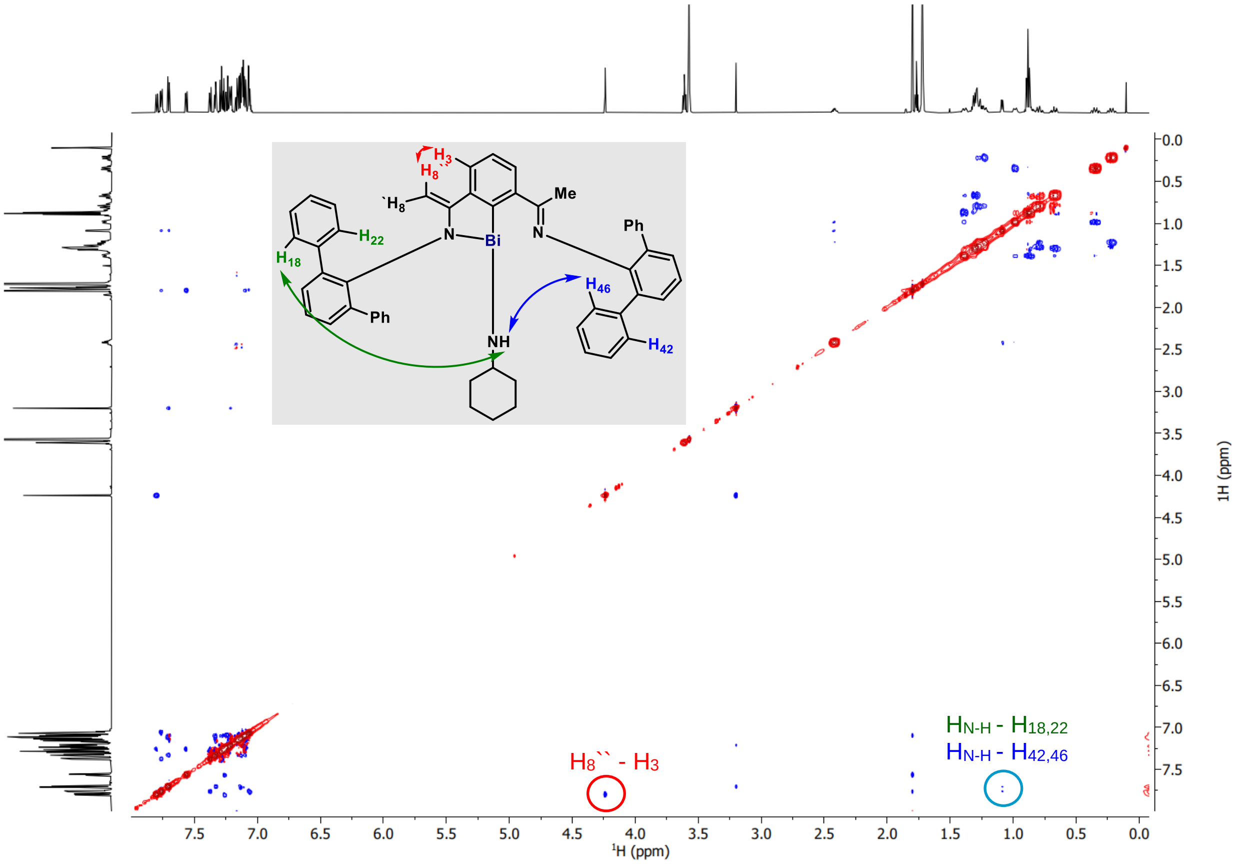

Fig. S5. Relevant ROE-correlations obtained from the 2D EASY-ROESY NMR spectrum of **15** in THF-d_8.

3.7 Preparation of **17**

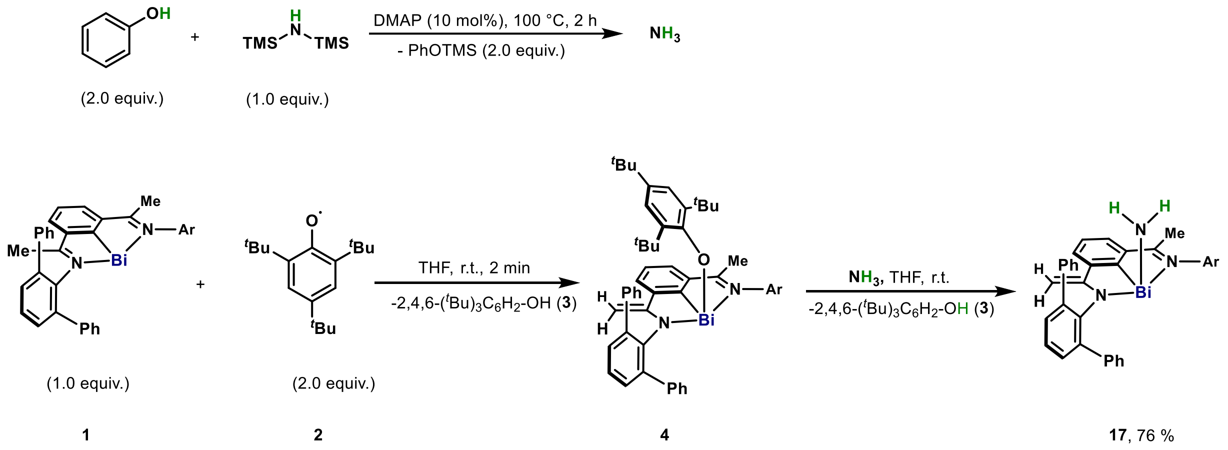

PhOH (5.0 mg, 0.053 mmol), 1,1,1,3,3,3-hexamethyldisilazane (4.3 mg, 0.027 mmol), and 4-dimethylaminopyridine (0.3 mg, 0.0027 mmol) were added to one chamber in a two-chamber system. In the other chamber, **1** (22 mg, 0.027 mmol) was dissolved in THF (2 mL), and 2,4,6-tri-*tert*-butylphenoxyl radical, **2**, (12.7 mg, 0.048 mmol) was added slowly under Ar. The first chamber was stirred for 2 h at 100 °C, while the second chamber was stirred at 25 °C in a water bath. The yellow solution in the second chamber was filtered and concentrated (~ 0.5 mL). After layering with *n*-pentane at -24 °C, yellow crystals, which are suitable for X-ray diffraction study, were obtained. The crystals were washed with *n*-pentane and dried under an Ar stream. Yield: 17 mg, 76%.

Note: **17** can be isolated with similar yield from **4** and NH_3. Due to the instability of **4**, the synthesis usually start from **1** and NH_3. Complex **17** can also be instantly formed using commercially available NH_3 gas. However, due to the residence of H_2O from gas, there was always a certain amount of **9** formed. The complex was stored at –24 °C in the dark. It is extremely moisture sensitive, and visible light sensitive in THF-d_8.

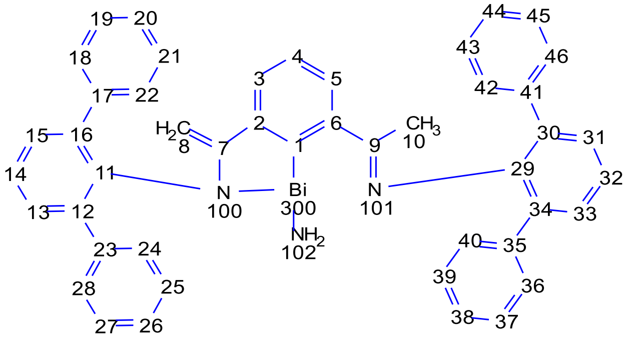

^{1}H NMR - (600 MHz, THF-d_8) δ 7.68 (d, J = 7.9 Hz, 1H, H^{3}), 7.76 – 7.65 (m, 2H, $H^{36,40}$), 7.60 (d, J = 7.5 Hz, 1H, H^{5}), 7.56 – 7.52 (m, 2H, $H^{24,28}$), 7.36 – 7.33 (m, 2H, $H^{31.33}$), 7.31 – 7.26 (m, 3H, $H^{32,43,45}$), 7.23 (m, 3H, $H^{4,13,44}$), 7.17 – 7.07 (m, 14H, $H^{14,15,18,19,21,22,25,26,27,37,38,39,42,46,}$), 7.02 (m, 1H, H^{20}), 4.16 (s, 1H, $H^{8´}$), 3.24 (s, 1H, $H^{8´´}$), 1.84 (s, 3H, H^{10}), 0.11 (s, 2H, H^{102}).

^{13}C NMR - (151 MHz, THF-d_8) δ 181.6 (C^{1}), 173.0 (C^{9}), 163.1 (C^{7}), 153.1 (C^{2}), 147.8 (C^{11}), 144.2 (C^{6}), 144.2 (C^{29}), 143.7 (C^{23}), 143.1 (C^{17}), 142.5 (C^{12}), 141.7 (C^{16}), 141.1 (C^{41}), 140.8 (C^{35}), 135.4 ($C^{30,34}$), 131.5 (C^{3}), 131.2 (C^{33}), 131.2 (C^{13}), 131.1 (C^{15}), 130.9 ($C^{36,40}$), 130.6 ($C^{42,46}$), 130.4 (C^{5}), 130.4 (C^{31}), 130.2 ($C^{24,28}$), 129.3 ($C^{18,22}$), 129.0 ($C^{43,45}$), 128.8 ($C^{37,39}$), 128.6 ($C^{25,27}$), 128.5 (C^{44}), 128.1 (C^{4}), 128.1 ($C^{19,21}$), 127.4 (C^{38}), 127.3 (C^{26}), 126.6 (C^{20}), 126.0 (C^{32}), 124.2 (C^{14}), 83.6 (C^{8}), 19.3 (C^{10}).

^{15}N NMR - (60.8 MHz, THF-d_8, F1 projection with ^{15}N Signals extracted from the ^{15}N-HMBC) δ -84.2 (N^{101}), -247.7 (N^{100}), -346.7 (N^{102}).

APCI HRMS calc'd for $C_{46}H_{34}BiN_2$ 823.25258; found: 823.25188. NH_2^- was lost in the HRMS measurement.

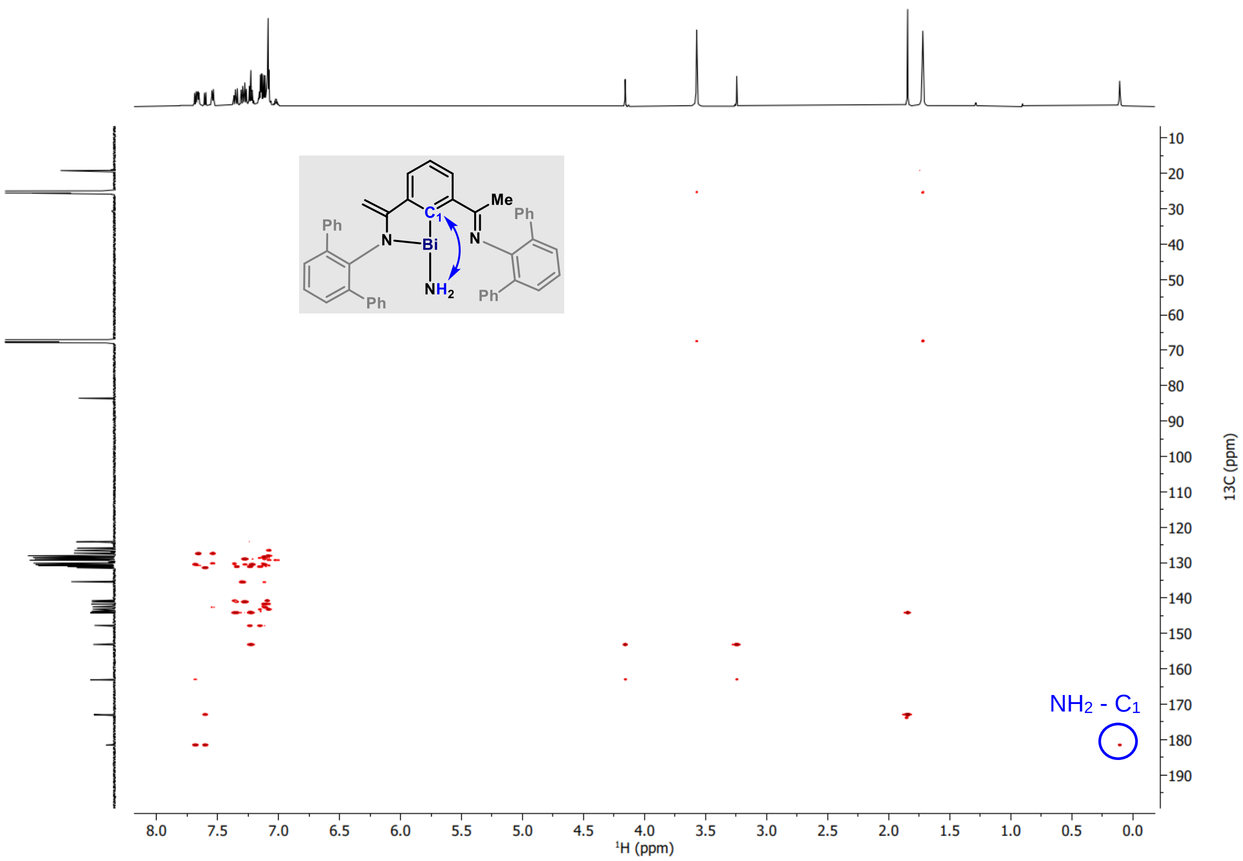

Fig. S6. Correlation between N*H* and C1 in HMBC spectrum of **17** in THF-d_8.

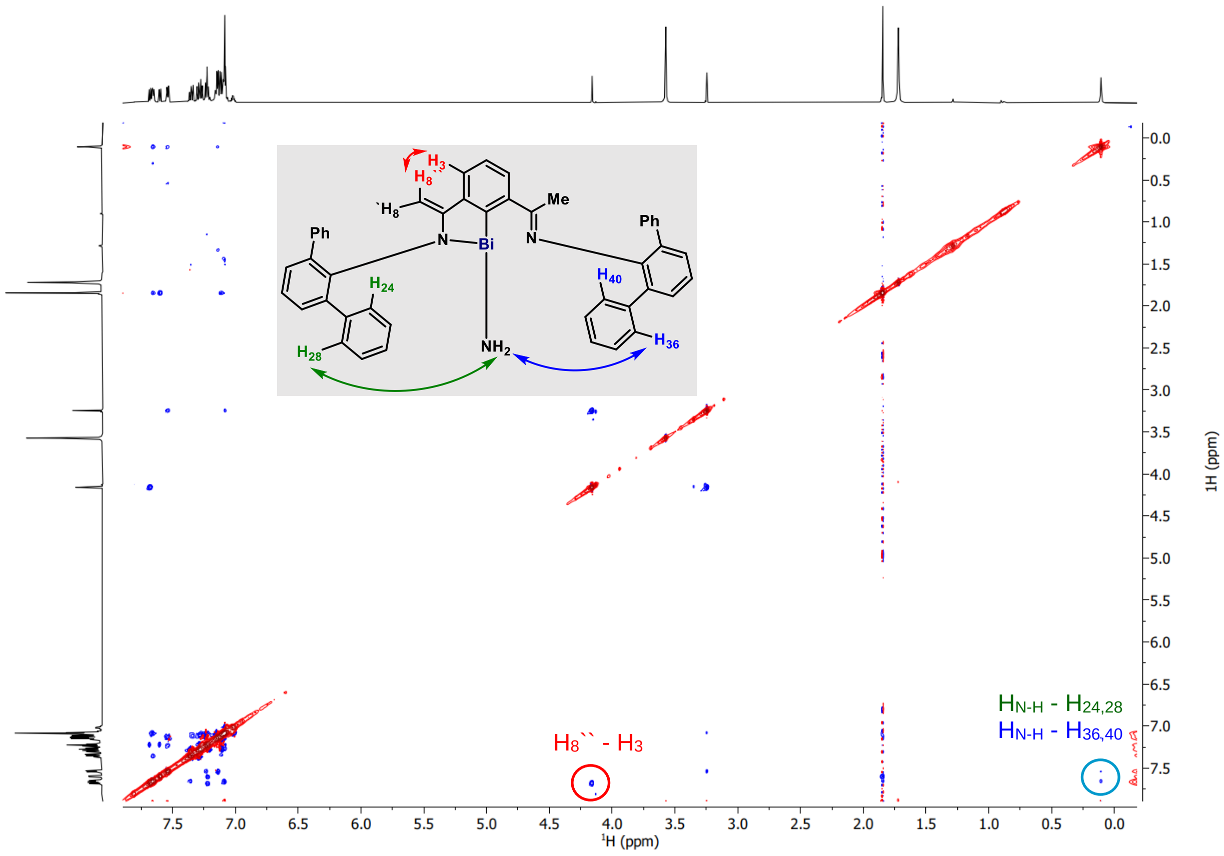

Fig. S7. Relevant ROE-correlations obtained from the 2D EASY-ROESY NMR spectrum of **17** in THF-d_8.

4 Reactivity of 15 and 17 towards H^+, H·, and H^-

4.1 The reaction of **15** and **17** with PhOH

15 (3.0 mg, 3.3 µmol) was dissolved in 0.6 mL THF-d_8 in a J-Young NMR tube. Then PhOH (0.3 mg, 3.3 µmol) was added at the top of the NMR tube. The tube was kept in a dry ice ethanol bath for 5 min. Then the mixture was mixed at this temperature, and the J-Young tube was injected into a 500 MHz NMR spectrometer, which was precooled to -80 °C. In the first recorded proton NMR spectrum, **15** was consumed completely, while **7** and $CyNH_2$ were formed in 82% NMR yield. 84% NMR yield was obtained in the reaction of **17** and PhOH.

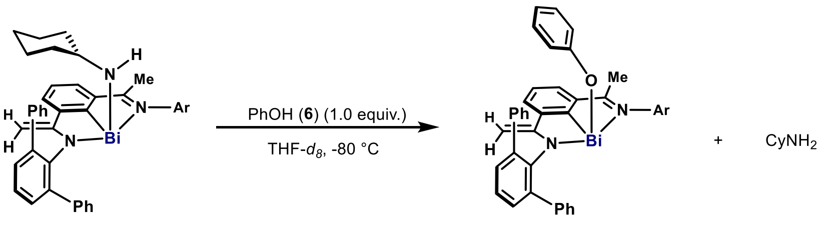

15 **7**, 82 % **14**

4.2 The reaction of **15** and **17** with H_2O

15 was generated in-situ from **1** (5 mg, 6.1µmol), **2** (3.2 mg, 12 µmol) and $CyNH_2$ (0.6 mg, 6.1 µmol) in 0.6 mL THF-d_8 in a J-Young NMR tube. The purple color of **1** disappeared quickly, and a light yellow solution formed, which indicates the full conversion to **15**. Then H_2O (0.2 mg, 12 µmol) was added at room temperature. **15** was consumed completely, while **9** and $CyNH_2$ were formed cleanly. The same reaction was obtained in the reaction of **17** and H_2O.

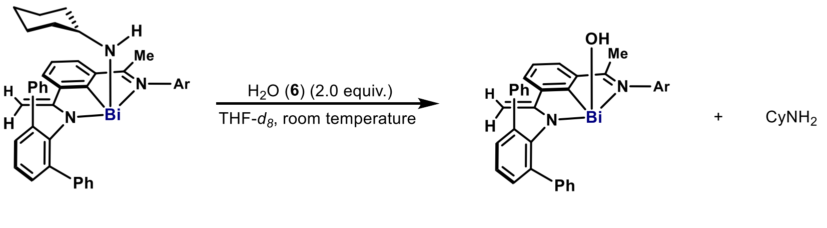

15 **9** **14**

4.3 <u>The reaction of **15** and **17** with HBpin</u>

15 (3.0 mg, 3.3 μmol) was dissolved in 0.6 mL THF-d_8 in a J-Young NMR tube. The tube was kept in a dry ice ethanol bath for 5 min. Then HBpin (0.5 μL, 3.3 μmol) was injected at this temperature. This mixture was monitored by proton NMR at -50 °C for 10 h.

As shown below, the decrease of **15** and increase of **1** was detected with time. A new species appeared towards the middle of the reaction and disappeared slowly in the end, which is proposed to be a bismuth hydride complex. The concentration of this intermediate plus the concentration of **1** equals to the concentration of **21**[9] during the reaction. The proton resonates at 26.03 ppm was proposed as a Bi-H due to the relativistic effect of bismuth. In addition, two protons at 3.96 and 3.09 ppm, as well as one methyl group at 1.93 ppm were detected together with Bi-H. So the intermediate was proposed as ***int-BiH***, which isomerized to **1** in 99% NMR yield (using the residual solvent peak as standard).

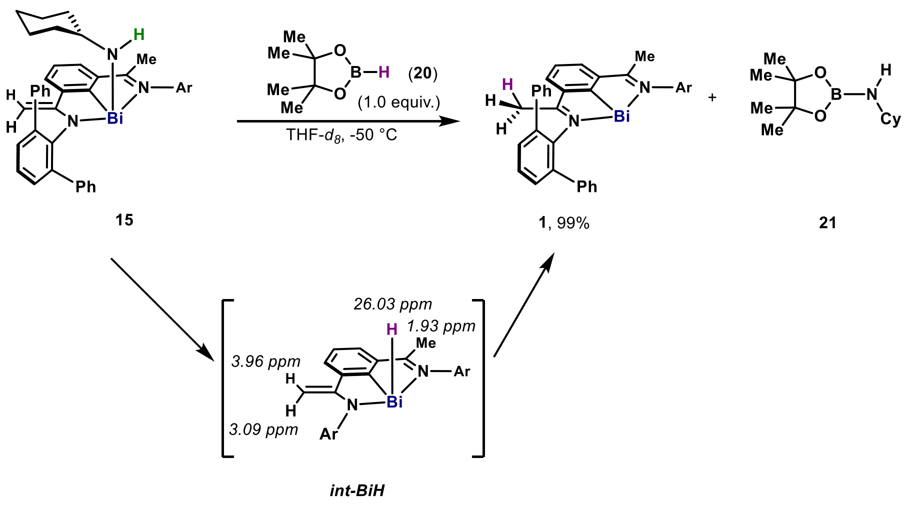

Scheme S2. The reaction of **15** and **20** in THF-d_8 at -50 °C, and the proposed structure of ***int-BiH***.

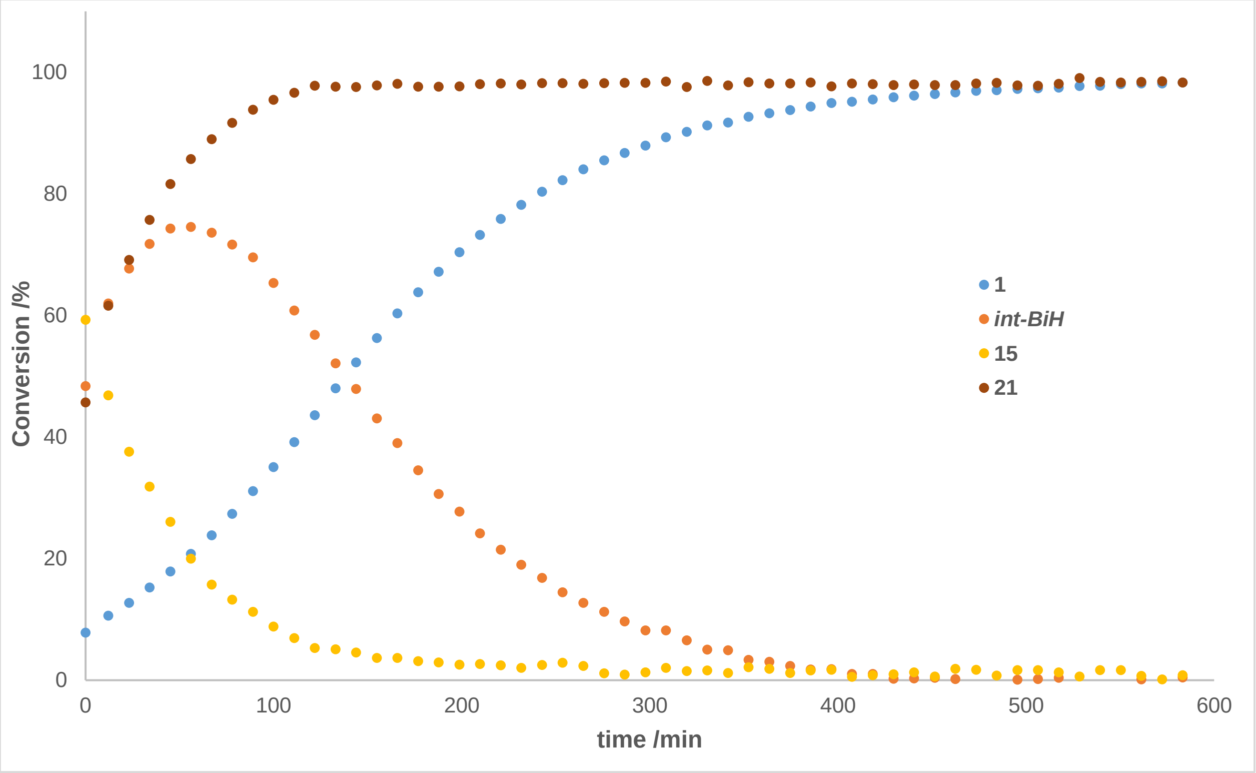

Fig. S8. Kinetic profile of the reaction of **15** and **20** in THF-d_8 at -50 °C.

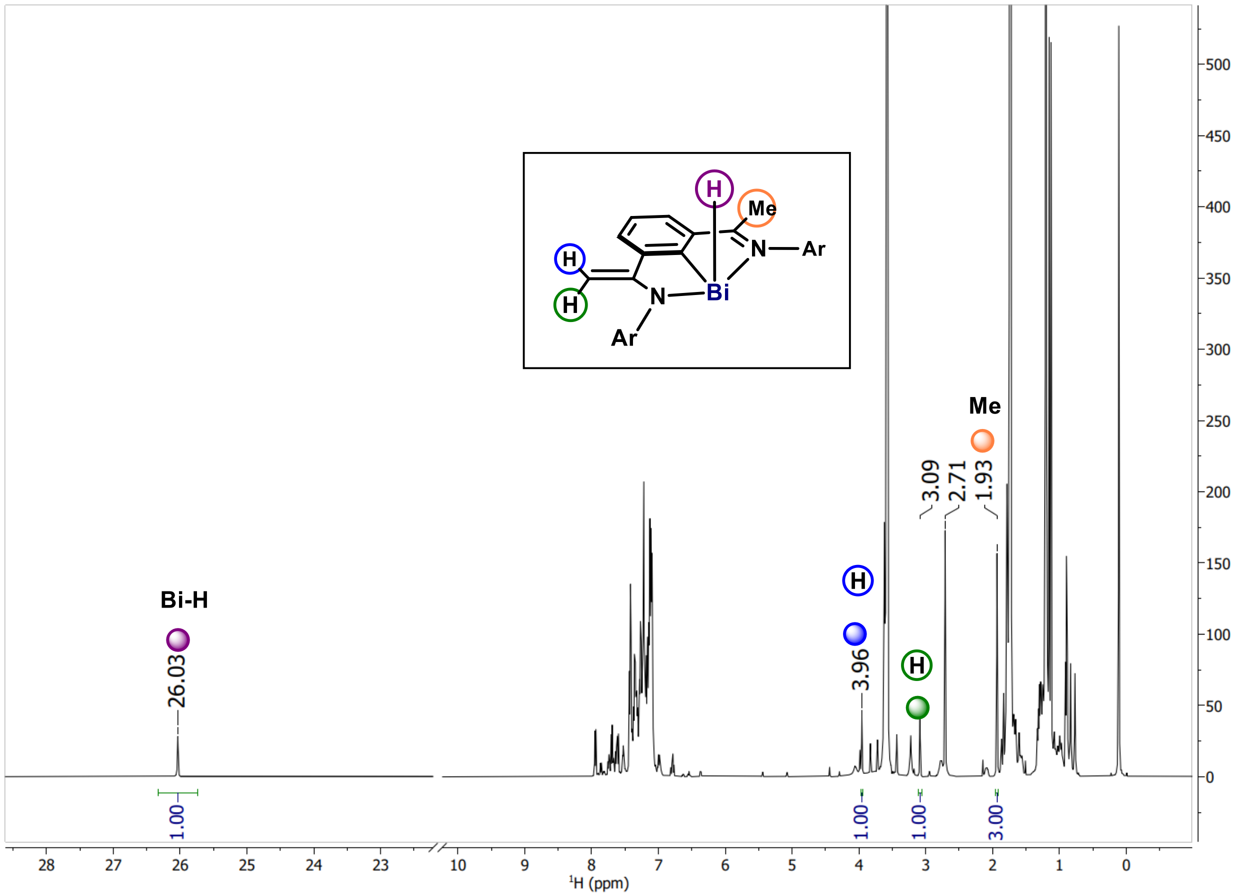

Fig. S9. Characteristic peaks of ***int-BiH*** in ^{1}H NMR in THF-d_8 at -50 °C.

If DBpin (92% deuterium labelling) was used in this reaction, the integration of Bi-H was around 8 % of other protons of ***int-Bi-H***, which confirms that proposed hydride is from the hydride of HBpin and it transfers to the methylene group on the backbone.

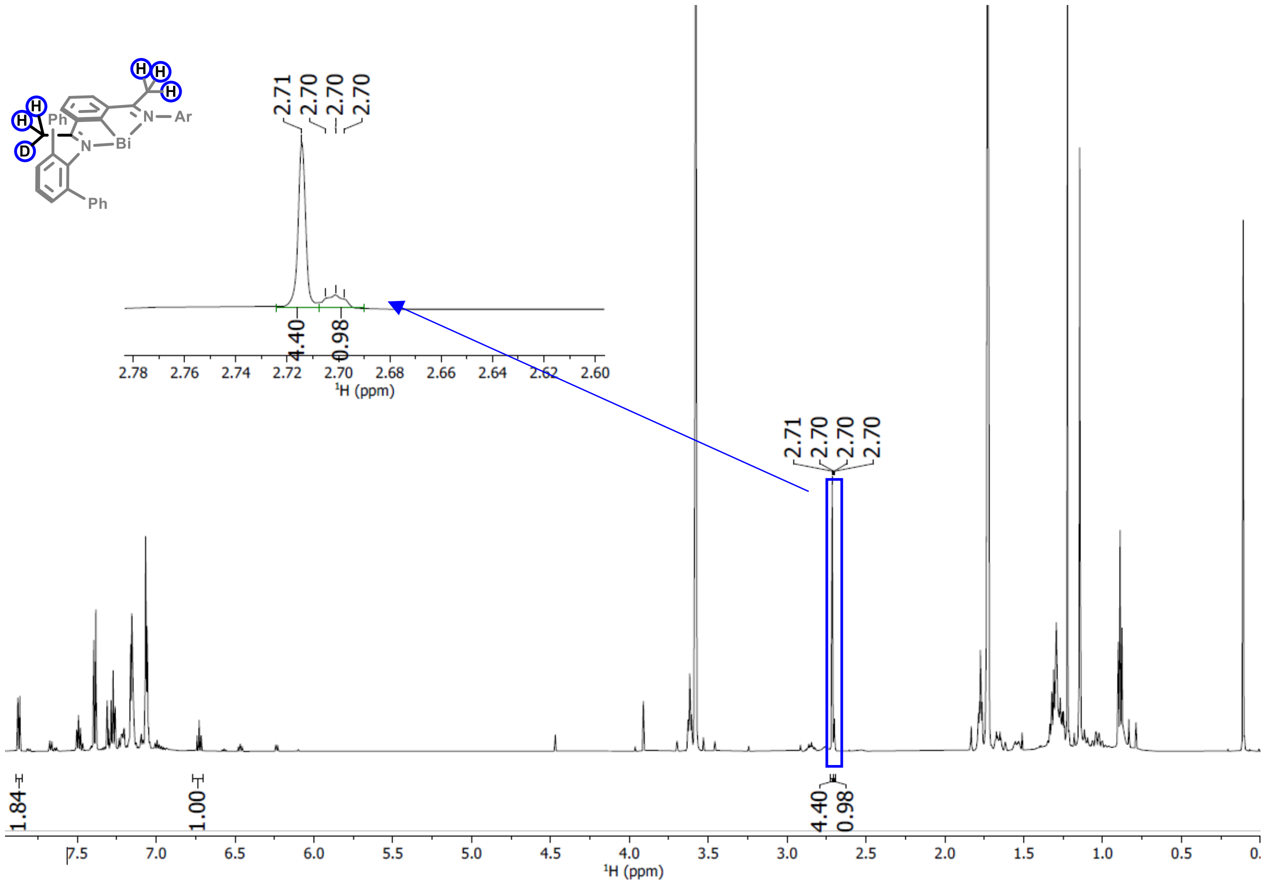

Fig. S10. Characteristic peaks of **1-D** in ^{1}H NMR in THF-d_8 at room temperature.

17 (3.0 mg, 3.6 μmol) was dissolved in 0.6 mL THF-d_8 in a J-Young NMR tube. The tube was kept in a dry ice ethanol bath for 5min. Then HBpin (0.5 μL, 3.6 μmol) was injected at this temperature. This mixture was monitored by proton NMR at -50 °C overnight. ***int-BiH*** was also detected in this reaction. Then **1** was generated quickly in 99% NMR yield (using the residual solvent peak as standard).

4.4 The reaction of **15** and **17** with BD_3

15 was generated in-situ from **1** (5 mg, 6.1μmol), **2** (3.2 mg, 12 μmol) and $CyNH_2$ (0.6 mg, 6.1 μmol) in 0.6 mL THF-d_8 in a J-Young NMR tube. The purple color of **1** disappeared quickly, and a light yellow solution formed, which indicates the full conversion to **15**. Then BD_3 solution (1M THF solution, 6.1 μL, 12 μmol) was added at room temperature. **15** was consumed completely, while **1** and $CyNHBD_2$ were formed cleanly. The same reaction was obtained in the reaction of **17** and BD_3.

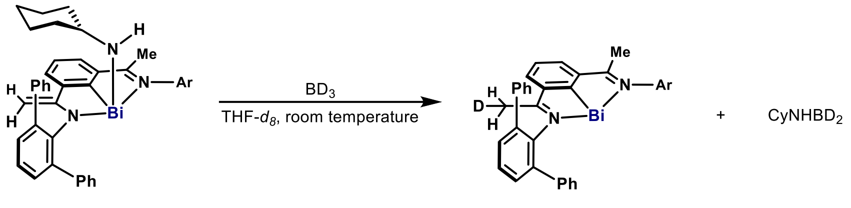

15 1

4.5 The reaction of **15** and **17** with Cp*CrH(CO)$_3$

15 (3 mg, 3.3 μmol) was dissolved in 0.6 mL THF-*d$_8$* in a J-Young NMR tube. Cp*CrH(CO)$_3$ (1.8 mg, 6.6 μmol) was placed at the top of the J-Young tube in a glovebox. The tube was frozen in liquid nitrogen and warmed until THF-*d$_8$* melted. Then the mixture was mixed at this temperature, and the J-Young tube was injected into a 500 MHz NMR spectrometer, which was precooled to -80 °C. In the first recorded proton NMR spectrum, **15** was consumed completely, while **1** and CyNH$_2$ were formed in 65% NMR yield. The same yield was obtained in the reaction of **17** and Cp*CrH(CO)$_3$.

Note: If the amount of Cp*CrH(CO)$_3$ was decreased to 1 eq., only 50% of **15** was consumed.

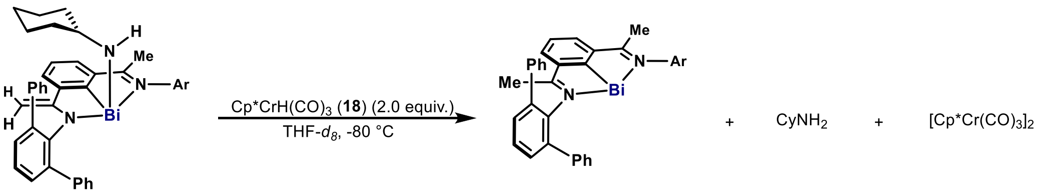

15 1, 65 % 14 19

Scheme S3. The reaction of **15** and **18** in THF-*d$_8$* at -80 °C.

4.6 The reaction of **15** and **17** with 2-naphthalenethiol

15 was generated in-situ from **1** (5 mg, 6.1μmol), **2** (3.2 mg, 12 μmol) and CyNH$_2$ (0.6 mg, 6.1 μmol) in 0.6 mL THF-*d$_8$* in a J-Young NMR tube. The purple color of **1** disappeared quickly, and a light yellow solution formed, which indicates the full conversion to **15**. Then 2 eq. of 2-naphthalenethiol was added at room temperature. **15** was consumed completely, while **1**, CyNH$_2$ and dithiol were formed cleanly. The same reaction was obtained in the reaction of **17** and H$_2$O.

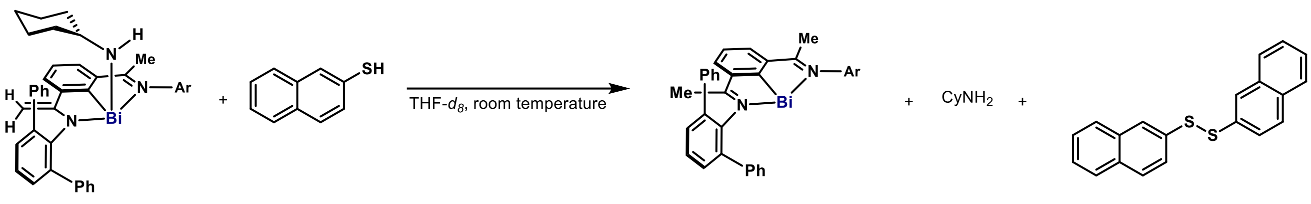

15 **2 eq.** **1** **14**

5 Isotope labeling experiments

5.1 The reaction of **4** with ND_3 in C_6D_6

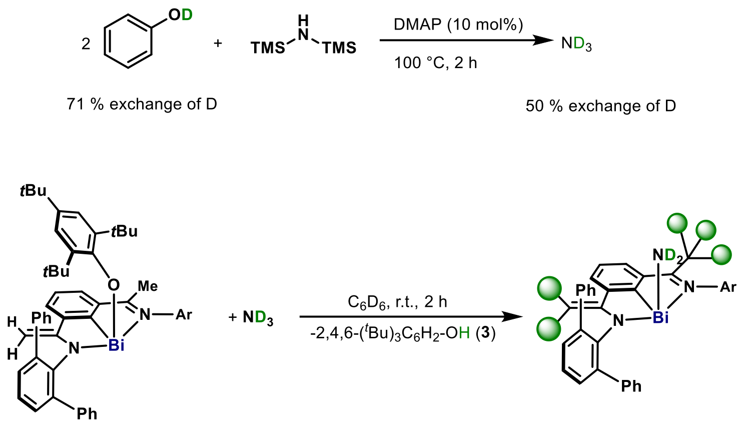

4 **17-D**

PhOD (5.0 mg, 0.053 mmol, 71% exchange of D), 1,1,1,3,3,3-hexamethyldisilazane (4.3 mg, 0.027 mmol), and 4-dimethylaminopyridine (0.3 mg, 0.0027 mmol) were added to one chamber of the two-chamber system. In the other chamber, **4** (5.0 mg, 0.0045 mmol) was dissolved in C_6D_6 (0.5 mL). The first chamber was stirred for 2 h at 100 °C, while the second chamber was stirred at 25 °C for 2 h in a water bath. ^{1}H NMR shows that 50% CH_2 protons are hydrogen atoms, and the rest 50% contain one deuterium.

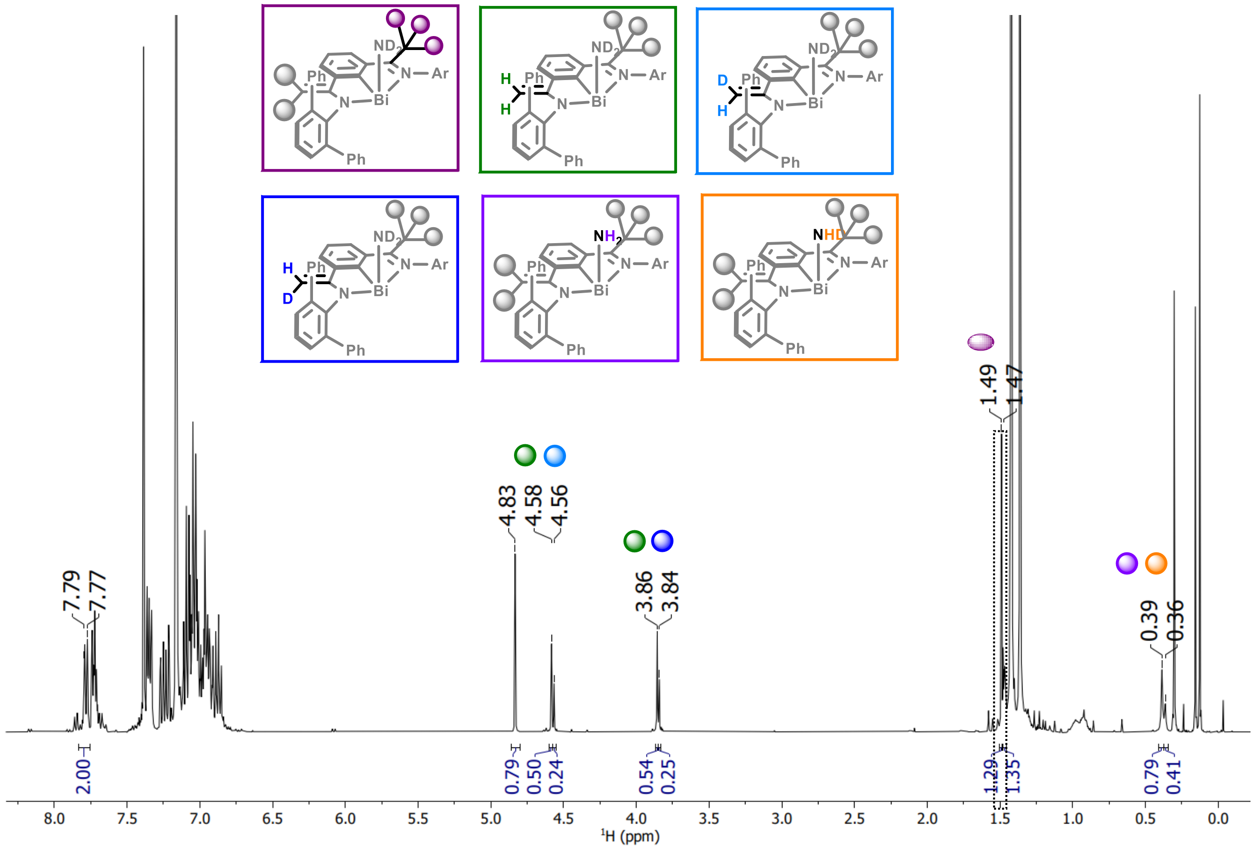

Fig. S11. ^{1}H NMR after the reaction of **4** and ND_3 was measured in toluene-d_8 at 25 °C.

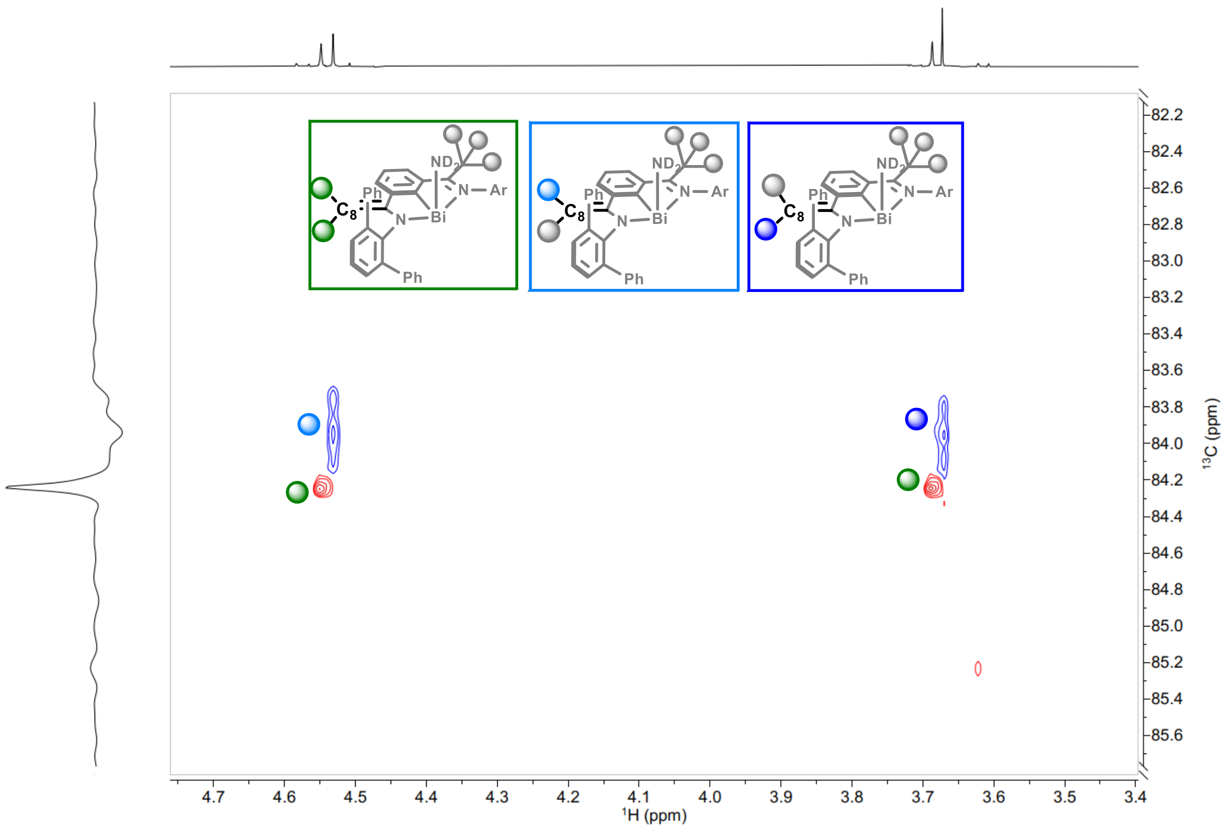

Fig. S12. High-Resolution edited- $^{1}H,^{13}C$-HSQC spectrum after the reaction of **4** and $CyND_2$ in toluene-d_8 at 25 °C. Red cross peaks show the presence of a CH_2 group, blue signals the presence of a CH or CH_3 group. The splitting of the blue peaks (1:1:1 triplett) is indicating a CHD group.

5.2 The reaction of **4** with ND_3 in C_6H_6

PhOD (20.0 mg, 0.210 mmol, 71% exchange of D), 1,1,1,3,3,3-hexamethyldisilazane (17.0 mg, 0.105 mmol), and 4-dimethylaminopyridine (1.3 mg, 0.0105 mmol) were added to one chamber of the two-chamber system. In the other chamber, **4** (10.0 mg, 0.0096 mmol) was dissolved in C_6H_6 (0.5 mL). The first chamber was stirred for 2 h at 100 °C, while the second chamber was stirred at 25 °C for 2 h in a water bath. ^{2}H NMR was scanned after this reaction.

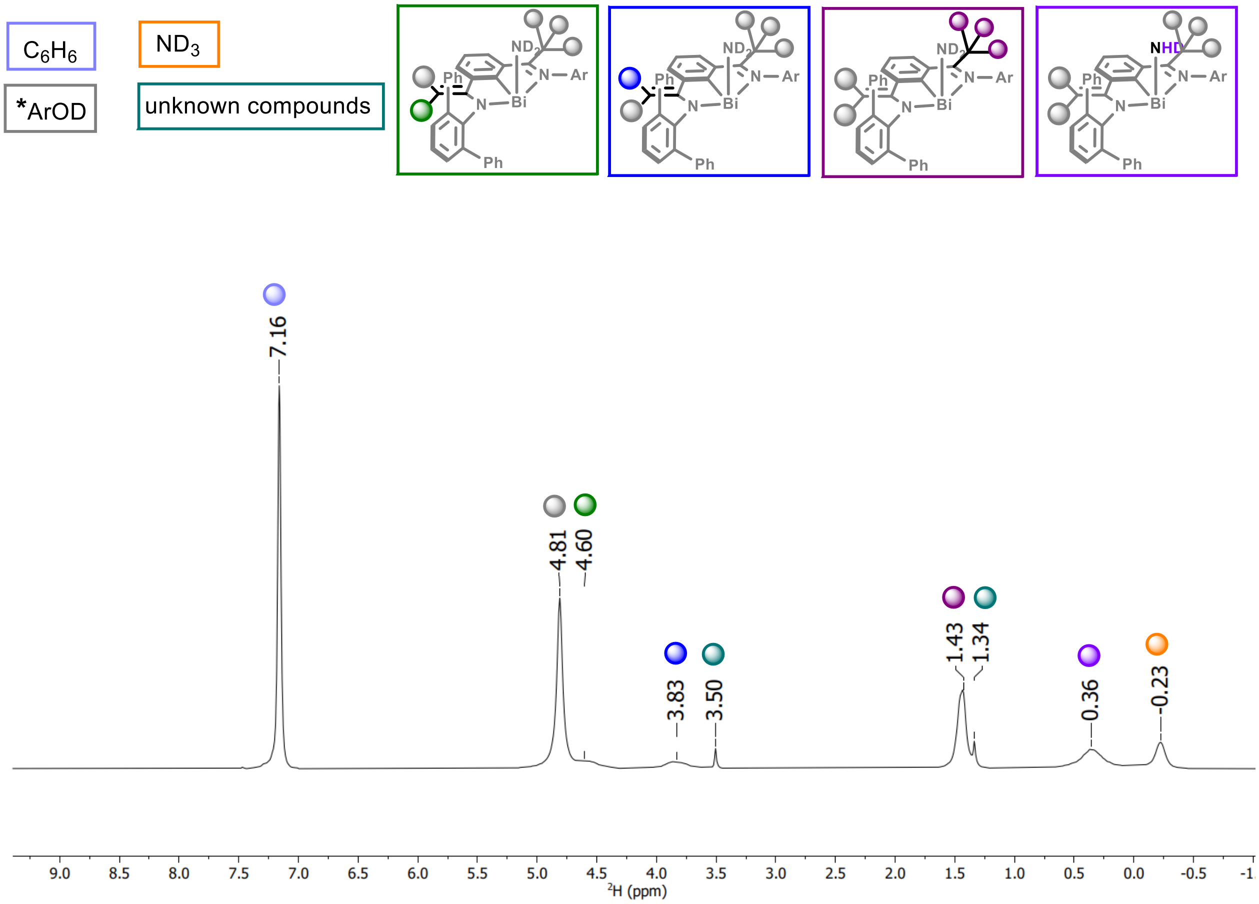

Fig. S13. ^{2}H-NMR spectrum after the reaction of **4** with ND_3 in C_6H_6 at 25 °C.

5.3 The reaction of **4** with ND_3 (< 1 eq.) in C_6H_6

PhOD (20.0 mg, 0.210 mmol, 71% exchange of D), 1,1,1,3,3,3-hexamethyldisilazane (17.0 mg, 0.105 mmol), and 4-dimethylaminopyridine (1.3 mg, 0.0105 mmol) were added to one chamber of the two-chamber system. In the other chamber, **4** (13.0 mg, 0.0125 mmol) was dissolved in C_6H_6 (0.5 mL). The first chamber was stirred for 2 h at 100 °C, while the second chamber was stirred at 25 °C for 2 h in a water bath. ^{2}H NMR was scanned after this reaction. 43% of **4** was converted to **17**, and around 1/6 of methylene proton were replaced by deuterium in both **4** and **17**.

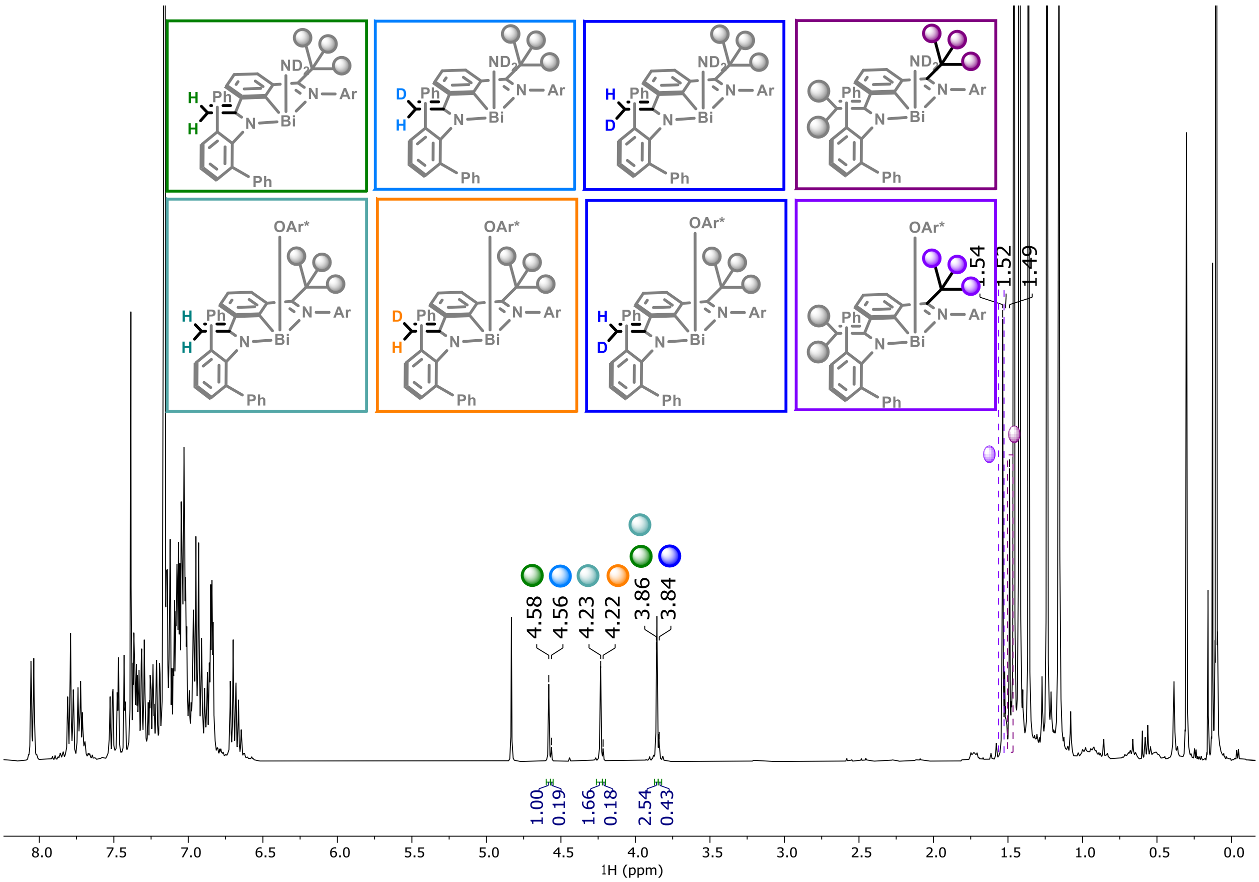

Fig. S14. ¹H NMR after the reaction of **4** and ND_3 (< 1 eq.) was measured in C_6D_6 at 25 °C. A mixture of **4** and **17** was detected.

5.4 The kinetic reaction of **4** with $CyND_2$ at -40 °C

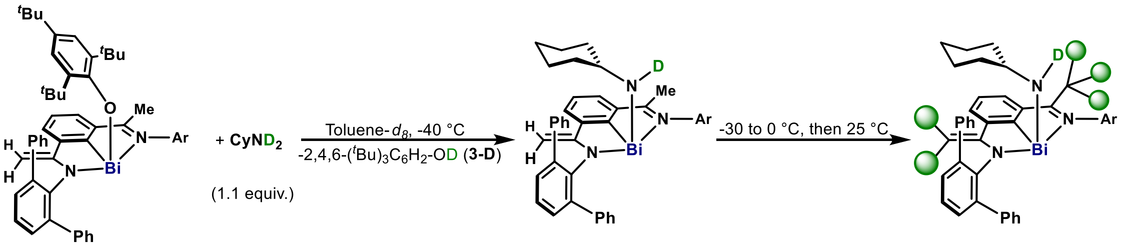

(1.0 equiv.)
4

15-D

15-D6

To a solution of **4** (5.0 mg, 0.0046 mmol) in toluene-d_8 (0.7 mL) was added $CyND_2$ (0.52 mg, 0.0052 mmol, 90% D exchange) at the top of the J-Young tube in a glovebox. The tube was cooled in dry ice, and the mixture was mixed at this temperature and injected into a 500 MHz NMR spectrometer, which was

precooled to -40 °C. This mixture was monitored by proton NMR at -40 °C for 7.6 h until **4** was consumed completely.

During this period, there was no deuterium incorporation detected for the methylene group, and the amount of **3** was around 10% of the generated **15-D**, which fits that $CyND_2$ 90% exchange of D.

The reaction was warmed up and stayed 20 min at -30 °C, -20 °C, -10 °C, and 0 °C, respectively. Then the reaction was monitored at 25 °C for 8.25 h until 46% of methylene protons were replaced by D.

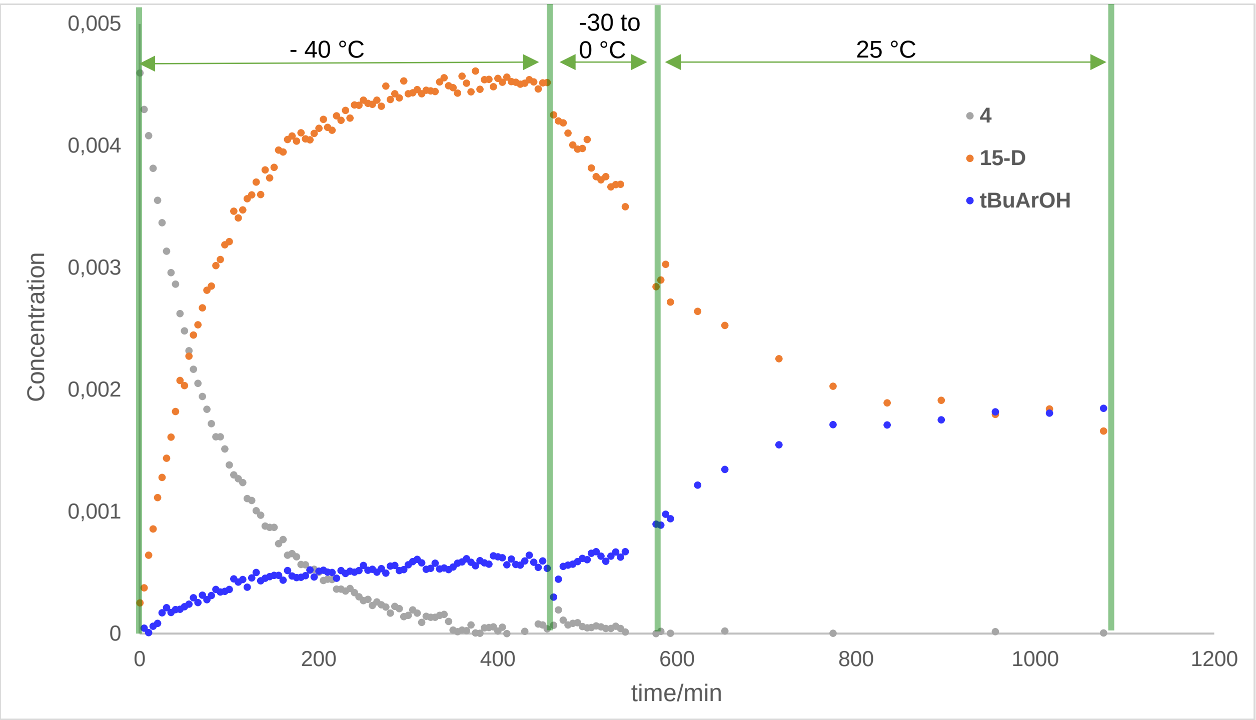

Fig. S15. Kinetic profile of the reaction of **4** and $CyND_2$ in toluene-d_8.

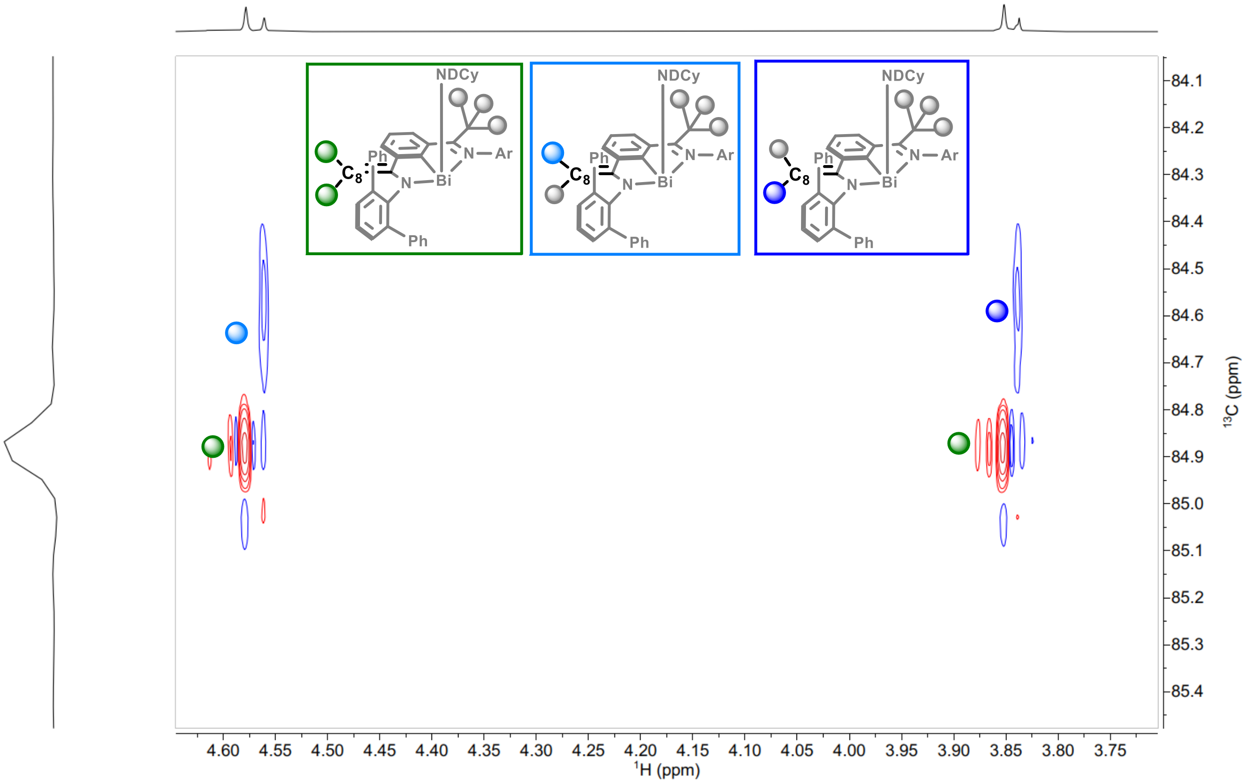

Fig. S16. High-resolution edited- $^{1}H,^{13}C$-HSQC spectrum after the reaction of **4** and $CyND_2$ in toluene-d_8 at 25 °C. Red cross peaks show the presence of a CH_2 group, blue signals the presence of a CH or CH_3 group.

6 EPR measurements

Methods: EPR spectra of **4** were recorded on a Bruker Elexsys 500 CW EPR spectrometer equipped with an ER4116DM resonator. To resolve the 0.2 MHz hyperfine splitting of the phenoxy radical, a field modulation amplitude of 0.005 mT was selected. A conversion time of 655ms was used and the EPR spectrum was recorded in one scan of 45 min over 4096 points using 0.5mW of microwave power. The microwave frequency was 9.62572 GHz.

The variable temperature EPR study was recorded on a Magnettech (Bruker) MS5000 desktop EPR spectrometer equipped with a N_2 flow cryostat and built-in temperature controller. Since, for these measurements, only the total amplitude of the phenoxy radical EPR spectrum was important, the field modulation amplitude was increased to 0.1 mT.

The solution EPR spectrum of **4** was simulated using the "garlic" routine in easyspin [10]:

```
% The spin system contains a total of 29 proton couplings in 3 groups
A_MHz = [4.76 1.045 0.20];     % hyperfine couplings in MHz
Sys.g = 2.00854;               % g-value
Sys.Nucs = '1H,1H,1H';         % 3 groups of equivalent nuclei
Sys.n = [2 9 18];              % # of nuclei in each group
Sys.A = A_MHz;                 % hyperfine interaction for each group.
Sys.lwpp = [0,0.006];          % line width (mT) Lorentzian shape
Exp.mwFreq = 9.532;            % microwave frequency
Exp.nPoints = 8192;            % number of points in spectrum
Exp.CenterSweep = [340,1];     % center field = 340 mT, Sweep is 1 mT.
% Simulation and plotting of the solution cw EPR spectrum
garlic(Sys,Exp);
```

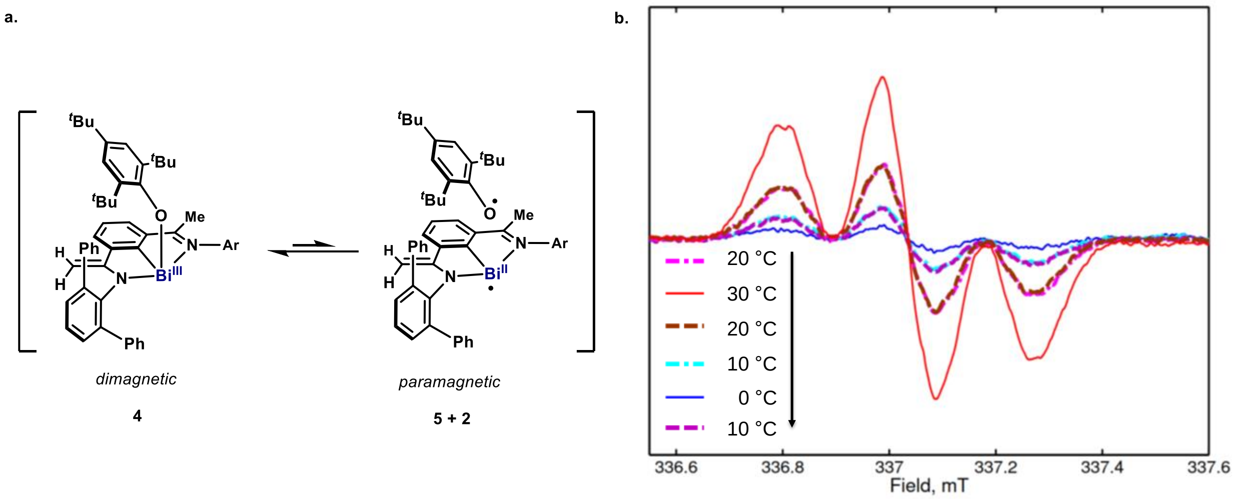

Fig. S17. a), Reversible homolysis of Bi-O bond of **4**. **b)**, VT EPR spectra of **4** in toluene.

Preparation of EPR sample for EPR spectra (Figure 2c, top), and VT EPR experiment (Fig. S17, b): **4** (5.0 mg, 0.006 mmol) was dissolved in toluene (0.5 mL) in a vial in a glovebox. 0.3 mL of this solution was transferred to a 0.5 mm quartz EPR tube, and measured at 25 °C or between 0 to 30 °C.

Note: No obvious signal decrease was detected at 20 °C after the temperature was increased to 30 °C in the VT EPR experiment.

Preparation of EPR sample for Van`t Hoff plot: **4** (8.52 mg, 7.83 μmol) was dissolved in toluene (0.6 mL) in a vial in a glovebox. The concentration of **4** was 13.08 mM. 0.3 mL solution was transferred to a 0.5 mm quartz EPR tube. The sample was measured between 20 °C and -30 °C within 2 h. The field modulation amplitude was increased to 0.1 mT.

Standard solution of TEMPO: TEMPO (4.4 mg, 28.2 μmol) was dissolved in 1.0 mL toluene, then 1 μL of the solution was diluted to 1.0 mL. The concentration of TEMPO was 0.0282 mM. The diluted solution was transferred to a 0.5 mm quartz EPR tube, which had an empty capillary.

The concentrations of free 2,4,6-tri-*tert*-butylphenoxyl radical, **2**, at different temperatures were calculated according to the integrations of EPR signals compared with standard TEMPO solution.

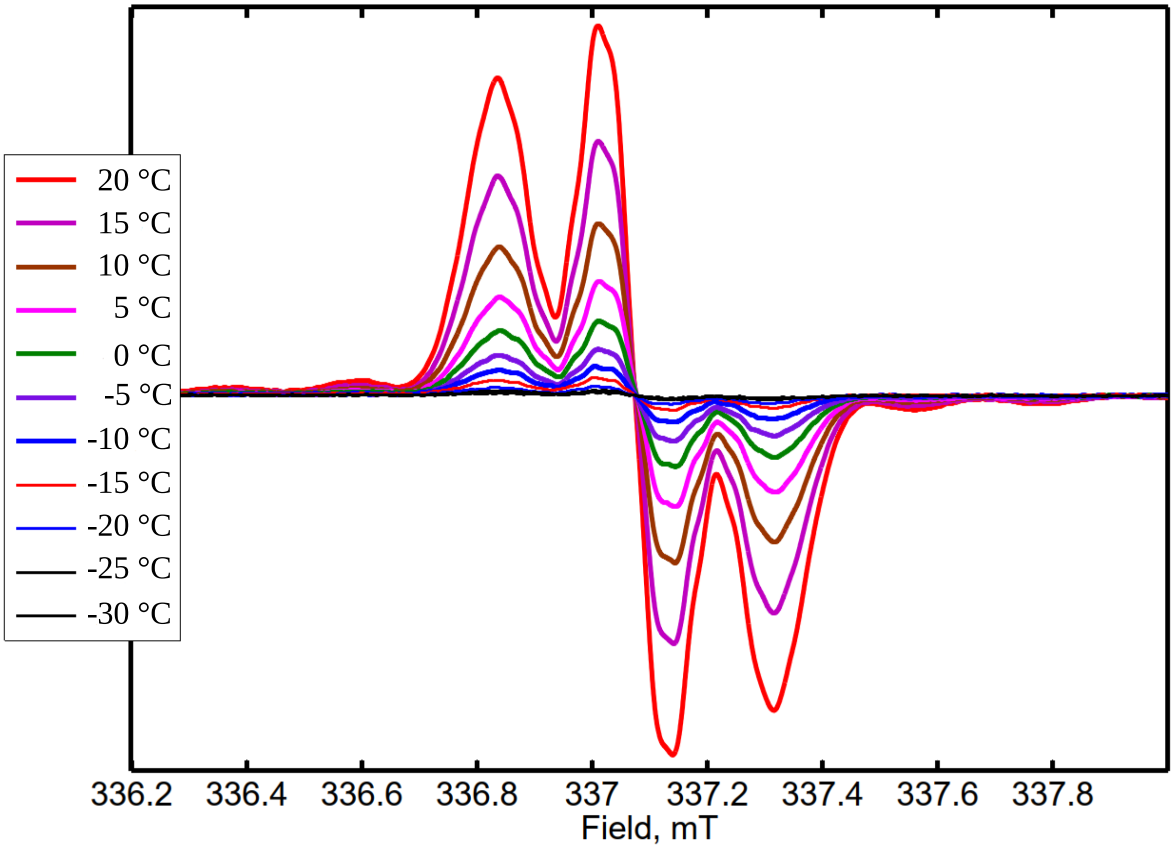

Fig. S18. VT EPR spectra of **4** in toluene used for the van`t Hoff plot.

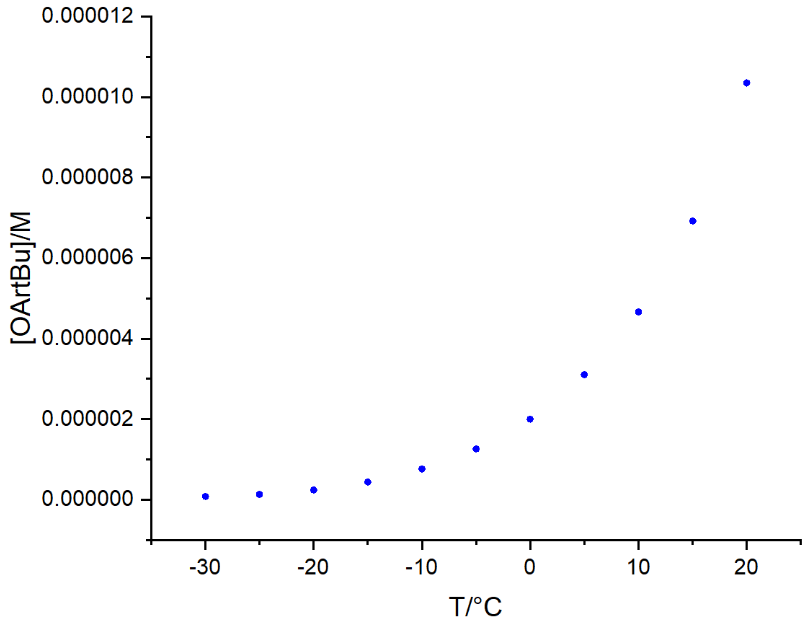

Fig. S19. Concentration of **2** calculated from VT EPR measurement.

ΔH and ΔS were determined by van`t Hoff equation.

$$\Delta G = \Delta H - T\Delta S = -RT lnK_{eq}$$

Rearranging:

$$lnK_{eq} = -\frac{\Delta H}{RT} + \frac{\Delta S}{R}$$

So by plotting lnK_{eq} vs 1/T, we get the slope - $\frac{\Delta H}{R}$ and abscissa $\frac{\Delta S}{R}$

$$K_{eq} = \frac{[\mathbf{5}] \times [\mathbf{2}]}{[\mathbf{4}]} = \frac{[\mathbf{2}]^2}{[\mathbf{4}]_0 - [\mathbf{2}]}$$

Table S1. Equilibrium constant $K_{eq.}$ between **4** and **5**+**2** in toluene at various temperatures.

T/°C	T (K)	1/T (K^{-1})	[**2**] (M)	$K_{eq.}$ (M)	$lnK_{eq.}$
20	293.15	0.003411223	1.03296E-05	8.18279E-09	-18.62123244
15	288.15	0.003470415	6.90713E-06	3.65776E-09	-19.42641607

10	283.15	0.003531697	4.65195E-06	1.65888E-09	-20.21712353
5	278.15	0.003595182	3.09785E-06	7.35551E-10	-21.03040128
0	273.15	0.003660992	1.99616E-06	3.05384E-10	-21.90944970
-5	268.15	0.003729256	1.25710E-06	1.21107E-10	-22.83434635
-10	263.15	0.003800114	7.56331E-07	4.38367E-11	-23.85054900
-15	258.15	0.003873717	4.35149E-07	1.44772E-11	-24.95844955
-20	253.15	0.003950227	2.38296E-07	4.34144E-12	-26.16281540
-25	248.15	0.004029821	1.27782E-07	1.24835E-12	-27.40920103
-30	243.15	0.004112688	7.94320E-08	4.82376E-13	-28.36005212

Matlab script used for van`t Hoff plot:

```
% MATLAB CODE to generate van't Hoff plot of VT EPR on 4
clear
% DATA: solvents (toluene) series temperature up/down
% Column contains [temperature, amplitude]
%---------------------------------------------------------------------
Told = [20, 2991; 15, 2000; 10, 1347; 5, 897; 0, 578; -5 364; -10, 219; -15,
126; -20, 69; -25, 37; -30, 23];
%----------------------------------------------------------------------------
% Concentrations of TEMPO (reference), Double Integration, Concentration of 4
in M
C_TEMPO = 0.0000282 ; DI_TEMPO = 324; C_4 = 0.01308;               % M
%----------------------------------------------------------------------------
% from the 2 spectra at 10°C also the double integration was taken
% since this has to be compared to that of TEMPO
DI = [62, 59, 67, 67, 57, 54]; Amp = [1518, 1464, 1744, 1682, 1477, 1347];
%----------------------------------------------------------------------------
% Conversion from DI to Amplitude and from DI to concentration
DI_Amp = mean(DI./Amp); C_DI = C_TEMPO/DI_TEMPO; %
% conversion of Amplitude to concentration: C = Amp*DI_Amp*C_DI ---------------
%%
% select VT trace (series with toluene and decreasing temperature)
% Take absolute temperature, convert amplitude to OAr concentration.
T = 273.15+Told(:,1); C_2 = Told(:,2)*DI_Amp*C_DI; nT = length(T);
% Calculate equilibrium constant for every temperature
Keq = C_2.^2 ./(C_4 - C_2);
plot(1./T,log(Keq),'o');
%----------------------------------------------------------------------------
%% linear regression : extract DeltaH and DeltaS
molgasR = 8.3145 % joule per mole per kelvin.
% solve overdetermined set of linear equations:
% -DeltaH/R * (1/T) + DeltaS/R = lnK or in matrix form:
% [1/T 1]*[-DeltaH/R DeltaS/R] = lnK in the form {matrix_A * vector_b =
vector_x}
% mldivide solves this set of linear equations: vector_b = matrix_A \ vector_x
ab = mldivide([1./T ones(nT,1)],log(Keq));
```

```
plot(1./T,log(Keq),'o',1./T,ab(2)+ab(1)./T,'-');

DeltaH = -ab(1)*molgasR * 0.000239006 ; % in kcal / mol
DeltaS = ab(2)*molgasR * 0.000239006 ; % in kcal / mol
```

Notice: To avoid the influence of THF, **4** used for VT EPR measurement and Van`t Hoff plot was synthesized in C_6H_6 with the same procedure in THF. With the concentration used for VT EPR experiment, the EPR signal was very weak below -30 °C; However, the signal of **2** was detectable at -150 °C if a higher concentration of **4** was used. If the temperature was increased to 40 °C, EPR signal at 20 °C decreased 20% compared with the initial measurement at 20 °C, which indicates significant decomposition of Bi complex at higher temperatures.

7 Computational Setup

All quantum chemical calculations in the present work were performed using the ORCA 5.0.1 program [11] suite employing the scalar relativistic zeroth order regular approximation (ZORA). Geometry optimizations were carried out using the BP86 density functional in conjunction with the ZORA-Def2-TZVP basis set for hydrogen (H), carbon (C), nitrogen (N), and oxygen (O) [12]. For Bismuth (Bi), the SARC-ZORA-TZVP basis set was used which features a contraction optimized for the ZORA Hamiltonian [12d, e]. The RI approximation with SARC/J fitting basis set was employed to accelerate the calculations [13]. Furthermore, atom-pairwise D3 dispersion correction with Becke-Johnson (D3BJ) damping was taken into account [14]. Subsequent frequency calculations revealed that all optimized geometries are local minima having no imaginary frequencies, while transition state geometries were verified by only one imaginary frequency. Single point energy calculations in solvent (Toluene) were carried out using PBE0 with the same basis set as in the geometry optimizations using the SMD solvation model [15]. Gibbs free energies (in kcal/mol) were calculated based on the (ZORA) PBE0-D3/Def2-TZVP (SMD:Toluene) single point solvation energies and free energy corrections at 298.15 K at the (ZORA) BP86-D3/Def2-TZVP level of theory. Note that computations of homolytic/heterolytic bond dissociation energies of organic and transition metal based complexes have shown good agreement with experimental data using the SMD continuum solvent model [16]. It has been found that computed bond dissociation free energies (BDFE) at (ZORA) PBE0-D3/Def2-TZVP level of theory show the ΔG value within 5 kcal/mol in comparison to the experimental results for the studied systems. The choice of functional is based on our experience and numerous benchmark studies that indicate that robust and accurate geometries can be obtained with this established combination of GGA and hybrid functionals (see a comparison of experimental and computed geometries below) [17].

7.1 Input files for DFT calculations

Input for optimization

```
!OPT FREQ BP86 D3BJ ZORA ZORA-def2-TZVP SARC/J

%basis

newgto Bi "SARC-ZORA-TZVP" end

end
```

Input for single-point solvation energy calculation

```
!PBE0 D3BJ ZORA ZORA-def2-TZVP SARC/J

%cpcm

smd True

smdsolvent "toluene"

end

%basis

newgto Bi "SARC-ZORA-TZVP" end

end
```

7.2 Bi-O bond dissociation free energy of complex **4**

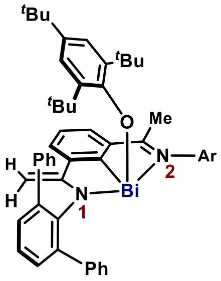

Fig. S20. Lewis structure of **4**.

Table S2. Comparison of optimized geometry of **4** with different methods with experimental crystal structure.

Geometrical parameter	PBE0	B3LYP	BP86	Exptl.
**Bi-O distance(Å)**	**2.12**	**2.14**	**2.17**	**2.17**
**Bi-C distance(Å)**	**2.21**	**2.23**	**2.22**	**2.20**

Bi-N1 distance(Å)	2.21	2.24	2.25	2.22
Bi-N2 distance (Å)	2.87	2.87	2.78	2.78
**<Bi-O-C1 angle (º)**	**154.3**	**152.0**	**148.2**	**136.2**
<C1-Bi-O angle (º)	97.7	99.6	91.3	95.0
<N1-Bi-O angle(º)	101.7	102.8	101.5	124
<N2-Bi-O angle(º)	89.1	88.6	84.2	85.0
Bi (Mulliken charges)	1.75e	2.12e	1.99e	---

We have performed a comparative study using the BP86, B3LYP, and PBE0 functional to assess their performance in the bond dissociation free energy (BDFE) calculations. The BP86-optimized geometry of **4** has shown good agreement with the experimental crystal structure. However, for the computations of the BDFE, we find results that are more consistent with experiment values using hybrid functionals [17c].

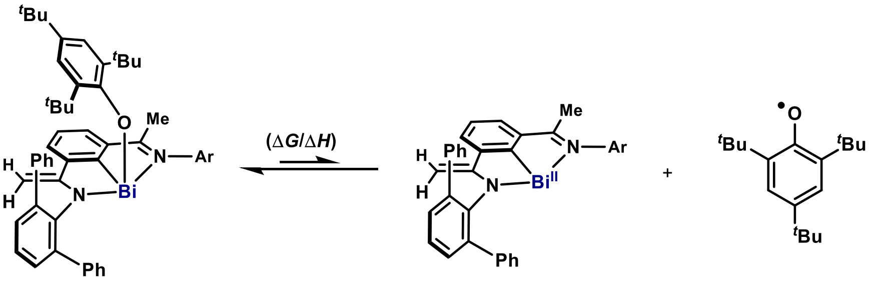

4 5 2

Table S3. Computed Bi-O bond homolytic energies of **4** at a different level of theory.

Data	PBE0-D3/Def2-TZVP(SMD:Toluene) //BP86-D3/Def2-TZVP	B3LYP-D3/Def2-TZVP(SMD:Toluene) //BP86-D3/Def2-TZVP	Exptl.
Δ*H* (kcal/mol)	25.0	28.3	28.0
Δ*G* (kcal/mol)	6.5	9.8	10.5
TΔ*S*(kcal/mol)	18.5	18.5	17.5

Table S4. Computed entropic contribution at the BP86-D3/Def2-TZVP level of theory (all energies are in kcal/mol).

TΔ*S*(elec.)	TΔ*S*(trans.)	TΔ*S*(rot.)	TΔ*S*(vib.)
0.82	12.35	9.59	-4.19
TΔ*S*(total) = 18.5 (computed)			
TΔ*S* = 17.5 (exptl.)			

7.3 Bi-O bond dissociation free energy of complex **7** and **17**

In addition to the Bi-O bond homolysis in compound **4**, we further computed the Bi-O and Bi-N bond homolysis taking 7 and 17 as a representative example. Interestingly, bond dissociation energies (Δ*G*) of **7** and **17** are 26.7 and 41.4 kcal/mol respectively. This larger Δ*G* values in comparison the compound **4** (Δ*G* = 6.5 kcal/mol) safely rule out the formation of radical spices either compound **7** or **17**.

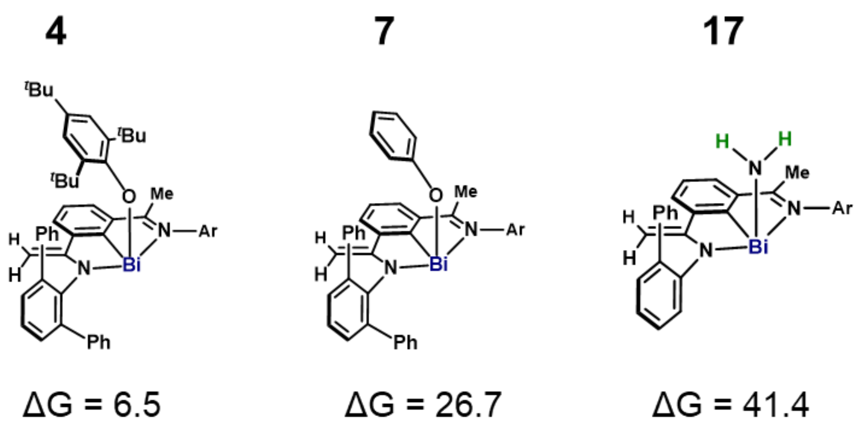

Fig. S21. Computed Bi-X (X = O,N) bond homolytic energies of **7** and **17** at (ZORA) PBE0-D3/Def2-TZVP (SMD:Toluene) level of theory.

7.4 Spin density plot

a

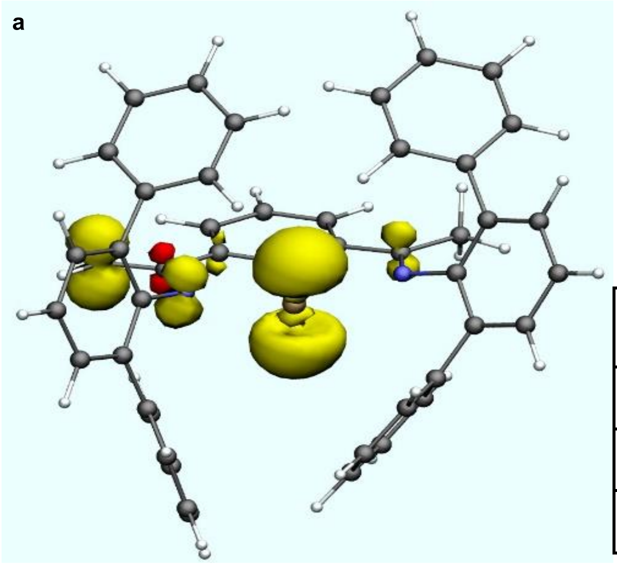

b

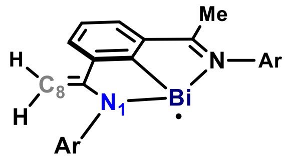

5

c

Spin population	Bi	N_1	C_8
Mulliken	76.9%	5.1%	27.7%
Löwdin	65.3%	5.3%	18.2%
Hirshfeld	61.0%	5.7%	16.4%

Fig. S22. **a**, Spin density of the Bi radical of **5** (isovalue = 0.004). **b**, Structure of **5**. **c**, Spin populations of Bi, N1, and C8 calculated by different methods.

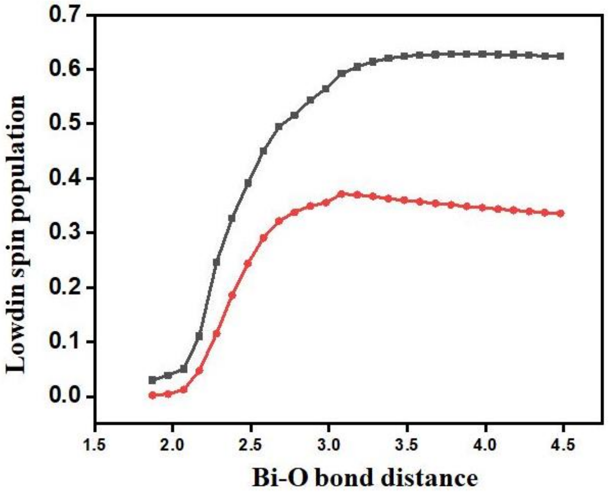

Fig. S23. Löwdin spin population distribution of Bi (black) and O (red) along the Bi-O bond dissociation. Note that the Löwdin spin population analysis is based on orthogonalized basis functions instead of the (possibly degenerate) original basis set and is more reliable in describing the difference between the α and β electron density.

7.5 Bond dissociation free energies (BDFE) values

The electronic energy for the hydrogen radical was found to be 13.616 eV (level of theory, see above), which is used for the BDFE calculations.

Table S5. Computed BDFE values of the selected species studied in this work.

Compound	X = N or O	
NH_3	$BDFE_{N\text{-}H}(NH_3)$ = 102.6 kcal/mol (calc.)<br>$BDFE_{N\text{-}H}(NH_3)$ = 100.3 kcal/mol (exptl.)	
H_2O	$BDFE_{O\text{-}H}(H_2O)$ = 113.1 kcal/mol (calc.)<br>$BDFE_{O\text{-}H}(H_2O)$ = 110.6 kcal/mol (exptl.)	
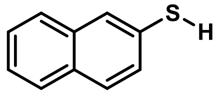	$BDFE_{S\text{-}H}$(2-naphthalenethiol) = 75.9 kcal/mol (calc.)	
Bi complex	X = N or O	
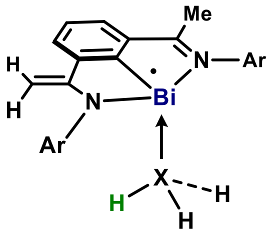	$BDFE_{N\text{-}H}(NH_3)$	47.0 kcal/mol
	$BDFE_{N\text{-}H}(CyNH_2)$	59.1 kcal/mol
	$BDFE_{O\text{-}H}(CyOH)$	52.3 kcal/mol
	$BDFE_{O\text{-}H}(H_2O)$	52.1 kcal/mol
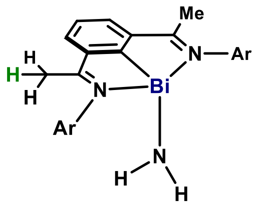	$BDFE_{C\text{-}H}$(ligand)	51.0 kcal/mol
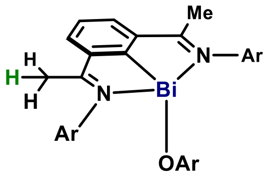	$BDFE_{C\text{-}H}$(ligand)	60.4 kcal/mol

<table>
<tr><td rowspan="2">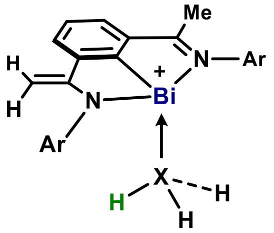
</td><td>$BDFE_{N-H}(NH_3)$</td><td>77.8 kcal/mol</td></tr>
<tr><td>$BDFE_{O-H}(H_2O)$</td><td>68.1 kcal/mol</td></tr>
</table>

7.6 Computational studies of N-H bond activation

In the deuterium labeling experiment, two distinct H/D exchange processes were observed: (i) H/D exchange into the vinylic positions of the Bi-amido complex, and (ii) H/D exchange between the Bi-amido complex and deuterated phenol. Experimentally, the H/D exchange of the NH_2 sites of the Bi-amido complex (**III**) occurs more rapidly than for the vinylic sites. This is indicated by the experimental studies as the generation of the Bi radical occurs from complex **4** prior to the H/D exchange. However, this H/D exchange behavior raises an important question: what is the likely mechanism for the H/D exchange process in the vinylic positions?

In the following, we will again outline the mechanistic hypothesis as obtained from DFT calculations and also present additional results that give a more detailed picture of plausible pathways of the mechanism and the H/D exchange experiments discussed above.

7.6.1 Results of the mechanistic pathway for H/D exchange: coordination followed by direct hydrogen transfer to OAr radical pathway

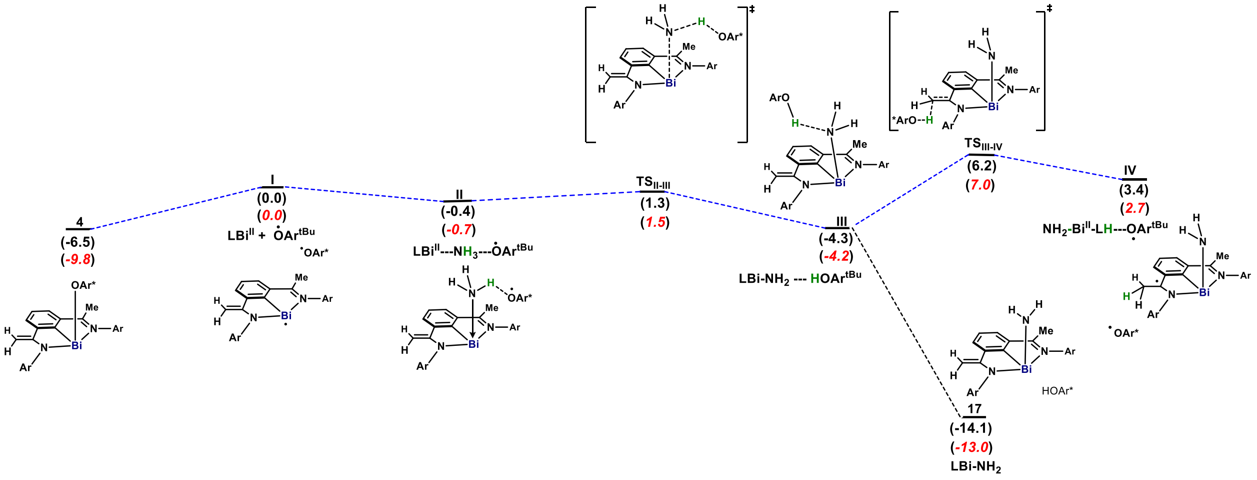

Fig. S24. Relative free energies (ΔG, in kcal/mol) computed based on (ZORA) PBE0-D3/Def2-TZVP (SMD:Toluene) single point energies, and free energy corrections at 298.15 K obtained at the (ZORA) BP86-D3/Def2-TZVP level of theory. Relative free energies (ΔG, in kcal/mol) computed at the (ZORA)B3LYP-D3BJ/Def2-TZVP level of theory are shown in red color.

Consistent with the experimental observations, our electronic structure calculations indicate that ammonia first coordinates to the Bi radical to form intermediate **II** as seen in **Fig. S24**. The transition state, which corresponds to the hydrogen transfer from ammonia to the OAr radical, has by far the lowest activation barrier (**TS_{II-III}**, 1.7 kcal/mol). The NBO charges of the H (0.45 of an electron charge) at TS_{II-III} indicate that homolytic cleavage of the N-H bond at the Bi center is concurrent with the formation of the O-H bond. Note that no broken-symmetry solution of TS_{II-III} could be found, indicating that the close-shell single state is by far the most stable configuration. The computed energy profile suggests that H/D exchange on the vinylic sites of the ligand occurs after the generation of hydrogen-bonded Bi-amido complex (**III**) as seen in **Fig. S24**. It should be noted that III-1 was isolated experimentally. From the experiments with the deuterated substrates, it is known that the rate of H/D exchange is much higher in NH_2 sites than the rate of H/D exchange at the vinylic sites of **III**. The calculations show that the transition state (**TS_{III-IV}**, 10.5 kcal/mol) corresponding to the hydrogen transfer from **3** to the vinylic position of the ligand is higher than the activation energy (**TS_{III-II}**, 5.6 kcal/mol) for the reverse reaction i.e. hydrogen exchange between intermediate **III** and **II**. DFT calculations with the B3LYP functional yield a free energy barrier for TS_{II-III} and TS_{III-IV} of 1.5 and 7.0 kcal/mol, respectively. Note that two different DFT functionals yield energies for TS_{II-III} and TS_{III-IV} which agree within ~5 kcal/mol. This provides a clear indication that H/D exchange is

more feasible at NH_2 sites of **III** at low temperature while H/D exchange at vinylic sites of **III** would be accessible at higher temperatures.

Participation of the ligand backbone in pincer ligand based metal complexes have been found to be a key step to a various number of catalytic reaction [18]. Hence, we also investigated other pathways to gain additional support for the description of H/D exchange process.

7.6.2 Mechanism of ammonia coordination and metal-pincer ligand cooperation pathway

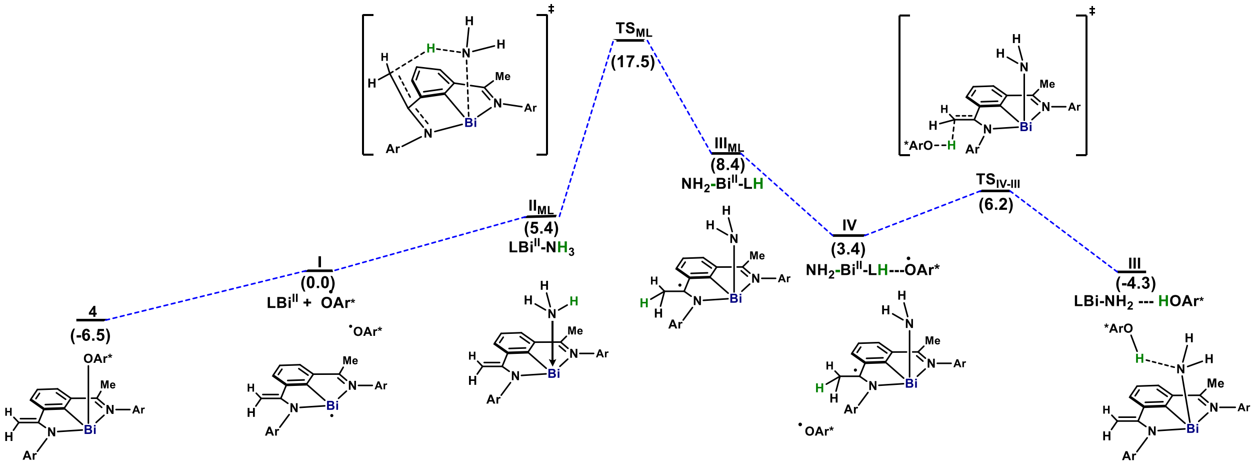

Fig. S25. Relative free energies (ΔG, in kcal/mol) computed based on (ZORA) PBE0-D3/Def2-TZVP (SMD:Toluene) single point energies, and free energy corrections at 298.15 K obtained at the (ZORA) BP86-D3/Def2-TZVP level of theory.

As seen in **Fig. S25**, the mechanism for the formation of Bi-amido complex (**III**) from the starting structure 4 involves three steps:(i) homolytic bond cleavage to generate the Bi radical followed by ammonia coordination (**II$_{ML}$**), (ii) hydrogen migration to the vinylic site of the pincer ligand, and (iii) hydrogen abstraction by OAr radical to form Bi-amido complex (**III**). After coordination, hydrogen migrates to the ligand over a free energy barrier of 17.5 kcal/mol via transition state **TS$_{ML}$** to form intermediate **III$_{ML}$**. Subsequently, the hydrogen radical of the vinylic site transfers to the OAr radical through transition state **TS$_{IV\text{-}III}$** of 6.2 kcal/mol producing the final Bi-amido complex. Computational results suggest that the H/D exchange occurs between intermediate **IV** and **III** with *ArOH (**3**)/*ArOD (**3-D**). However, the species **III$_{ML}$** formed from the hydrogen migration through **TS$_{ML}$** state is considered a high-energy barrier of this pathway and, hence, less favorable according to the experimental reaction conditions.

7.6.3 Mechanism of ammonia coordination and heterolytic cleavage of N-H bond pathway

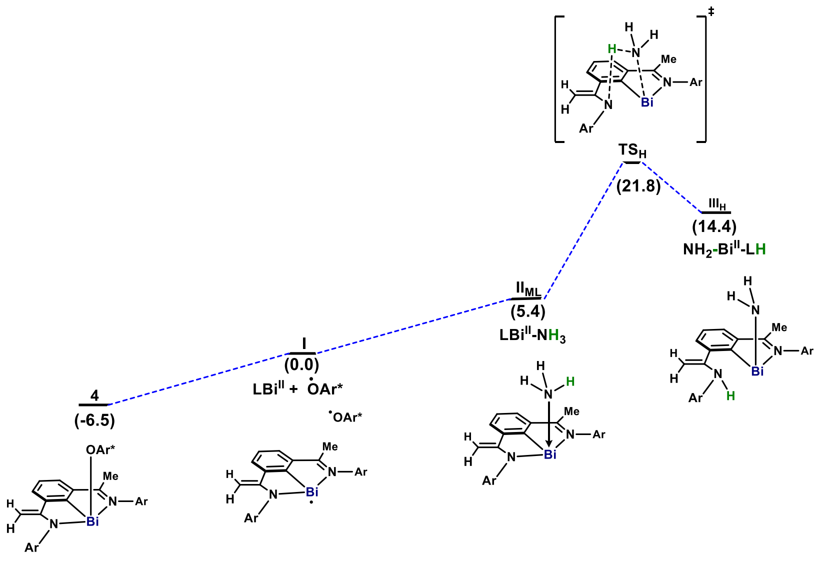

Fig. S26. Relative free energies (ΔG, in kcal/mol) are computed based on (ZORA) PBE0-D3/Def2-TZVP (SMD:Toluene) single point energies, and free energy correction at 298.15 K obtained at the (ZORA) BP86-D3/Def2-TZVP level of theory.

As an alternative pathway, we also investigated the heterolytic N-H bond cleavage from the intermediate **II$_{ML}$** where hydrogen migrates to the adjacent N sites of the pincer ligand as seen in **Fig. S26**. The computed free energy barrier of the transition state **TS$_H$** of the proposed pathway is 21.8 kcal/mol (see **Fig. S26**). As a consequence, the computational results suggest that this pathway is also unfavorable to proceed under the experimental reaction conditions due to high-energy transition state **TS$_H$** and intermediate **III$_H$**.

7.6.4 Mechanism of N-H bond cleavage by complex **4** pathway

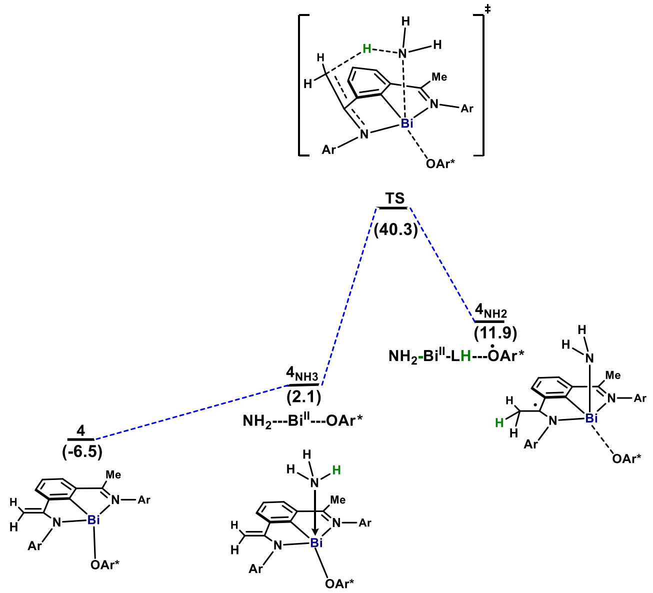

Fig. S27. Relative free energies (ΔG, in kcal/mol) are computed based on (ZORA) PBE0-D3/Def2-TZVP (SMD:Toluene) single point energies, and free energy correction at 298.15 K obtained at the (ZORA) BP86-D3/Def2-TZVP level of theory.

In addition, we also investigated the N-H bond activation of ammonia with starting complex **4** based on metal-pincer ligand cooperation, as seen in **Fig. S27**. Here, ammonia first coordinates to the Bi center in trans orientation with the OAr ligand to form intermediate $\mathbf{4_{NH3}}$ of 2.1 kcal/mol. In this pathway, the transition state is located corresponding to the hydrogen migration to the vinylic site of the pincer ligand. It is, however, unfavorable because the activation energy of the hydrogen transfer is 40.3 kcal/mol. Therefore, this route is not considered further.

Overall, the calculations show that the reaction based on the coordination of ammonia at Bi radical followed by hydrogen transfer to OAr radical is the most feasible pathway (see **Fig. S24**). H/D exchange at the NH_2 sites at lower temperatures and vinylic sites at higher temperatures implies that the catalytically active species is a Bi-amido complex. The reaction profile shows that the Bi-amido complex is located between these two limiting states; hence, the reaction condition will strongly influence this step.

8 Crystallographic data

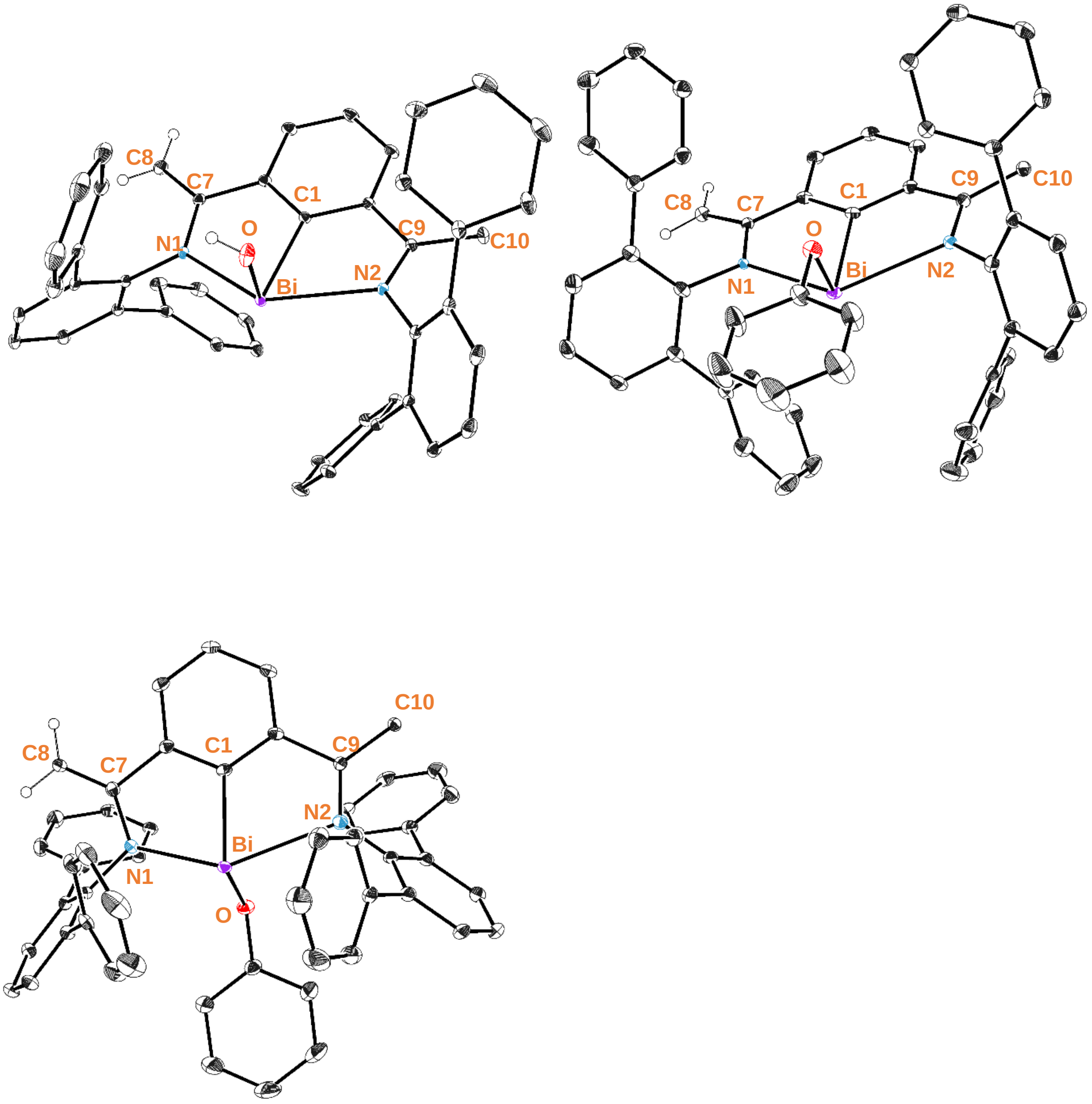

Fig. S28. Solid state structure of **7** and **9**, illustrated using 30% probability ellipsoids. Solvents, hydrogen atoms, except those on C8 and O, have been omitted for clarity.

Table S6. Summary of important bond distances and angels in **4**, **7**, **9**, **15**, **17**.

	**4**	**7-1**	**7-2**	**9**	**15**	**17**
Bi-C1 / Å	2.199(4)	2.191(3)	2.196(3)	2.196(2)	2.191(4)	2.1932(16)
Bi-N1 / Å	2.214(3)	2.210(9)	2.211(9)	2.248(2)	2.254(3)	2.2616(14)
Bi-N2 / Å	2.793(3)	2.729(9)	2.717(9)	2.626(2)	2.665(3)	2.6326(14)

Bi-X / Å	2.178(3)	2.140(2)	2.132(2)	2.143(2)	2.161(3)	2.1775(16)
C7-C8 / Å	1.355(6)	1.571(15)	1.464(15)	1.354(4)	1.352(5)	1.357(2)
C7-N1 / Å	1.379(5)	1.339(17)	1.340(17)	1.384(3)	1.368(5)	1.381(2)
C1-Bi-N1 / °	75.48(13)	74.2(3)	91.6(3)	75.28(8)	74.96(12)	75.20(6)
C1-Bi-X / °	95.07(12)	102.70(9)	102.84(9)	94.32(9)	99.36(14)	94.42(6)
N1-Bi-X / °	85.65(12)	81.3(2)	74.2(3)	98.81(8)	96.79(12)	99.26(6)
Sum / °	256.2	258.2	268.64	268.4	271.11	268.88

8.1 Single crystal structure analysis of **4**

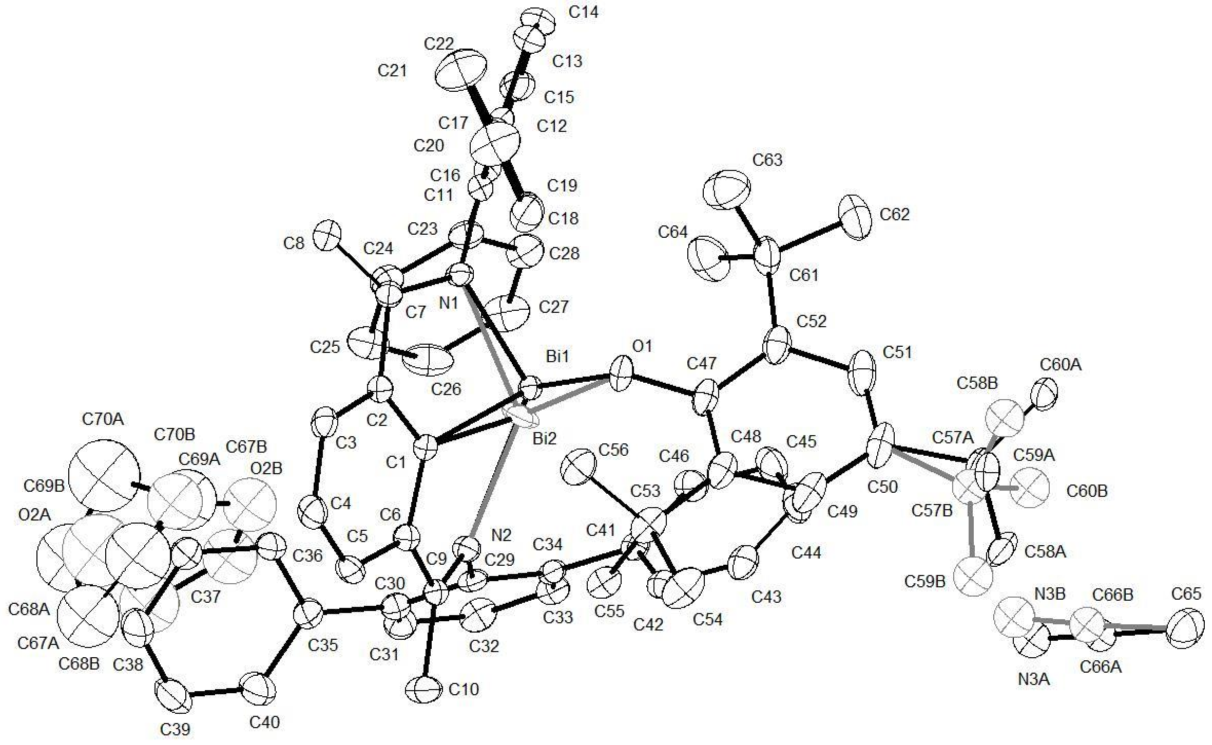

Fig. S29. The molecular structure of complex **4**. H atoms have been removed for clarity.

X-ray Crystal Structure Analysis of complex **4**:

$C_{138}H_{145}Bi_2N_5O_4$, M_r = 2355.54 g mol^{-1}, orange prism, crystal size 0.063 x 0.033 x 0.033 mm^3, monoclinic, space group $P2_1$/c [14], a = 11.0628(6) Å, b = 34.091(2) Å, c = 14.6775(9) Å,

β = 93.122(3)°, V = 5527.2(6) Å^3, T = 100(2) K, Z = 2, D_{calc} = 1.415 g·cm^3, λ = 0.71073 Å, μ(*Mo-K*$_\alpha$) = 3.239 mm^{-1}, analytical absorption correction ($T_{\min}$ = 0.83913, $T_{\max}$ = 0.92888), Bruker-AXS Kappa Mach3 with APEX-II detector and IμS microfocus X-ray source, 1.195 < θ < 29.131°, 166703 measured reflections, 14879 independent reflections, 12538 reflections with $I > 2\sigma(I)$, R_{int} = 0.0657. The structure was solved by *SHELXT* and refined by full-matrix least-squares (*SHELXL*) against F^2 to R_1 = 0.0495 [$I > 2\sigma(I)$], wR_2 = 0.0824, 726 parameters and 29 restraints.

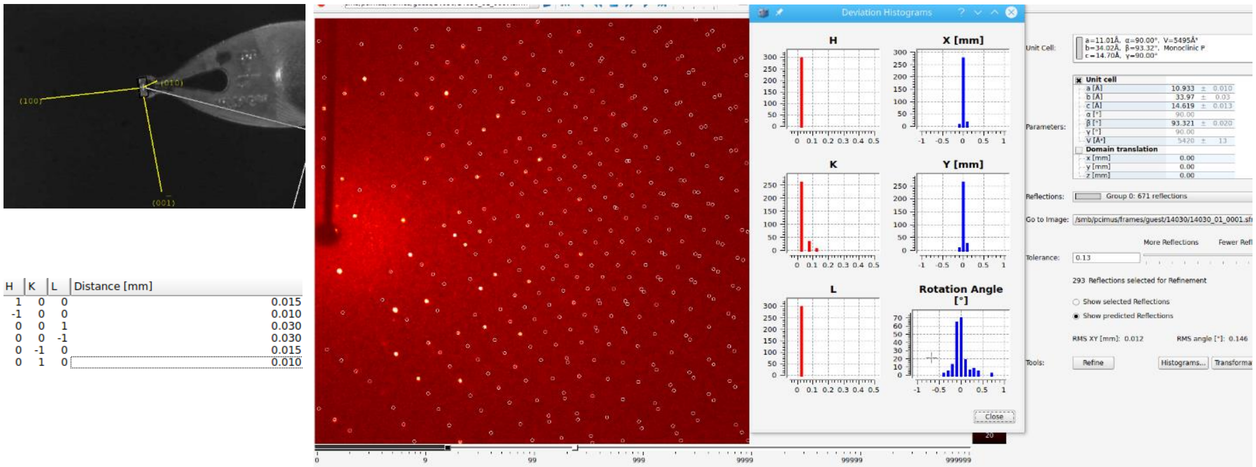

Fig. S30. Crystal faces and unit cell determination/refinement of **4**.

INTENSITY STATISTICS FOR DATASET

Resolution	#Data	#Theory	%Complete	Redundancy	Mean I	Mean I/s	Rmerge	Rsigma
Inf - 2.89	257	258	99.6	17.56	125.80	86.41	0.0194	0.0083
2.89 - 1.92	605	605	100.0	18.26	74.81	72.58	0.0265	0.0097
1.92 - 1.52	860	860	100.0	18.32	56.85	61.16	0.0347	0.0116
1.52 - 1.32	885	885	100.0	18.28	40.71	50.75	0.0470	0.0146
1.32 - 1.20	846	846	100.0	17.84	34.03	42.77	0.0569	0.0174
1.20 - 1.11	898	898	100.0	17.13	29.20	36.27	0.0690	0.0208
1.11 - 1.04	929	929	100.0	13.32	25.92	27.67	0.0784	0.0280
1.04 - 0.99	834	834	100.0	11.26	22.30	22.38	0.0915	0.0360
0.99 - 0.95	799	799	100.0	9.92	20.75	19.10	0.1020	0.0420
0.95 - 0.91	942	942	100.0	8.84	17.77	15.74	0.1184	0.0523
0.91 - 0.88	833	833	100.0	8.07	16.67	13.70	0.1260	0.0595

0.88 - 0.85	911	911	100.0	7.77	14.10	11.61	0.1447	0.0727
0.85 - 0.83	742	742	100.0	7.49	13.28	10.72	0.1574	0.0796
0.83 - 0.80	1195	1195	100.0	7.29	11.81	9.24	0.1775	0.0940
0.80 - 0.78	878	878	100.0	7.00	11.09	8.26	0.1964	0.1056
0.78 - 0.77	504	504	100.0	6.75	9.90	7.37	0.2064	0.1215
0.77 - 0.75	1055	1055	100.0	6.74	9.56	6.89	0.2236	0.1294
0.75 - 0.73	1169	1169	100.0	6.54	8.93	6.21	0.2495	0.1460
0.73 - 0.72	644	644	100.0	6.22	7.73	5.23	0.2780	0.1748
0.72 - 0.71	688	688	100.0	6.27	7.53	4.96	0.2934	0.1833
0.71 - 0.70	658	683	96.3	5.17	7.69	4.58	0.2951	0.2078
0.80 - 0.70	5596	5621	99.6	6.43	9.02	6.31	0.2397	0.1453
Inf - 0.70	17132	17158	99.8	10.50	23.04	22.36	0.0673	0.0430

The unusually large, negative residual electron density of -4.79 e·$Å^{-3}$ in 1.71 Å distance from C19 could possibly be caused by impurities of another crystalline domain. The dataset was tested for twinning and higher/lower metric symmetry (using ADDSYM and TWINROTMAT routines in PLATON software suite) and no evidence of other model errors could be found. A resolution cut off (SHEL 99 0.73) was applied to exclude poorly determined intensities at high diffraction angles. Increasing the cut off level to 0.8 Å leads to lower residual electron density. A high residual electron density was initially observed close to the Bi central atom during the initial refinement cycles. This could be caused by anharmonic displacement of the heavy atom and has been taken into account by splitting the metal atom into two parts. The atomic displacement parameters of both Bi components were restrained to be isotropic with an effective standard deviation of 0.01. The tert.-butyl entity in para-position to the substituted phenoxy group is positionally disordered. It was refined with occupancy of 60:40% and for the minor component, isotropic displacement parameters were used. In addition, the structure contains two disordered solute molecules (THF and acetonitrile). Both have been modeled by using the DSR plugin in the OLEX2 software and isotropic displacement parameters are partially applied (minor component). Hydrogen atom positions appeared as residual electron density peaks and the hydrogen atoms were partially refined without using a riding model. The observations are consistent with the characteristic NMR coupling signals.

Complete .cif-data of the compound are available under the CCDC number **CCDC-2128290**.

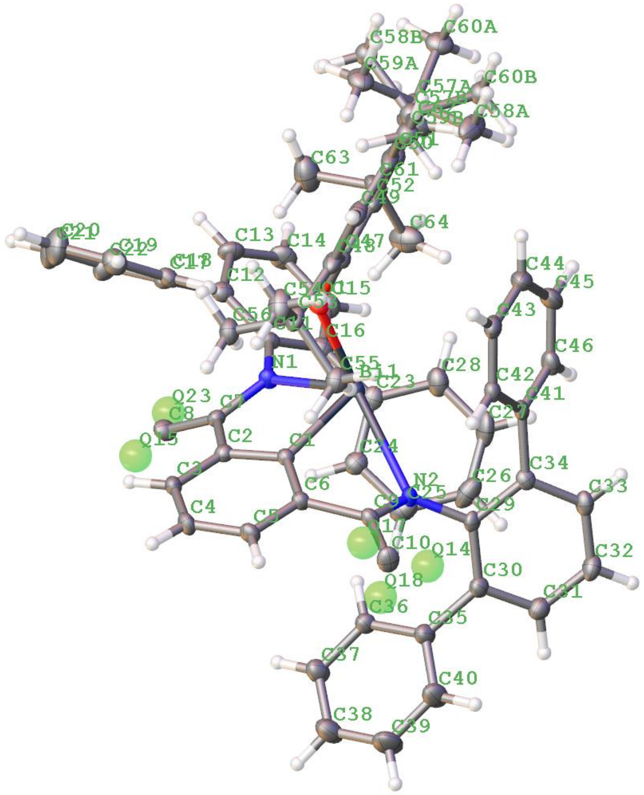

Fig. S31. Methyl and methylene hydrogen atoms of the NCN-pincer ligand could be located via residual electron density peaks (green).

Table S7. Crystal data and structure refinement of **4**

Identification code	14030	
Empirical formula	$C_{138} H_{145} Bi_2 N_5 O_4$	
Color	orange	
Formula weight	2355.54 g·mol^{-1}	
Temperature	100(2) K	
Wavelength	0.71073 Å	
Crystal system	Monoclinic	
Space group	$P2_1$/c, (no. 14)	
Unit cell dimensions	a = 11.0628(6) Å	α= 90°.
	b = 34.091(2) Å	β= 93.122(3)°.
	c = 14.6775(9) Å	γ = 90°.
Volume	5527.2(6) Å^3	
Z	2	
Density (calculated)	1.415 Mg·m^{-3}	
Absorption coefficient	3.239 mm^{-1}	
F(000)	2412 e	
Crystal size	0.063 x 0.033 x 0.033 mm^3	
θ range for data collection	1.195 to 29.131°.	
Index ranges	-15 ≤ h ≤ 15, -46≤ k ≤ 46, -20 ≤ l ≤ 20	
Reflections collected	166703	
Independent reflections	14879 [R_{int} = 0.0657]	
Reflections with I>2σ(I)	12538	
Completeness to θ = 25.242°	100.0 %	
Absorption correction	Gaussian	
Max. and min. transmission	0.92888 and 0.83913	
Refinement method	Full-matrix least-squares on F^2	
Data / restraints / parameters	14879 / 29 / 726	
Goodness-of-fit on F^2	1.250	
Final R indices [I>2σ(I)]	R_1 = 0.0495	wR^2 = 0.0824
R indices (all data)	R_1 = 0.0643	wR^2 = 0.0857
Extinction coefficient	n/a	
Largest diff. peak and hole	1.831 and -4.607 e·Å^{-3}	

Table S8. Bond lengths [Å] and angles [°] of **4**

Bi(1)-O(1)	2.178(3)	Bi(1)-N(2)	2.793(3)
Bi(1)-N(1)	2.214(3)	Bi(1)-C(1)	2.199(4)
O(1)-C(47)	1.356(5)	O(1)-Bi(2)	2.573(18)
N(2)-C(29)	1.413(5)	N(2)-C(9)	1.283(5)
N(2)-Bi(2)	2.283(17)	N(1)-C(7)	1.379(5)
N(1)-C(11)	1.410(5)	N(1)-Bi(2)	2.615(16)
C(3)-H(3)	0.9500	C(3)-C(2)	1.404(6)
C(3)-C(4)	1.382(6)	C(1)-C(2)	1.389(5)
C(1)-C(6)	1.403(5)	C(1)-Bi(2)	2.151(8)
C(29)-C(34)	1.409(6)	C(29)-C(30)	1.410(6)
C(34)-C(41)	1.486(6)	C(34)-C(33)	1.389(6)
C(2)-C(7)	1.478(5)	C(42)-H(42)	0.9500
C(42)-C(41)	1.389(6)	C(42)-C(43)	1.391(6)
C(9)-C(6)	1.492(5)	C(9)-C(10)	1.490(5)
C(7)-C(8)	1.355(6)	C(41)-C(46)	1.395(6)
C(17)-C(12)	1.478(6)	C(17)-C(18)	1.403(6)
C(17)-C(22)	1.396(6)	C(11)-C(16)	1.414(6)
C(11)-C(12)	1.417(6)	C(31)-H(31)	0.9500
C(31)-C(30)	1.389(6)	C(31)-C(32)	1.381(6)
C(33)-H(33)	0.9500	C(33)-C(32)	1.380(6)
C(36)-H(36)	0.9500	C(36)-C(35)	1.393(6)
C(36)-C(37)	1.387(6)	C(6)-C(5)	1.396(5)
C(43)-H(43)	0.9500	C(43)-C(44)	1.374(6)
C(15)-H(15)	0.9500	C(15)-C(16)	1.394(6)
C(15)-C(14)	1.379(6)	C(46)-H(46)	0.9500
C(46)-C(45)	1.380(6)	C(8)-H(8A)	0.92(5)
C(8)-H(8B)	1.00(5)	C(4)-H(4)	0.9500
C(4)-C(5)	1.383(6)	C(16)-C(23)	1.488(6)
C(12)-C(13)	1.401(6)	C(30)-C(35)	1.489(6)
C(48)-C(49)	1.393(6)	C(48)-C(53)	1.533(6)
C(48)-C(47)	1.430(6)	C(49)-H(49)	0.9500
C(49)-C(50)	1.392(7)	C(18)-H(18)	0.9500
C(18)-C(19)	1.384(6)	C(13)-H(13)	0.9500

C(13)-C(14)	1.367(6)	C(45)-H(45)	0.9500
C(45)-C(44)	1.377(7)	C(24)-H(24)	0.9500
C(24)-C(25)	1.386(6)	C(24)-C(23)	1.394(6)
C(35)-C(40)	1.399(6)	C(61)-C(52)	1.538(7)
C(61)-C(64)	1.533(7)	C(61)-C(62)	1.529(7)
C(61)-C(63)	1.525(7)	C(44)-H(44)	0.9500
C(10)-H(10A)	0.9800	C(10)-H(10B)	0.9800
C(10)-H(10C)	0.9800	C(5)-H(5)	0.9500
C(51)-H(51)	0.9500	C(51)-C(50)	1.372(7)
C(51)-C(52)	1.400(6)	C(37)-H(37)	0.9500
C(37)-C(38)	1.376(6)	C(53)-C(55)	1.534(6)
C(53)-C(56)	1.545(6)	C(53)-C(54)	1.529(6)
C(32)-H(32)	0.9500	C(55)-H(55A)	0.9800
C(55)-H(55B)	0.9800	C(55)-H(55C)	0.9800
C(25)-H(25)	0.9500	C(25)-C(26)	1.374(7)
C(14)-H(14)	0.9500	C(23)-C(28)	1.392(6)
C(50)-C(57A)	1.534(10)	C(50)-C(57B)	1.57(2)
C(22)-H(22)	0.9500	C(22)-C(21)	1.382(7)
C(28)-H(28)	0.9500	C(28)-C(27)	1.391(7)
C(52)-C(47)	1.425(6)	C(56)-H(56A)	0.9800
C(56)-H(56B)	0.9800	C(56)-H(56C)	0.9800
C(26)-H(26)	0.9500	C(26)-C(27)	1.377(7)
C(19)-H(19)	0.9500	C(19)-C(20)	1.383(7)
C(54)-H(54A)	0.9800	C(54)-H(54B)	0.9800
C(54)-H(54C)	0.9800	C(27)-H(27)	0.9500
C(57A)-C(58A)	1.522(12)	C(57A)-C(59A)	1.530(12)
C(57A)-C(60A)	1.528(13)	C(39)-H(39)	0.9500
C(39)-C(40)	1.379(7)	C(39)-C(38)	1.378(7)
C(64)-H(64A)	0.9800	C(64)-H(64B)	0.9800
C(64)-H(64C)	0.9800	C(20)-H(20)	0.9500
C(20)-C(21)	1.386(8)	C(21)-H(21)	0.9500
C(40)-H(40)	0.9500	C(62)-H(62A)	0.9800
C(62)-H(62B)	0.9800	C(62)-H(62C)	0.9800
C(38)-H(38)	0.9500	C(63)-H(63A)	0.9800
C(63)-H(63B)	0.9800	C(63)-H(63C)	0.9800

C(58A)-H(58A)	0.9800	C(58A)-H(58B)	0.9800
C(58A)-H(58C)	0.9800	C(59A)-H(59A)	0.9800
C(59A)-H(59B)	0.9800	C(59A)-H(59C)	0.9800
C(60A)-H(60A)	0.9800	C(60A)-H(60B)	0.9800
C(60A)-H(60C)	0.9800	C(67B)-H(67A)	0.9900
C(67B)-H(67B)	0.9900	C(67B)-C(68B)	1.43(2)
C(67B)-O(2B)	1.444(17)	C(60B)-H(60D)	0.9800
C(60B)-H(60E)	0.9800	C(60B)-H(60F)	0.9800
C(60B)-C(57B)	1.52(2)	C(58B)-H(58D)	0.9800
C(58B)-H(58E)	0.9800	C(58B)-H(58F)	0.9800
C(58B)-C(57B)	1.55(2)	C(59B)-H(59D)	0.9800
C(59B)-H(59E)	0.9800	C(59B)-H(59F)	0.9800
C(59B)-C(57B)	1.53(2)	N(3A)-C(66A)	1.133(8)
C(66A)-C(65)	1.441(8)	C(65)-H(65C)	0.9800
C(65)-H(65A)	0.9800	C(65)-H(65B)	0.9800
C(65)-H(65F)	0.9800	C(65)-H(65E)	0.9800
C(65)-H(65D)	0.9800	C(65)-C(66B)	1.52(2)
C(68B)-H(68A)	0.9900	C(68B)-H(68B)	0.9900
C(68B)-C(69B)	1.69(3)	O(2B)-C(70B)	1.409(15)
C(69B)-H(69A)	0.9900	C(69B)-H(69B)	0.9900
C(69B)-C(70B)	1.468(17)	C(70B)-H(70A)	0.9900
C(70B)-H(70B)	0.9900	O(2A)-C(67A)	1.54(2)
O(2A)-C(70A)	1.37(2)	C(67A)-H(67C)	0.9900
C(67A)-H(67D)	0.9900	C(67A)-C(68A)	1.42(3)
C(69A)-H(69C)	0.9900	C(69A)-H(69D)	0.9900
C(69A)-C(68A)	1.39(3)	C(69A)-C(70A)	1.62(3)
C(68A)-H(68C)	0.9900	C(68A)-H(68D)	0.9900
C(70A)-H(70C)	0.9900	C(70A)-H(70D)	0.9900
N(3B)-C(66B)	1.15(2)		
O(1)-Bi(1)-N(2)	124.24(11)	O(1)-Bi(1)-N(1)	85.65(12)
O(1)-Bi(1)-C(1)	95.07(12)	N(1)-Bi(1)-N(2)	130.38(11)
C(1)-Bi(1)-N(2)	64.34(12)	C(1)-Bi(1)-N(1)	75.48(13)
C(47)-O(1)-Bi(1)	136.2(3)	C(47)-O(1)-Bi(2)	130.0(3)
C(29)-N(2)-Bi(1)	125.6(2)	C(29)-N(2)-Bi(2)	122.1(4)

C(9)-N(2)-Bi(1)	108.9(2)	C(9)-N(2)-C(29)	125.3(3)
C(9)-N(2)-Bi(2)	112.4(4)	C(7)-N(1)-Bi(1)	116.4(2)
C(7)-N(1)-C(11)	122.5(3)	C(7)-N(1)-Bi(2)	111.5(3)
C(11)-N(1)-Bi(1)	120.6(3)	C(11)-N(1)-Bi(2)	124.1(3)
C(2)-C(3)-H(3)	120.0	C(4)-C(3)-H(3)	120.0
C(4)-C(3)-C(2)	119.9(4)	C(2)-C(1)-Bi(1)	114.4(3)
C(2)-C(1)-C(6)	121.4(4)	C(2)-C(1)-Bi(2)	126.5(5)
C(6)-C(1)-Bi(1)	124.1(3)	C(6)-C(1)-Bi(2)	111.9(5)
C(34)-C(29)-N(2)	120.0(4)	C(34)-C(29)-C(30)	119.7(4)
C(30)-C(29)-N(2)	119.4(4)	C(29)-C(34)-C(41)	122.1(4)
C(33)-C(34)-C(29)	118.9(4)	C(33)-C(34)-C(41)	119.0(4)
C(3)-C(2)-C(7)	124.4(4)	C(1)-C(2)-C(3)	118.3(4)
C(1)-C(2)-C(7)	117.1(3)	C(41)-C(42)-H(42)	119.7
C(41)-C(42)-C(43)	120.6(4)	C(43)-C(42)-H(42)	119.7
N(2)-C(9)-C(6)	115.0(3)	N(2)-C(9)-C(10)	125.3(4)
C(10)-C(9)-C(6)	119.7(3)	N(1)-C(7)-C(2)	114.2(3)
C(8)-C(7)-N(1)	124.7(4)	C(8)-C(7)-C(2)	120.8(4)
C(42)-C(41)-C(34)	122.0(4)	C(42)-C(41)-C(46)	118.2(4)
C(46)-C(41)-C(34)	119.7(4)	C(18)-C(17)-C(12)	123.4(4)
C(22)-C(17)-C(12)	118.7(4)	C(22)-C(17)-C(18)	117.7(4)
N(1)-C(11)-C(16)	118.6(4)	N(1)-C(11)-C(12)	122.1(4)
C(16)-C(11)-C(12)	119.2(4)	C(30)-C(31)-H(31)	119.2
C(32)-C(31)-H(31)	119.2	C(32)-C(31)-C(30)	121.6(4)
C(34)-C(33)-H(33)	119.1	C(32)-C(33)-C(34)	121.7(4)
C(32)-C(33)-H(33)	119.1	C(35)-C(36)-H(36)	119.4
C(37)-C(36)-H(36)	119.4	C(37)-C(36)-C(35)	121.3(4)
C(1)-C(6)-C(9)	118.3(3)	C(5)-C(6)-C(1)	118.5(4)
C(5)-C(6)-C(9)	122.5(3)	C(42)-C(43)-H(43)	119.9
C(44)-C(43)-C(42)	120.2(4)	C(44)-C(43)-H(43)	119.9
C(16)-C(15)-H(15)	119.5	C(14)-C(15)-H(15)	119.5
C(14)-C(15)-C(16)	120.9(4)	C(41)-C(46)-H(46)	119.6
C(45)-C(46)-C(41)	120.8(4)	C(45)-C(46)-H(46)	119.6
C(7)-C(8)-H(8A)	120(3)	C(7)-C(8)-H(8B)	123(3)
H(8A)-C(8)-H(8B)	118(4)	C(3)-C(4)-H(4)	119.4
C(3)-C(4)-C(5)	121.2(4)	C(5)-C(4)-H(4)	119.4

C(11)-C(16)-C(23)	121.9(4)	C(15)-C(16)-C(11)	119.7(4)
C(15)-C(16)-C(23)	118.5(4)	C(11)-C(12)-C(17)	124.1(4)
C(13)-C(12)-C(17)	117.4(4)	C(13)-C(12)-C(11)	118.4(4)
C(29)-C(30)-C(35)	123.5(4)	C(31)-C(30)-C(29)	119.0(4)
C(31)-C(30)-C(35)	117.5(4)	C(49)-C(48)-C(53)	119.0(4)
C(49)-C(48)-C(47)	117.9(4)	C(47)-C(48)-C(53)	123.2(4)
C(48)-C(49)-H(49)	118.3	C(50)-C(49)-C(48)	123.4(5)
C(50)-C(49)-H(49)	118.3	C(17)-C(18)-H(18)	119.6
C(19)-C(18)-C(17)	120.7(4)	C(19)-C(18)-H(18)	119.6
C(12)-C(13)-H(13)	119.0	C(14)-C(13)-C(12)	122.0(4)
C(14)-C(13)-H(13)	119.0	C(46)-C(45)-H(45)	119.9
C(44)-C(45)-C(46)	120.3(4)	C(44)-C(45)-H(45)	119.9
C(25)-C(24)-H(24)	119.7	C(25)-C(24)-C(23)	120.6(4)
C(23)-C(24)-H(24)	119.7	C(36)-C(35)-C(30)	123.5(4)
C(36)-C(35)-C(40)	117.6(4)	C(40)-C(35)-C(30)	118.8(4)
C(64)-C(61)-C(52)	114.5(4)	C(62)-C(61)-C(52)	112.5(4)
C(62)-C(61)-C(64)	106.0(4)	C(63)-C(61)-C(52)	108.8(4)
C(63)-C(61)-C(64)	108.2(4)	C(63)-C(61)-C(62)	106.4(4)
C(43)-C(44)-C(45)	119.9(4)	C(43)-C(44)-H(44)	120.0
C(45)-C(44)-H(44)	120.0	C(9)-C(10)-H(10A)	109.5
C(9)-C(10)-H(10B)	109.5	C(9)-C(10)-H(10C)	109.5
H(10A)-C(10)-H(10B)	109.5	H(10A)-C(10)-H(10C)	109.5
H(10B)-C(10)-H(10C)	109.5	C(6)-C(5)-H(5)	120.1
C(4)-C(5)-C(6)	119.8(4)	C(4)-C(5)-H(5)	120.1
C(50)-C(51)-H(51)	117.8	C(50)-C(51)-C(52)	124.4(5)
C(52)-C(51)-H(51)	117.8	C(36)-C(37)-H(37)	120.0
C(38)-C(37)-C(36)	119.9(4)	C(38)-C(37)-H(37)	120.0
C(48)-C(53)-C(55)	109.6(3)	C(48)-C(53)-C(56)	112.3(4)
C(55)-C(53)-C(56)	109.7(4)	C(54)-C(53)-C(48)	112.3(4)
C(54)-C(53)-C(55)	106.4(4)	C(54)-C(53)-C(56)	106.4(4)
C(31)-C(32)-H(32)	120.4	C(33)-C(32)-C(31)	119.1(4)
C(33)-C(32)-H(32)	120.4	C(53)-C(55)-H(55A)	109.5
C(53)-C(55)-H(55B)	109.5	C(53)-C(55)-H(55C)	109.5
H(55A)-C(55)-H(55B)	109.5	H(55A)-C(55)-H(55C)	109.5
H(55B)-C(55)-H(55C)	109.5	C(24)-C(25)-H(25)	119.6

C(26)-C(25)-C(24)	120.7(5)	C(26)-C(25)-H(25)	119.6
C(15)-C(14)-H(14)	120.1	C(13)-C(14)-C(15)	119.8(4)
C(13)-C(14)-H(14)	120.1	C(24)-C(23)-C(16)	121.6(4)
C(28)-C(23)-C(16)	120.1(4)	C(28)-C(23)-C(24)	118.3(4)
C(49)-C(50)-C(57A)	126.2(6)	C(49)-C(50)-C(57B)	110.8(7)
C(51)-C(50)-C(49)	116.9(4)	C(51)-C(50)-C(57A)	116.7(6)
C(51)-C(50)-C(57B)	132.2(7)	C(17)-C(22)-H(22)	119.3
C(21)-C(22)-C(17)	121.4(5)	C(21)-C(22)-H(22)	119.3
C(23)-C(28)-H(28)	119.8	C(27)-C(28)-C(23)	120.5(5)
C(27)-C(28)-H(28)	119.8	C(51)-C(52)-C(61)	118.0(4)
C(51)-C(52)-C(47)	117.2(4)	C(47)-C(52)-C(61)	124.5(4)
C(53)-C(56)-H(56A)	109.5	C(53)-C(56)-H(56B)	109.5
C(53)-C(56)-H(56C)	109.5	H(56A)-C(56)-H(56B)	109.5
H(56A)-C(56)-H(56C)	109.5	H(56B)-C(56)-H(56C)	109.5
C(25)-C(26)-H(26)	120.3	C(25)-C(26)-C(27)	119.4(4)
C(27)-C(26)-H(26)	120.3	C(18)-C(19)-H(19)	119.8
C(20)-C(19)-C(18)	120.4(5)	C(20)-C(19)-H(19)	119.8
O(1)-C(47)-C(48)	119.4(4)	O(1)-C(47)-C(52)	120.4(4)
C(52)-C(47)-C(48)	120.0(4)	C(53)-C(54)-H(54A)	109.5
C(53)-C(54)-H(54B)	109.5	C(53)-C(54)-H(54C)	109.5
H(54A)-C(54)-H(54B)	109.5	H(54A)-C(54)-H(54C)	109.5
H(54B)-C(54)-H(54C)	109.5	C(28)-C(27)-H(27)	119.7
C(26)-C(27)-C(28)	120.6(5)	C(26)-C(27)-H(27)	119.7
C(58A)-C(57A)-C(50)	106.8(7)	C(58A)-C(57A)-C(59A)	110.0(8)
C(58A)-C(57A)-C(60A)	107.8(7)	C(59A)-C(57A)-C(50)	107.8(7)
C(60A)-C(57A)-C(50)	117.1(7)	C(60A)-C(57A)-C(59A)	107.3(7)
C(40)-C(39)-H(39)	119.8	C(38)-C(39)-H(39)	119.8
C(38)-C(39)-C(40)	120.5(5)	C(61)-C(64)-H(64A)	109.5
C(61)-C(64)-H(64B)	109.5	C(61)-C(64)-H(64C)	109.5
H(64A)-C(64)-H(64B)	109.5	H(64A)-C(64)-H(64C)	109.5
H(64B)-C(64)-H(64C)	109.5	C(19)-C(20)-H(20)	120.2
C(19)-C(20)-C(21)	119.7(5)	C(21)-C(20)-H(20)	120.2
C(22)-C(21)-C(20)	119.9(5)	C(22)-C(21)-H(21)	120.0
C(20)-C(21)-H(21)	120.0	C(35)-C(40)-H(40)	119.6
C(39)-C(40)-C(35)	120.9(5)	C(39)-C(40)-H(40)	119.6

C(61)-C(62)-H(62A)	109.5	C(61)-C(62)-H(62B)	109.5
C(61)-C(62)-H(62C)	109.5	H(62A)-C(62)-H(62B)	109.5
H(62A)-C(62)-H(62C)	109.5	H(62B)-C(62)-H(62C)	109.5
C(37)-C(38)-C(39)	119.8(5)	C(37)-C(38)-H(38)	120.1
C(39)-C(38)-H(38)	120.1	C(61)-C(63)-H(63A)	109.5
C(61)-C(63)-H(63B)	109.5	C(61)-C(63)-H(63C)	109.5
H(63A)-C(63)-H(63B)	109.5	H(63A)-C(63)-H(63C)	109.5
H(63B)-C(63)-H(63C)	109.5	C(57A)-C(58A)-H(58A)	109.5
C(57A)-C(58A)-H(58B)	109.5	C(57A)-C(58A)-H(58C)	109.5
H(58A)-C(58A)-H(58B)	109.5	H(58A)-C(58A)-H(58C)	109.5
H(58B)-C(58A)-H(58C)	109.5	C(57A)-C(59A)-H(59A)	109.5
C(57A)-C(59A)-H(59B)	109.5	C(57A)-C(59A)-H(59C)	109.5
H(59A)-C(59A)-H(59B)	109.5	H(59A)-C(59A)-H(59C)	109.5
H(59B)-C(59A)-H(59C)	109.5	C(57A)-C(60A)-H(60A)	109.5
C(57A)-C(60A)-H(60B)	109.5	C(57A)-C(60A)-H(60C)	109.5
H(60A)-C(60A)-H(60B)	109.5	H(60A)-C(60A)-H(60C)	109.5
H(60B)-C(60A)-H(60C)	109.5	H(67A)-C(67B)-H(67B)	108.3
C(68B)-C(67B)-H(67A)	109.9	C(68B)-C(67B)-H(67B)	109.9
C(68B)-C(67B)-O(2B)	108.9(13)	O(2B)-C(67B)-H(67A)	109.9
O(2B)-C(67B)-H(67B)	109.9	H(60D)-C(60B)-H(60E)	109.5
H(60D)-C(60B)-H(60F)	109.5	H(60E)-C(60B)-H(60F)	109.5
C(57B)-C(60B)-H(60D)	109.5	C(57B)-C(60B)-H(60E)	109.5
C(57B)-C(60B)-H(60F)	109.5	H(58D)-C(58B)-H(58E)	109.5
H(58D)-C(58B)-H(58F)	109.5	H(58E)-C(58B)-H(58F)	109.5
C(57B)-C(58B)-H(58D)	109.5	C(57B)-C(58B)-H(58E)	109.5
C(57B)-C(58B)-H(58F)	109.5	H(59D)-C(59B)-H(59E)	109.5
H(59D)-C(59B)-H(59F)	109.5	H(59E)-C(59B)-H(59F)	109.5
C(57B)-C(59B)-H(59D)	109.5	C(57B)-C(59B)-H(59E)	109.5
C(57B)-C(59B)-H(59F)	109.5	C(60B)-C(57B)-C(50)	105.9(13)
C(60B)-C(57B)-C(58B)	108.4(13)	C(60B)-C(57B)-C(59B)	109.6(15)
C(58B)-C(57B)-C(50)	102.7(13)	C(59B)-C(57B)-C(50)	121.9(13)
C(59B)-C(57B)-C(58B)	107.6(14)	N(3A)-C(66A)-C(65)	176(2)
C(66A)-C(65)-H(65C)	109.5	C(66A)-C(65)-H(65A)	109.5
C(66A)-C(65)-H(65B)	109.5	H(65C)-C(65)-H(65A)	109.5
H(65C)-C(65)-H(65B)	109.5	H(65A)-C(65)-H(65B)	109.5

H(65F)-C(65)-H(65E)	109.5	H(65F)-C(65)-H(65D)	109.5
H(65E)-C(65)-H(65D)	109.5	C(66B)-C(65)-H(65F)	109.5
C(66B)-C(65)-H(65E)	109.5	C(66B)-C(65)-H(65D)	109.5
C(67B)-C(68B)-H(68A)	110.6	C(67B)-C(68B)-H(68B)	110.6
C(67B)-C(68B)-C(69B)	105.8(14)	H(68A)-C(68B)-H(68B)	108.7
C(69B)-C(68B)-H(68A)	110.6	C(69B)-C(68B)-H(68B)	110.6
C(70B)-O(2B)-C(67B)	109.1(12)	C(68B)-C(69B)-H(69A)	112.2
C(68B)-C(69B)-H(69B)	112.2	H(69A)-C(69B)-H(69B)	109.8
C(70B)-C(69B)-C(68B)	97.9(15)	C(70B)-C(69B)-H(69A)	112.2
C(70B)-C(69B)-H(69B)	112.2	O(2B)-C(70B)-C(69B)	114.8(16)
O(2B)-C(70B)-H(70A)	108.6	O(2B)-C(70B)-H(70B)	108.6
C(69B)-C(70B)-H(70A)	108.6	C(69B)-C(70B)-H(70B)	108.6
H(70A)-C(70B)-H(70B)	107.5	C(70A)-O(2A)-C(67A)	109.0(16)
O(2A)-C(67A)-H(67C)	111.8	O(2A)-C(67A)-H(67D)	111.8
H(67C)-C(67A)-H(67D)	109.6	C(68A)-C(67A)-O(2A)	99.6(16)
C(68A)-C(67A)-H(67C)	111.8	C(68A)-C(67A)-H(67D)	111.8
H(69C)-C(69A)-H(69D)	110.2	C(68A)-C(69A)-H(69C)	112.7
C(68A)-C(69A)-H(69D)	112.7	C(68A)-C(69A)-C(70A)	95.0(18)
C(70A)-C(69A)-H(69C)	112.7	C(70A)-C(69A)-H(69D)	112.7
C(67A)-C(68A)-H(68C)	109.6	C(67A)-C(68A)-H(68D)	109.6
C(69A)-C(68A)-C(67A)	110(2)	C(69A)-C(68A)-H(68C)	109.6
C(69A)-C(68A)-H(68D)	109.6	H(68C)-C(68A)-H(68D)	108.1
O(2A)-C(70A)-C(69A)	100.3(18)	O(2A)-C(70A)-H(70C)	111.7
O(2A)-C(70A)-H(70D)	111.7	C(69A)-C(70A)-H(70C)	111.7
C(69A)-C(70A)-H(70D)	111.7	H(70C)-C(70A)-H(70D)	109.5
N(3B)-C(66B)-C(65)	174(2)	O(1)-Bi(2)-N(1)	70.3(5)
N(2)-Bi(2)-O(1)	130.1(3)	N(2)-Bi(2)-N(1)	136.6(3)
C(1)-Bi(2)-O(1)	85.8(5)	C(1)-Bi(2)-N(2)	75.1(3)
C(1)-Bi(2)-N(1)	68.3(4)		

8.2 Single crystal structure analysis of **7**

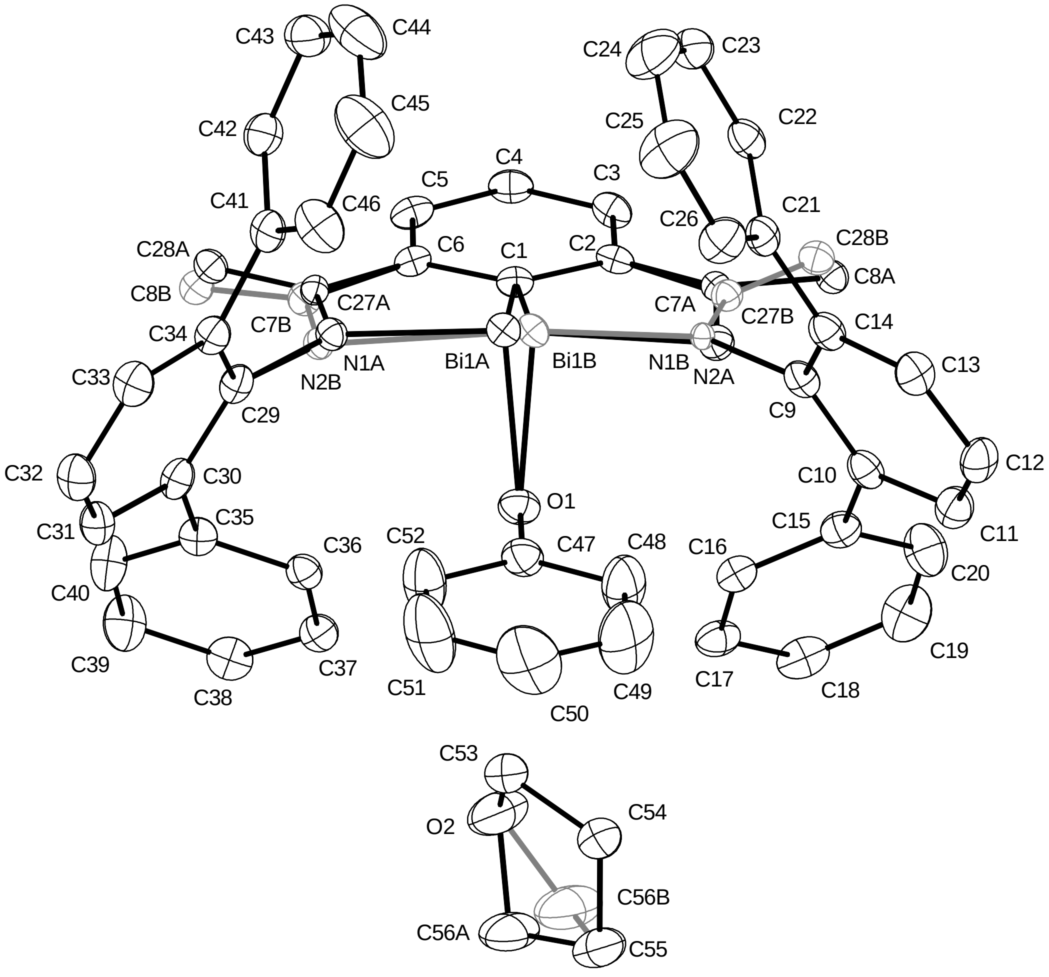

Fig. S32. The molecular structure of complex **7**. H atoms have been removed for clarity.

X-ray Crystal Structure Analysis of complex **7**:

$C_{56}H_{47}BiN_2O_2$, M_r = 988.93 g mol^{-1}, orange prism, crystal size 0.041 x 0.034 x 0.031 mm^3, monoclinic, space group $P2_1/n$ [14], a = 11.6775(5) Å, b = 19.4134(9) Å, c = 19.7788(8) Å, β = 103.406(2)°, V = 4361.7(3) Å^3, T = 100(2) K, Z = 4, D_{calc} = 1.506 g·cm^3, λ = 0.71073 Å, $\mu(Mo\text{-}K_\alpha)$ = 4.088 mm^{-1}, analytical absorption correction ($T_{\min}$ = 0.88679, $T_{\max}$ = 0.93297), Bruker-AXS Kappa Mach3 with APEX-II detector and IµS microfocus X-ray source, 1.490 < θ < 30.508°, 142154 measured reflections, 13328 independent reflections, 9079 reflections with $I > 2\sigma(I)$, R_{int} = 0.0472. The structure was solved by

SHELXT and refined by full-matrix least-squares (*SHELXL*) against F^2 to R_1 = 0.0320 [$I > 2\sigma(I)$], wR_2 = 0.0650, 625 parameters and 72 restraints.

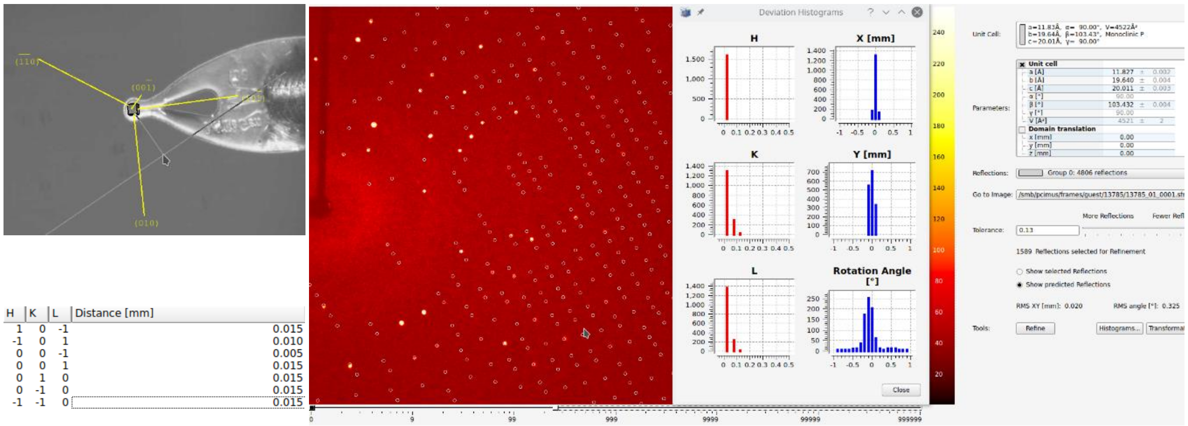

Fig. S33. Crystal faces and unit cell determination/refinement of **7**.

INTENSITY STATISTICS FOR DATASET

Resolution	#Data	#Theory	%Complete	Redundancy	Mean I	Mean I/s	Rmerge	Rsigma
Inf - 2.58	296	296	100.0	17.20	66.62	89.92	0.0145	0.0063
2.58 - 1.70	700	700	100.0	18.25	38.82	80.21	0.0194	0.0071
1.70 - 1.34	1007	1007	100.0	18.15	23.54	61.07	0.0274	0.0091
1.34 - 1.17	975	975	100.0	17.85	16.39	49.86	0.0398	0.0121
1.17 - 1.06	1018	1018	100.0	15.43	13.18	38.80	0.0501	0.0166
1.06 - 0.98	1037	1037	100.0	11.33	10.53	25.96	0.0640	0.0254
0.98 - 0.92	1055	1055	100.0	9.36	8.32	18.91	0.0801	0.0357
0.92 - 0.88	860	860	100.0	8.17	6.76	14.50	0.0971	0.0473
0.88 - 0.84	1010	1010	100.0	7.68	5.74	11.98	0.1133	0.0586
0.84 - 0.80	1261	1261	100.0	7.30	4.89	9.83	0.1358	0.0735
0.80 - 0.78	698	698	100.0	7.05	4.18	8.11	0.1653	0.0902
0.78 - 0.75	1241	1242	99.9	6.78	3.77	7.16	0.1836	0.1055
0.75 - 0.73	910	910	100.0	6.52	3.31	6.08	0.2129	0.1275
0.73 - 0.71	1055	1055	100.0	6.30	3.13	5.32	0.2350	0.1432
0.71 - 0.69	1149	1149	100.0	6.03	2.69	4.43	0.2650	0.1754
0.69 - 0.68	653	653	100.0	5.87	2.33	3.86	0.3092	0.2106
0.68 - 0.66	1375	1375	100.0	5.71	2.18	3.40	0.3349	0.2336
0.66 - 0.65	756	756	100.0	5.48	1.86	2.85	0.3929	0.2905
0.65 - 0.64	810	810	100.0	5.36	1.60	2.44	0.4256	0.3469
0.64 - 0.62	1869	2104	88.8	3.90	1.44	1.95	0.4615	0.4739
0.72 - 0.62	7142	7377	96.8	5.24	2.04	3.17	0.3407	0.2700
Inf - 0.62	19735	19971	98.8	8.82	8.33	18.34	0.0538	0.0481

The crystal comprises two enantiomers crystallizing in the centro-symmetric space group $P2_1$/n. Both enantiomers occupy similar positions in the unit cell and are described as disorder with occupancy of 50:50%. The disordered part of the NCN-pincer backbone was treated by restraining the atomic displacement parameters of the respective atoms to be isotropic with an effective standard deviation of 0.001. The structure was checked for high symmetry (mirror plane) but no further symmetry elements could be identified. The possible mirror is broken by two meta-terphenyl ligands adopting slightly different conformations. There was no evidence of additional reflections in the diffraction frames. A resolution cut off (SHEL 99 0.7) was applied to exclude poorly measured intensities at higher diffraction angles. One partially disordered (refined 63:37%) solute molecule (THF) is present in the asymmetric unit.

Complete .cif-data of the compound are available under the CCDC number **CCDC-2128291**.

Table S9. Crystal data and structure refinement of **7**.

Identification code	13785	
Empirical formula	$C_{56} H_{47} Bi N_2 O_2$	
Color	orange	
Formula weight	988.93 g·mol^{-1}	
Temperature	100(2) K	
Wavelength	0.71073 Å	
Crystal system	Monoclinic	
Space group	$P2_1$/n, (no. 14)	
Unit cell dimensions	a = 11.6775(5) Å	α= 90°.
	b = 19.4134(9) Å	β= 103.406(2)°.
	c = 19.7788(8) Å	γ = 90°.
Volume	4361.7(3) Å^3	
Z	4	
Density (calculated)	1.506 Mg·m^{-3}	
Absorption coefficient	4.088 mm^{-1}	
F(000)	1984 e	
Crystal size	0.041 x 0.034 x 0.031 mm^3	
θ range for data collection	1.490 to 30.508°.	
Index ranges	-16 ≤ h ≤ 16, -27≤ k ≤ 27, -28 ≤ l ≤ 28	
Reflections collected	142154	
Independent reflections	13328 [R_{int} = 0.0472]	
Reflections with I>2σ(I)	9079	
Completeness to θ = 25.242°	100.0 %	
Absorption correction	Gaussian	
Max. and min. transmission	0.93297 and 0.88679	
Refinement method	Full-matrix least-squares on F^2	
Data / restraints / parameters	13328 / 72 / 625	
Goodness-of-fit on F^2	1.027	
Final R indices [I>2σ(I)]	R_1 = 0.0320	wR^2 = 0.0649
R indices (all data)	R_1 = 0.0620	wR^2 = 0.0755
Extinction coefficient	n/a	
Largest diff. peak and hole	2.161 and -0.863 e·Å^{-3}	

Table S10. Bond lengths [Å] and angles [°] of **7**.

Bi(1B)-O(1)	2.132(2)	Bi(1B)-C(1)	2.196(3)
Bi(1B)-N(2B)	2.717(9)	Bi(1B)-N(1B)	2.211(9)
O(1)-C(47)	1.376(3)	O(1)-Bi(1A)	2.140(2)
N(1A)-C(27A)	1.339(17)	N(1A)-C(29)	1.483(10)
N(1A)-Bi(1A)	2.210(9)	N(2A)-C(9)	1.350(11)
N(2A)-C(7A)	1.349(18)	N(2A)-Bi(1A)	2.729(9)
C(1)-C(6)	1.396(3)	C(1)-C(2)	1.391(3)
C(1)-Bi(1A)	2.191(3)	O(2)-C(53)	1.423(4)
O(2)-C(56B)	1.443(8)	O(2)-C(56A)	1.456(12)
C(6)-C(27A)	1.427(12)	C(6)-C(5)	1.396(4)
C(6)-C(7B)	1.538(13)	C(2)-C(3)	1.401(4)
C(2)-C(7A)	1.412(12)	C(2)-C(27B)	1.563(12)
C(4)-H(4)	0.9500	C(4)-C(5)	1.382(4)
C(4)-C(3)	1.389(4)	C(16)-H(16)	0.9500
C(16)-C(17)	1.391(4)	C(16)-C(15)	1.386(4)
C(27A)-C(28A)	1.399(15)	C(5)-H(5)	0.9500
C(30)-C(29)	1.409(4)	C(30)-C(35)	1.489(4)
C(30)-C(31)	1.396(4)	C(36)-H(36)	0.9500
C(36)-C(35)	1.394(4)	C(36)-C(37)	1.387(4)
C(3)-H(3)	0.9500	C(34)-C(33)	1.393(4)
C(34)-C(29)	1.408(4)	C(34)-C(41)	1.490(4)
C(9)-C(10)	1.413(4)	C(9)-C(14)	1.405(4)
C(9)-N(1B)	1.478(10)	C(33)-H(33)	0.9500
C(33)-C(32)	1.379(4)	C(29)-N(2B)	1.357(10)
C(17)-H(17)	0.9500	C(17)-C(18)	1.384(4)
C(22)-H(22)	0.9500	C(22)-C(21)	1.392(4)
C(22)-C(23)	1.381(4)	C(35)-C(40)	1.393(4)
C(10)-C(11)	1.398(4)	C(10)-C(15)	1.493(4)
C(32)-H(32)	0.9500	C(32)-C(31)	1.381(4)
C(40)-H(40)	0.9500	C(40)-C(39)	1.387(4)
C(31)-H(31)	0.9500	C(21)-C(14)	1.484(4)
C(21)-C(26)	1.394(4)	C(14)-C(13)	1.392(4)
C(7A)-C(8A)	1.571(15)	C(37)-H(37)	0.9500
C(37)-C(38)	1.379(4)	C(41)-C(42)	1.396(4)

C(41)-C(46)	1.389(4)	C(11)-H(11)	0.9500
C(11)-C(12)	1.385(4)	C(13)-H(13)	0.9500
C(13)-C(12)	1.376(4)	C(15)-C(20)	1.387(4)
C(38)-H(38)	0.9500	C(38)-C(39)	1.378(4)
C(18)-H(18)	0.9500	C(18)-C(19)	1.378(4)
C(47)-C(52)	1.393(4)	C(47)-C(48)	1.385(4)
C(39)-H(39)	0.9500	C(12)-H(12)	0.9500
C(23)-H(23)	0.9500	C(23)-C(24)	1.381(5)
C(43)-H(43)	0.9500	C(43)-C(42)	1.389(4)
C(43)-C(44)	1.370(5)	C(26)-H(26)	0.9500
C(26)-C(25)	1.388(4)	C(42)-H(42)	0.9500
C(20)-H(20)	0.9500	C(20)-C(19)	1.388(4)
C(46)-H(46)	0.9500	C(46)-C(45)	1.392(5)
C(19)-H(19)	0.9500	C(25)-H(25)	0.9500
C(25)-C(24)	1.379(5)	C(52)-H(52)	0.9500
C(52)-C(51)	1.394(5)	C(48)-H(48)	0.9500
C(48)-C(49)	1.397(5)	C(54)-H(54A)	0.9900
C(54)-H(54B)	0.9900	C(54)-C(55)	1.533(4)
C(54)-C(53)	1.506(4)	C(44)-H(44)	0.9500
C(44)-C(45)	1.376(6)	C(24)-H(24)	0.9500
C(55)-H(55C)	0.9900	C(55)-H(55D)	0.9900
C(55)-H(55A)	0.9900	C(55)-H(55B)	0.9900
C(55)-C(56B)	1.554(10)	C(55)-C(56A)	1.441(13)
C(45)-H(45)	0.9500	C(53)-H(53A)	0.9900
C(53)-H(53B)	0.9900	C(49)-H(49)	0.9500
C(49)-C(50)	1.384(6)	C(51)-H(51)	0.9500
C(51)-C(50)	1.366(5)	C(50)-H(50)	0.9500
C(56B)-H(56A)	0.9900	C(56B)-H(56B)	0.9900
C(56A)-H(56C)	0.9900	C(56A)-H(56D)	0.9900
C(8B)-H(8BA)	0.9800	C(8B)-H(8BB)	0.9800
C(8B)-H(8BC)	0.9800	C(8B)-C(7B)	1.464(15)
C(8A)-H(8AA)	0.9800	C(8A)-H(8AB)	0.9800
C(8A)-H(8AC)	0.9800	C(28B)-H(28A)	0.9500
C(28B)-H(28B)	0.9500	C(28B)-C(27B)	1.290(15)
C(28A)-H(28C)	0.9500	C(28A)-H(28D)	0.9500

C(27B)-N(1B)	1.340(17)	C(7B)-N(2B)	1.323(18)
O(1)-Bi(1B)-C(1)	102.84(9)	O(1)-Bi(1B)-N(2B)	81.7(2)
O(1)-Bi(1B)-N(1B)	91.6(3)	C(1)-Bi(1B)-N(2B)	68.1(2)
C(1)-Bi(1B)-N(1B)	74.2(3)	N(1B)-Bi(1B)-N(2B)	139.0(3)
C(47)-O(1)-Bi(1B)	113.96(17)	C(47)-O(1)-Bi(1A)	114.15(17)
C(27A)-N(1A)-C(29)	119.3(8)	C(27A)-N(1A)-Bi(1A)	116.7(8)
C(29)-N(1A)-Bi(1A)	124.0(6)	C(9)-N(2A)-Bi(1A)	122.9(6)
C(7A)-N(2A)-C(9)	130.8(9)	C(7A)-N(2A)-Bi(1A)	105.0(7)
C(6)-C(1)-Bi(1B)	123.54(18)	C(6)-C(1)-Bi(1A)	114.19(18)
C(2)-C(1)-Bi(1B)	113.70(17)	C(2)-C(1)-C(6)	122.2(2)
C(2)-C(1)-Bi(1A)	123.01(18)	C(53)-O(2)-C(56B)	112.6(4)
C(53)-O(2)-C(56A)	99.3(6)	C(1)-C(6)-C(27A)	117.6(6)
C(1)-C(6)-C(5)	118.3(2)	C(1)-C(6)-C(7B)	120.1(6)
C(5)-C(6)-C(27A)	124.1(6)	C(5)-C(6)-C(7B)	121.5(6)
C(1)-C(2)-C(3)	118.1(2)	C(1)-C(2)-C(7A)	119.2(6)
C(1)-C(2)-C(27B)	119.8(5)	C(3)-C(2)-C(7A)	122.6(6)
C(3)-C(2)-C(27B)	121.9(5)	C(5)-C(4)-H(4)	119.6
C(5)-C(4)-C(3)	120.7(2)	C(3)-C(4)-H(4)	119.6
C(17)-C(16)-H(16)	119.7	C(15)-C(16)-H(16)	119.7
C(15)-C(16)-C(17)	120.7(3)	N(1A)-C(27A)-C(6)	115.7(9)
N(1A)-C(27A)-C(28A)	125.3(11)	C(28A)-C(27A)-C(6)	118.9(10)
C(6)-C(5)-H(5)	119.8	C(4)-C(5)-C(6)	120.3(2)
C(4)-C(5)-H(5)	119.8	C(29)-C(30)-C(35)	123.2(2)
C(31)-C(30)-C(29)	118.7(2)	C(31)-C(30)-C(35)	118.1(2)
C(35)-C(36)-H(36)	119.9	C(37)-C(36)-H(36)	119.9
C(37)-C(36)-C(35)	120.2(2)	C(2)-C(3)-H(3)	119.9
C(4)-C(3)-C(2)	120.2(2)	C(4)-C(3)-H(3)	119.9
C(33)-C(34)-C(29)	119.6(2)	C(33)-C(34)-C(41)	118.6(2)
C(29)-C(34)-C(41)	121.7(2)	N(2A)-C(9)-C(10)	118.7(5)
N(2A)-C(9)-C(14)	121.9(5)	C(10)-C(9)-N(1B)	123.7(4)
C(14)-C(9)-C(10)	119.4(2)	C(14)-C(9)-N(1B)	116.7(4)
C(34)-C(33)-H(33)	119.6	C(32)-C(33)-C(34)	120.8(3)
C(32)-C(33)-H(33)	119.6	C(30)-C(29)-N(1A)	124.9(4)
C(34)-C(29)-N(1A)	115.2(4)	C(34)-C(29)-C(30)	119.6(2)

N(2B)-C(29)-C(30)	119.2(5)	N(2B)-C(29)-C(34)	121.1(5)
C(16)-C(17)-H(17)	119.8	C(18)-C(17)-C(16)	120.3(3)
C(18)-C(17)-H(17)	119.8	C(21)-C(22)-H(22)	119.6
C(23)-C(22)-H(22)	119.6	C(23)-C(22)-C(21)	120.9(3)
C(36)-C(35)-C(30)	121.8(2)	C(40)-C(35)-C(30)	119.6(2)
C(40)-C(35)-C(36)	118.5(2)	C(9)-C(10)-C(15)	123.1(2)
C(11)-C(10)-C(9)	118.9(2)	C(11)-C(10)-C(15)	118.0(2)
C(33)-C(32)-H(32)	120.2	C(33)-C(32)-C(31)	119.6(3)
C(31)-C(32)-H(32)	120.2	C(35)-C(40)-H(40)	119.6
C(39)-C(40)-C(35)	120.9(3)	C(39)-C(40)-H(40)	119.6
C(30)-C(31)-H(31)	119.2	C(32)-C(31)-C(30)	121.6(3)
C(32)-C(31)-H(31)	119.2	C(22)-C(21)-C(14)	122.0(2)
C(22)-C(21)-C(26)	118.5(3)	C(26)-C(21)-C(14)	119.4(2)
C(9)-C(14)-C(21)	121.5(2)	C(13)-C(14)-C(9)	119.6(2)
C(13)-C(14)-C(21)	118.9(2)	N(2A)-C(7A)-C(2)	121.4(10)
N(2A)-C(7A)-C(8A)	114.8(9)	C(2)-C(7A)-C(8A)	122.6(9)
C(36)-C(37)-H(37)	119.6	C(38)-C(37)-C(36)	120.7(3)
C(38)-C(37)-H(37)	119.6	C(42)-C(41)-C(34)	121.0(3)
C(46)-C(41)-C(34)	120.1(3)	C(46)-C(41)-C(42)	118.7(3)
C(10)-C(11)-H(11)	119.2	C(12)-C(11)-C(10)	121.6(3)
C(12)-C(11)-H(11)	119.2	C(14)-C(13)-H(13)	119.2
C(12)-C(13)-C(14)	121.6(3)	C(12)-C(13)-H(13)	119.2
C(16)-C(15)-C(10)	121.5(2)	C(16)-C(15)-C(20)	118.4(3)
C(20)-C(15)-C(10)	119.9(2)	C(37)-C(38)-H(38)	120.2
C(39)-C(38)-C(37)	119.6(3)	C(39)-C(38)-H(38)	120.2
C(17)-C(18)-H(18)	120.3	C(19)-C(18)-C(17)	119.3(3)
C(19)-C(18)-H(18)	120.3	O(1)-C(47)-C(52)	120.5(3)
O(1)-C(47)-C(48)	120.0(3)	C(48)-C(47)-C(52)	119.5(3)
C(40)-C(39)-H(39)	120.0	C(38)-C(39)-C(40)	120.1(3)
C(38)-C(39)-H(39)	120.0	C(11)-C(12)-H(12)	120.5
C(13)-C(12)-C(11)	119.0(3)	C(13)-C(12)-H(12)	120.5
C(22)-C(23)-H(23)	120.0	C(24)-C(23)-C(22)	120.0(3)
C(24)-C(23)-H(23)	120.0	C(42)-C(43)-H(43)	119.7
C(44)-C(43)-H(43)	119.7	C(44)-C(43)-C(42)	120.5(3)
C(21)-C(26)-H(26)	119.7	C(25)-C(26)-C(21)	120.5(3)

C(25)-C(26)-H(26)	119.7	C(41)-C(42)-H(42)	119.9
C(43)-C(42)-C(41)	120.1(3)	C(43)-C(42)-H(42)	119.9
C(15)-C(20)-H(20)	119.5	C(15)-C(20)-C(19)	121.0(3)
C(19)-C(20)-H(20)	119.5	C(41)-C(46)-H(46)	119.8
C(41)-C(46)-C(45)	120.5(3)	C(45)-C(46)-H(46)	119.8
C(18)-C(19)-C(20)	120.3(3)	C(18)-C(19)-H(19)	119.9
C(20)-C(19)-H(19)	119.9	C(26)-C(25)-H(25)	120.0
C(24)-C(25)-C(26)	120.0(3)	C(24)-C(25)-H(25)	120.0
C(47)-C(52)-H(52)	120.1	C(47)-C(52)-C(51)	119.8(3)
C(51)-C(52)-H(52)	120.1	C(47)-C(48)-H(48)	120.3
C(47)-C(48)-C(49)	119.5(3)	C(49)-C(48)-H(48)	120.3
H(54A)-C(54)-H(54B)	109.1	C(55)-C(54)-H(54A)	111.1
C(55)-C(54)-H(54B)	111.1	C(53)-C(54)-H(54A)	111.1
C(53)-C(54)-H(54B)	111.1	C(53)-C(54)-C(55)	103.2(3)
C(43)-C(44)-H(44)	120.0	C(43)-C(44)-C(45)	120.1(3)
C(45)-C(44)-H(44)	120.0	C(23)-C(24)-H(24)	119.9
C(25)-C(24)-C(23)	120.1(3)	C(25)-C(24)-H(24)	119.9
C(54)-C(55)-H(55C)	111.4	C(54)-C(55)-H(55D)	111.4
C(54)-C(55)-H(55A)	111.2	C(54)-C(55)-H(55B)	111.2
C(54)-C(55)-C(56B)	102.0(3)	H(55C)-C(55)-H(55D)	109.2
H(55A)-C(55)-H(55B)	109.1	C(56B)-C(55)-H(55C)	111.4
C(56B)-C(55)-H(55D)	111.4	C(56A)-C(55)-C(54)	102.7(4)
C(56A)-C(55)-H(55A)	111.2	C(56A)-C(55)-H(55B)	111.2
C(46)-C(45)-H(45)	120.0	C(44)-C(45)-C(46)	120.1(3)
C(44)-C(45)-H(45)	120.0	O(2)-C(53)-C(54)	105.9(2)
O(2)-C(53)-H(53A)	110.6	O(2)-C(53)-H(53B)	110.6
C(54)-C(53)-H(53A)	110.6	C(54)-C(53)-H(53B)	110.6
H(53A)-C(53)-H(53B)	108.7	C(48)-C(49)-H(49)	119.4
C(50)-C(49)-C(48)	121.1(3)	C(50)-C(49)-H(49)	119.4
C(52)-C(51)-H(51)	119.4	C(50)-C(51)-C(52)	121.2(4)
C(50)-C(51)-H(51)	119.4	C(49)-C(50)-H(50)	120.5
C(51)-C(50)-C(49)	118.9(4)	C(51)-C(50)-H(50)	120.5
O(2)-C(56B)-C(55)	103.0(7)	O(2)-C(56B)-H(56A)	111.2
O(2)-C(56B)-H(56B)	111.2	C(55)-C(56B)-H(56A)	111.2
C(55)-C(56B)-H(56B)	111.2	H(56A)-C(56B)-H(56B)	109.1

O(2)-C(56A)-H(56C)	110.1	O(2)-C(56A)-H(56D)	110.1
C(55)-C(56A)-O(2)	108.2(8)	C(55)-C(56A)-H(56C)	110.1
C(55)-C(56A)-H(56D)	110.1	H(56C)-C(56A)-H(56D)	108.4
O(1)-Bi(1A)-N(1A)	92.6(3)	O(1)-Bi(1A)-N(2A)	81.3(2)
O(1)-Bi(1A)-C(1)	102.70(9)	N(1A)-Bi(1A)-N(2A)	139.4(4)
C(1)-Bi(1A)-N(1A)	74.2(3)	C(1)-Bi(1A)-N(2A)	68.3(3)
H(8BA)-C(8B)-H(8BB)	109.5	H(8BA)-C(8B)-H(8BC)	109.5
H(8BB)-C(8B)-H(8BC)	109.5	C(7B)-C(8B)-H(8BA)	109.5
C(7B)-C(8B)-H(8BB)	109.5	C(7B)-C(8B)-H(8BC)	109.5
C(7A)-C(8A)-H(8AA)	109.5	C(7A)-C(8A)-H(8AB)	109.5
C(7A)-C(8A)-H(8AC)	109.5	H(8AA)-C(8A)-H(8AB)	109.5
H(8AA)-C(8A)-H(8AC)	109.5	H(8AB)-C(8A)-H(8AC)	109.5
H(28A)-C(28B)-H(28B)	120.0	C(27B)-C(28B)-H(28A)	120.0
C(27B)-C(28B)-H(28B)	120.0	C(27A)-C(28A)-H(28C)	120.0
C(27A)-C(28A)-H(28D)	120.0	H(28C)-C(28A)-H(28D)	120.0
C(28B)-C(27B)-C(2)	116.2(10)	C(28B)-C(27B)-N(1B)	134.0(11)
N(1B)-C(27B)-C(2)	107.4(9)	C(8B)-C(7B)-C(6)	121.2(10)
N(2B)-C(7B)-C(6)	114.3(10)	N(2B)-C(7B)-C(8B)	124.5(11)
C(29)-N(2B)-Bi(1B)	122.3(6)	C(7B)-N(2B)-Bi(1B)	112.1(7)
C(7B)-N(2B)-C(29)	125.4(9)	C(9)-N(1B)-Bi(1B)	125.0(6)
C(27B)-N(1B)-Bi(1B)	122.9(8)	C(27B)-N(1B)-C(9)	112.1(8)

8.3 Single crystal structure analysis of **15**

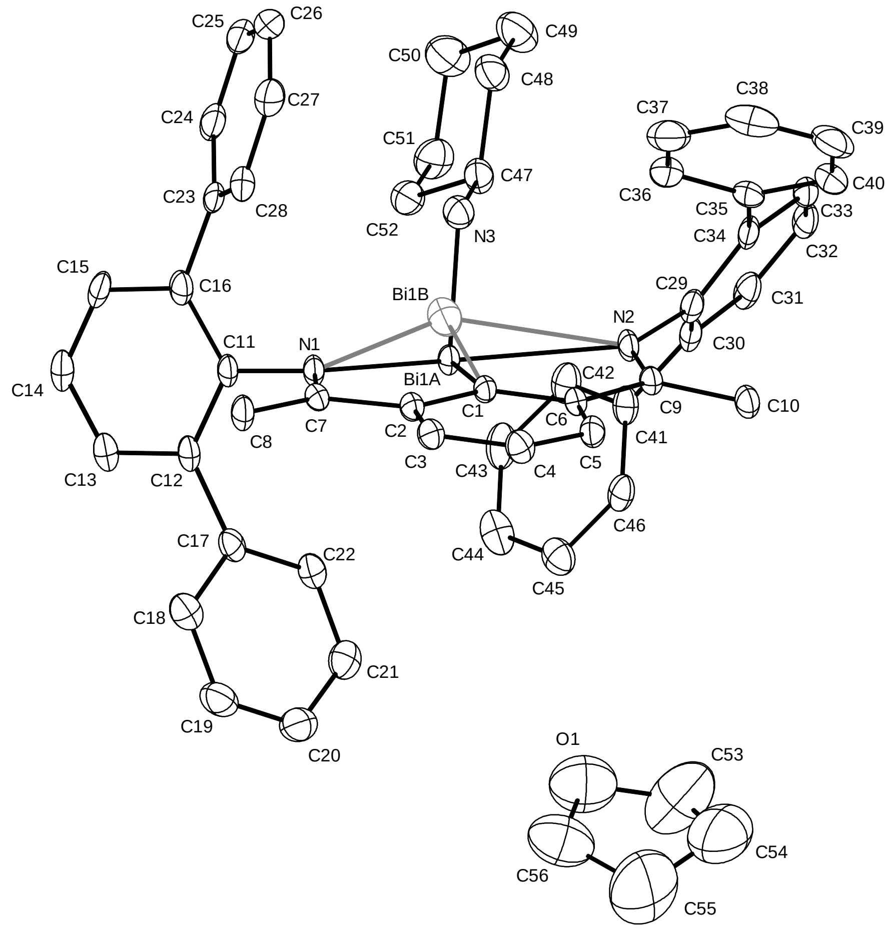

Fig. S34. The molecular structure of complex **15**. H atoms have been removed for clarity.

X-ray Crystal Structure Analysis of complex **15**:

$C_{56}H_{54}BiN_3O$, M_r = 994.00 g mol^{-1}, orange-yellow prism, crystal size 0.076 x 0.047 x 0.022 mm^3, triclinic, space group P-1 [2], a = 12.1225(7) Å, b = 13.4664(8) Å, c = 15.6630(9) Å, α = 103.603(3)°, β = 110.487(3)°, γ = 100.739(3)°, V = 2224.4(2) Å^3, T = 100(2) K, Z = 2, D_{calc} = 1.484 g·cm^3, λ = 0.71073 Å, $\mu(Mo\text{-}K_\alpha)$ = 4.007 mm^{-1}, analytical absorption correction ($T_{\min}$ = 0.84218, $T_{\max}$ = 0.93230), Bruker-AXS Kappa Mach3 with APEX-II detector and IμS microfocus X-ray source, 1.468 < θ < 27.485°, 59309 measured reflections, 10211 independent reflections, 8996 reflections with $I > 2\sigma(I)$, R_{int} = 0.0484. The structure was solved by *SHELXT* and refined by full-matrix least-squares (*SHELXL*) against F^2 to R_1 = 0.0357 [$I > 2\sigma(I)$], wR_2 = 0.0853, 584 parameters and 12 restraints.

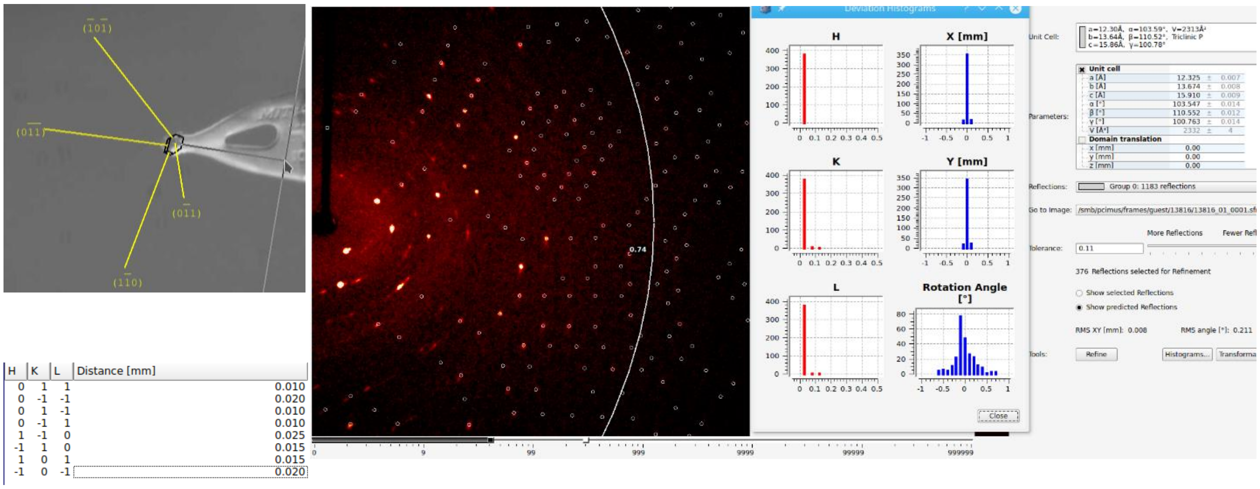

Fig. S35. Crystal faces and unit cell determination/refinement of **15**.

INTENSITY STATISTICS FOR DATASET

Resolution	#Data	#Theory	%Complete	Redundancy	Mean I	Mean I/s	Rmerge	Rsigma
Inf - 2.58	296	296	100.0	17.20	66.62	89.92	0.0145	0.0063
2.58 - 1.70	700	700	100.0	18.25	38.82	80.21	0.0194	0.0071
1.70 - 1.34	1007	1007	100.0	18.15	23.54	61.07	0.0274	0.0091
1.34 - 1.17	975	975	100.0	17.85	16.39	49.86	0.0398	0.0121
1.17 - 1.06	1018	1018	100.0	15.43	13.18	38.80	0.0501	0.0166
1.06 - 0.98	1037	1037	100.0	11.33	10.53	25.96	0.0640	0.0254
0.98 - 0.92	1055	1055	100.0	9.36	8.32	18.91	0.0801	0.0357
0.92 - 0.88	860	860	100.0	8.17	6.76	14.50	0.0971	0.0473
0.88 - 0.84	1010	1010	100.0	7.68	5.74	11.98	0.1133	0.0586
0.84 - 0.80	1261	1261	100.0	7.30	4.89	9.83	0.1358	0.0735
0.80 - 0.78	698	698	100.0	7.05	4.18	8.11	0.1653	0.0902
0.78 - 0.75	1241	1242	99.9	6.78	3.77	7.16	0.1836	0.1055
0.75 - 0.73	910	910	100.0	6.52	3.31	6.08	0.2129	0.1275
0.73 - 0.71	1055	1055	100.0	6.30	3.13	5.32	0.2350	0.1432
0.71 - 0.69	1149	1149	100.0	6.03	2.69	4.43	0.2650	0.1754
0.69 - 0.68	653	653	100.0	5.87	2.33	3.86	0.3092	0.2106
0.68 - 0.66	1375	1375	100.0	5.71	2.18	3.40	0.3349	0.2336
0.66 - 0.65	756	756	100.0	5.48	1.86	2.85	0.3929	0.2905
0.65 - 0.64	810	810	100.0	5.36	1.60	2.44	0.4256	0.3469
0.64 - 0.62	1869	2104	88.8	3.90	1.44	1.95	0.4615	0.4739
0.72 - 0.62	7142	7377	96.8	5.24	2.04	3.17	0.3407	0.2700
Inf - 0.62	19735	19971	98.8	8.82	8.33	18.34	0.0538	0.0481

The high residual electron density close to the Bi central atom could be caused by anharmonic displacement. This was taken into account by splitting the atom into two parts. Atomic displacement ellipsoids of the two components were restrained to be isotropic with an effective standard deviation of 0.001. There is no evidence of a second partial cyclohexylamine molecule in the remaining electron density map. We cannot rule out the possibility that the crystal is contaminated with Bi containing side products. Hydrogen positions could be found in a residual electron density map and were partially refined without using a riding model. The structure is consistent with the characteristic NMR coupling signals.

Complete .cif-data of the compound are available under the CCDC number **CCDC-2128288**.

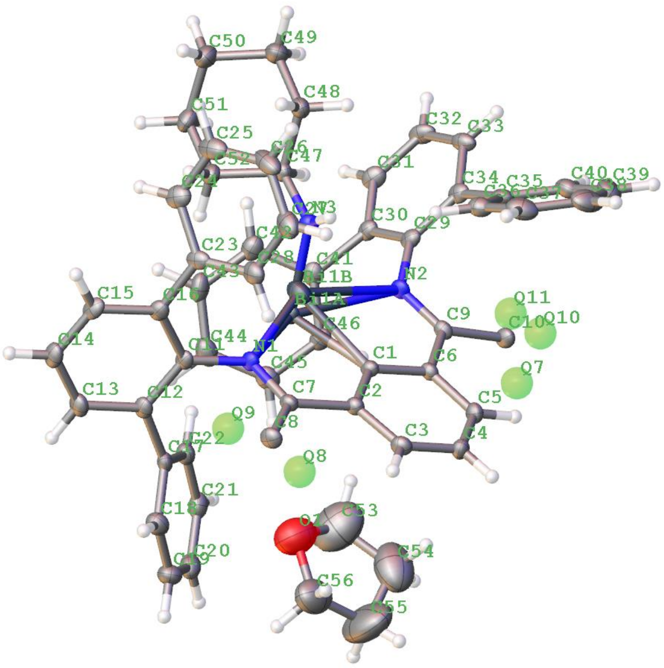

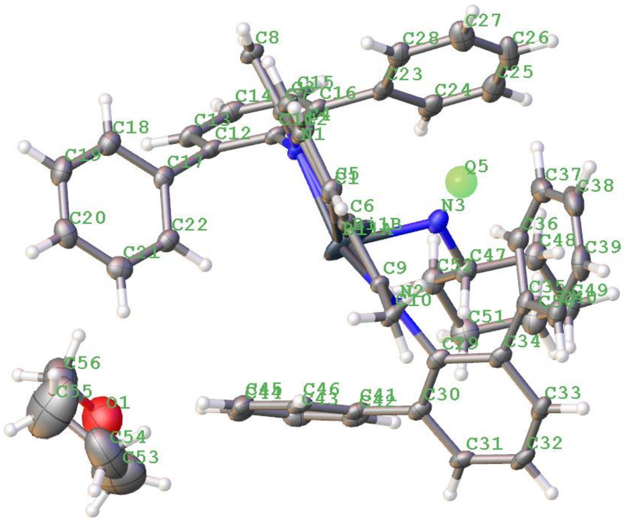

Fig. S36. There was evidence of hydrogen atoms attached to N3, C8 and C10 in the residual electron density map (e.g., shown bottom as a green circle for the hydrogen atom bonded to N3). The position and isotropic atomic displacement parameters of the hydrogen atoms could be successfully refined.

Table S11. Crystal data and structure refinement of **15**.

Identification code	13816	
Empirical formula	C_{56} H_{54} Bi N_3 O	
Color	orange-yellow	
Formula weight	994.00 g·mol^{-1}	
Temperature	100(2) K	
Wavelength	0.71073 Å	
Crystal system	Triclinic	
Space group	*P*-1, (no. 2)	
Unit cell dimensions	a = 12.1225(7) Å	α= 103.603(3)°.
	b = 13.4664(8) Å	β= 110.487(3)°.
	c = 15.6630(9) Å	γ = 100.739(3)°.
Volume	2224.4(2) Å^3	
Z	2	
Density (calculated)	1.484 Mg·m^{-3}	
Absorption coefficient	4.007 mm^{-1}	
F(000)	1004 e	
Crystal size	0.076 x 0.047 x 0.022 mm^3	
θ range for data collection	1.468 to 27.485°.	
Index ranges	-15 ≤ h ≤ 15, -17≤ k ≤ 17, -20 ≤ l ≤ 20	
Reflections collected	59309	
Independent reflections	10211 [R_{int} = 0.0484]	
Reflections with I>2σ(I)	8996	
Completeness to θ = 25.242°	100.0 %	
Absorption correction	Gaussian	
Max. and min. transmission	0.93230 and 0.84218	
Refinement method	Full-matrix least-squares on F^2	
Data / restraints / parameters	10211 / 12 / 584	
Goodness-of-fit on F^2	1.065	
Final R indices [I>2σ(I)]	R_1 = 0.0357	wR^2 = 0.0853
R indices (all data)	R_1 = 0.0446	wR^2 = 0.0892
Extinction coefficient	n/a	
Largest diff. peak and hole	3.309 and -2.022 e·Å^{-3}	

Table S12. Bond lengths [Å] and angles [°] of **15**.

Bi(1A)-N(1)	2.254(3)	Bi(1A)-N(2)	2.665(3)
Bi(1A)-N(3)	2.161(3)	Bi(1A)-C(1)	2.191(4)
N(1)-C(11)	1.427(5)	N(1)-C(7)	1.368(5)
N(1)-Bi(1B)	2.234(4)	N(2)-C(9)	1.284(5)
N(2)-C(29)	1.412(5)	N(2)-Bi(1B)	2.722(4)
N(3)-C(47)	1.482(6)	N(3)-H(3)	0.98(5)
C(5)-H(5)	0.9500	C(5)-C(4)	1.384(5)
C(5)-C(6)	1.390(6)	C(46)-H(46)	0.9500
C(46)-C(41)	1.393(6)	C(46)-C(45)	1.387(6)
C(4)-H(4)	0.9500	C(4)-C(3)	1.381(5)
C(8)-C(7)	1.352(5)	C(8)-H(8A)	0.91(5)
C(8)-H(8B)	0.91(5)	C(11)-C(16)	1.409(6)
C(11)-C(12)	1.411(5)	C(17)-C(22)	1.393(6)
C(17)-C(12)	1.491(6)	C(17)-C(18)	1.398(6)
C(9)-C(10)	1.496(5)	C(9)-C(6)	1.480(5)
C(10)-H(10A)	0.91(5)	C(10)-H(10B)	1.01(5)
C(10)-H(10C)	0.92(5)	C(15)-H(15)	0.9500
C(15)-C(16)	1.403(5)	C(15)-C(14)	1.377(6)
C(3)-H(3A)	0.9500	C(3)-C(2)	1.388(6)
C(16)-C(23)	1.481(6)	C(6)-C(1)	1.400(5)
C(22)-H(22)	0.9500	C(22)-C(21)	1.384(6)
C(34)-C(35)	1.478(6)	C(34)-C(29)	1.413(6)
C(34)-C(33)	1.400(6)	C(1)-C(2)	1.392(5)
C(1)-Bi(1B)	2.212(5)	C(35)-C(36)	1.399(6)
C(35)-C(40)	1.395(6)	C(23)-C(24)	1.397(6)
C(23)-C(28)	1.388(6)	C(7)-C(2)	1.494(5)
C(12)-C(13)	1.394(5)	C(52)-H(52A)	0.9900
C(52)-H(52B)	0.9900	C(52)-C(47)	1.529(6)
C(52)-C(51)	1.521(7)	C(29)-C(30)	1.400(6)
C(42)-H(42)	0.9500	C(42)-C(41)	1.391(6)
C(42)-C(43)	1.390(6)	C(13)-H(13)	0.9500
C(13)-C(14)	1.379(6)	C(14)-H(14)	0.9500
C(24)-H(24)	0.9500	C(24)-C(25)	1.381(6)

C(41)-C(30)	1.492(6)	C(36)-H(36)	0.9500
C(36)-C(37)	1.378(7)	C(30)-C(31)	1.394(5)
C(18)-H(18)	0.9500	C(18)-C(19)	1.371(6)
C(33)-H(33)	0.9500	C(33)-C(32)	1.371(6)
C(31)-H(31)	0.9500	C(31)-C(32)	1.387(6)
C(40)-H(40)	0.9500	C(40)-C(39)	1.388(7)
C(28)-H(28)	0.9500	C(28)-C(27)	1.391(6)
C(32)-H(32)	0.9500	C(45)-H(45)	0.9500
C(45)-C(44)	1.392(7)	C(47)-H(47)	1.0000
C(47)-C(48)	1.520(6)	C(50)-H(50A)	0.9900
C(50)-H(50B)	0.9900	C(50)-C(51)	1.515(7)
C(50)-C(49)	1.515(7)	C(27)-H(27)	0.9500
C(27)-C(26)	1.381(7)	C(51)-H(51A)	0.9900
C(51)-H(51B)	0.9900	C(19)-H(19)	0.9500
C(19)-C(20)	1.382(7)	C(21)-H(21)	0.9500
C(21)-C(20)	1.381(6)	C(48)-H(48A)	0.9900
C(48)-H(48B)	0.9900	C(48)-C(49)	1.517(7)
C(25)-H(25)	0.9500	C(25)-C(26)	1.371(8)
C(20)-H(20)	0.9500	C(43)-H(43)	0.9500
C(43)-C(44)	1.379(7)	C(44)-H(44)	0.9500
C(26)-H(26)	0.9500	C(37)-H(37)	0.9500
C(37)-C(38)	1.391(8)	C(39)-H(39)	0.9500
C(39)-C(38)	1.366(8)	C(38)-H(38)	0.9500
O(1)-C(56)	1.451(9)	O(1)-C(53)	1.451(11)
C(49)-H(49A)	0.9900	C(49)-H(49B)	0.9900
C(56)-H(56A)	0.9900	C(56)-H(56B)	0.9900
C(56)-C(55)	1.456(12)	C(54)-H(54A)	0.9900
C(54)-H(54B)	0.9900	C(54)-C(53)	1.489(13)
C(54)-C(55)	1.495(14)	C(53)-H(53A)	0.9900
C(53)-H(53B)	0.9900	C(55)-H(55A)	0.9900
C(55)-H(55B)	0.9900		

N(1)-Bi(1A)-N(2)	142.16(11)	N(3)-Bi(1A)-N(1)	96.79(12)
N(3)-Bi(1A)-N(2)	81.52(11)	N(3)-Bi(1A)-C(1)	99.36(14)
C(1)-Bi(1A)-N(1)	74.96(12)	C(1)-Bi(1A)-N(2)	68.14(12)

C(11)-N(1)-Bi(1A)	122.8(2)	C(11)-N(1)-Bi(1B)	124.8(3)
C(7)-N(1)-Bi(1A)	116.8(2)	C(7)-N(1)-C(11)	119.0(3)
C(7)-N(1)-Bi(1B)	115.8(2)	C(9)-N(2)-Bi(1A)	111.1(2)
C(9)-N(2)-C(29)	123.4(3)	C(9)-N(2)-Bi(1B)	112.2(2)
C(29)-N(2)-Bi(1A)	125.4(2)	C(29)-N(2)-Bi(1B)	122.8(2)
Bi(1A)-N(3)-H(3)	119(3)	C(47)-N(3)-Bi(1A)	110.7(3)
C(47)-N(3)-H(3)	130(3)	C(4)-C(5)-H(5)	120.0
C(4)-C(5)-C(6)	120.0(3)	C(6)-C(5)-H(5)	120.0
C(41)-C(46)-H(46)	119.8	C(45)-C(46)-H(46)	119.8
C(45)-C(46)-C(41)	120.4(4)	C(5)-C(4)-H(4)	119.6
C(3)-C(4)-C(5)	120.7(4)	C(3)-C(4)-H(4)	119.6
C(7)-C(8)-H(8A)	121(3)	C(7)-C(8)-H(8B)	117(3)
H(8A)-C(8)-H(8B)	122(4)	C(16)-C(11)-N(1)	120.4(3)
C(16)-C(11)-C(12)	119.4(3)	C(12)-C(11)-N(1)	120.2(3)
C(22)-C(17)-C(12)	122.3(4)	C(22)-C(17)-C(18)	118.0(4)
C(18)-C(17)-C(12)	119.4(4)	N(2)-C(9)-C(10)	124.9(3)
N(2)-C(9)-C(6)	116.8(3)	C(6)-C(9)-C(10)	118.3(3)
C(9)-C(10)-H(10A)	112(3)	C(9)-C(10)-H(10B)	105(3)
C(9)-C(10)-H(10C)	106(3)	H(10A)-C(10)-H(10B)	107(4)
H(10A)-C(10)-H(10C)	108(4)	H(10B)-C(10)-H(10C)	119(4)
C(16)-C(15)-H(15)	119.3	C(14)-C(15)-H(15)	119.3
C(14)-C(15)-C(16)	121.3(4)	C(4)-C(3)-H(3A)	119.7
C(4)-C(3)-C(2)	120.6(4)	C(2)-C(3)-H(3A)	119.7
C(11)-C(16)-C(23)	123.5(3)	C(15)-C(16)-C(11)	118.9(4)
C(15)-C(16)-C(23)	117.6(4)	C(5)-C(6)-C(9)	121.9(3)
C(5)-C(6)-C(1)	118.7(3)	C(1)-C(6)-C(9)	119.4(3)
C(17)-C(22)-H(22)	119.4	C(21)-C(22)-C(17)	121.1(4)
C(21)-C(22)-H(22)	119.4	C(29)-C(34)-C(35)	123.1(4)
C(33)-C(34)-C(35)	118.7(4)	C(33)-C(34)-C(29)	118.2(4)
C(6)-C(1)-Bi(1A)	122.2(3)	C(6)-C(1)-Bi(1B)	124.0(3)
C(2)-C(1)-Bi(1A)	115.8(3)	C(2)-C(1)-C(6)	121.5(4)
C(2)-C(1)-Bi(1B)	113.5(3)	C(36)-C(35)-C(34)	121.5(4)
C(40)-C(35)-C(34)	119.9(4)	C(40)-C(35)-C(36)	118.6(4)
C(24)-C(23)-C(16)	119.7(4)	C(28)-C(23)-C(16)	122.3(4)
C(28)-C(23)-C(24)	118.0(4)	N(1)-C(7)-C(2)	114.1(3)

C(8)-C(7)-N(1)	124.7(4)	C(8)-C(7)-C(2)	121.3(4)
C(11)-C(12)-C(17)	124.2(3)	C(13)-C(12)-C(11)	119.6(4)
C(13)-C(12)-C(17)	116.2(3)	H(52A)-C(52)-H(52B)	107.9
C(47)-C(52)-H(52A)	109.1	C(47)-C(52)-H(52B)	109.1
C(51)-C(52)-H(52A)	109.1	C(51)-C(52)-H(52B)	109.1
C(51)-C(52)-C(47)	112.3(4)	N(2)-C(29)-C(34)	121.0(4)
C(30)-C(29)-N(2)	118.5(3)	C(30)-C(29)-C(34)	120.1(4)
C(3)-C(2)-C(1)	118.5(3)	C(3)-C(2)-C(7)	123.5(3)
C(1)-C(2)-C(7)	118.0(3)	C(41)-C(42)-H(42)	120.0
C(43)-C(42)-H(42)	120.0	C(43)-C(42)-C(41)	120.1(4)
C(12)-C(13)-H(13)	119.5	C(14)-C(13)-C(12)	120.9(4)
C(14)-C(13)-H(13)	119.5	C(15)-C(14)-C(13)	119.8(4)
C(15)-C(14)-H(14)	120.1	C(13)-C(14)-H(14)	120.1
C(23)-C(24)-H(24)	119.4	C(25)-C(24)-C(23)	121.2(5)
C(25)-C(24)-H(24)	119.4	C(46)-C(41)-C(30)	122.8(4)
C(42)-C(41)-C(46)	119.2(4)	C(42)-C(41)-C(30)	118.0(4)
C(35)-C(36)-H(36)	119.9	C(37)-C(36)-C(35)	120.1(4)
C(37)-C(36)-H(36)	119.9	C(29)-C(30)-C(41)	121.5(3)
C(31)-C(30)-C(29)	119.7(4)	C(31)-C(30)-C(41)	118.7(4)
C(17)-C(18)-H(18)	119.7	C(19)-C(18)-C(17)	120.6(4)
C(19)-C(18)-H(18)	119.7	C(34)-C(33)-H(33)	119.1
C(32)-C(33)-C(34)	121.8(4)	C(32)-C(33)-H(33)	119.1
C(30)-C(31)-H(31)	119.8	C(32)-C(31)-C(30)	120.4(4)
C(32)-C(31)-H(31)	119.8	C(35)-C(40)-H(40)	119.6
C(39)-C(40)-C(35)	120.7(4)	C(39)-C(40)-H(40)	119.6
C(23)-C(28)-H(28)	119.7	C(23)-C(28)-C(27)	120.6(4)
C(27)-C(28)-H(28)	119.7	C(33)-C(32)-C(31)	119.8(4)
C(33)-C(32)-H(32)	120.1	C(31)-C(32)-H(32)	120.1
C(46)-C(45)-H(45)	119.9	C(46)-C(45)-C(44)	120.2(4)
C(44)-C(45)-H(45)	119.9	N(3)-C(47)-C(52)	115.4(3)
N(3)-C(47)-H(47)	107.5	N(3)-C(47)-C(48)	109.7(4)
C(52)-C(47)-H(47)	107.5	C(48)-C(47)-C(52)	109.0(4)
C(48)-C(47)-H(47)	107.5	H(50A)-C(50)-H(50B)	108.0
C(51)-C(50)-H(50A)	109.5	C(51)-C(50)-H(50B)	109.5
C(49)-C(50)-H(50A)	109.5	C(49)-C(50)-H(50B)	109.5

C(49)-C(50)-C(51)	110.9(4)	C(28)-C(27)-H(27)	119.9
C(26)-C(27)-C(28)	120.2(5)	C(26)-C(27)-H(27)	119.9
C(52)-C(51)-H(51A)	109.5	C(52)-C(51)-H(51B)	109.5
C(50)-C(51)-C(52)	110.6(4)	C(50)-C(51)-H(51A)	109.5
C(50)-C(51)-H(51B)	109.5	H(51A)-C(51)-H(51B)	108.1
C(18)-C(19)-H(19)	119.4	C(18)-C(19)-C(20)	121.1(4)
C(20)-C(19)-H(19)	119.4	C(22)-C(21)-H(21)	120.0
C(20)-C(21)-C(22)	120.0(4)	C(20)-C(21)-H(21)	120.0
C(47)-C(48)-H(48A)	109.2	C(47)-C(48)-H(48B)	109.2
H(48A)-C(48)-H(48B)	107.9	C(49)-C(48)-C(47)	112.0(4)
C(49)-C(48)-H(48A)	109.2	C(49)-C(48)-H(48B)	109.2
C(24)-C(25)-H(25)	119.9	C(26)-C(25)-C(24)	120.1(5)
C(26)-C(25)-H(25)	119.9	C(19)-C(20)-H(20)	120.4
C(21)-C(20)-C(19)	119.2(4)	C(21)-C(20)-H(20)	120.4
C(42)-C(43)-H(43)	119.6	C(44)-C(43)-C(42)	120.7(4)
C(44)-C(43)-H(43)	119.6	C(45)-C(44)-H(44)	120.3
C(43)-C(44)-C(45)	119.4(4)	C(43)-C(44)-H(44)	120.3
C(27)-C(26)-H(26)	120.0	C(25)-C(26)-C(27)	119.9(4)
C(25)-C(26)-H(26)	120.0	C(36)-C(37)-H(37)	119.8
C(36)-C(37)-C(38)	120.4(5)	C(38)-C(37)-H(37)	119.8
C(40)-C(39)-H(39)	120.0	C(38)-C(39)-C(40)	120.0(5)
C(38)-C(39)-H(39)	120.0	C(37)-C(38)-H(38)	119.9
C(39)-C(38)-C(37)	120.2(5)	C(39)-C(38)-H(38)	119.9
C(56)-O(1)-C(53)	106.5(7)	C(50)-C(49)-C(48)	112.0(4)
C(50)-C(49)-H(49A)	109.2	C(50)-C(49)-H(49B)	109.2
C(48)-C(49)-H(49A)	109.2	C(48)-C(49)-H(49B)	109.2
H(49A)-C(49)-H(49B)	107.9	O(1)-C(56)-H(56A)	109.7
O(1)-C(56)-H(56B)	109.7	O(1)-C(56)-C(55)	109.6(8)
H(56A)-C(56)-H(56B)	108.2	C(55)-C(56)-H(56A)	109.7
C(55)-C(56)-H(56B)	109.7	H(54A)-C(54)-H(54B)	109.0
C(53)-C(54)-H(54A)	111.0	C(53)-C(54)-H(54B)	111.0
C(53)-C(54)-C(55)	104.0(9)	C(55)-C(54)-H(54A)	111.0
C(55)-C(54)-H(54B)	111.0	O(1)-C(53)-C(54)	105.9(9)
O(1)-C(53)-H(53A)	110.6	O(1)-C(53)-H(53B)	110.6
C(54)-C(53)-H(53A)	110.6	C(54)-C(53)-H(53B)	110.6

H(53A)-C(53)-H(53B)	108.7	C(56)-C(55)-C(54)	104.0(8)
C(56)-C(55)-H(55A)	111.0	C(56)-C(55)-H(55B)	111.0
C(54)-C(55)-H(55A)	111.0	C(54)-C(55)-H(55B)	111.0
H(55A)-C(55)-H(55B)	109.0	N(1)-Bi(1B)-N(2)	139.6(2)
N(1)-Bi(1B)-C(47)	131.7(2)	C(1)-Bi(1B)-N(1)	74.94(15)
C(1)-Bi(1B)-N(2)	66.76(14)	C(1)-Bi(1B)-C(47)	144.8(2)
C(47)-Bi(1B)-N(2)	88.41(14)		

8.4 Single crystal structure analysis of **17**

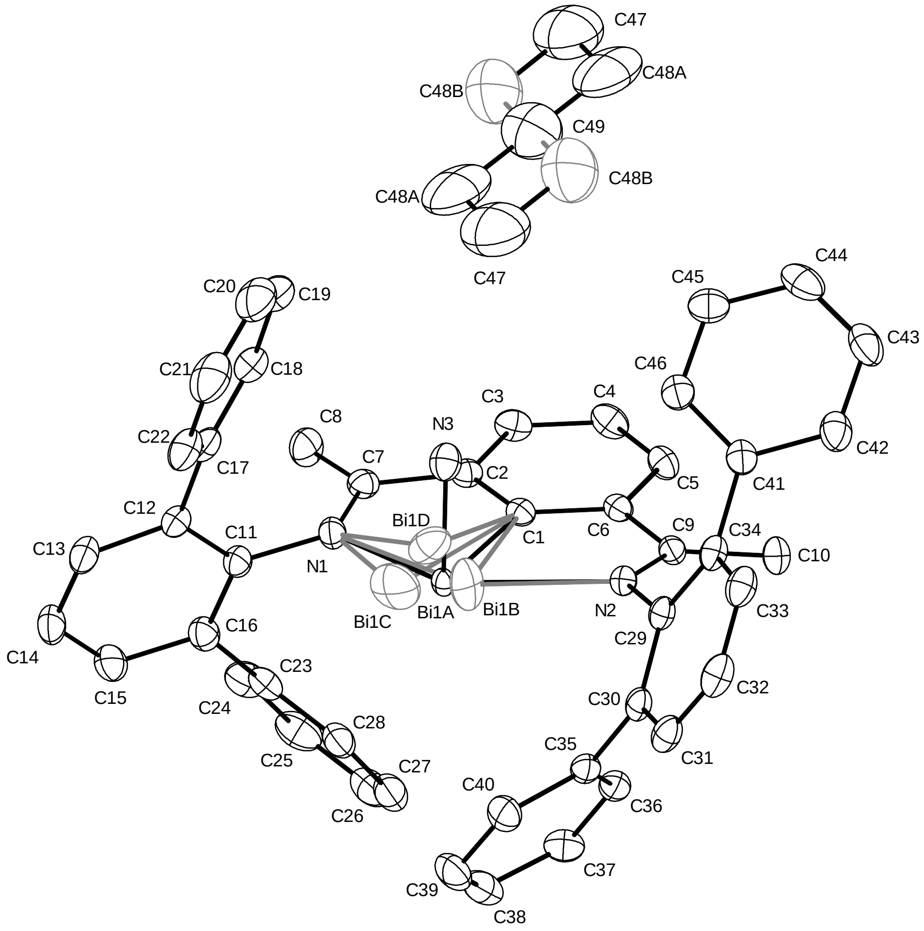

Fig. S37. The molecular structure of complex **17**. H atoms have been removed for clarity.

X-ray Crystal Structure Analysis of complex 17: C_{97} H_{82} Bi_2 N_6, M_r = 1749.64 g mol^{-1}, orange prism, crystal size 0.046 x 0.042 x 0.031 mm^3, triclinic, space group *P*-1 [2], a = 12.0598(7) Å, b = 12.3802(7) Å, c = 14.5534(9) Å, α = 101.113(2)°, β = 94.466(2)°, γ = 114.201(2)°, V = 1914.6(2) Å^3, T = 100(2) K, Z = 1, D_{calc} = 1.517 g·cm^3, λ = 0.71073 Å, $\mu(Mo\text{-}K_\alpha)$ = 4.642 mm^{-1}, analytical absorption correction ($T_{\min}$ = 0.87009, $T_{\max}$ = 0.90794), Bruker-AXS Kappa Mach3 with APEX-II detector and IµS micro focuse source, 1.861 < θ < 29.130°, 93756 measured reflections, 10286 independent reflections, 9664 reflections with $I > 2\sigma(I)$, R_{int} = 0.0338. The structure was solved by *SHELXT* and refined by full-matrix least-squares (*SHELXL*) against F^2 to R_1 = 0.0167 [$I > 2\sigma(I)$], wR_2 = 0.0385, 529 parameters and 24 restraints.

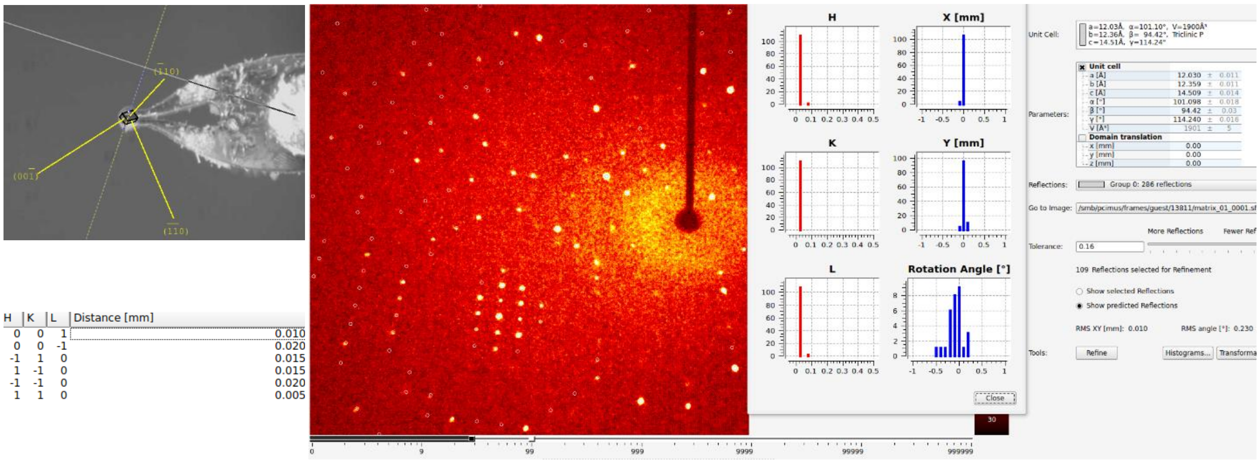

Fig. S38. Crystall faces and unit cell determination/refinement of **17**.

INTENSITY STATISTICS FOR DATASET

Resolution	#Data	#Theory	%Complete	Redundancy	Mean I	Mean I/s	Rmerge	Rsigma
Inf - 2.56	240	240	100.0	13.09	141.84	119.01	0.0165	0.0074
2.56 - 1.71	556	556	100.0	12.03	85.76	100.59	0.0192	0.0083
1.71 - 1.36	801	801	100.0	11.21	58.97	86.13	0.0221	0.0096
1.36 - 1.18	849	849	100.0	10.94	42.87	72.73	0.0276	0.0112
1.18 - 1.08	749	749	100.0	10.36	36.10	61.67	0.0314	0.0130
1.08 - 1.00	815	815	100.0	9.85	29.07	52.64	0.0377	0.0156
1.00 - 0.94	819	819	100.0	9.30	26.61	46.38	0.0430	0.0177
0.94 - 0.89	857	857	100.0	8.79	21.65	38.61	0.0484	0.0216
0.89 - 0.85	837	837	100.0	8.41	18.64	32.81	0.0572	0.0255
0.85 - 0.82	746	746	100.0	8.06	16.53	28.98	0.0642	0.0295
0.82 - 0.79	862	862	100.0	7.69	14.95	25.49	0.0717	0.0338
0.79 - 0.77	656	656	100.0	7.39	14.19	22.95	0.0765	0.0371
0.77 - 0.74	1115	1115	100.0	7.05	12.37	19.78	0.0870	0.0437
0.74 - 0.72	854	854	100.0	6.11	12.04	17.22	0.0918	0.0507
0.72 - 0.71	449	449	100.0	5.71	10.61	14.78	0.1016	0.0591
0.71 - 0.69	1001	1001	100.0	5.31	9.56	12.97	0.1072	0.0685
0.69 - 0.68	562	562	100.0	5.04	9.50	12.38	0.1139	0.0729
0.68 - 0.66	1178	1181	99.7	4.90	8.42	10.77	0.1275	0.0835
0.66 - 0.65	641	644	99.5	4.65	7.47	9.49	0.1379	0.0964
0.65 - 0.64	713	717	99.4	4.60	7.37	9.11	0.1432	0.1003
0.64 - 0.63	589	614	95.9	4.19	6.24	7.41	0.1617	0.1244
0.73 - 0.63	5591	5626	99.4	4.99	8.69	11.38	0.1195	0.0799
Inf - 0.63	15889	15924	99.8	7.66	23.53	34.90	0.0392	0.0248

During initial refinement cycles, a high residual electron density close to the Bi central atom was observed which might be the result of anharmonic atomic displacement of the Bi atom, possibly caused by loss or

disorder of solvent in the crystal. The Bi central atom was split into four parts in order to describe the local electron density around the heavy atom. We cannot rule out that the crystal is contaminated. For example, a 10% impurity would show up as a residual electron density of ca. 8 electrons from Bi (83 electrons) but reveal little of the lighter atoms in the structure. We note, however, that when the electron density around the Bi atom is satisfactorily described using the split-atom model, it is possible to see the locations of all the hydrogen atoms via a difference Fourier map. When all atoms are included in the model, the residual density reduces to an acceptable level and the R_1 value is excellent. In order to avoid high correlations, the atomic displacement parameters of the Bi components were retrained to be equal with an effective standard deviation of 0.001. In addition, the structure contains a disordered pentane molecule on a crystallographic special position (inversion centre). The positions of the H atoms are consistent with the characteristic NMR coupling signals.

Complete .cif-data of the compound are available under the CCDC number **CCDC-2128289**.

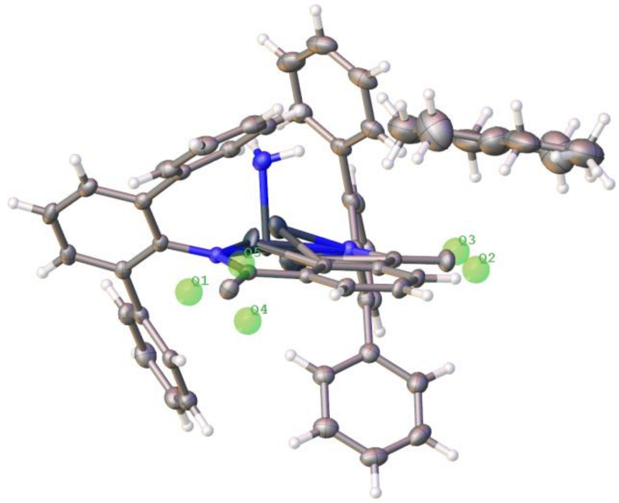

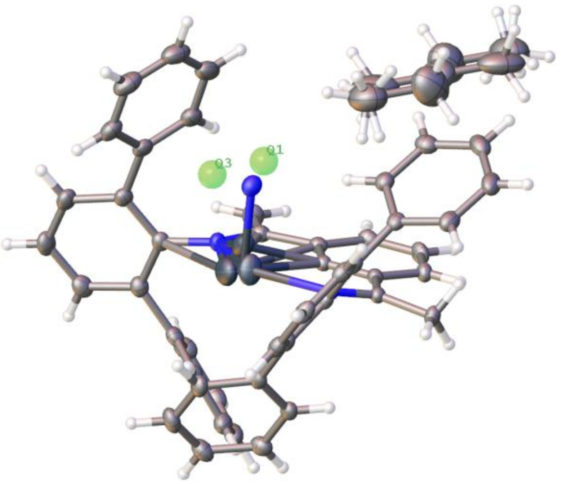

Fig. S39. Likely hydrogen atom positions attached to N3, C8 and C10 could be observed as residual electron density peaks (green) after refinement with the multiple Bi atom component model.

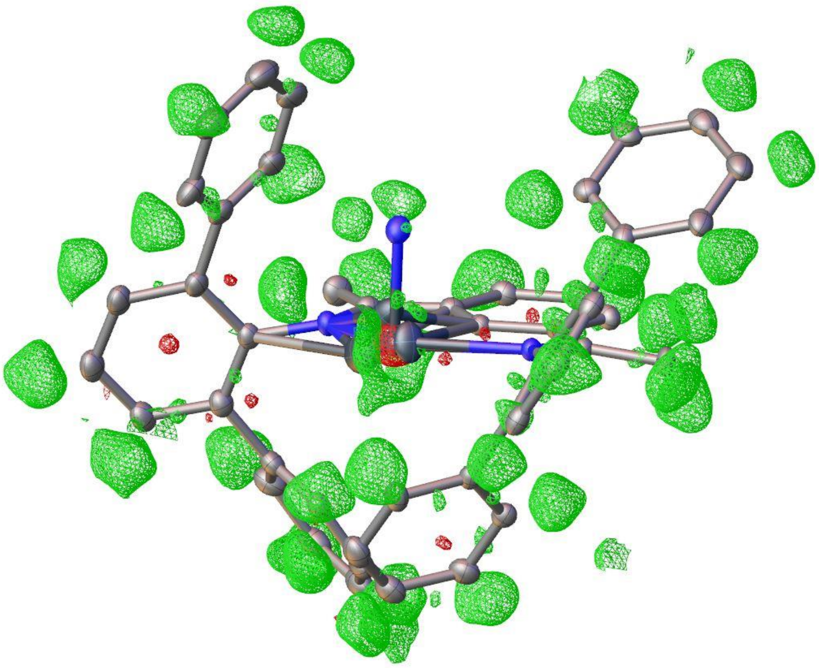

Fig. S40. Residual electron density map (Olex 2.0, diff. map. peak: min. -1.193 e/Å^3, max. 1.122 e/Å^3, surface level 0.3 e/Å^3) based on the phases from the non-hydrogen atoms using the multiple Bi atom component model, revealing the positions of all the hydrogen atoms.

Table S13. Crystal data and structure refinement of **17**.

Identification code	13811	
Empirical formula	$C_{97} H_{82} Bi_2 N_6$	
Color	orange	
Formula weight	1749.64 g·mol^{-1}	
Temperature	100(2) K	
Wavelength	0.71073 Å	
Crystal system	Triclinic	
Space group	*P*-1, (no. 2)	
Unit cell dimensions	a = 12.0598(7) Å	α= 101.113(2)°.
	b = 12.3802(7) Å	β= 94.466(2)°.
	c = 14.5534(9) Å	γ = 114.201(2)°.
Volume	1914.6(2) Å^3	
Z	1	
Density (calculated)	1.517 Mg·m^{-3}	
Absorption coefficient	4.642 mm^{-1}	
F(000)	872 e	
Crystal size	0.046 x 0.042 x 0.031 mm^3	
θ range for data collection	1.861 to 29.130°.	
Index ranges	-16 ≤ h ≤ 16, -16≤ k ≤ 16, -19 ≤ l ≤ 19	
Reflections collected	93756	

Independent reflections	10286 [R_{int} = 0.0338]	
Reflections with I>2σ(I)	9664	
Completeness to θ = 25.242°	99.8 %	
Absorption correction	Gaussian	
Max. and min. transmission	0.90794 and 0.87009	
Refinement method	Full-matrix least-squares on F^2	
Data / restraints / parameters	10286 / 24 / 529	
Goodness-of-fit on F^2	1.070	
Final R indices [I>2σ(I)]	R_1 = 0.0167	wR^2 = 0.0385
R indices (all data)	R_1 = 0.0193	wR^2 = 0.0393
Extinction coefficient	n/a	
Largest diff. peak and hole	0.595 and -0.702 e·Å^{-3}	

Table S14. Bond lengths [Å] and angles [°] of **17**.

C(1)-C(2)	1.393(2)	C(1)-C(6)	1.396(2)
C(1)-Bi(1A)	2.1932(16)	C(1)-Bi(1C)	2.313(4)
C(1)-Bi(1B)	2.339(5)	C(1)-Bi(1D)	2.196(4)
C(2)-C(3)	1.410(2)	C(2)-C(7)	1.491(2)
C(2)-Bi(1C)	2.774(4)	C(3)-H(3)	0.9500
C(3)-C(4)	1.381(3)	C(4)-H(4)	0.9500
C(4)-C(5)	1.392(3)	C(5)-H(5)	0.9500
C(5)-C(6)	1.398(2)	C(6)-C(9)	1.486(2)
C(7)-C(8)	1.357(2)	C(7)-N(1)	1.381(2)
C(7)-Bi(1C)	2.483(5)	C(8)-H(8A)	0.97(3)
C(8)-H(8B)	0.95(2)	C(9)-C(10)	1.497(2)
C(9)-N(2)	1.290(2)	C(10)-H(10A)	0.9800
C(10)-H(10B)	0.9800	C(10)-H(10C)	0.9800
C(11)-C(12)	1.414(3)	C(11)-C(16)	1.417(3)
C(11)-N(1)	1.417(2)	C(11)-Bi(1C)	2.410(6)
C(12)-C(13)	1.403(3)	C(12)-C(17)	1.490(3)
C(13)-H(13)	0.9500	C(13)-C(14)	1.381(3)
C(14)-H(14)	0.9500	C(14)-C(15)	1.384(3)
C(15)-H(15)	0.9500	C(15)-C(16)	1.401(3)
C(16)-C(23)	1.495(3)	C(17)-C(18)	1.398(3)
C(17)-C(22)	1.395(3)	C(18)-H(18)	0.9500
C(18)-C(19)	1.391(3)	C(19)-H(19)	0.9500
C(19)-C(20)	1.385(4)	C(20)-H(20)	0.9500
C(20)-C(21)	1.374(4)	C(21)-H(21)	0.9500
C(21)-C(22)	1.392(3)	C(22)-H(22)	0.9500
C(23)-C(24)	1.392(3)	C(23)-C(28)	1.395(3)
C(24)-H(24)	0.9500	C(24)-C(25)	1.394(3)
C(25)-H(25)	0.9500	C(25)-C(26)	1.376(3)
C(26)-H(26)	0.9500	C(26)-C(27)	1.385(3)
C(27)-H(27)	0.9500	C(27)-C(28)	1.391(3)
C(28)-H(28)	0.9500	C(29)-C(30)	1.409(2)
C(29)-C(34)	1.415(2)	C(29)-N(2)	1.417(2)

C(30)-C(31)	1.396(2)	C(30)-C(35)	1.492(2)
C(31)-H(31)	0.9500	C(31)-C(32)	1.384(3)
C(32)-H(32)	0.9500	C(32)-C(33)	1.384(3)
C(33)-H(33)	0.9500	C(33)-C(34)	1.398(2)
C(34)-C(41)	1.490(3)	C(35)-C(36)	1.395(2)
C(35)-C(40)	1.403(2)	C(36)-H(36)	0.9500
C(36)-C(37)	1.389(3)	C(37)-H(37)	0.9500
C(37)-C(38)	1.391(3)	C(38)-H(38)	0.9500
C(38)-C(39)	1.382(3)	C(39)-H(39)	0.9500
C(39)-C(40)	1.389(3)	C(40)-H(40)	0.9500
C(41)-C(42)	1.401(3)	C(41)-C(46)	1.399(3)
C(42)-H(42)	0.9500	C(42)-C(43)	1.387(3)
C(43)-H(43)	0.9500	C(43)-C(44)	1.384(3)
C(44)-H(44)	0.9500	C(44)-C(45)	1.389(3)
C(45)-H(45)	0.9500	C(45)-C(46)	1.390(3)
C(46)-H(46)	0.9500	Bi(1A)-N(1)	2.2616(14)
Bi(1A)-N(2)	2.6326(14)	Bi(1A)-N(3)	2.1775(16)
N(1)-Bi(1C)	1.452(5)	N(1)-Bi(1B)	2.740(4)
N(1)-Bi(1D)	2.096(5)	N(2)-Bi(1B)	2.247(5)
N(3)-H(3A)	0.94(3)	N(3)-H(3B)	0.98(5)
C(49)-C(48A)	1.537(10)	C(49)-C(48A)#1	1.537(10)
C(49)-C(48B)#1	1.529(9)	C(49)-C(48B)	1.529(9)
C(47)-H(47A)	0.9800	C(47)-H(47B)	0.9800
C(47)-H(47C)	0.9800	C(47)-H(47D)	0.9800
C(47)-H(47E)	0.9800	C(47)-H(47F)	0.9800
C(47)-C(48A)	1.554(9)	C(47)-C(48B)	1.539(9)
C(48A)-H(48A)	0.9900	C(48A)-H(48B)	0.9900
C(48B)-H(48C)	0.9900	C(48B)-H(48D)	0.9900

C(2)-C(1)-C(6)	122.37(15)	C(2)-C(1)-Bi(1A)	115.50(12)
C(2)-C(1)-Bi(1C)	93.61(17)	C(2)-C(1)-Bi(1B)	126.85(16)
C(2)-C(1)-Bi(1D)	108.60(17)	C(6)-C(1)-Bi(1A)	122.13(12)
C(6)-C(1)-Bi(1C)	143.05(17)	C(6)-C(1)-Bi(1B)	110.70(16)

C(6)-C(1)-Bi(1D)	127.38(17)	C(1)-C(2)-C(3)	117.69(16)
C(1)-C(2)-C(7)	119.03(15)	C(1)-C(2)-Bi(1C)	56.32(14)
C(3)-C(2)-C(7)	123.28(15)	C(3)-C(2)-Bi(1C)	171.55(15)
C(7)-C(2)-Bi(1C)	63.02(14)	C(2)-C(3)-H(3)	119.9
C(4)-C(3)-C(2)	120.24(16)	C(4)-C(3)-H(3)	119.9
C(3)-C(4)-H(4)	119.3	C(3)-C(4)-C(5)	121.47(16)
C(5)-C(4)-H(4)	119.3	C(4)-C(5)-H(5)	120.4
C(4)-C(5)-C(6)	119.25(16)	C(6)-C(5)-H(5)	120.4
C(1)-C(6)-C(5)	118.93(15)	C(1)-C(6)-C(9)	119.76(15)
C(5)-C(6)-C(9)	121.30(15)	C(2)-C(7)-Bi(1C)	84.64(14)
C(8)-C(7)-C(2)	122.03(17)	C(8)-C(7)-N(1)	124.43(17)
C(8)-C(7)-Bi(1C)	152.75(18)	N(1)-C(7)-C(2)	113.54(14)
N(1)-C(7)-Bi(1C)	29.55(12)	C(7)-C(8)-H(8A)	119.2(15)
C(7)-C(8)-H(8B)	123.0(14)	H(8A)-C(8)-H(8B)	118(2)
C(6)-C(9)-C(10)	119.36(15)	N(2)-C(9)-C(6)	115.90(14)
N(2)-C(9)-C(10)	124.74(15)	C(9)-C(10)-H(10A)	109.5
C(9)-C(10)-H(10B)	109.5	C(9)-C(10)-H(10C)	109.5
H(10A)-C(10)-H(10B)	109.5	H(10A)-C(10)-H(10C)	109.5
H(10B)-C(10)-H(10C)	109.5	C(12)-C(11)-C(16)	118.90(16)
C(12)-C(11)-N(1)	120.85(16)	C(12)-C(11)-Bi(1C)	115.21(15)
C(16)-C(11)-Bi(1C)	113.54(15)	N(1)-C(11)-C(16)	120.19(16)
N(1)-C(11)-Bi(1C)	33.30(10)	C(11)-C(12)-C(17)	122.18(16)
C(13)-C(12)-C(11)	119.60(17)	C(13)-C(12)-C(17)	118.22(17)
C(12)-C(13)-H(13)	119.4	C(14)-C(13)-C(12)	121.17(18)
C(14)-C(13)-H(13)	119.4	C(13)-C(14)-H(14)	120.2
C(13)-C(14)-C(15)	119.57(18)	C(15)-C(14)-H(14)	120.2
C(14)-C(15)-H(15)	119.4	C(14)-C(15)-C(16)	121.22(18)
C(16)-C(15)-H(15)	119.4	C(11)-C(16)-C(23)	122.49(16)
C(15)-C(16)-C(11)	119.47(18)	C(15)-C(16)-C(23)	117.99(17)
C(18)-C(17)-C(12)	121.72(18)	C(22)-C(17)-C(12)	119.88(18)
C(22)-C(17)-C(18)	118.36(19)	C(17)-C(18)-H(18)	120.0
C(19)-C(18)-C(17)	120.0(2)	C(19)-C(18)-H(18)	120.0
C(18)-C(19)-H(19)	119.4	C(20)-C(19)-C(18)	121.1(2)
C(20)-C(19)-H(19)	119.4	C(19)-C(20)-H(20)	120.4
C(21)-C(20)-C(19)	119.2(2)	C(21)-C(20)-H(20)	120.4

C(20)-C(21)-H(21)	119.8	C(20)-C(21)-C(22)	120.5(2)
C(22)-C(21)-H(21)	119.8	C(17)-C(22)-H(22)	119.6
C(21)-C(22)-C(17)	120.9(2)	C(21)-C(22)-H(22)	119.6
C(24)-C(23)-C(16)	120.10(18)	C(24)-C(23)-C(28)	118.77(19)
C(28)-C(23)-C(16)	120.97(17)	C(23)-C(24)-H(24)	119.8
C(23)-C(24)-C(25)	120.4(2)	C(25)-C(24)-H(24)	119.8
C(24)-C(25)-H(25)	119.8	C(26)-C(25)-C(24)	120.3(2)
C(26)-C(25)-H(25)	119.8	C(25)-C(26)-H(26)	120.1
C(25)-C(26)-C(27)	119.8(2)	C(27)-C(26)-H(26)	120.1
C(26)-C(27)-H(27)	119.9	C(26)-C(27)-C(28)	120.2(2)
C(28)-C(27)-H(27)	119.9	C(23)-C(28)-H(28)	119.8
C(27)-C(28)-C(23)	120.46(19)	C(27)-C(28)-H(28)	119.8
C(30)-C(29)-C(34)	120.33(15)	C(30)-C(29)-N(2)	118.04(15)
C(34)-C(29)-N(2)	121.25(15)	C(29)-C(30)-C(35)	120.61(15)
C(31)-C(30)-C(29)	119.07(16)	C(31)-C(30)-C(35)	120.31(16)
C(30)-C(31)-H(31)	119.5	C(32)-C(31)-C(30)	120.97(18)
C(32)-C(31)-H(31)	119.5	C(31)-C(32)-H(32)	120.1
C(31)-C(32)-C(33)	119.80(17)	C(33)-C(32)-H(32)	120.1
C(32)-C(33)-H(33)	119.3	C(32)-C(33)-C(34)	121.47(17)
C(34)-C(33)-H(33)	119.3	C(29)-C(34)-C(41)	122.27(15)
C(33)-C(34)-C(29)	118.34(17)	C(33)-C(34)-C(41)	119.36(16)
C(36)-C(35)-C(30)	121.39(15)	C(36)-C(35)-C(40)	118.33(17)
C(40)-C(35)-C(30)	120.28(16)	C(35)-C(36)-H(36)	119.7
C(37)-C(36)-C(35)	120.58(17)	C(37)-C(36)-H(36)	119.7
C(36)-C(37)-H(37)	119.9	C(36)-C(37)-C(38)	120.23(19)
C(38)-C(37)-H(37)	119.9	C(37)-C(38)-H(38)	120.0
C(39)-C(38)-C(37)	119.96(19)	C(39)-C(38)-H(38)	120.0
C(38)-C(39)-H(39)	120.1	C(38)-C(39)-C(40)	119.85(19)
C(40)-C(39)-H(39)	120.1	C(35)-C(40)-H(40)	119.5
C(39)-C(40)-C(35)	121.03(18)	C(39)-C(40)-H(40)	119.5
C(42)-C(41)-C(34)	118.76(17)	C(46)-C(41)-C(34)	122.56(16)
C(46)-C(41)-C(42)	118.67(18)	C(41)-C(42)-H(42)	119.8
C(43)-C(42)-C(41)	120.47(19)	C(43)-C(42)-H(42)	119.8
C(42)-C(43)-H(43)	119.7	C(44)-C(43)-C(42)	120.58(19)
C(44)-C(43)-H(43)	119.7	C(43)-C(44)-H(44)	120.3

C(43)-C(44)-C(45)	119.40(19)	C(45)-C(44)-H(44)	120.3
C(44)-C(45)-H(45)	119.7	C(44)-C(45)-C(46)	120.62(19)
C(46)-C(45)-H(45)	119.7	C(41)-C(46)-H(46)	119.9
C(45)-C(46)-C(41)	120.19(18)	C(45)-C(46)-H(46)	119.9
C(1)-Bi(1A)-N(1)	75.20(6)	C(1)-Bi(1A)-N(2)	68.69(5)
N(1)-Bi(1A)-N(2)	143.84(5)	N(3)-Bi(1A)-C(1)	94.42(6)
N(3)-Bi(1A)-N(1)	99.26(6)	N(3)-Bi(1A)-N(2)	81.97(5)
C(7)-N(1)-C(11)	119.55(14)	C(7)-N(1)-Bi(1A)	116.68(11)
C(7)-N(1)-Bi(1C)	122.48(18)	C(7)-N(1)-Bi(1B)	116.00(15)
C(7)-N(1)-Bi(1D)	115.04(16)	C(11)-N(1)-Bi(1A)	123.17(11)
C(11)-N(1)-Bi(1C)	114.30(18)	C(11)-N(1)-Bi(1B)	124.17(15)
C(11)-N(1)-Bi(1D)	124.97(16)	C(9)-N(2)-C(29)	124.36(14)
C(9)-N(2)-Bi(1A)	112.67(11)	C(9)-N(2)-Bi(1B)	119.61(16)
C(29)-N(2)-Bi(1A)	122.93(10)	C(29)-N(2)-Bi(1B)	116.03(16)
Bi(1A)-N(3)-H(3A)	103.7(19)	Bi(1A)-N(3)-H(3B)	106(3)
H(3A)-N(3)-H(3B)	104(3)	C(1)-Bi(1C)-C(2)	30.07(7)
C(1)-Bi(1C)-C(7)	62.30(10)	C(1)-Bi(1C)-C(11)	121.46(19)
C(7)-Bi(1C)-C(2)	32.34(7)	C(11)-Bi(1C)-C(2)	91.52(16)
C(11)-Bi(1C)-C(7)	59.19(13)	N(1)-Bi(1C)-C(1)	89.42(16)
N(1)-Bi(1C)-C(2)	59.97(13)	N(1)-Bi(1C)-C(7)	27.98(10)
N(1)-Bi(1C)-C(11)	32.40(13)	C(1)-Bi(1B)-N(1)	64.17(12)
N(2)-Bi(1B)-C(1)	73.57(17)	N(2)-Bi(1B)-N(1)	137.7(2)
C(48A)#1-C(49)-C(48A)	180.0	C(48B)-C(49)-C(48B)#1	180.0
H(47A)-C(47)-H(47B)	109.5	H(47A)-C(47)-H(47C)	109.5
H(47B)-C(47)-H(47C)	109.5	H(47D)-C(47)-H(47E)	109.5
H(47D)-C(47)-H(47F)	109.5	H(47E)-C(47)-H(47F)	109.5
C(48A)-C(47)-H(47A)	109.5	C(48A)-C(47)-H(47B)	109.5
C(48A)-C(47)-H(47C)	109.5	C(48B)-C(47)-H(47D)	109.5
C(48B)-C(47)-H(47E)	109.5	C(48B)-C(47)-H(47F)	109.5
C(49)-C(48A)-C(47)	109.8(5)	C(49)-C(48A)-H(48A)	109.7
C(49)-C(48A)-H(48B)	109.7	C(47)-C(48A)-H(48A)	109.7
C(47)-C(48A)-H(48B)	109.7	H(48A)-C(48A)-H(48B)	108.2
C(49)-C(48B)-C(47)	111.1(5)	C(49)-C(48B)-H(48C)	109.4
C(49)-C(48B)-H(48D)	109.4	C(47)-C(48B)-H(48C)	109.4
C(47)-C(48B)-H(48D)	109.4	H(48C)-C(48B)-H(48D)	108.0

N(1)-Bi(1D)-C(1)	78.56(15)

Symmetry transformations used to generate equivalent atoms:

#1 -x+1,-y+2,-z

9 NMR spectra of bismuth compounds

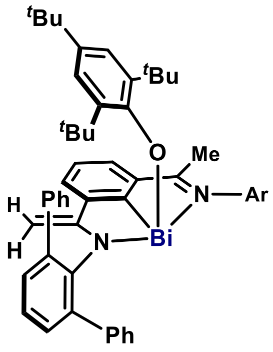

4, ^{1}H NMR in THF-d_8

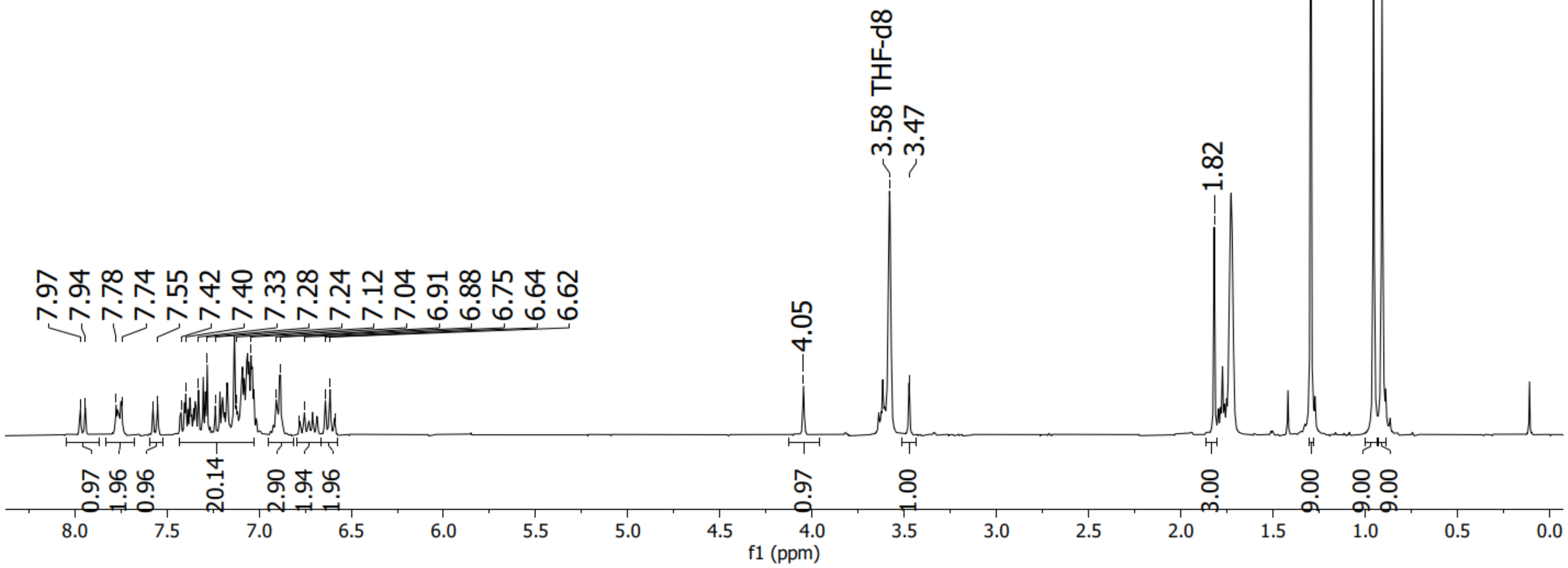

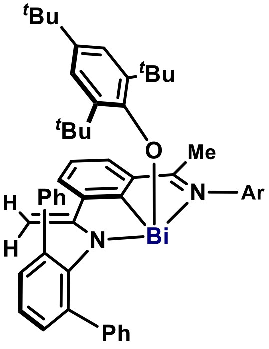

4, ^{13}C NMR in THF-d_8

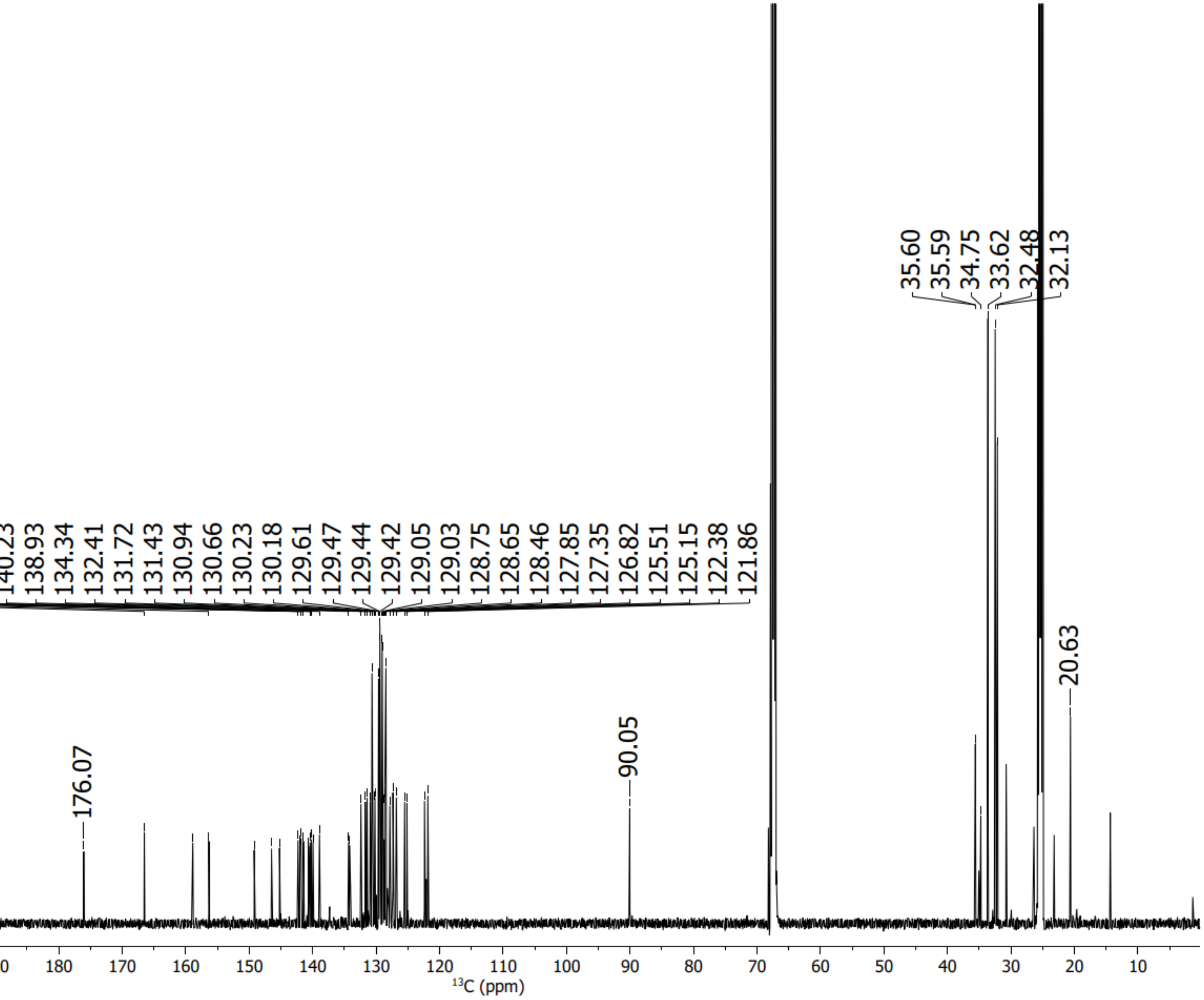

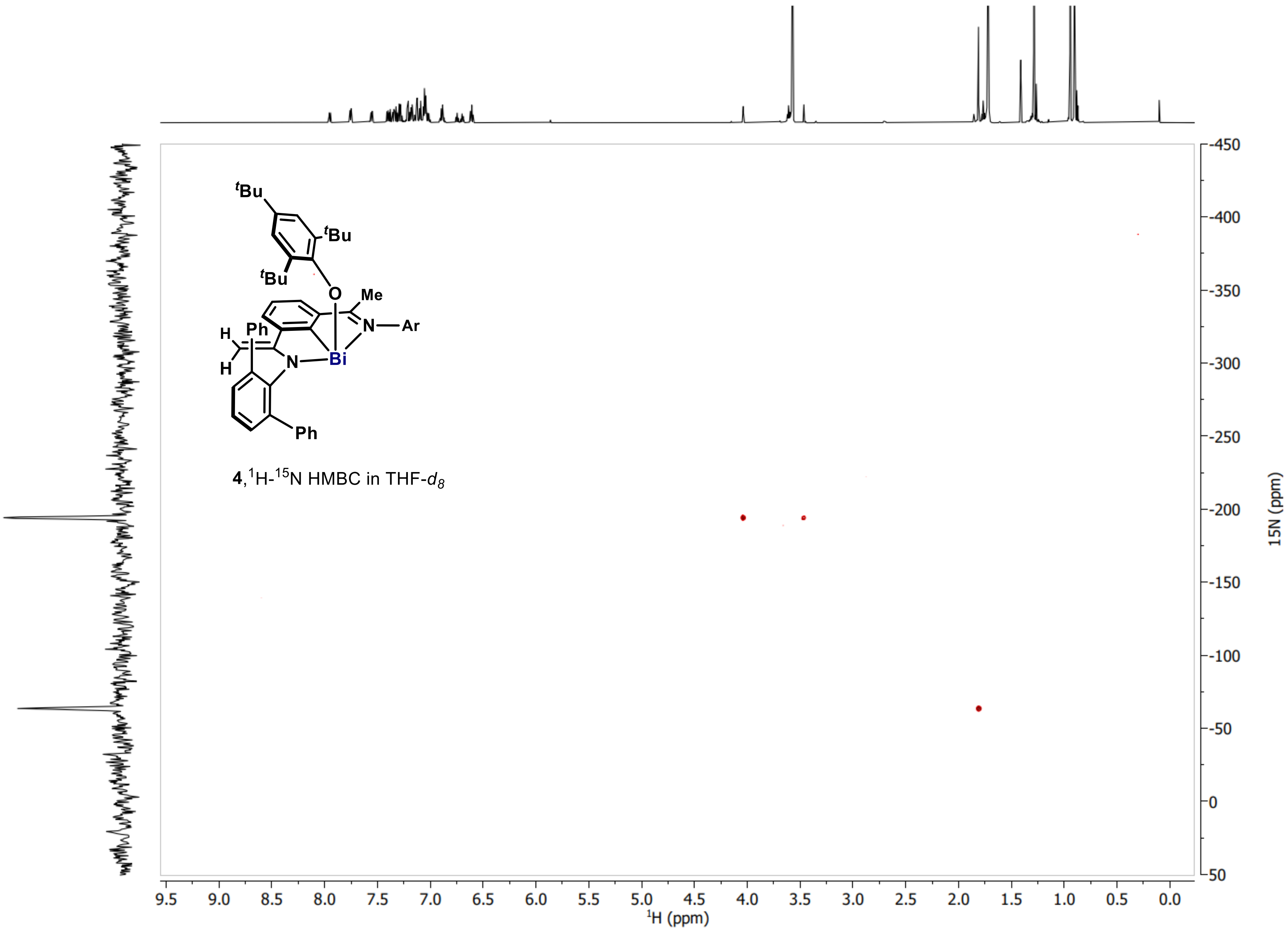

tBu
tBu
tBu
O
Me
N—Ar
H
Ph
N—Bi
H
Ph
4, 1H-15N HMBC in THF-d8
-450
-400
-350
-300
-250
-200
-150
-100
-50
0
50
15N (ppm)
9.5 9.0 8.5 8.0 7.5 7.0 6.5 6.0 5.5 5.0 4.5 4.0 3.5 3.0 2.5 2.0 1.5 1.0 0.5 0.0
1H (ppm)

F1 projection with ^{15}N Signals extracted from the ^{15}N-HMBC

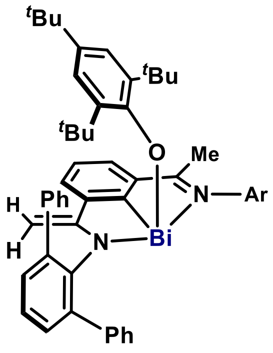

4, ^{15}N NMR in THF-d_8

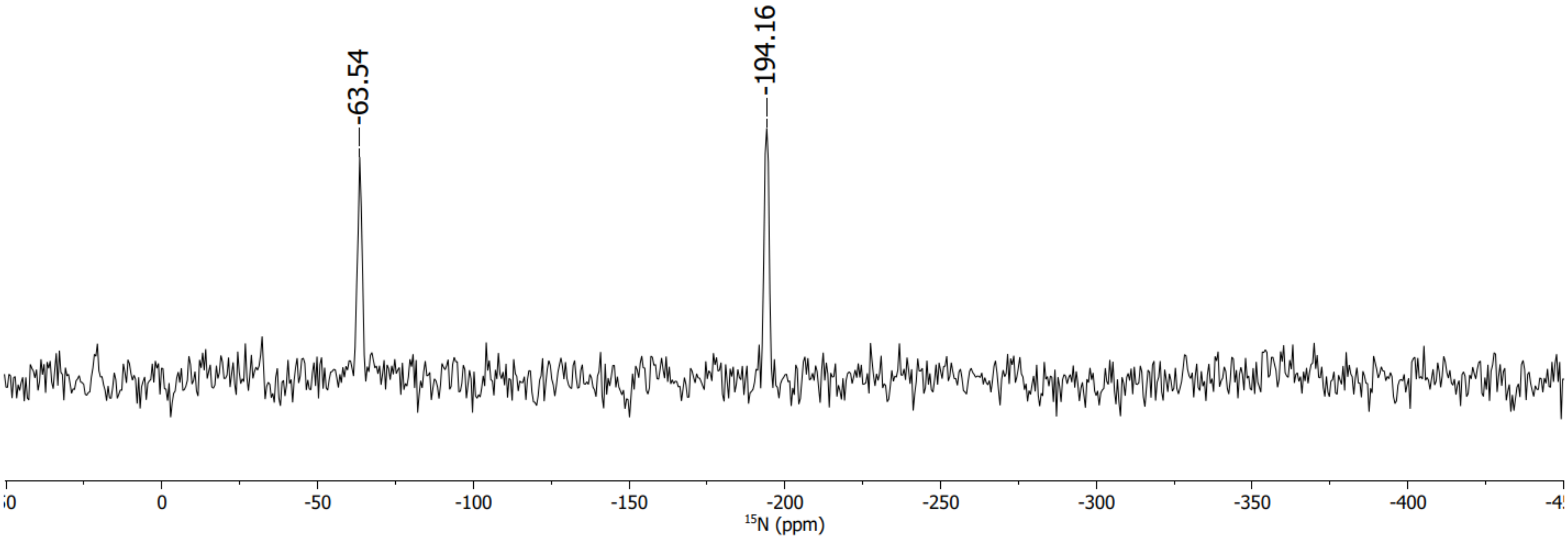

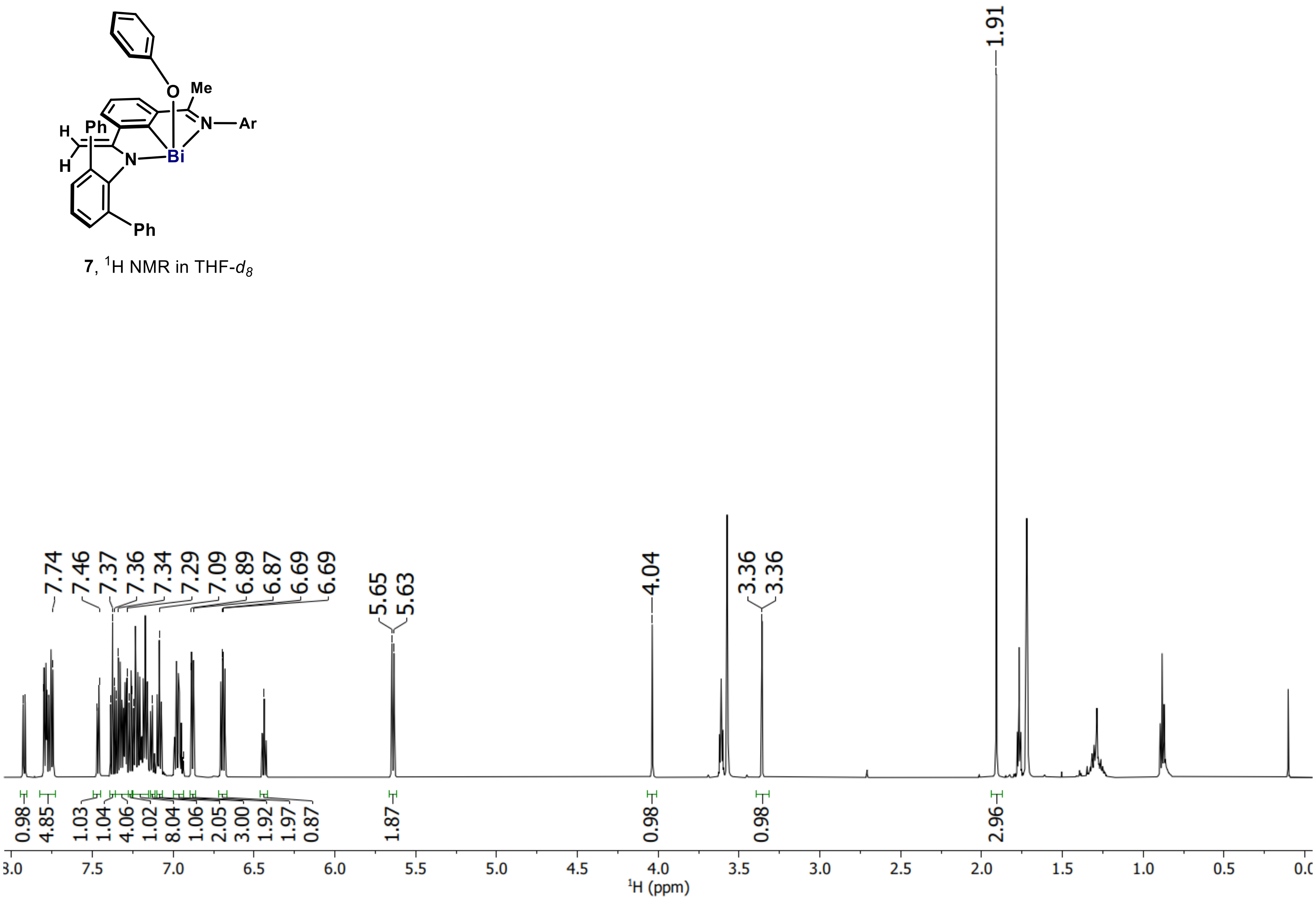

O
Me
N
Ar
Ph
H
H
N
Bi
Ph
7, 1H NMR in THF-d8
1.91
7.74
7.46
7.37
7.36
7.34
7.29
7.09
6.89
6.87
6.69
6.69
5.65
5.63
4.04
3.36
3.36
0.98
4.85
1.03
1.04
4.06
1.02
8.04
1.06
2.05
3.00
1.92
1.97
0.87
1.87
0.98
0.98
2.96
1H (ppm)

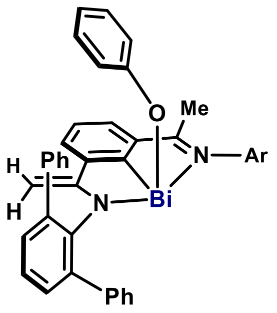

7, ^{13}C NMR in THF-d_8

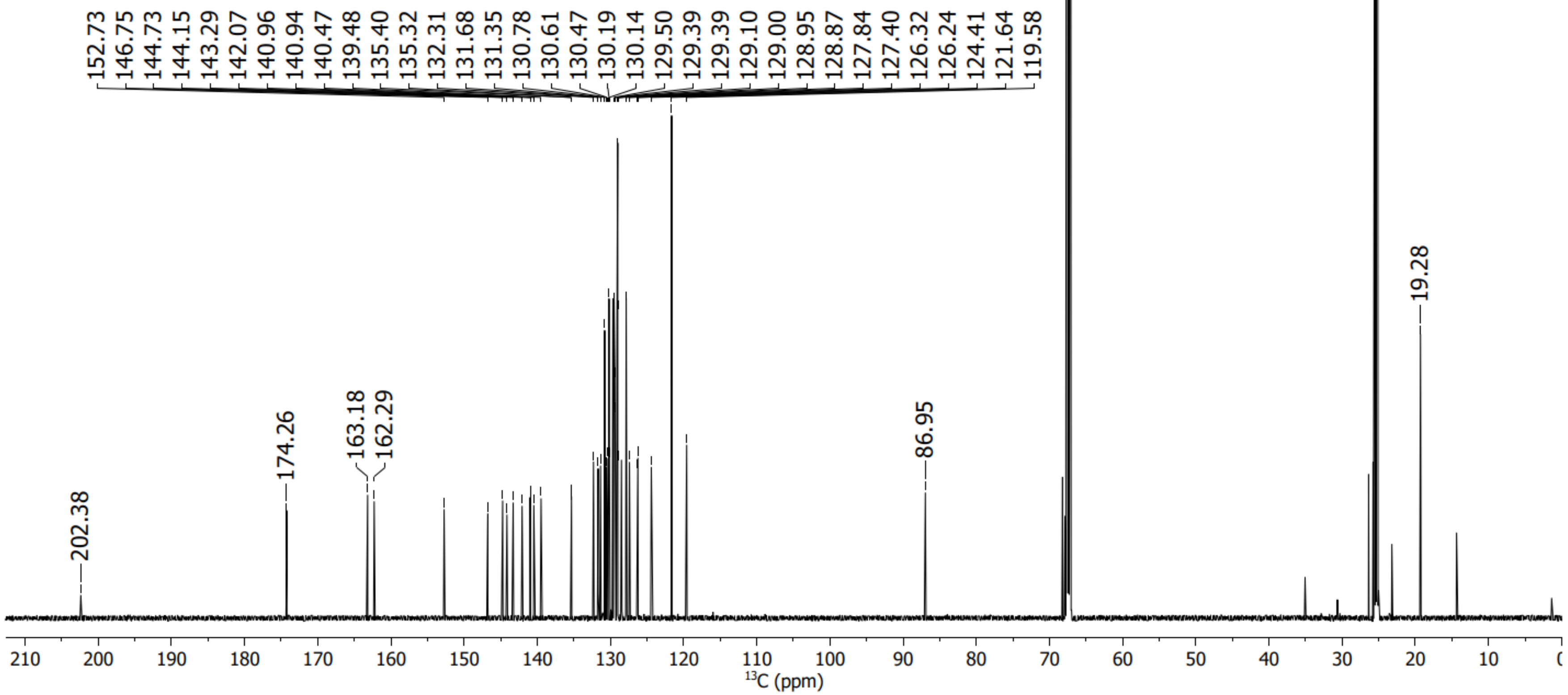

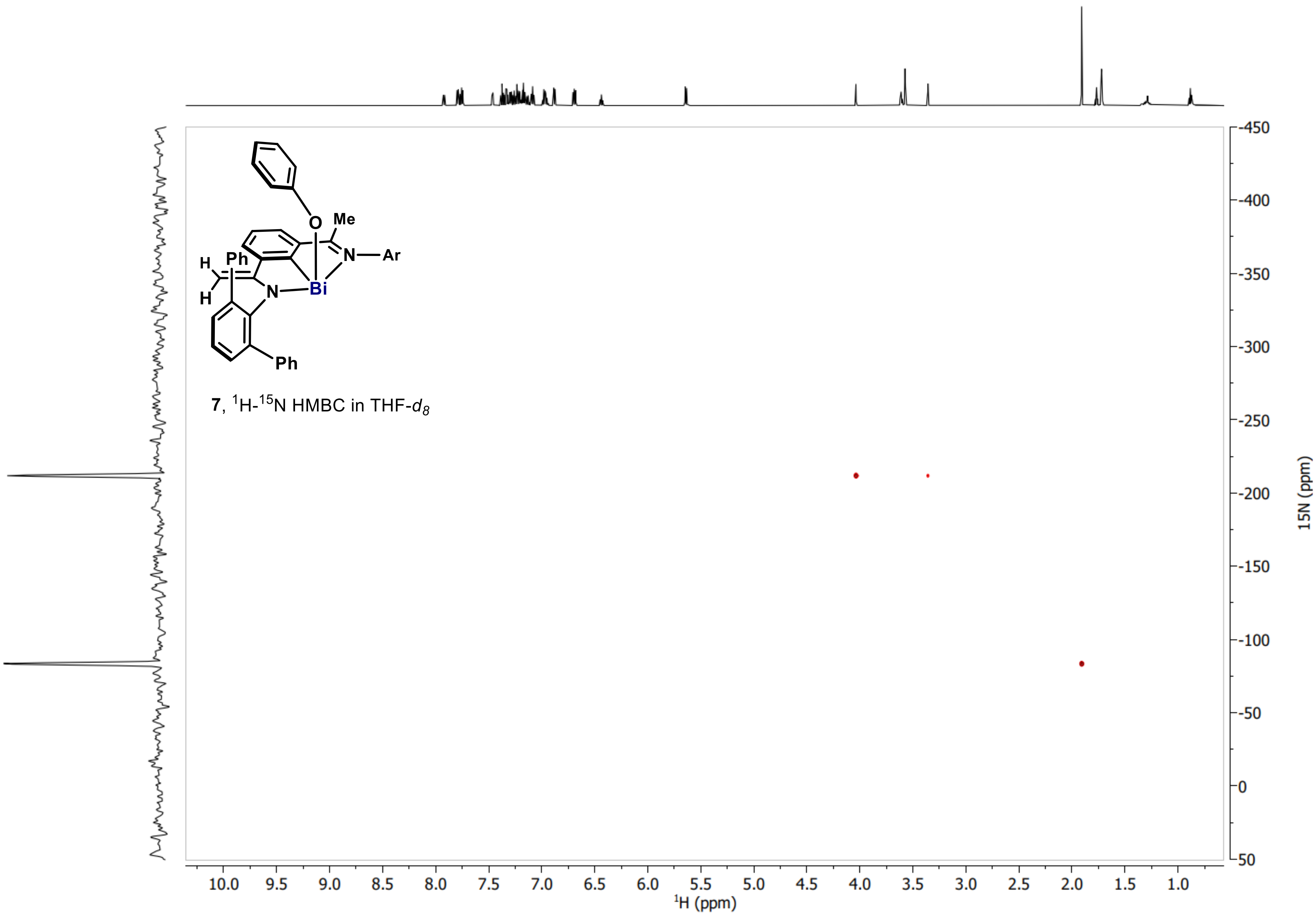

O
Me
N—Ar
Ph
H
H
N
Bi
Ph
7, ¹H-¹⁵N HMBC in THF-d8
-450
-400
-350
-300
-250
-200
-150
-100
-50
0
50
15N (ppm)
10.0
9.5
9.0
8.5
8.0
7.5
7.0
6.5
6.0
5.5
5.0
4.5
4.0
3.5
3.0
2.5
2.0
1.5
1.0
¹H (ppm)

F1 projection with ^{15}N Signals extracted from the ^{15}N-HMBC

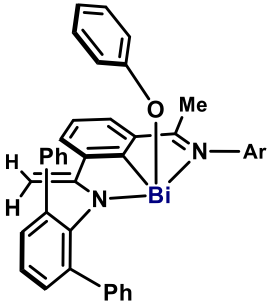

7, ^{15}N NMR in THF-d_8

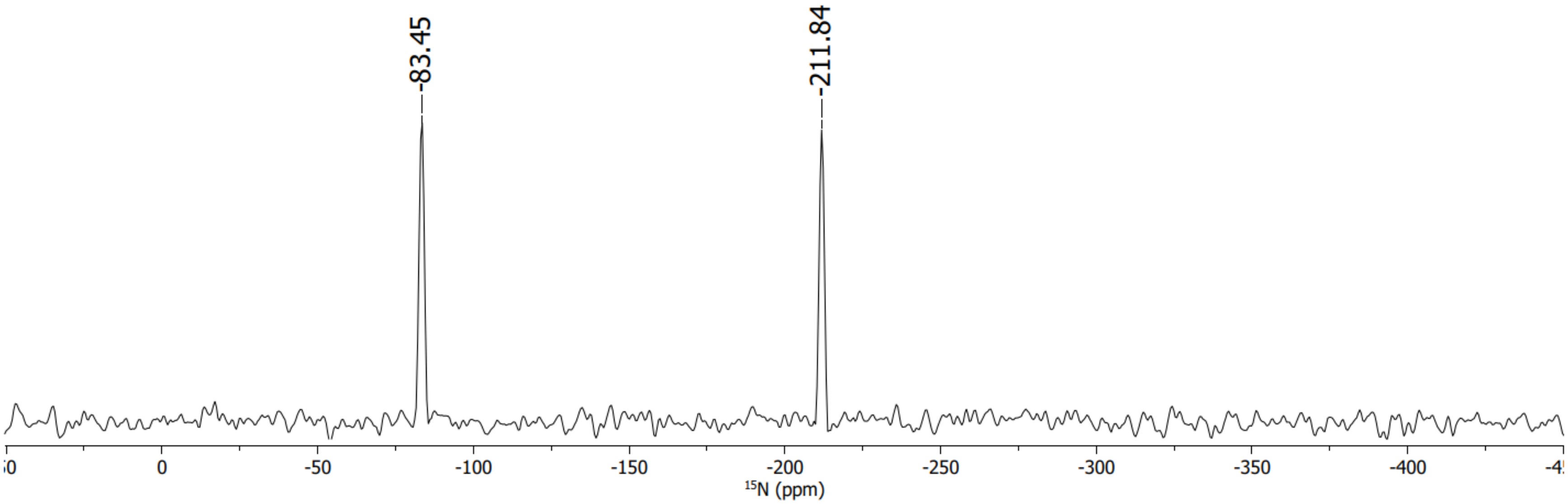

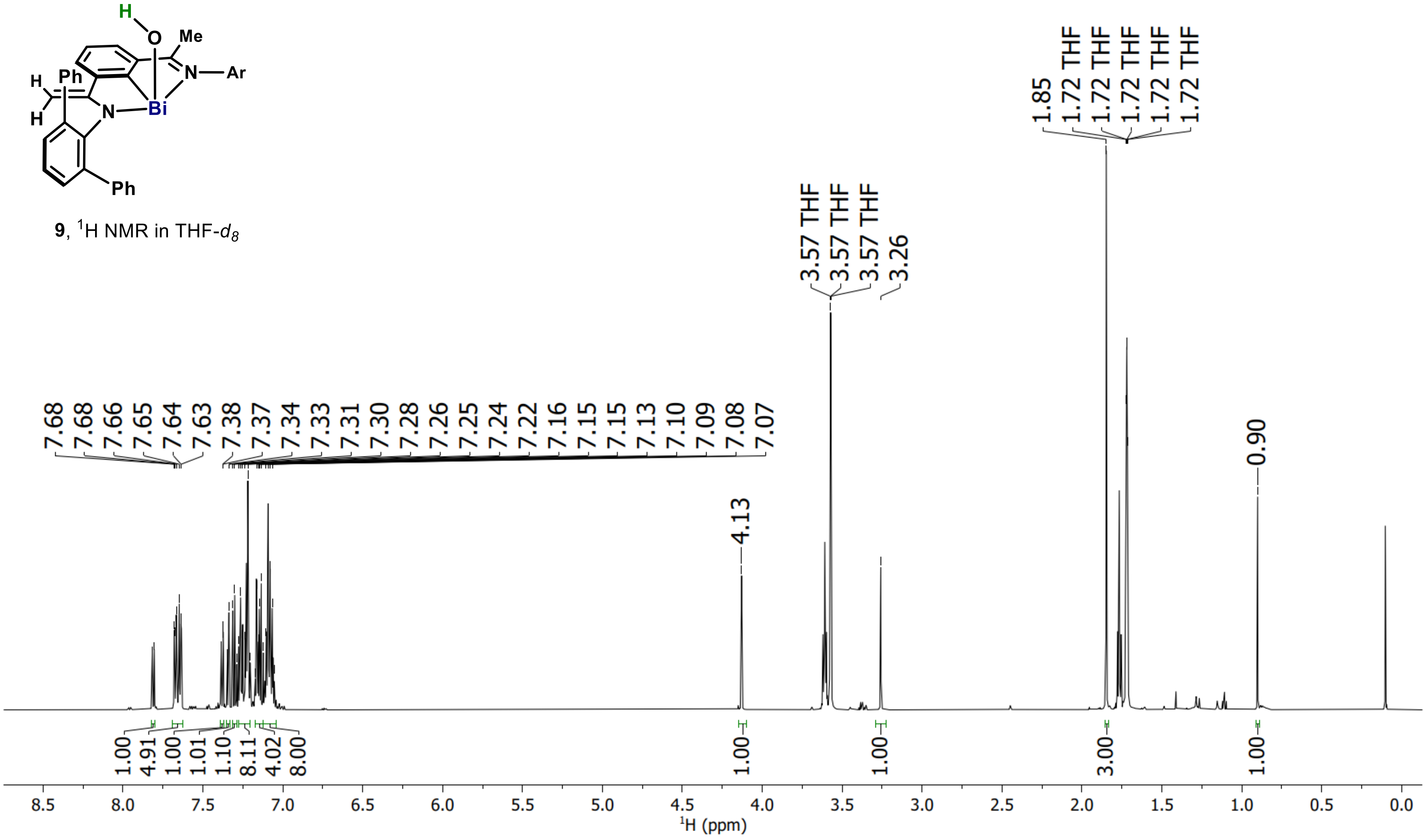

9, ^{1}H NMR in THF-d_8

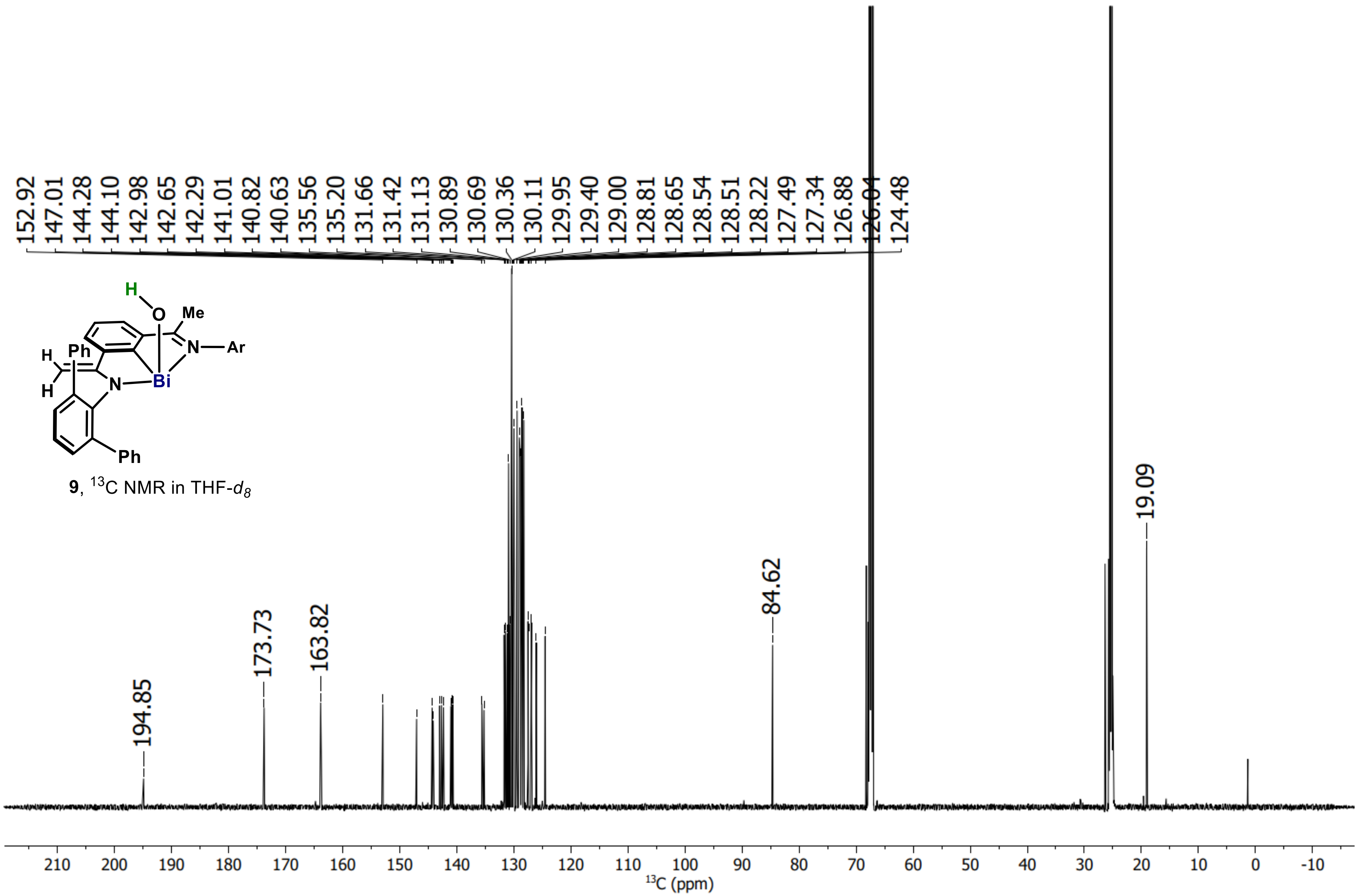

9, ^{13}C NMR in THF-d_8

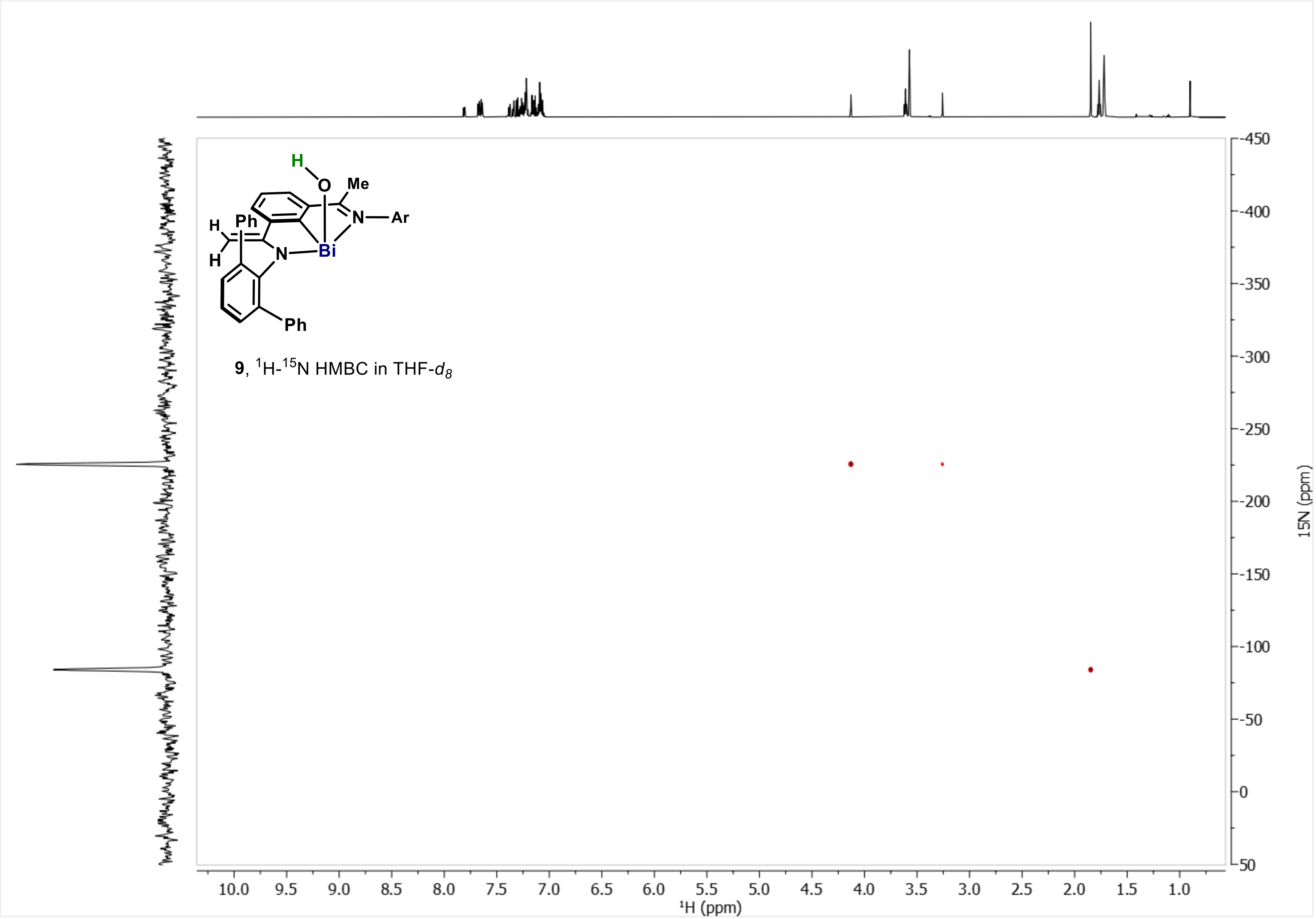
H
O
Me
N
Ar
Ph
H
H
N
Bi
Ph
9, ¹H-¹⁵N HMBC in THF-d8
15N (ppm)
¹H (ppm)
-450
-400
-350
-300
-250
-200
-150
-100
-50
0
50
10.0
9.5
9.0
8.5
8.0
7.5
7.0
6.5
6.0
5.5
5.0
4.5
4.0
3.5
3.0
2.5
2.0
1.5
1.0

F1 projection with ^{15}N Signals extracted from the ^{15}N-HMBC

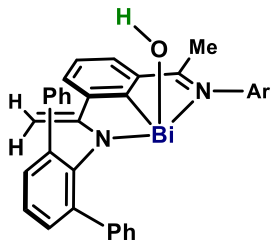

9, ^{15}N NMR in THF-d_8

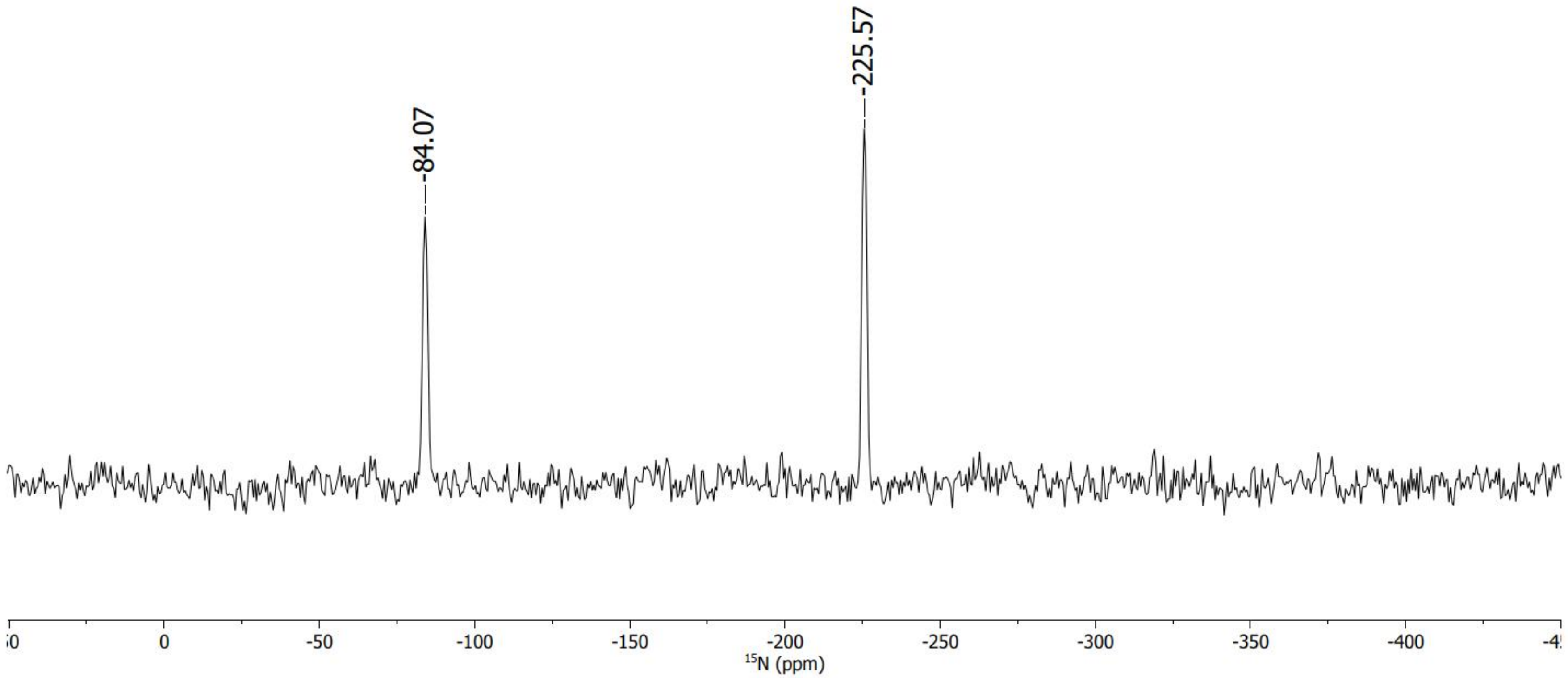

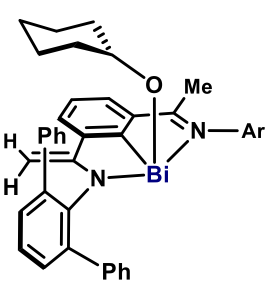

11, ^{1}H NMR in THF-d_8

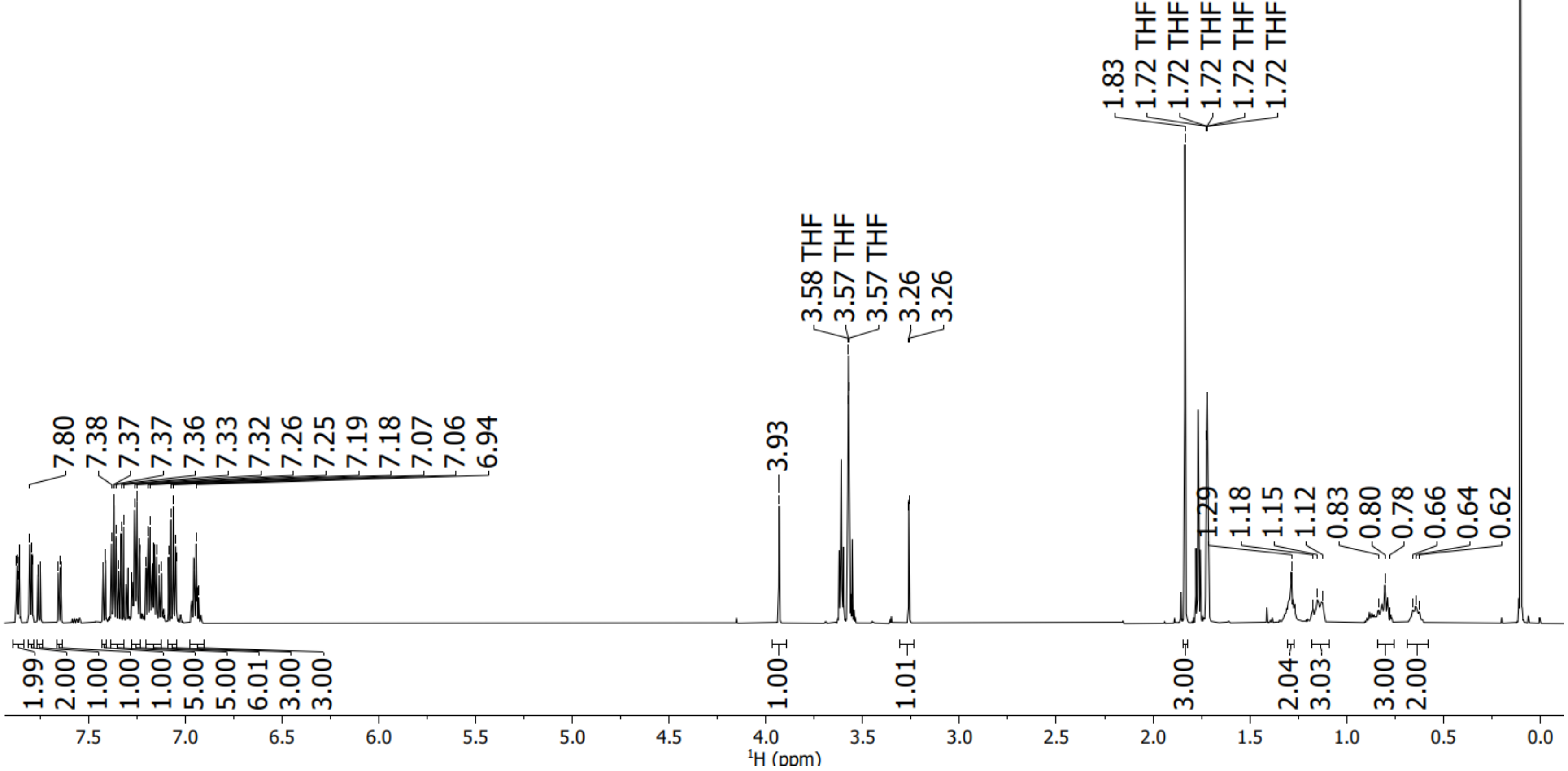

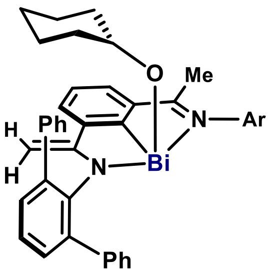

11, ^{13}C NMR in THF-d_8

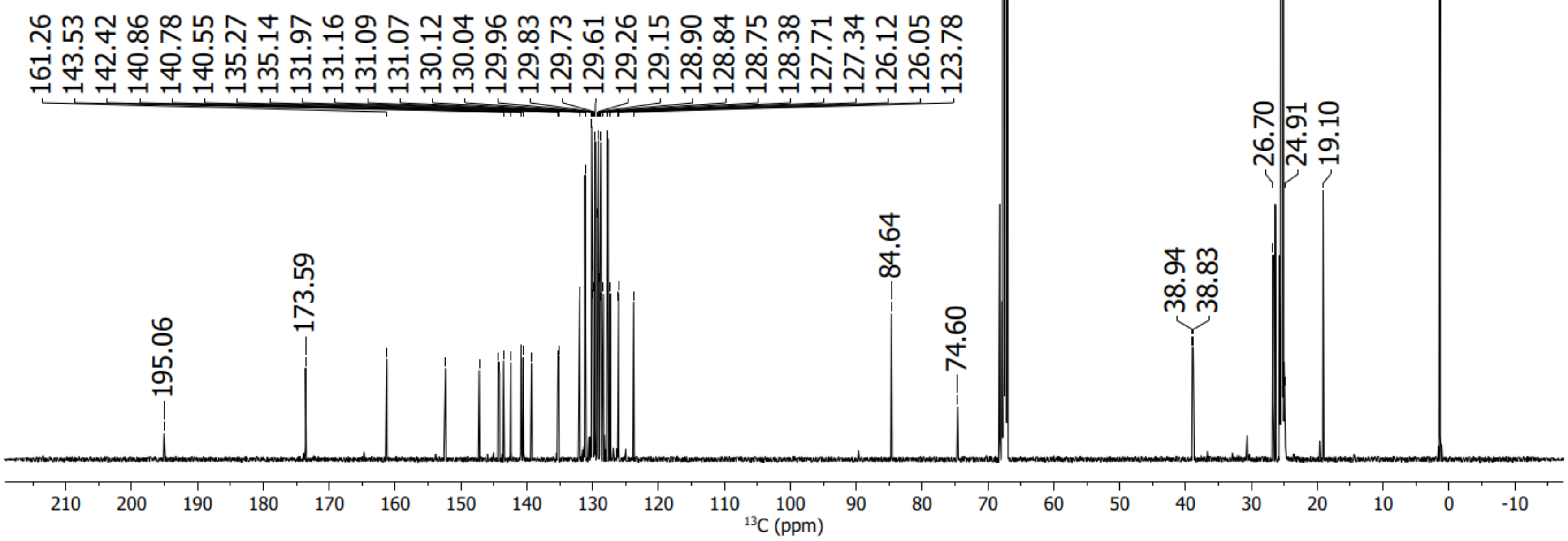

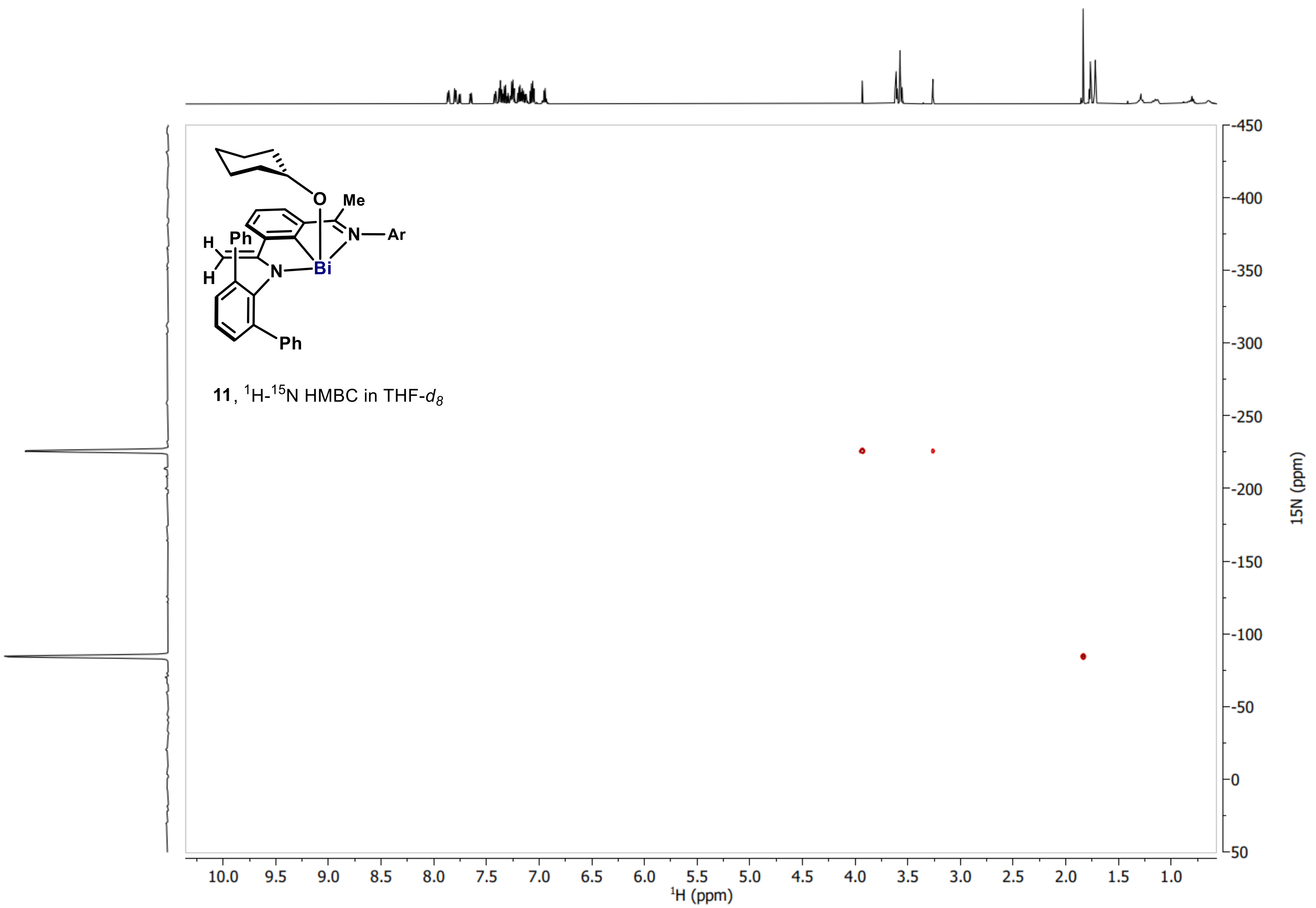
Me
N
Ar
O
Ph
H
H
N
Bi
Ph
11, ¹H-¹⁵N HMBC in THF-d8
¹H (ppm)
15N (ppm)

F1 projection with ^{15}N Signals extracted from the ^{15}N-HMBC

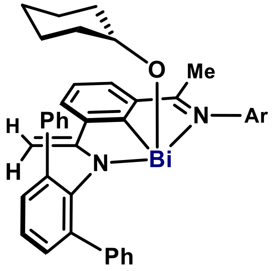

11, ^{15}N NMR in THF-d_8

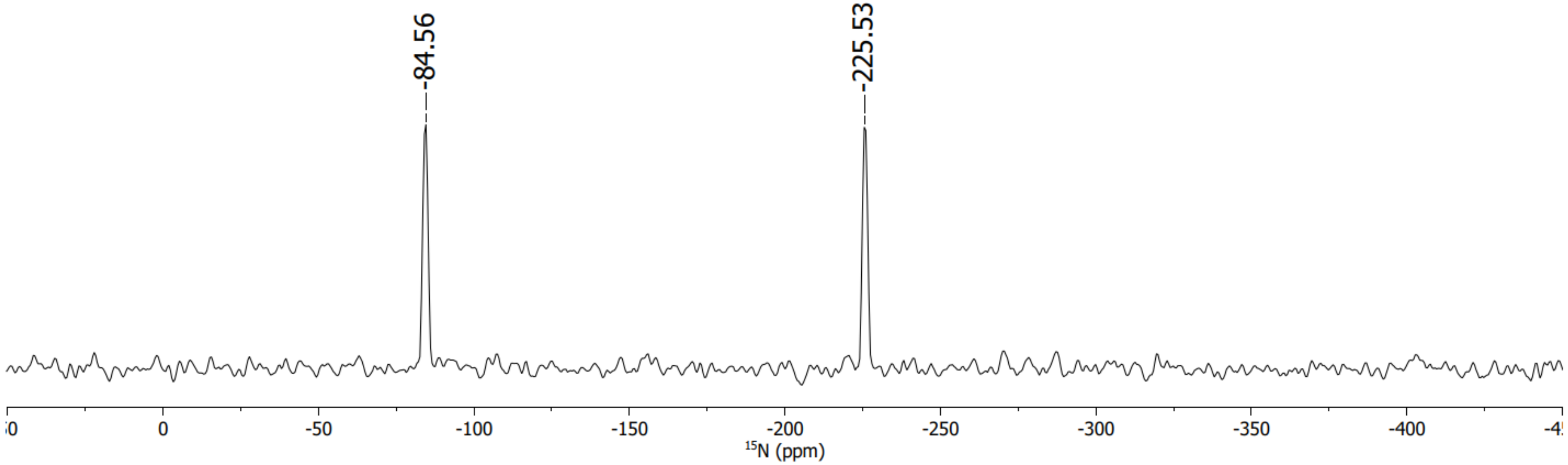

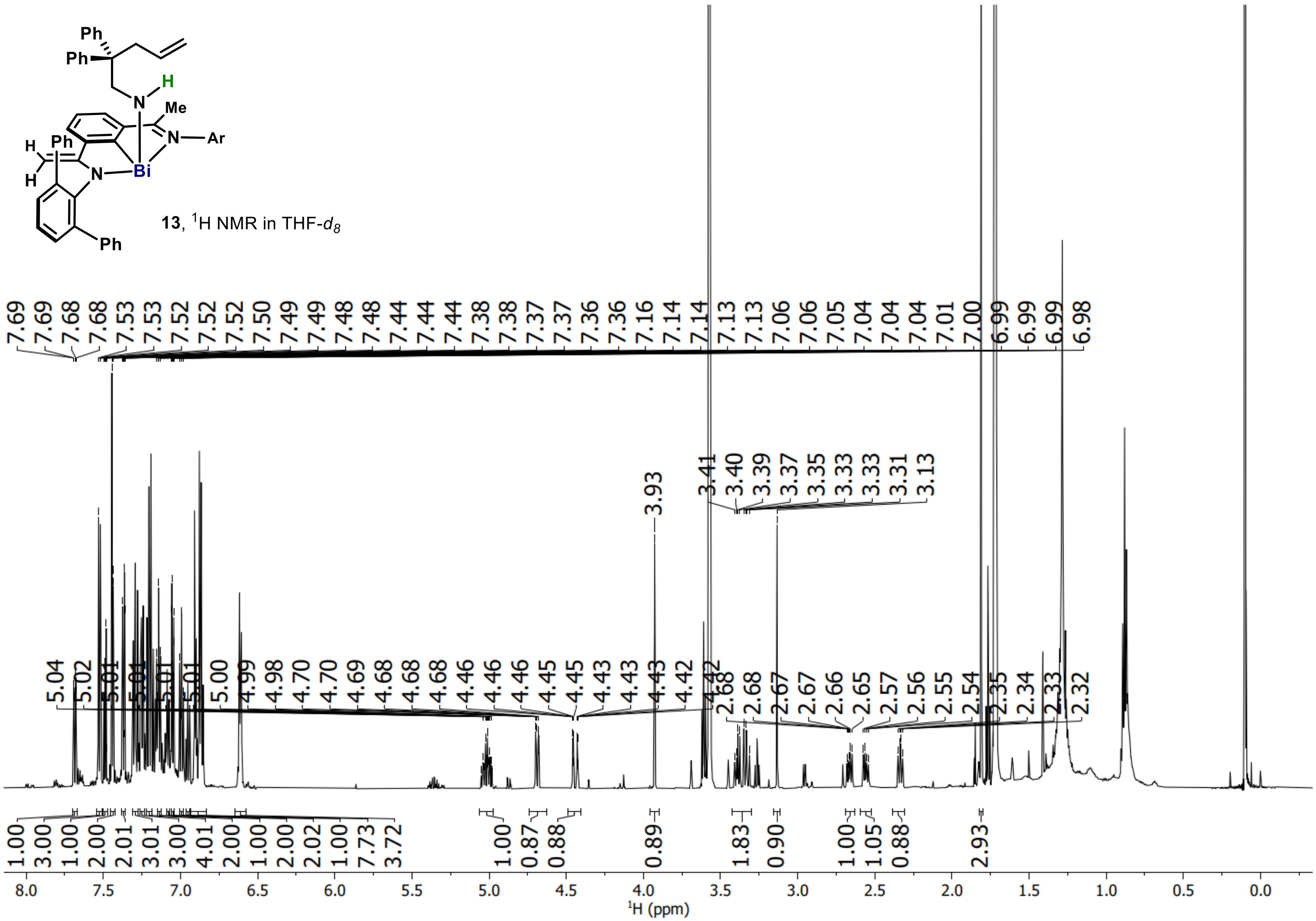

13, ^{1}H NMR in THF-d_8

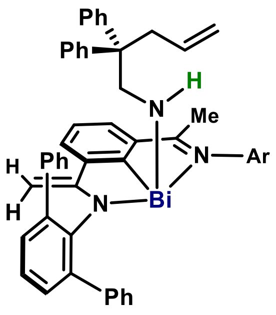

13, ^{13}C NMR in THF-d_8

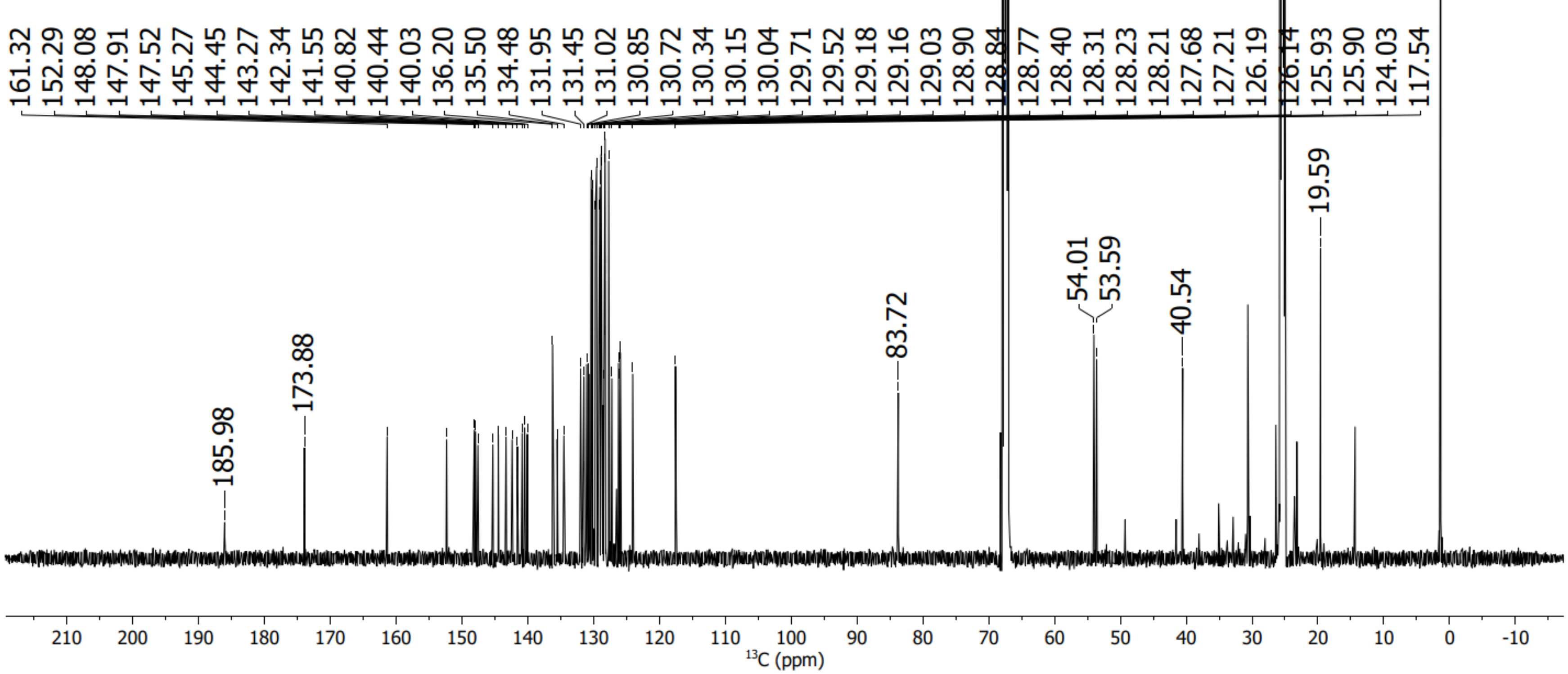

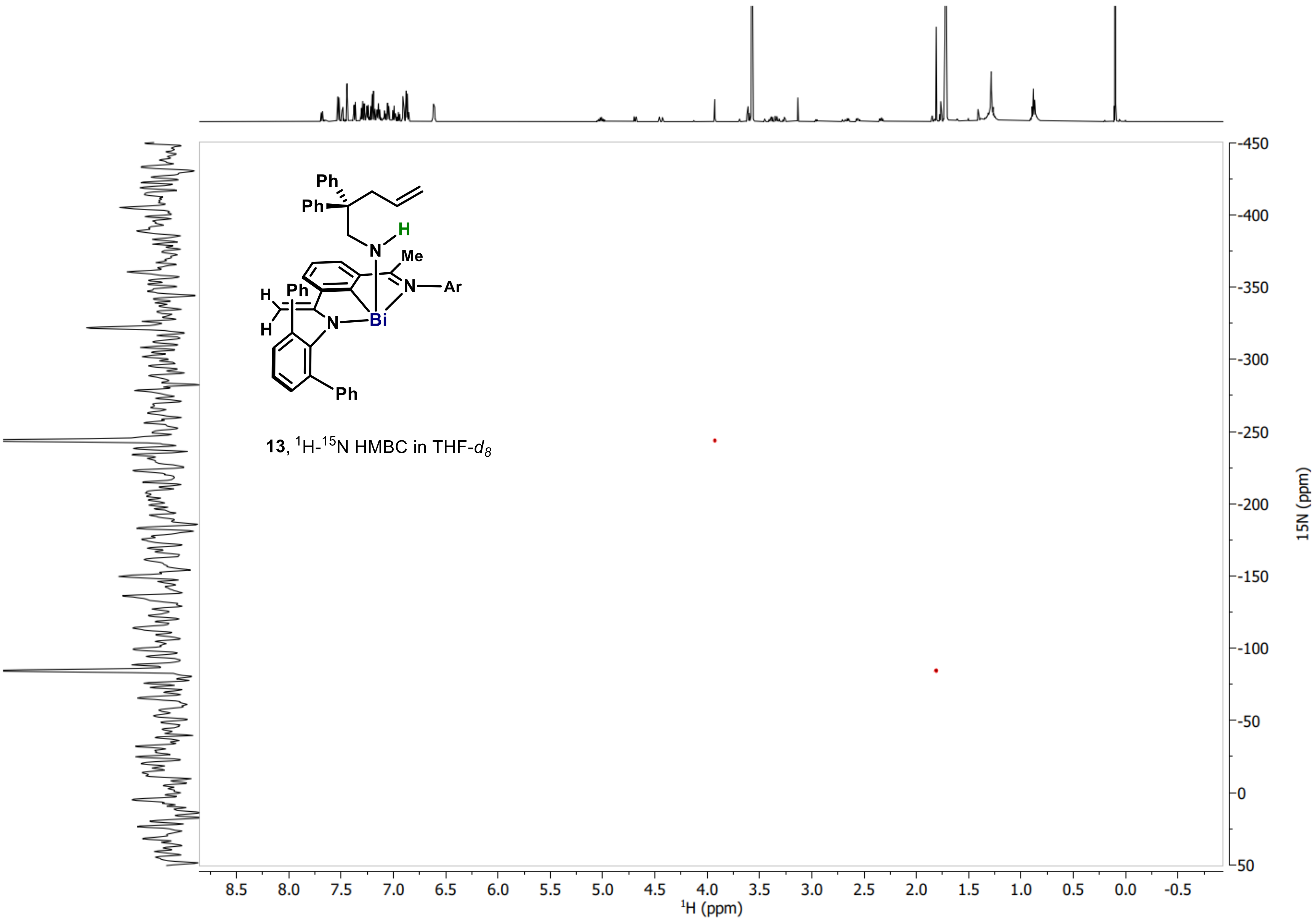
Ph
Ph
H
N
Me
N—Ar
Ph
H
H
N
Bi
Ph
13, ¹H-¹⁵N HMBC in THF-d8
15N (ppm)
¹H (ppm)

F1 projection with ^{15}N Signals extracted from the ^{15}N-HMBC

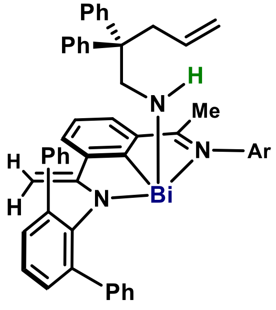

13, ^{15}N NMR in THF-d_8

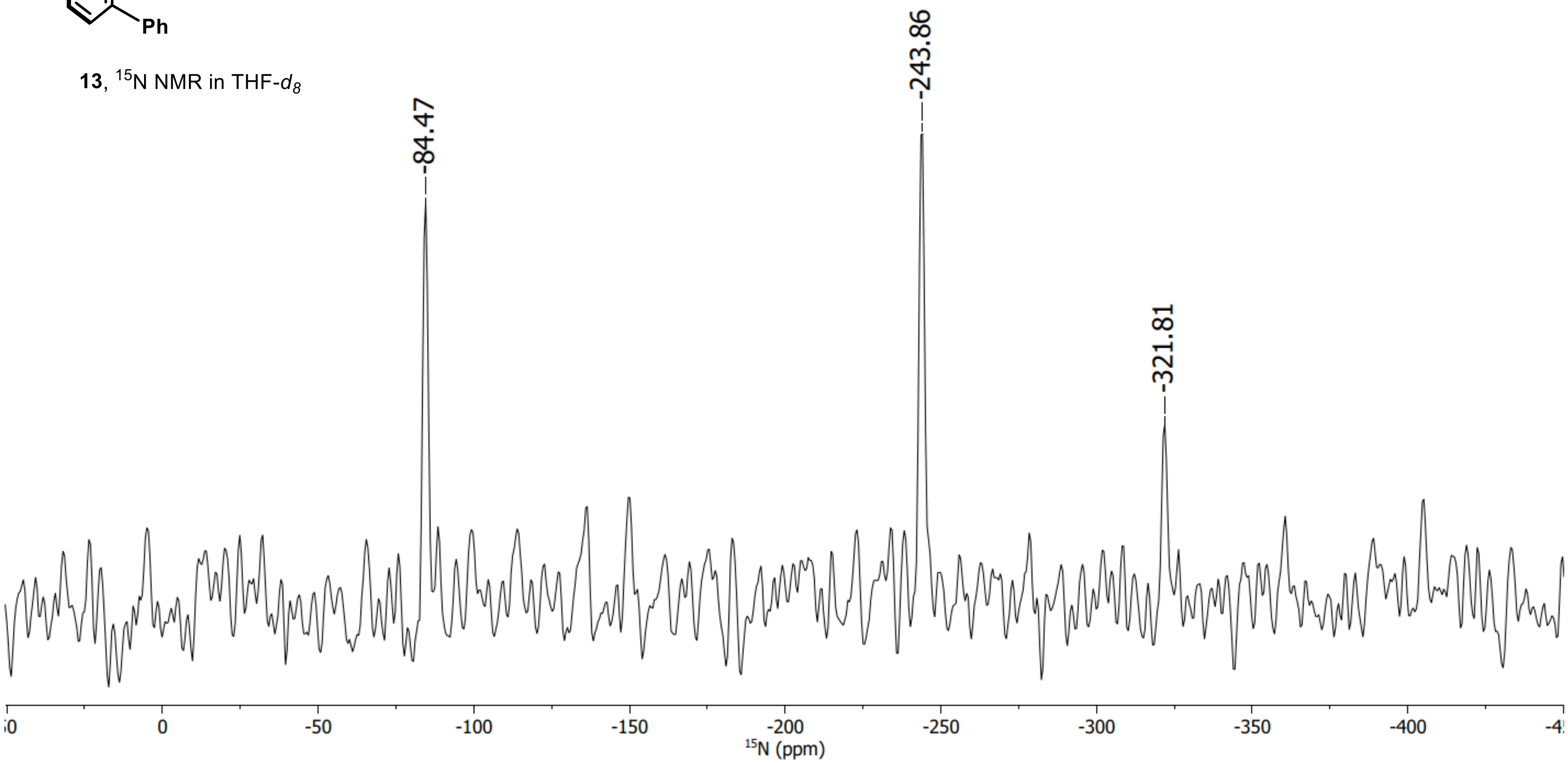

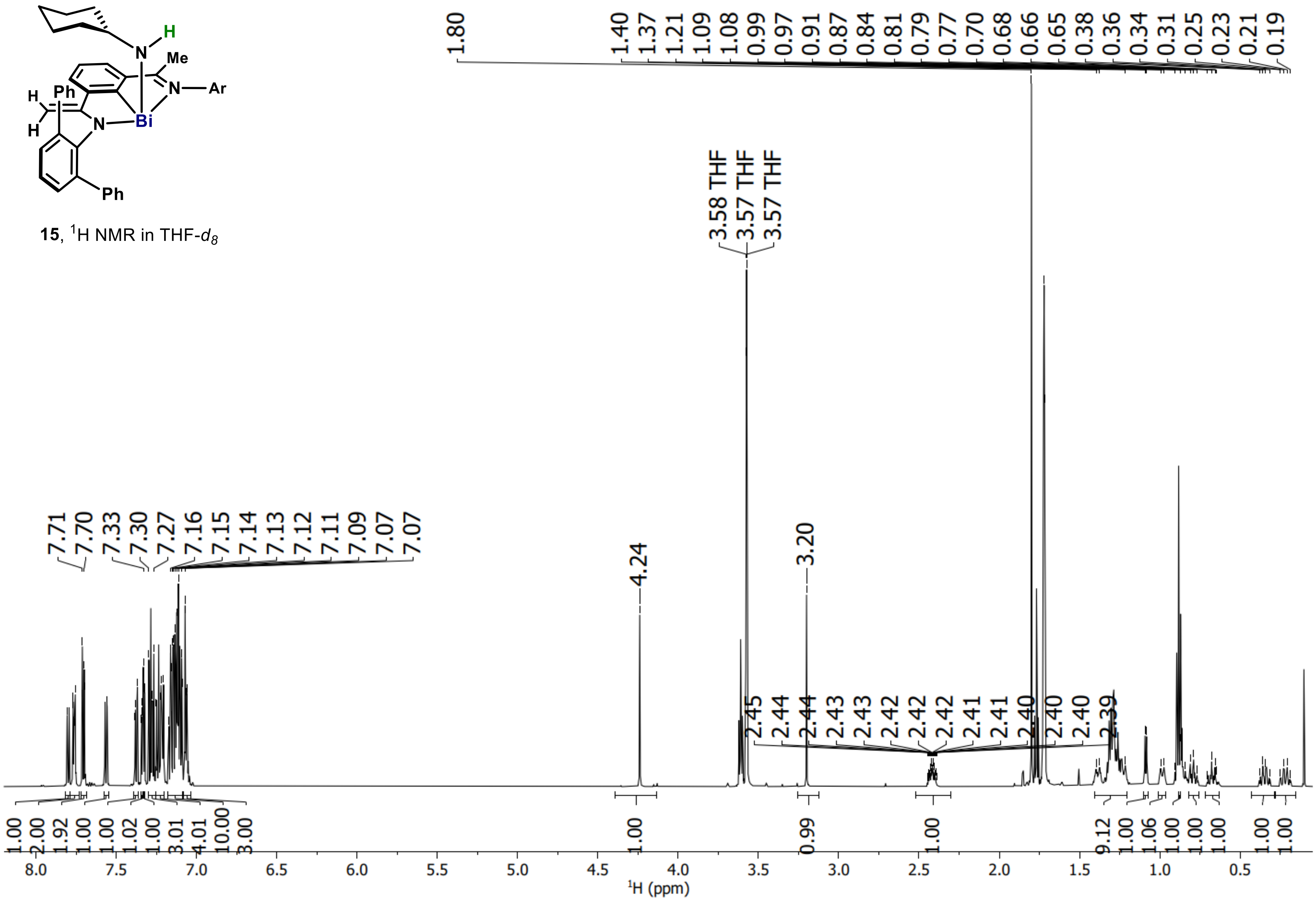

15, ^{1}H NMR in THF-d_8

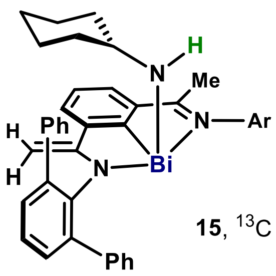

15, ^{13}C NMR in THF-d_8

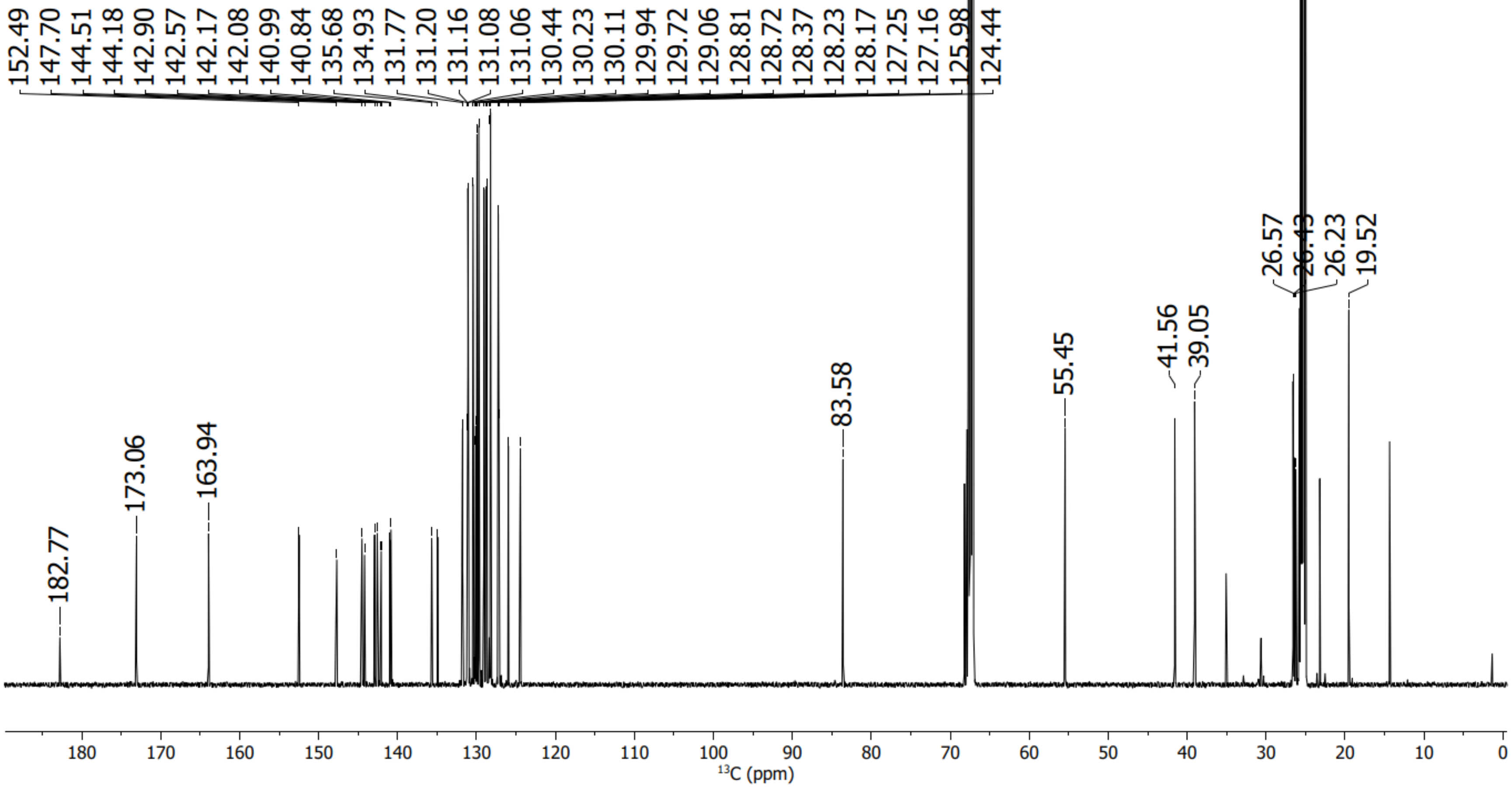

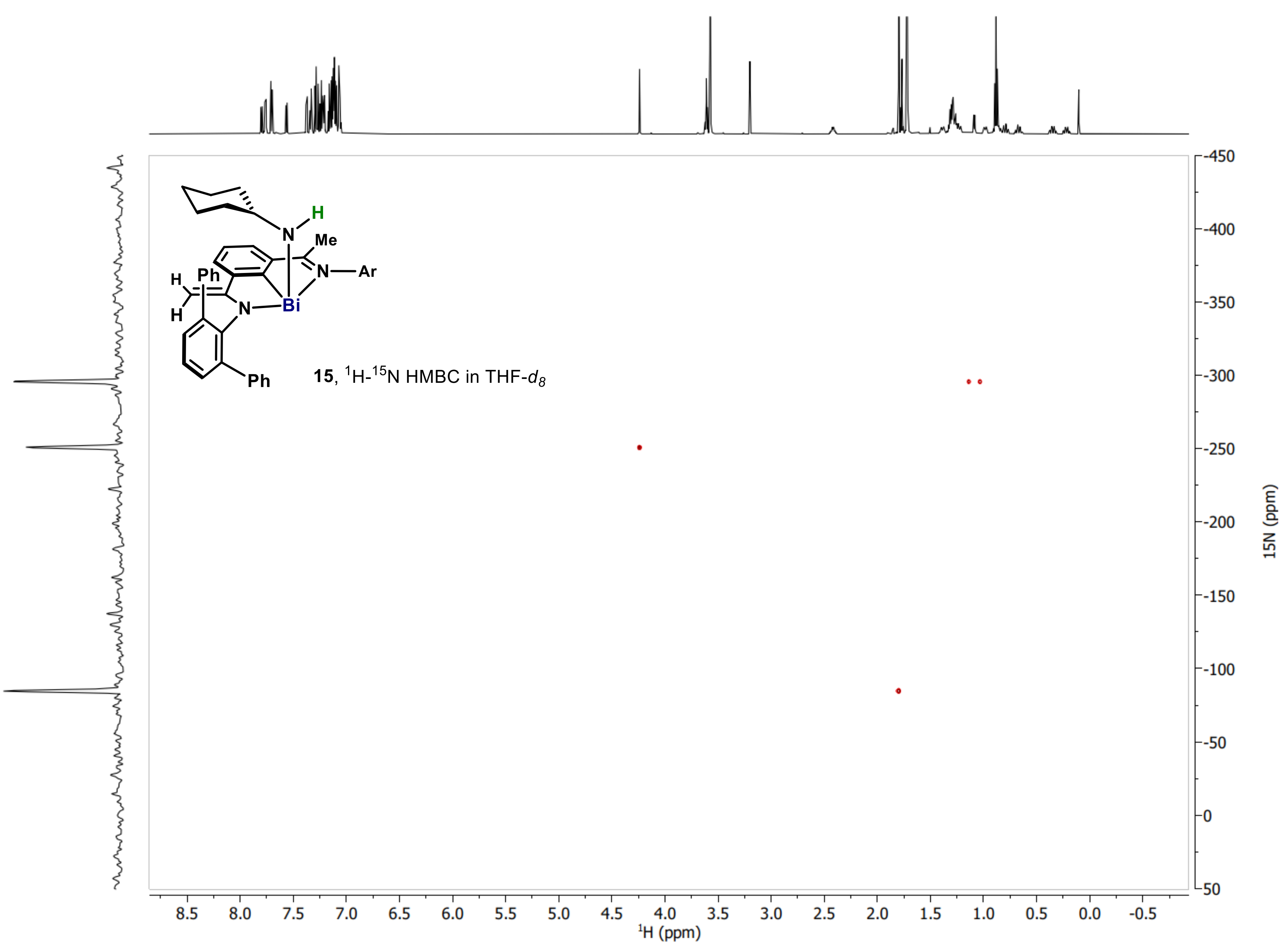

15, ^{1}H-^{15}N HMBC in THF-d_8
Me
N
Ar
Bi
H
Ph
15N (ppm)
^{1}H (ppm)

F1 projection with ^{15}N Signals extracted from the ^{15}N-HMBC

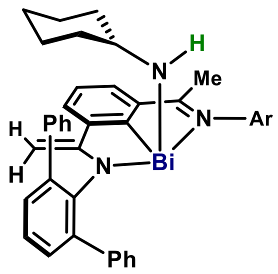

15, ^{15}N NMR in THF-d_8

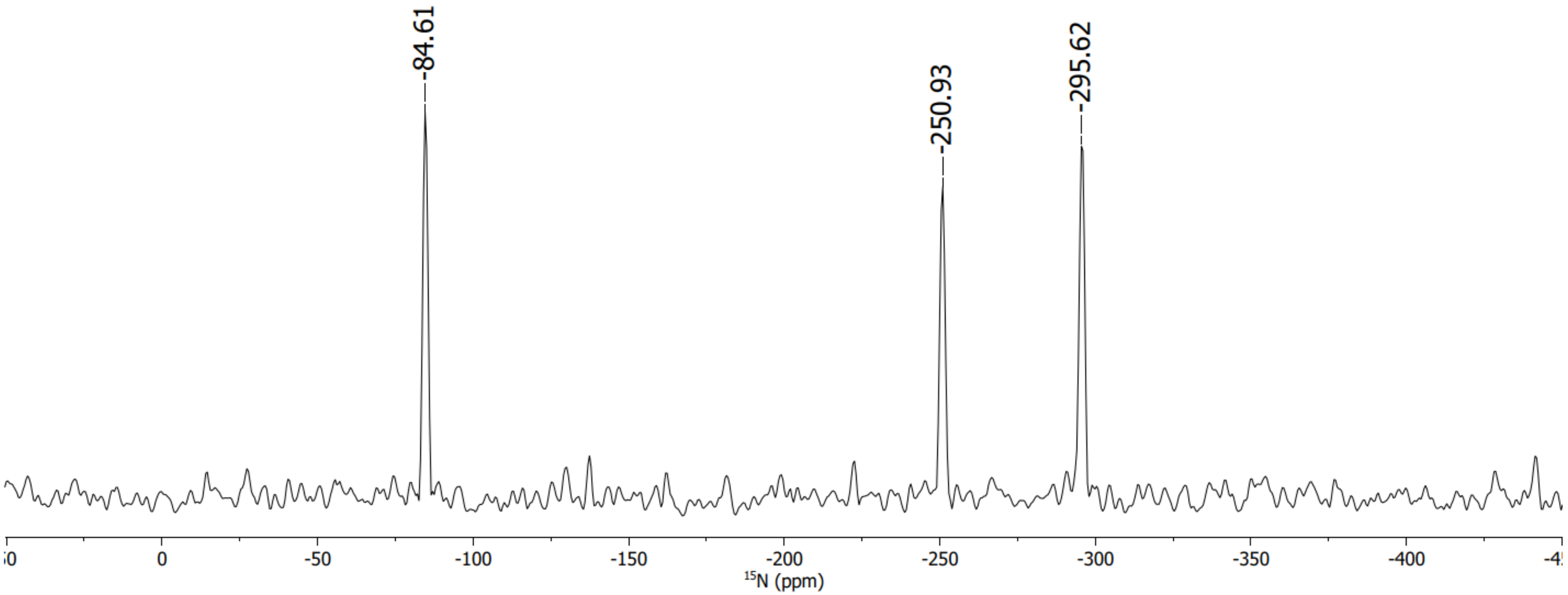

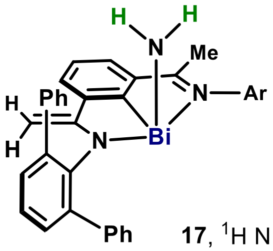

17, ^{1}H NMR in THF-d_8

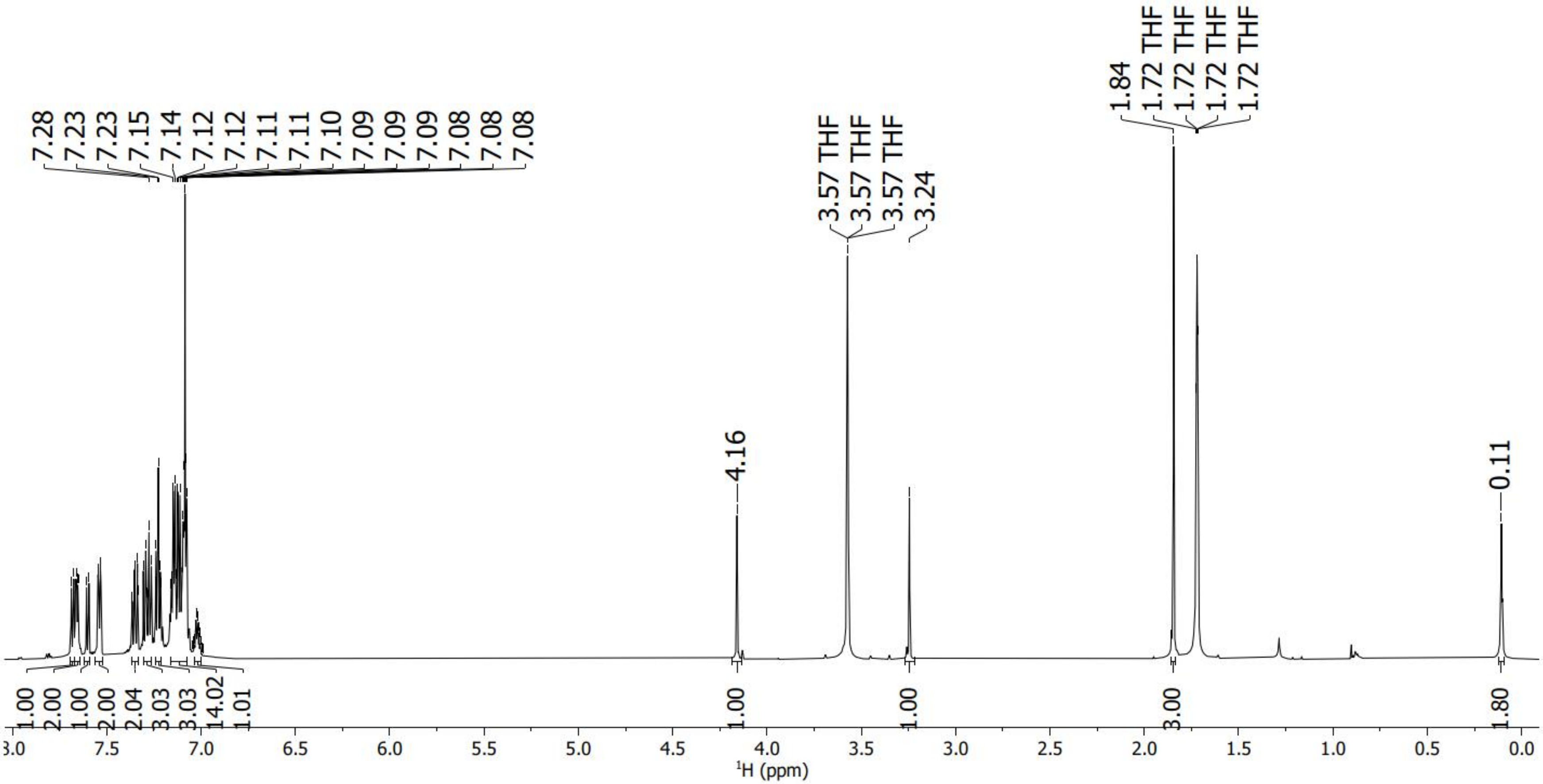

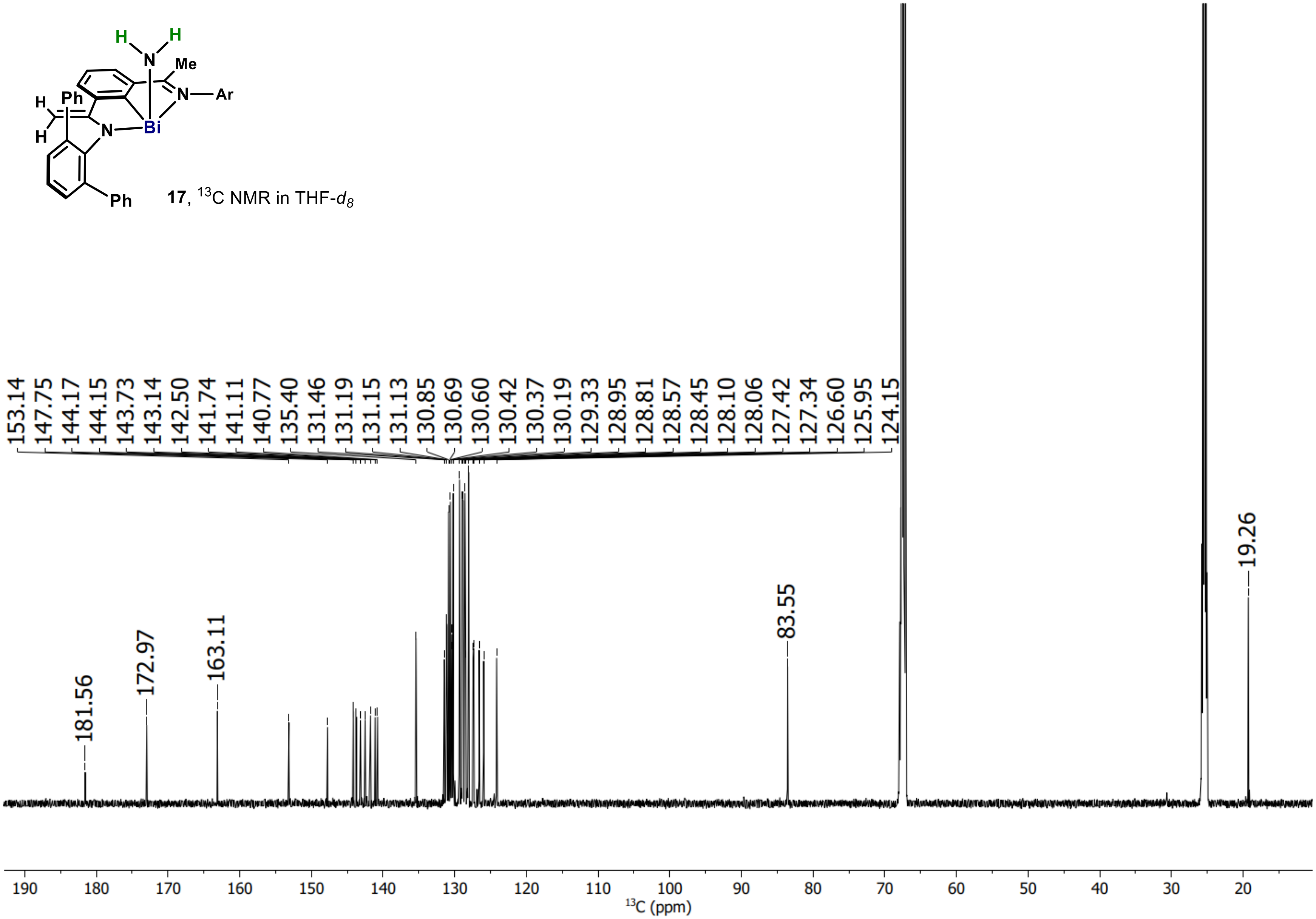

17, ^{13}C NMR in THF-d_8

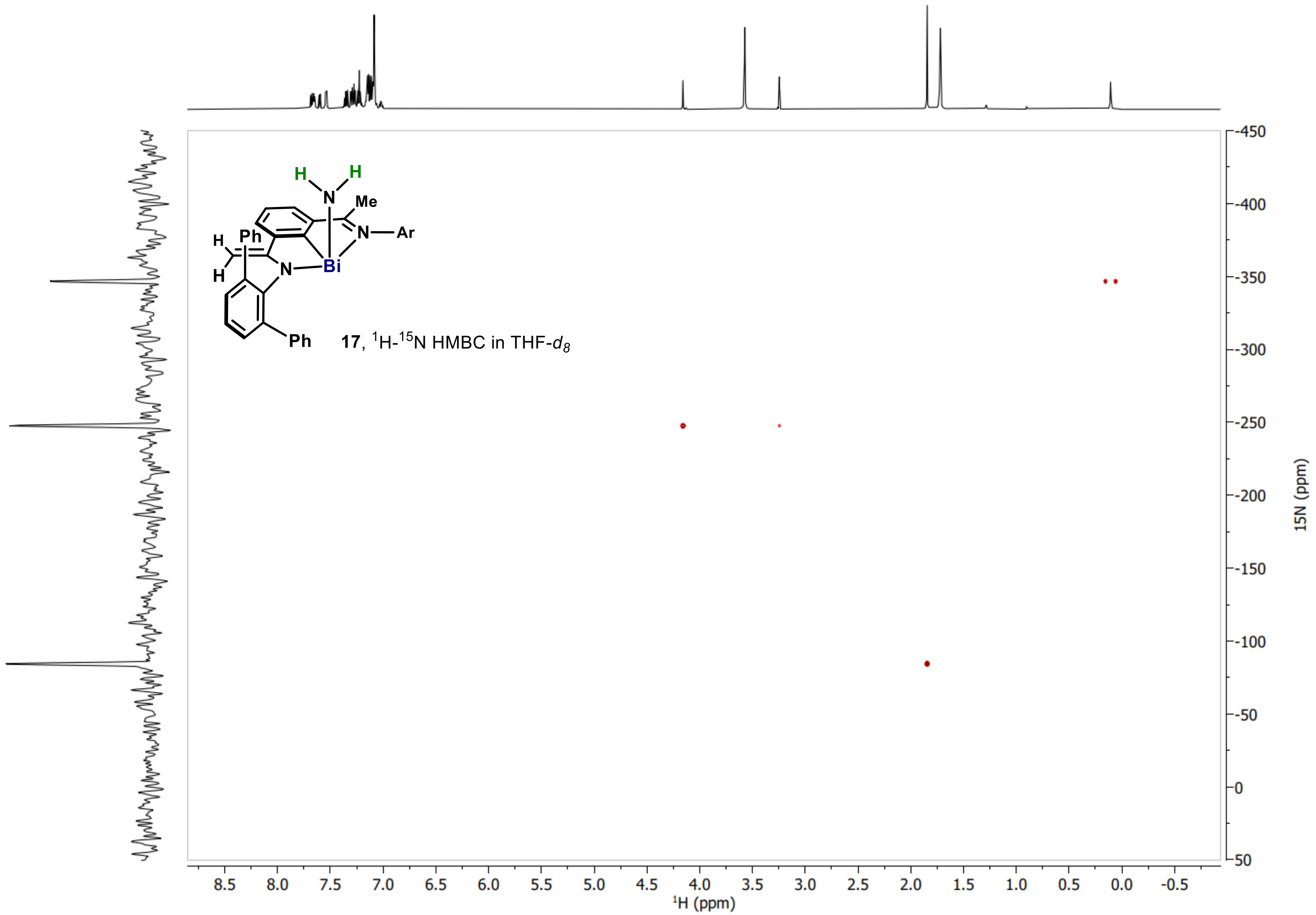

17, 1H-15N HMBC in THF-d8
H
H
N
Me
N
Ar
Ph
H
H
N
Bi
Ph
15N (ppm)
1H (ppm)

F1 projection with ^{15}N Signals extracted from the ^{15}N-HMBC

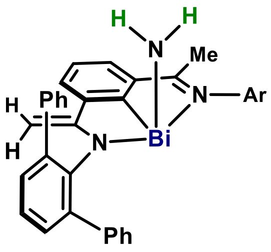

17, ^{15}N NMR in THF-d_8

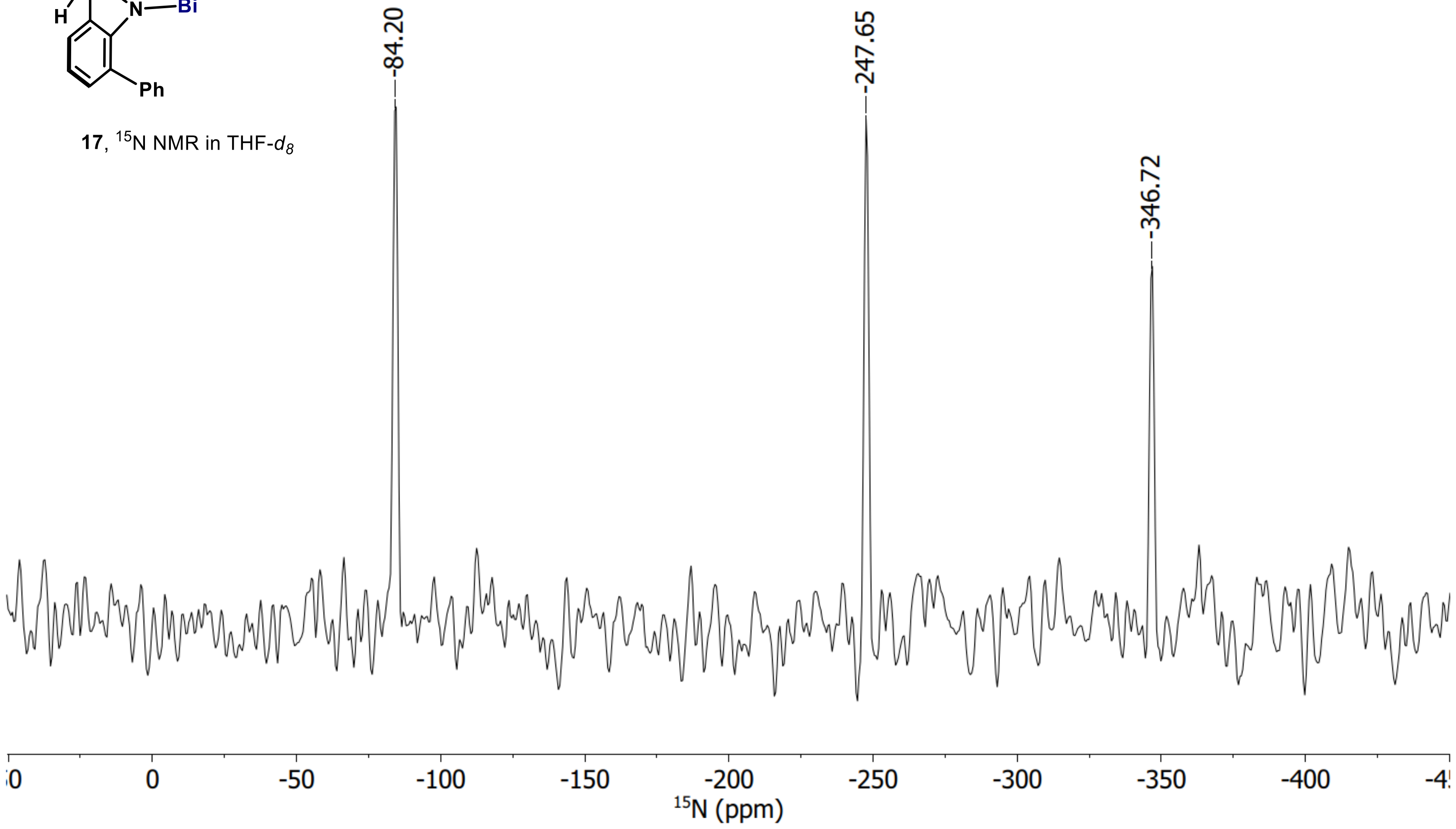

10 Coordinates from Geometry Optimizations

Complex_4

Bi	-0.06008459125406	0.86327976074044	0.33357641062662
N	-1.99610619657084	0.86673848711736	2.29166057035912
N	1.80118448955991	-0.29950535096998	-0.04168613213094
C	0.65604488172340	0.24435954388013	2.35071796090033
C	-0.03549279863064	0.48682509569106	3.53807146821755
C	0.58186133995361	0.20759930636036	4.75170654779285
H	0.07633999716192	0.39681487780675	5.69065312206691
C	1.85525123986327	-0.35175476596200	4.76182734999192
H	2.34565195173942	-0.54983757954019	5.70757975819928
C	2.47837952907221	-0.70669831783759	3.58014243069789
H	3.44738396541388	-1.18955071744051	3.60867382622637
C	1.86583177838158	-0.43252970232439	2.35294000911266
C	-1.47221099940964	0.81554158666090	3.45918189607378
C	2.38788499554023	-0.90812452573827	1.05144729330410

C	-2.26128926538467	0.94392954662391	4.71905108722149
H	-2.28709486750681	-0.01198175141987	5.24822304827098
H	-1.79374912041454	1.67941903630954	5.37678059843857
H	-3.28331131877801	1.25194962794847	4.50693548858257
C	3.25986796758853	-1.93079957293041	0.99237128343906
H	3.59363550216900	-2.33773583339264	0.04849668262859
H	3.66197423758085	-2.36839747649107	1.89337568426808
C	-3.36978595824829	0.75095358735284	2.05622822102687
C	-4.01146568477468	-0.47982451887603	2.31473431686594
C	-4.08652198911244	1.80948377868815	1.48646612752525
C	-5.34456957160524	-0.62558174311327	1.93518603614592
C	-5.41903954465223	1.62430366167855	1.13728791293409
C	-6.04524459192622	0.40680870584263	1.33941773846344
H	-5.82894710355554	-1.58056990989504	2.10373681086067
H	-5.95999862086363	2.45640235970922	0.70161596312942
H	-7.07781239286413	0.26614966404017	1.04347565117629

C	2.27264097645636	-0.56482197046309	-1.33187005863256
C	1.37227525791935	-1.01391314621756	-2.31747276379033
C	3.61180566120767	-0.31176775276667	-1.70045885175246
C	1.79791355671636	-1.19485540899799	-3.63017102895227
C	4.00727233426237	-0.54169896241521	-3.01491656441446
C	3.11607736333173	-0.97620191965099	-3.98067751314271
H	1.08275177243213	-1.53782864577244	-4.36942614936319
H	5.03494537508786	-0.32839228844934	-3.28616952809778
H	3.44656119197433	-1.13063981005351	-5.00067996816672
C	4.61998812244053	0.22227781388897	-0.75855894566626
C	5.87255754888436	-0.38520934640282	-0.68283458647777
C	4.37153163477529	1.33992015797814	0.03636741367051
C	6.85073765909922	0.09976356249284	0.17014071935969
H	6.07078933189037	-1.26502036300771	-1.28543269463105
C	5.35137066004233	1.82555067562532	0.88619251297137
H	3.40682855292455	1.82891490173109	-0.00238361511848

C	6.59189102835582	1.20851986276198	0.96102385387198
H	7.81428735490893	-0.39472848086566	0.22024023296044
H	5.14015054250457	2.69557038944519	1.49603811780379
H	7.35246461784911	1.59078849488938	1.63220110089721
C	-0.04934026281615	-1.25459968580535	-1.99043808913826
C	-1.04805058059518	-0.59984126141382	-2.71099400028406
C	-0.43180220615639	-2.10841057101127	-0.94980250173860
C	-2.38641488315373	-0.74422412788125	-2.37472373783262
H	-0.76065193919048	0.06472248743299	-3.51628953483928
C	-1.77157842274373	-2.27282461729579	-0.63285868889988
H	0.33228597985255	-2.64081839519485	-0.39381157381365
C	-2.75235631051066	-1.57156427562093	-1.32522816285019
H	-3.14224758662985	-0.19960051244403	-2.92861230002563
H	-2.05579734252447	-2.94856544496094	0.16382796320746
H	-3.79421205763717	-1.67808879303838	-1.04752975524803
O	1.10057237160534	2.66548833865965	0.19862197980312

C	1.05735088144187	3.98505610504033	-0.01844878936715
C	1.14435090923959	4.87991385256794	1.07664781723181
C	1.00255681719462	4.51422431852859	-1.33813399743377
C	1.07864563170719	6.24800116028648	0.83294837423141
C	0.94238038765558	5.89221806615580	-1.49390114712770
C	0.95294353168711	6.78785846825308	-0.43451778127135
H	1.12574765584297	6.91887413937251	1.67620530791250
H	0.88556448643829	6.29314163999073	-2.49572683672236
C	0.86111912331735	8.28569793128331	-0.69984842359367
C	1.26979938039034	4.39408507547433	2.52344872001963
C	0.98355257208025	3.64112284032950	-2.59832559665807
C	-0.41950837619661	8.59185410035560	-1.48335241074644
H	-0.44081447603228	8.06673335826615	-2.44001007994680
H	-1.30458958886590	8.28844100848692	-0.91840193829562
H	-0.49645916019890	9.66366605070251	-1.68784510948816
C	0.83461385566492	9.09848828861647	0.59075174427439

H	1.74723358415743	8.95814285798916	1.17455071146864
H	0.75183893309215	10.16197034848772	0.35355174885427
H	-0.01743915201130	8.82981399545620	1.22082412939126
C	2.07278847383743	8.73307978122198	-1.52380697491566
H	3.00074052241905	8.53634376588405	-0.98186792039449
H	2.12425820975771	8.20253510482812	-2.47645237555776
H	2.01893438334334	9.80497335671321	-1.73630324391188
C	-0.05369023851473	3.75967338275785	2.94598180827126
H	-0.84509239440516	4.50968158649806	2.96797721684985
H	-0.36914150804847	2.98314347216777	2.25424372605534
H	0.03468865587197	3.31430532888571	3.94093805276716
C	1.53740450314410	5.53839382782972	3.50316481838526
H	0.70929705623340	6.24954111011923	3.54643128057847
H	1.65951673408877	5.11940997877852	4.50519532533301
H	2.45213133741785	6.08346242355091	3.25727152712336
C	2.43101052917842	3.40892870113490	2.68443882642482

H	2.30449072696386	2.52311709071322	2.07121143647334
H	3.36992171408272	3.89183280943112	2.40194699138564
H	2.51196058543649	3.09618791572171	3.72945182903025
C	-0.36748162847307	2.93654416388097	-2.72552180771330
H	-1.18294058405635	3.66330439499291	-2.75893265873633
H	-0.40145339567842	2.34714886782737	-3.64575329934327
H	-0.56354886936615	2.25920508367334	-1.89472801511377
C	1.15935852087786	4.45894366036777	-3.88005850113338
H	2.08274876120791	5.04202282129006	-3.86355140108705
H	1.21614027274164	3.77020414321290	-4.72652589638780
H	0.32296982960723	5.13659415053555	-4.06751964365874
C	2.12058315641588	2.62151235065754	-2.58266702291913
H	3.08862288118651	3.12789810626511	-2.59185443565524
H	2.08652357906396	1.99866227918047	-1.69732021963349
H	2.06831653146938	1.97235669205183	-3.46097147451546
C	-3.34725693729348	-1.60432458180084	3.01284272241795

C	-4.03664177912069	-2.27700859574200	4.02447152170986
C	-2.05440991080368	-2.03292981279273	2.71131329056393
C	-3.45724536973798	-3.33657441306230	4.70440866100815
H	-5.03320648417902	-1.94649116498201	4.29477856536819
C	-1.47217404131169	-3.09049678439460	3.38840879699367
H	-1.48670055893839	-1.52933464511972	1.94345508374146
C	-2.17067337065537	-3.74819063723199	4.39028172253857
H	-4.01240878158763	-3.83641659978323	5.48984236904625
H	-0.46311286552973	-3.39265723282150	3.13205097382531
H	-1.71347367175928	-4.57156393155688	4.92620043393533
C	-3.48536175982464	3.15579767879158	1.32546510661988
C	-3.78509698800693	4.14562734738023	2.25714722329868
C	-2.68176725374258	3.47986766826364	0.23594767796011
C	-3.29486684959963	5.43384287021527	2.10200221513028
H	-4.41534140306802	3.90064977575461	3.10504330243909
C	-2.18455101654362	4.76546001707525	0.08375729490886

H	-2.46670771604425	2.72348883414935	-0.51195574561972
C	-2.49256727802454	5.74441892073028	1.01605129533622
H	-3.53704486250210	6.19336920230604	2.83672305768332
H	-1.54306913798932	5.00724628393910	-0.75355680043823
H	-2.08984571890545	6.74265784916770	0.89753447671298

II

O	2.68575230476221	5.11102898748961	5.77097561470969
N	5.51982601315195	8.84373822091150	6.05671410663410
N	5.75746595863250	6.82302881125938	1.93436492027628
C	4.23971676473748	8.18092811994716	3.71344593904448
C	3.55460811981936	8.87094904555243	4.72588119600706
C	2.29802479464297	9.41759192707226	4.43175713542329

H	1.73315164082211	9.95375460634359	5.18925201558094
C	1.77109586411656	9.27485591876371	3.14664711756433
H	0.79338899626238	9.70042021815001	2.92270788278242
C	2.46746423490895	8.59370686967796	2.15183898313013
H	2.02669967429873	8.50099907675012	1.15914200949411
C	3.72893981175442	8.02762454950694	2.42606377294123
C	4.25983840546356	9.18656690798227	5.97725261194471
C	3.52534720310006	9.93318649984328	7.04766883250447
H	2.59455012134578	9.39154903095305	7.27336653328028
H	3.24990312181863	10.94032878094419	6.70476225760978
H	4.13086916782730	10.02833360614415	7.95535909758992
C	4.54567278704484	7.28299110376606	1.43298224010075
C	4.12974035726639	7.05966217370693	0.16054773151907
H	4.74222910548476	6.49584422604438	-0.54026157599690
H	3.18201123467716	7.44853670505916	-0.19998251386621
C	6.47745914621012	9.47570878281107	6.85969396895068

C	7.47171635700105	8.70399750185202	7.51065866093991
C	8.48819324796497	9.34214784631143	8.22993507411915
H	9.23441733871608	8.72620505849179	8.73378784023633
C	8.54889637042713	10.73236335573920	8.30958400977642
H	9.34220520160702	11.21787070954570	8.87833182413778
C	7.61240473811787	11.49501313629760	7.61727765502480
H	7.69205546501460	12.58305509968838	7.60947108730305
C	6.58963474775909	10.89897706867335	6.86600254356859
C	7.49661877791990	7.21888478243080	7.42444084562627
C	6.60683999881912	6.42859815562225	8.16072570113291
H	5.86035903274987	6.91191170938084	8.78705478834948
C	6.67262856117495	5.03414029814952	8.09941310462142
H	5.96591100132332	4.43570901777328	8.67525463829269
C	7.62971096114081	4.41135592670422	7.29292405552574
H	7.67110015745957	3.32356518230666	7.23452411020432
C	8.52980541042681	5.18980280948869	6.56025036095872

H	9.27842114047369	4.71228532548380	5.92767384341758
C	8.46954630782546	6.58279198198292	6.63264861226448
H	9.17654930606602	7.19264398203259	6.06757191086030
C	6.80770666086790	6.46213583634284	1.07276224928424
C	7.41233698647946	5.18876736931600	1.18651115236861
C	8.50804626418534	4.86650021029976	0.36986567590585
H	8.95769403672786	3.87642011819400	0.46206716999356
C	8.98755750925814	5.76995067113390	-0.57467299938527
H	9.82215238955076	5.49756761951475	-1.22150185959113
C	8.40773956931368	7.03723340980409	-0.67171251624702
H	8.80970928739892	7.77490443754280	-1.36852650921056
C	7.34752252239613	7.40762279821381	0.16155534248642
C	6.91844801249256	4.19263659764644	2.16792311519724
C	7.80566036690618	3.62787350259076	3.09966513628781
H	8.84340204901459	3.96595239777120	3.11152964354944
C	7.37140704571462	2.65845776450740	4.00642598675263

H	8.07441149559672	2.23361564791267	4.72437681306633
C	6.04191229222764	2.23240978985511	3.99120785778979
H	5.69665363822866	1.47683419978049	4.69764851921502
C	5.14800699094166	2.78894032206431	3.06824339767357
H	4.10500818584630	2.47297428273610	3.05971325270655
C	5.57813363742628	3.76827111479767	2.17173570372435
H	4.87261105841294	4.21510639291044	1.47097715943486
C	6.86294807331182	8.81181759406126	0.15751437631182
C	7.08973413588090	9.62950219723471	1.27537055148138
H	7.64108697454505	9.22474245360477	2.12771177974043
C	6.67104791377135	10.96001467807969	1.28538420074271
H	6.87625186696094	11.58814709457156	2.15268885420747
C	6.00878492852343	11.49172699406960	0.17598450803751
H	5.68034575393302	12.53211876151053	0.18190923843005
C	5.78769373598713	10.69106554754354	-0.94693563995314
H	5.27764131433084	11.10180396962337	-1.81957783658943

C	6.21310949438559	9.36135515228503	-0.95576526036841
H	6.02890671340517	8.73088170096317	-1.82660545022560
C	1.69231976221608	5.97413814052090	5.69325362031744
C	1.33299177012918	6.79521054271526	6.82401075141723
C	0.34701252982388	7.77039594809919	6.67122315459548
H	0.08767208215759	8.39157698157984	7.52928097522203
C	-0.37666042441145	7.94789060011352	5.48362951431109
C	-0.12336551972675	7.03987974988672	4.45036756182814
H	-0.72809280068544	7.10132294063910	3.54910230280898
C	0.85597739314136	6.04683256786029	4.52607816035513
C	1.87605865270707	6.45717404546703	8.22374307723341
C	1.44832743885035	5.00859174778532	8.55626240589415
H	0.35201616483750	4.92355942609236	8.55274426658686
H	1.81387853361365	4.72136123833022	9.55555242992580
H	1.85718473614979	4.31352336927238	7.81275002853975
C	3.40709738243385	6.54580521184483	8.29430481142902

H	3.75255312242610	6.26700286111133	9.30383949577392
H	3.75825487615094	7.56505774906661	8.08811370365088
H	3.85530869697013	5.86957593031163	7.56231518537006
C	1.30764020986330	7.37545400022040	9.31842229754876
H	1.57776454275622	8.43091778988208	9.16011084233241
H	1.72004122654558	7.07291850176432	10.29265044829411
H	0.21238804283295	7.30881290174481	9.38516107211197
C	-1.50002841949813	8.99196334610348	5.41876364192489
C	-2.16462021941348	9.05088106959863	4.03480314494917
H	-2.65381743345446	8.10095177639450	3.77708937443353
H	-1.43550807730258	9.28085457246673	3.24406652697453
H	-2.93356021631136	9.83750216904066	4.02414646442484
C	-0.95975708503290	10.39872387172022	5.74919837829338
H	-0.26574451414872	10.74890481102886	4.97125466987820
H	-0.42777376187578	10.41082066933060	6.71094041784297
H	-1.78488383081039	11.12537363062517	5.81044370745392

C	-2.58628993922966	8.62257154068017	6.45430469475995
H	-2.18148066820425	8.62825029010693	7.47579093864584
H	-2.97777047078175	7.61485087827963	6.25528723393755
H	-3.42378615588681	9.33726102126938	6.41444462704976
C	0.92444233434186	4.94787973750007	3.45071478854287
C	0.62153719558445	3.59318519581144	4.13226351746225
H	1.35773714691112	3.39417297365041	4.92059036305851
H	0.65854493255963	2.77791704060908	3.39149156659594
H	-0.38224003397340	3.60361171902621	4.58196083733678
C	2.30241585042407	4.87178624611797	2.77501794155107
H	2.30805750453076	4.06138654502573	2.02739984475516
H	3.07946112851771	4.67104428939526	3.51606876309572
H	2.54056884190014	5.80505085368142	2.25536046860755
C	-0.11365047105817	5.15713599798933	2.33601718486540
H	-1.14283274332943	5.16092338065498	2.72345942473098
H	-0.03380926258039	4.33433522005126	1.60984125936087

H	0.05471130436000	6.09903244507353	1.79145810149789
Bi	6.19947979940161	7.25462768430357	4.09941275280761
H	5.95675775669736	10.65576287013131	4.20850114745763
C	4.63238415114077	12.32329275582836	3.93537949393058
H	4.45204726291674	12.08182664454391	2.88749812574920
C	4.00793714345199	13.42409576547094	4.52789020818578
H	3.33210809119649	14.05088409486110	3.94498402459998
C	4.25593294403492	13.71625045827121	5.87132519874880
H	3.77791665809377	14.57647935477786	6.34223932037136
C	5.09915785376061	12.89689876849156	6.62342996795730
H	5.25874177844270	13.10534191215821	7.68273114706643
C	5.48317364911813	11.51374315372709	4.68469848456018
C	5.71439531029968	11.77303779278804	6.04660756107872
N	5.11878149457876	5.45832991216996	5.10385740921502
H	4.00868246622504	5.45978203841273	5.36280843654279
H	5.25925647813791	4.65728010424771	4.47327468962276

H	5.63578446632562	5.23499456215886	5.96349615212720

$TS_{II\text{-}III}$

O	2.68584496166988	5.09877925497844	5.77004830375213
N	5.53378617305613	8.85962578367862	6.05487007337296
N	5.73908303908068	6.81400518061803	1.93182691992837
C	4.24755068208270	8.20274902482409	3.71030432587140
C	3.56646278342138	8.89046861490391	4.72563153507289
C	2.30489112079392	9.42980536040824	4.44153337836515
H	1.74024274320374	9.95928752289641	5.20430357049911
C	1.77070287779319	9.28715653245589	3.15933899848252
H	0.78850920429715	9.70609318826549	2.94210307827380
C	2.46551095372153	8.61251122345396	2.15923975743113
H	2.01818497444856	8.51797449063895	1.16952031033486

C	3.72945103902420	8.04881987679264	2.42579776286124
C	4.27573916981832	9.20284353885413	5.97752539007520
C	3.54173308319098	9.94422566007387	7.05218496133141
H	2.61315791675352	9.39888376501329	7.27826643041458
H	3.26198402572735	10.95164709046258	6.71386875438396
H	4.14898160506177	10.03745310554080	7.95891665868184
C	4.53749385893040	7.29850483823560	1.42953883092463
C	4.12059177622383	7.09397204694228	0.15404318113871
H	4.72381993773811	6.52482685989866	-0.55040570540964
H	3.18422354164194	7.50911786672935	-0.20684700550893
C	6.49061519329452	9.48566133622512	6.86298525984470
C	7.48043286228469	8.70969989453519	7.51521755358109
C	8.49562869431916	9.34382188209426	8.23977459707663
H	9.23864027766176	8.72482639648389	8.74462303388893
C	8.55990914433734	10.73374056542606	8.32232322496748
H	9.35286779262456	11.21586740715104	8.89444855758960

C	7.62774215057362	11.50050059684639	7.62872112229143
H	7.71015036644723	12.58835000960775	7.62368267027809
C	6.60564472827537	10.90866765948305	6.87328397371988
C	7.50134074622423	7.22474681041469	7.42551684192049
C	6.61234491473810	6.43538532733729	8.16360599610547
H	5.87030530802141	6.91985823432725	8.79446860373178
C	6.67351885087422	5.04101725181664	8.09821492777135
H	5.96771094497584	4.44279240146000	8.67537350369545
C	7.62515704007866	4.41780099235824	7.28621029454051
H	7.66197283433873	3.33013555588184	7.22356245958073
C	8.52489853531350	5.19513400123275	6.55241931780206
H	9.26881579612339	4.71691763410349	5.91486845761262
C	8.46935601504427	6.58808211291076	6.62848597286745
H	9.17565969169601	7.19729021375748	6.06189624255036
C	6.78965427258608	6.45058005973296	1.07272716749492
C	7.39243564820209	5.17605788056781	1.19045140088268

C	8.48740759028853	4.84848047595183	0.37507149567398
H	8.93417307064105	3.85738624627399	0.47055614053890
C	8.96965700070952	5.74741813424097	-0.57236574101411
H	9.80340384855920	5.47075469953563	-1.21847295912391
C	8.39390177589926	7.01629856049061	-0.67195982883427
H	8.79926627389062	7.75162012338900	-1.36936678577003
C	7.33482699501512	7.39246114367370	0.16023395668971
C	6.89746848092292	4.18490315767161	2.17618638715275
C	7.78466286358593	3.61886734518492	3.10709492509321
H	8.82434918036944	3.95107657340489	3.11413857955575
C	7.34760809404477	2.65635532679438	4.01974725582226
H	8.05068425064509	2.23022513384808	4.73689372865923
C	6.01524434311235	2.23946948842388	4.01224618233821
H	5.66769956082974	1.49042823350545	4.72454192342223
C	5.12160666570112	2.79675223638018	3.08968178472096
H	4.07631232485149	2.48789595981781	3.08726876126214

C	5.55507434244678	3.76782447415357	2.18630444406770
H	4.85096217608672	4.21535677921797	1.48478216813145
C	6.86028492250397	8.79984909270851	0.15349436738781
C	7.08007301758221	9.61399439625787	1.27528317614414
H	7.61579629673544	9.20306316776164	2.13445890456400
C	6.67429565348371	10.94853856574176	1.28115842835516
H	6.87542340378450	11.57397753314075	2.15137841576152
C	6.03167005297526	11.48811859779858	0.16403912259397
H	5.71355759129565	12.53175416487401	0.16692075847140
C	5.81752838825139	10.69081725127269	-0.96265921606702
H	5.32285506099149	11.10738212745703	-1.84140641300054
C	6.23037865973083	9.35720368970029	-0.96749084624123
H	6.05169301809336	8.72953892741654	-1.84154763526677
C	1.68485602315682	5.97678730628809	5.68662562788213
C	1.32834326242803	6.78741811753478	6.81716258395411
C	0.33075426944607	7.75347200565369	6.66868868205812

H	0.07008581037501	8.37267003029793	7.52715691991968
C	-0.39709920251485	7.92332351418547	5.48403983655496
C	-0.13487471990954	7.02401720933314	4.44786276160541
H	-0.73919428679449	7.08174216817899	3.54648670380741
C	0.85693883863171	6.04018738773566	4.52117365232252
C	1.87673135112605	6.45512281543201	8.21716429396529
C	1.44535684572506	5.00957443338228	8.55822226784309
H	0.34887610174840	4.92691321441476	8.55618164518352
H	1.81091021206166	4.72821723842126	9.55902956054488
H	1.85178223582037	4.30753946917906	7.82010843764167
C	3.40779204903841	6.54117922835231	8.28754565306596
H	3.74840934859186	6.29016668485444	9.30576730772088
H	3.76385597300333	7.55260189633365	8.05399742799484
H	3.86089798844620	5.84377364765154	7.57949175526230
C	1.31186500574336	7.38057244037196	9.30812831337600
H	1.58164113449911	8.43488885520189	9.14224115518865

H	1.72801323206706	7.08377854803962	10.28234993795996
H	0.21701381977793	7.31436626374130	9.38024211790473
C	-1.52417806809095	8.96362871926774	5.41901958058954
C	-2.18761353126298	9.02040365161724	4.03451485898328
H	-2.67264951592588	8.06859679135463	3.77574306341553
H	-1.45855578022157	9.25421845002418	3.24484688155048
H	-2.95979689634010	9.80372651070401	4.02361763903220
C	-0.98587294198981	10.37117065982916	5.74907427720522
H	-0.28784092517795	10.71965381268536	4.97405502505116
H	-0.45887527454156	10.38575481545917	6.71343891060028
H	-1.81177461127054	11.09723377079687	5.80445994641098
C	-2.60864990362894	8.59083479824834	6.45468796181196
H	-2.20361243263397	8.59664386119084	7.47609769512651
H	-2.99850617593073	7.58258954960062	6.25509872536401
H	-3.44743903686221	9.30383541214689	6.41565645614131
C	0.93767824268074	4.94642989493119	3.44022979641192

C	0.67302701960039	3.58089735491478	4.11601262645753
H	1.42580053463821	3.38282127945391	4.88815370742700
H	0.70660204170861	2.77505003335648	3.36511487655113
H	-0.32177720153267	3.57069269682065	4.58538739355743
C	2.30449994608536	4.91280856917194	2.73952464065433
H	2.34257675491075	4.06217573012986	2.03925884076769
H	3.11594071402554	4.80708563751262	3.46240584279728
H	2.46996108347001	5.82528303332343	2.15716355029831
C	-0.12250168209977	5.13515510183474	2.34183610640538
H	-1.14539234211564	5.10938007029543	2.74476566049170
H	-0.03121040292284	4.31899207393259	1.60962875153921
H	0.01358746252504	6.08357370698930	1.80013507316253
Bi	6.18736703453361	7.22821787572613	4.10387739564494
H	5.97883085498331	10.67296499539857	4.21428125647180
C	4.65844866182482	12.34425143828465	3.94166127722310
H	4.48110976698071	12.10670189303388	2.89234582452844

C	4.03416087807183	13.44442919511893	4.53564039511761
H	3.36156313434677	14.07442699224295	3.95244085484687
C	4.27843333948001	13.73201511438112	5.88071821300719
H	3.80066479616910	14.59178676031407	6.35275499027749
C	5.11822966376662	12.90905450269628	6.63278829309219
H	5.27561258262514	13.11437020032965	7.69304659380401
C	5.50556062774306	11.53080483579253	4.69093635444948
C	5.73350954784059	11.78611350290248	6.05427030950670
N	5.06590901185050	5.49320397392765	5.10952530865749
H	3.85559359021907	5.44189758737377	5.39761078425116
H	5.22586372215673	4.68351159467370	4.49395295927681
H	5.58935216610153	5.28329919711877	5.96926579212404

III

O	2.57887474412934	5.00442727268396	5.81967208526265

N	5.57304584543451	8.88270130422539	6.06211716537600
N	5.76347093816697	6.81020487578987	1.93032955508629
C	4.29434706887665	8.22490282709907	3.71141368228932
C	3.60589807322908	8.89734854787164	4.73168469963862
C	2.32457826066539	9.39794144443698	4.46434854417862
H	1.75311841868326	9.91120291816273	5.23400658546219
C	1.77961972820859	9.24269984897402	3.18825191335983
H	0.78352075542776	9.63387305192274	2.98147872470881
C	2.48301910911089	8.58846062899477	2.18014950180458
H	2.02685065731955	8.48048469484918	1.19567831981080
C	3.76177575749010	8.05366984821984	2.43441321994166
C	4.31672082896766	9.22185826046125	5.98122690015310
C	3.58214990923916	9.97194603275845	7.05139142420493
H	2.65158282125568	9.43355715439021	7.28345093468051
H	3.30618312809543	10.97827097316432	6.70669203226515
H	4.18934457343704	10.06837867256915	7.95794245630292

C	4.56404687687270	7.30076660267729	1.43364731973385
C	4.13547172904383	7.09806278441895	0.16062528751190
H	4.73086322550632	6.52702966051771	-0.54897342940628
H	3.20254907160596	7.52479936297986	-0.19563677513834
C	6.52812110337288	9.50931777897048	6.86961633523087
C	7.51464699939016	8.73148142326891	7.52490480565931
C	8.53309396915711	9.36468387818605	8.24549987448361
H	9.27379375865286	8.74484623336473	8.75273376731769
C	8.60391564046031	10.75484805562412	8.32090334879290
H	9.40006555416831	11.23618312653238	8.88930434858709
C	7.67432165042489	11.52272234074201	7.62499426726987
H	7.76189763147242	12.61015659677438	7.61454307103683
C	6.64849071330779	10.93183831322561	6.87375930006476
C	7.52638535971081	7.24666702254407	7.43957914108265
C	6.61021451247039	6.46533140938178	8.15242114189002
H	5.85453132542334	6.95687487347320	8.76164984684931

C	6.66343326864420	5.07084102178254	8.09134609516431
H	5.93766076687480	4.47815374276150	8.64912502995682
C	7.63176852725449	4.43925983132862	7.30630424454685
H	7.66131635657265	3.35125883041107	7.24526174434113
C	8.55589954694940	5.20852224019345	6.59492669579581
H	9.31093414802396	4.72357742343935	5.97563443750661
C	8.50956899183257	6.60179277365946	6.66814914299576
H	9.23283548018769	7.20522479642218	6.11717910180150
C	6.80989057349357	6.45252137747682	1.06555678783606
C	7.41387211660336	5.17772918032887	1.17743575491521
C	8.50871643700273	4.85222603113857	0.36165652991561
H	8.95617727359466	3.86121979548679	0.45569634683129
C	8.98959622762236	5.75339595635484	-0.58464570320869
H	9.82407763631069	5.47953408159545	-1.23106571035558
C	8.41024508114042	7.02080658545949	-0.68281735946282
H	8.81336992516756	7.75766479606933	-1.38002547528503

C	7.35154570287360	7.39480475013487	0.15100897590364
C	6.91323595055668	4.18309906283064	2.15662473975406
C	7.78905637982325	3.62497917592884	3.10205705752374
H	8.82617054936891	3.96449209935086	3.12508201591915
C	7.34304500620062	2.66302336359126	4.01110181110575
H	8.03673188098332	2.24465876502916	4.74191731517433
C	6.01443274936047	2.23669983420327	3.98227379685038
H	5.66064196124714	1.48687685807686	4.69074778353424
C	5.13260915365838	2.78518742736182	3.04358054303706
H	4.09060821972692	2.46563794935745	3.02298072166868
C	5.57338218386810	3.75805205162560	2.14623274891140
H	4.87877592757825	4.20196406390353	1.43261189630096
C	6.87546348417329	8.80141203905051	0.14277107900643
C	7.08965743761263	9.61626874994525	1.26499146034260
H	7.61687494882638	9.20483997819064	2.12878557702140
C	6.68664133736331	10.95167200987063	1.26614459426524

H	6.88491312887669	11.57795951281632	2.13645120139880
C	6.05136386662270	11.49131551744000	0.14476829338911
H	5.73586056542336	12.53582007550626	0.14419534983463
C	5.84104030957257	10.69242613905681	-0.98158649961624
H	5.35129867398122	11.10833806747371	-1.86348194788060
C	6.25165070107086	9.35811128137279	-0.98200561566892
H	6.07630917691808	8.72910476325782	-1.85582157980376
C	1.57875476380141	5.93844702498580	5.71452726857979
C	1.25277475431527	6.74179527415619	6.84134660546555
C	0.25150190242126	7.70691563864394	6.69549635291285
H	0.00115253698632	8.33441961051036	7.54957920359293
C	-0.47798815498780	7.86953082969526	5.51254059746341
C	-0.20900081103955	6.98462538994810	4.46901809295681
H	-0.80211464322885	7.05347069645061	3.56210394550197
C	0.78702083757892	6.00023115592132	4.54147173554701
C	1.84004191720696	6.44163435839531	8.23326510503353

C	1.41468707367560	5.00810005758533	8.62895157110357
H	0.31843168959635	4.92373178559438	8.64614467537076
H	1.79436097842551	4.76691662900118	9.63440833425175
H	1.81110457065640	4.27535860716274	7.91654281996223
C	3.37322014181214	6.53462995974374	8.26614365884554
H	3.72960087195335	6.30626150775315	9.28369978265408
H	3.72009357274728	7.54321074161741	8.00886284361154
H	3.83343932952796	5.83025196326363	7.56971859017984
C	1.30208580914345	7.39916318101132	9.31093410487998
H	1.56509750724622	8.44760611731484	9.10403492521070
H	1.74668900169425	7.13291574794395	10.28095812095502
H	0.21005810972795	7.33273032240979	9.41722106627005
C	-1.59099164125488	8.92432545569024	5.44041438764071
C	-2.25981548160658	8.97374667943216	4.05844289545635
H	-2.75280204550906	8.02337489006068	3.80928940492049
H	-1.53317036303377	9.19840310482665	3.26394295400269

H	-3.02653534526845	9.76203049638642	4.04553669827251
C	-1.02156365154274	10.32487325154280	5.74717082411898
H	-0.30964931282299	10.63975600259111	4.97083485154521
H	-0.50148702899583	10.34712513777315	6.71494433305862
H	-1.83117932330426	11.06985748864414	5.78133286568468
C	-2.67324978216870	8.58297786485589	6.48812242123176
H	-2.26171851343284	8.59260689651841	7.50683370819873
H	-3.08703958399905	7.58144463624454	6.30310186273803
H	-3.49663390417269	9.31287263211167	6.44615808387185
C	0.90151059176197	4.93685017047459	3.43208889802987
C	0.60802577545389	3.55212534600489	4.05457471718009
H	1.34358319797219	3.31124143182425	4.83019157326338
H	0.64606735179021	2.77553438171566	3.27432350354166
H	-0.39543429849954	3.53551456749607	4.50488431371556
C	2.28952691699949	4.92130899311739	2.77261463188372
H	2.32289276081746	4.13079903125014	2.00613926581925

H	3.08427162812188	4.72919701768839	3.49678054134409
H	2.50055589197784	5.87279094289621	2.27503919966411
C	-0.12314590873643	5.16634575507126	2.30741764042072
H	-1.15917366768817	5.12962437904835	2.67444156915653
H	-0.00944274881426	4.37326541623125	1.55403142033923
H	0.03248912677276	6.12974933029655	1.79889018437126
Bi	6.23204595348141	7.22494734705253	4.11067713956268
H	6.01581478519278	10.68507783657214	4.21661418777363
C	4.70065932397621	12.36001038618333	3.93894967508379
H	4.52113576019230	12.11886436105554	2.89080366957993
C	4.08198923510829	13.46577556528602	4.52868611226980
H	3.41144215003117	14.09602817910713	3.94332798304963
C	4.33017876423806	13.75911216258536	5.87177359617011
H	3.85740401737228	14.62355424312651	6.34041111475949
C	5.16833566963059	12.93640476710831	6.62607123438839
H	5.32938076237754	13.14671718229550	7.68483239127616

C	5.54653398659798	11.54686662301552	4.69005259161978
C	5.77836423062552	11.80835870200692	6.05178097310142
N	5.09268028739429	5.54847774028221	5.06297747342354
H	3.49369751516353	5.35422408640746	5.48968868474855
H	5.20850439019642	4.77320121197640	4.39503030079692
H	5.65810790660403	5.26414963049799	5.87363820881735

$TS_{III\text{-}IV}$

O	1.63636046485508	4.73020379878866	4.41497009277779
N	6.73167274784695	9.50536838423570	6.66361093315165
N	4.85533120325617	7.20386847341546	3.00758045565355
C	4.49543866382391	8.62958434604846	5.30870432746784
C	4.37298812796342	9.35636678042454	6.50110654470146
C	3.08392143399377	9.66788506938240	6.97147572066138
H	2.95253206734026	10.24726308741243	7.88551889818503

C	1.96151764065964	9.23967812317577	6.26389832649413
H	0.96456253681653	9.47261915532713	6.63270278982482
C	2.09563578426429	8.47122866012209	5.10784518768395
H	1.19803807465654	8.09429742189953	4.61846407022577
C	3.37633357751982	8.15722982906532	4.61768344426023
C	5.59001896919016	9.76315680286950	7.22569939135079
C	5.45928078069310	10.38949868273206	8.58247945683127
H	4.75564727033661	9.80942002428493	9.19543914647849
H	5.07033267823814	11.41521060953462	8.50193994970509
H	6.43010452697894	10.43505347224845	9.08727163754375
C	3.59061646633532	7.31345346565305	3.41389056540022
C	2.49234701806572	6.63784101346456	2.80743242394938
H	2.69300771249845	6.22910664924441	1.81360773285699
H	2.17242771021039	5.64384338384260	3.56816840604787
C	7.97740619512585	9.93599971751299	7.14885290817735
C	8.99329857256687	8.96516247672626	7.33011581022998

C	10.26614060602295	9.37432784984239	7.74788707806133
H	11.03452956998042	8.61682422625227	7.90853948765140
C	10.54431529216039	10.71981641448212	7.97813015415222
H	11.53735215558682	11.02591862972500	8.30845490511064
C	9.55319781692915	11.67477831373962	7.76314647368442
H	9.77557052696444	12.73446429144705	7.89570552123947
C	8.26750570892952	11.31338640305587	7.33413573274356
C	8.73293299441160	7.53755889561236	7.03670037670294
C	7.61038787701870	6.85951023116036	7.54530375886185
H	6.93400335253670	7.37163501826701	8.22824087809729
C	7.35555994968913	5.53631376402886	7.18458015966319
H	6.47243120990434	5.03321741663800	7.57954629388722
C	8.21931386604579	4.86461015474376	6.31491148430963
H	8.01140912008851	3.83591666398248	6.02031561102102
C	9.34788393558128	5.52037249364085	5.81813382905120
H	10.02327638959043	5.00363404075912	5.13512101647295

C	9.60362038635123	6.84341556579809	6.17840379111275
H	10.46620901737534	7.36731964831423	5.76372743394393
C	5.19271529975209	6.53412514766922	1.80204679556823
C	5.23495973644667	5.12291975945934	1.72691693663182
C	5.62760487785081	4.51745141515521	0.52202503692802
H	5.65972860379815	3.42801189973806	0.47844257803942
C	5.98824780258158	5.27668298837544	-0.58736584889882
H	6.29691088762064	4.78706896763772	-1.51168816644091
C	5.93950071307892	6.66538709810270	-0.51112476510685
H	6.18283191020942	7.27337793661010	-1.38360481844921
C	5.53169581743305	7.31147590876501	0.66592257709145
C	4.85910415299640	4.23552358520449	2.85454491726228
C	5.50188648012972	4.29162987526086	4.09930810149027
H	6.28092886688502	5.03412212276306	4.27794325868134
C	5.18815202312158	3.37022179122503	5.09685066195224
H	5.69399388575622	3.42209348553177	6.06065856736114

C	4.22071252363660	2.38936364173066	4.87634586551684
H	3.96849257301137	1.68233618503490	5.66661733426960
C	3.56051840754475	2.33770692219963	3.64911250973873
H	2.77687361372271	1.59951735498217	3.47777287578247
C	3.88242184338611	3.24856428761661	2.64596613902857
H	3.35507040448176	3.21709639332756	1.69147938962396
C	5.44862614726737	8.79177191003517	0.66709833084916
C	6.52953669976091	9.55308609433718	0.19099467066267
H	7.43141980238634	9.03977220270296	-0.14806776013256
C	6.47424704253025	10.94772132062015	0.17552975673949
H	7.32979567106175	11.52032908250524	-0.18555965003251
C	5.33218204942195	11.60836028619150	0.63408622355309
H	5.29000561628065	12.69812639643535	0.63141932459919
C	4.24172693076100	10.86158005817421	1.09053112788867
H	3.33764004912876	11.36693845429035	1.43354072005078
C	4.29677986097195	9.46762238533331	1.10310298686314

H	3.43348401256921	8.89473166261386	1.43927242360676
C	0.62751245987694	5.33350678253959	5.03637841918178
C	0.75472318002726	5.75658117079928	6.40267084537828
C	-0.23072726923268	6.58352322386716	6.93988119998059
H	-0.11969726596510	6.92653331850495	7.96855080309842
C	-1.36549928011913	6.98836898340073	6.22069106959781
C	-1.54913685785114	6.42470602761405	4.95508564974095
H	-2.47022531633099	6.63796159911511	4.41832093971895
C	-0.61675286616989	5.56043555536299	4.36459706060341
C	1.89473412488799	5.21523951225755	7.28439144617920
C	1.84615292908779	3.67235105663643	7.23017340898771
H	0.87927246299623	3.30352090016046	7.60322732102615
H	2.64562263731259	3.24444259800491	7.85673047400249
H	1.98159942646596	3.32811572499367	6.19878483851814
C	3.27948050257333	5.69769860948939	6.81392532905257
H	3.38407318581357	6.77997447745025	6.96362190126160

H	3.43987366838074	5.45818780656119	5.75894053246155
H	4.06384261011432	5.20007447139759	7.40773460359784
C	1.74190759419626	5.64030091505836	8.75401003920087
H	1.81389320111292	6.73233438947478	8.87339377911902
H	2.55191787914927	5.18744895130316	9.34568956165982
H	0.78557957647658	5.30647294102306	9.18235495585217
C	-2.39694007762815	7.91775285215010	6.87380744274677
C	-3.55563856621691	8.26122491378868	5.92513476820052
H	-4.12529435395337	7.36563663120784	5.63888673881073
H	-3.19409174620744	8.74547317708611	5.00614125790582
H	-4.25040916809678	8.95404042880230	6.42268489581013
C	-1.72129638012741	9.23844064736506	7.29597030054381
H	-1.32863759807478	9.76990287508228	6.41579737553847
H	-0.88751026469827	9.05691037727858	7.98915270487014
H	-2.44085277974690	9.90160550245082	7.80144695191258
C	-2.98341869990473	7.23224056270459	8.12666901949355

H	-2.19856122284176	7.00322133566664	8.86092492476996
H	-3.47266175975130	6.28620740565724	7.85406226404027
H	-3.72786721201276	7.88170959762573	8.61387187562936
C	-0.99935600873560	4.73815479247753	3.11508645884637
C	-0.85335719316262	3.24122774850323	3.47841147565049
H	0.17982829503342	3.02254236366316	3.77481996013045
H	-1.11730476599583	2.61298726725328	2.61270315680510
H	-1.52185378994577	2.97951577762730	4.31153897843041
C	-0.12597731781348	5.02601413931349	1.87899084728364
H	-0.50506815519653	4.45209059819905	1.01827470229605
H	0.91247780666392	4.73341095870017	2.05860052477924
H	-0.15502900336163	6.09206122044105	1.60926892140786
C	-2.46236373481092	4.97788076250578	2.70237262240267
H	-3.16206316950912	4.75177240286087	3.51981936484496
H	-2.71008643421484	4.31971415044110	1.85650805773075
H	-2.63513803126294	6.01491069178564	2.37659259433706

Bi	6.49277255803733	8.08880791420380	4.49336554792932
H	6.73297679243294	11.63941659317742	5.09388544479192
C	5.60136779699863	13.40223610682750	5.62890046985528
H	5.04654533831321	13.40952331613409	4.68934071845330
C	5.36738828494643	14.39224579434376	6.58819798445921
H	4.62246656716136	15.16842036017338	6.40711495843843
C	6.09899856380536	14.38576715787922	7.77808257449895
H	5.92808153815480	15.15633235534405	8.53138605532374
C	7.04445391023011	13.38570005119271	8.01130475449470
H	7.59567048140481	13.36653422938654	8.95349854315819
C	6.54643571156228	12.40229664735904	5.85614683675477
C	7.27088579273588	12.37698275230134	7.06003350969043
H	1.53763074673941	7.17093420855010	2.85803507042243
N	6.79018938592325	10.02364368913509	3.53890891950304
H	7.42515041060233	9.89310137755764	2.74489300376092
H	5.89814665573301	10.30405186224895	3.11445480888160

IV

O	2.57887474412934	5.00442727268396	5.81967208526265
N	5.57304584543451	8.88270130422539	6.06211716537600
N	5.76347093816697	6.81020487578987	1.93032955508629
C	4.29434706887665	8.22490282709907	3.71141368228932
C	3.60589807322908	8.89734854787164	4.73168469963862
C	2.32457826066539	9.39794144443698	4.46434854417862
H	1.75311841868326	9.91120291816273	5.23400658546219
C	1.77961972820859	9.24269984897402	3.18825191335983
H	0.78352075542776	9.63387305192274	2.98147872470881
C	2.48301910911089	8.58846062899477	2.18014950180458
H	2.02685065731955	8.48048469484918	1.19567831981080
C	3.76177575749010	8.05366984821984	2.43441321994166

C	4.31672082896766	9.22185826046125	5.98122690015310
C	3.58214990923916	9.97194603275845	7.05139142420493
H	2.65158282125568	9.43355715439021	7.28345093468051
H	3.30618312809543	10.97827097316432	6.70669203226515
H	4.18934457343704	10.06837867256915	7.95794245630292
C	4.56404687687270	7.30076660267729	1.43364731973385
C	4.13547172904383	7.09806278441895	0.16062528751190
H	4.73086322550632	6.52702966051771	-0.54897342940628
H	3.20254907160596	7.52479936297986	-0.19563677513834
C	6.52812110337288	9.50931777897048	6.86961633523087
C	7.51464699939016	8.73148142326891	7.52490480565931
C	8.53309396915711	9.36468387818605	8.24549987448361
H	9.27379375865286	8.74484623336473	8.75273376731769
C	8.60391564046031	10.75484805562412	8.32090334879290
H	9.40006555416831	11.23618312653238	8.88930434858709
C	7.67432165042489	11.52272234074201	7.62499426726987

H	7.76189763147242	12.61015659677438	7.61454307103683
C	6.64849071330779	10.93183831322561	6.87375930006476
C	7.52638535971081	7.24666702254407	7.43957914108265
C	6.61021451247039	6.46533140938178	8.15242114189002
H	5.85453132542334	6.95687487347320	8.76164984684931
C	6.66343326864420	5.07084102178254	8.09134609516431
H	5.93766076687480	4.47815374276150	8.64912502995682
C	7.63176852725449	4.43925983132862	7.30630424454685
H	7.66131635657265	3.35125883041107	7.24526174434113
C	8.55589954694940	5.20852224019345	6.59492669579581
H	9.31093414802396	4.72357742343935	5.97563443750661
C	8.50956899183257	6.60179277365946	6.66814914299576
H	9.23283548018769	7.20522479642218	6.11717910180150
C	6.80989057349357	6.45252137747682	1.06555678783606
C	7.41387211660336	5.17772918032887	1.17743575491521
C	8.50871643700273	4.85222603113857	0.36165652991561

H	8.95617727359466	3.86121979548679	0.45569634683129
C	8.98959622762236	5.75339595635484	-0.58464570320869
H	9.82407763631069	5.47953408159545	-1.23106571035558
C	8.41024508114042	7.02080658545949	-0.68281735946282
H	8.81336992516756	7.75766479606933	-1.38002547528503
C	7.35154570287360	7.39480475013487	0.15100897590364
C	6.91323595055668	4.18309906283064	2.15662473975406
C	7.78905637982325	3.62497917592884	3.10205705752374
H	8.82617054936891	3.96449209935086	3.12508201591915
C	7.34304500620062	2.66302336359126	4.01110181110575
H	8.03673188098332	2.24465876502916	4.74191731517433
C	6.01443274936047	2.23669983420327	3.98227379685038
H	5.66064196124714	1.48687685807686	4.69074778353424
C	5.13260915365838	2.78518742736182	3.04358054303706
H	4.09060821972692	2.46563794935745	3.02298072166868
C	5.57338218386810	3.75805205162560	2.14623274891140

H	4.87877592757825	4.20196406390353	1.43261189630096
C	6.87546348417329	8.80141203905051	0.14277107900643
C	7.08965743761263	9.61626874994525	1.26499146034260
H	7.61687494882638	9.20483997819064	2.12878557702140
C	6.68664133736331	10.95167200987063	1.26614459426524
H	6.88491312887669	11.57795951281632	2.13645120139880
C	6.05136386662270	11.49131551744000	0.14476829338911
H	5.73586056542336	12.53582007550626	0.14419534983463
C	5.84104030957257	10.69242613905681	-0.98158649961624
H	5.35129867398122	11.10833806747371	-1.86348194788060
C	6.25165070107086	9.35811128137279	-0.98200561566892
H	6.07630917691808	8.72910476325782	-1.85582157980376
C	1.57875476380141	5.93844702498580	5.71452726857979
C	1.25277475431527	6.74179527415619	6.84134660546555
C	0.25150190242126	7.70691563864394	6.69549635291285
H	0.00115253698632	8.33441961051036	7.54957920359293

C	-0.47798815498780	7.86953082969526	5.51254059746341
C	-0.20900081103955	6.98462538994810	4.46901809295681
H	-0.80211464322885	7.05347069645061	3.56210394550197
C	0.78702083757892	6.00023115592132	4.54147173554701
C	1.84004191720696	6.44163435839531	8.23326510503353
C	1.41468707367560	5.00810005758533	8.62895157110357
H	0.31843168959635	4.92373178559438	8.64614467537076
H	1.79436097842551	4.76691662900118	9.63440833425175
H	1.81110457065640	4.27535860716274	7.91654281996223
C	3.37322014181214	6.53462995974374	8.26614365884554
H	3.72960087195335	6.30626150775315	9.28369978265408
H	3.72009357274728	7.54321074161741	8.00886284361154
H	3.83343932952796	5.83025196326363	7.56971859017984
C	1.30208580914345	7.39916318101132	9.31093410487998
H	1.56509750724622	8.44760611731484	9.10403492521070
H	1.74668900169425	7.13291574794395	10.28095812095502

H	0.21005810972795	7.33273032240979	9.41722106627005
C	-1.59099164125488	8.92432545569024	5.44041438764071
C	-2.25981548160658	8.97374667943216	4.05844289545635
H	-2.75280204550906	8.02337489006068	3.80928940492049
H	-1.53317036303377	9.19840310482665	3.26394295400269
H	-3.02653534526845	9.76203049638642	4.04553669827251
C	-1.02156365154274	10.32487325154280	5.74717082411898
H	-0.30964931282299	10.63975600259111	4.97083485154521
H	-0.50148702899583	10.34712513777315	6.71494433305862
H	-1.83117932330426	11.06985748864414	5.78133286568468
C	-2.67324978216870	8.58297786485589	6.48812242123176
H	-2.26171851343284	8.59260689651841	7.50683370819873
H	-3.08703958399905	7.58144463624454	6.30310186273803
H	-3.49663390417269	9.31287263211167	6.44615808387185
C	0.90151059176197	4.93685017047459	3.43208889802987
C	0.60802577545389	3.55212534600489	4.05457471718009

H	1.34358319797219	3.31124143182425	4.83019157326338
H	0.64606735179021	2.77553438171566	3.27432350354166
H	-0.39543429849954	3.53551456749607	4.50488431371556
C	2.28952691699949	4.92130899311739	2.77261463188372
H	2.32289276081746	4.13079903125014	2.00613926581925
H	3.08427162812188	4.72919701768839	3.49678054134409
H	2.50055589197784	5.87279094289621	2.27503919966411
C	-0.12314590873643	5.16634575507126	2.30741764042072
H	-1.15917366768817	5.12962437904835	2.67444156915653
H	-0.00944274881426	4.37326541623125	1.55403142033923
H	0.03248912677276	6.12974933029655	1.79889018437126
Bi	6.23204595348141	7.22494734705253	4.11067713956268
H	6.01581478519278	10.68507783657214	4.21661418777363
C	4.70065932397621	12.36001038618333	3.93894967508379
H	4.52113576019230	12.11886436105554	2.89080366957993
C	4.08198923510829	13.46577556528602	4.52868611226980

H	3.41144215003117	14.09602817910713	3.94332798304963
C	4.33017876423806	13.75911216258536	5.87177359617011
H	3.85740401737228	14.62355424312651	6.34041111475949
C	5.16833566963059	12.93640476710831	6.62607123438839
H	5.32938076237754	13.14671718229550	7.68483239127616
C	5.54653398659798	11.54686662301552	4.69005259161978
C	5.77836423062552	11.80835870200692	6.05178097310142
N	5.09268028739429	5.54847774028221	5.06297747342354
H	3.49369751516353	5.35422408640746	5.48968868474855
H	5.20850439019642	4.77320121197640	4.39503030079692
H	5.65810790660403	5.26414963049799	5.87363820881735

11 References

(1) Jia, X.; Kramer, S.; Skrydstrup, T.; Lian, Z. Design and applications of a SO_2 surrogate in palladium-catalyzed direct aminosulfonylation between aryl iodides and amines. *Angew. Chem. Int. Ed.* **2021**, *60*, 7353-7359.

(2) Pang, Y.; Leutzsch, M.; Nöthling, N.; Cornella, J. Catalytic activation of N_2O at a low-valent bismuth redox platform. *J. Am. Chem. Soc.* **2020**, *142*, 19473-19479.

(3) Manner, V. W.; Markle, T. F.; Freudenthal, J. H.; Roth, J. P.; Mayer, J. M. The first crystal structure of a monomeric phenoxyl radical: 2,4,6-tri-*tert*-butylphenoxyl radical. *Chem. Commun.* **2008**, 256-258.

(4) Clayden, J.; Hebditch, K. R.; Read, B.; Helliwell, M. Oxidative fragmentation of bicyclic hydroxy silanes and stannanes: a strategy for the stereoselective synthesis of kainoids. *Tetrahedron Lett.* **2007**, *48*, 8550-8553.

(5) Bentley, J. N.; Caputo, C. B. Catalytic hydroarylation of alkenes with phenols using $B(C_6F_5)_3$. *Organometallics* **2018**, *37*, 3654-3658.

(6) Cohen, S. G.; Chao, H. M. Photoreduction of aromatic ketones by amines. Studies of quantum yields and mechanism. *J. Am. Chem. Soc.* **1968**, *90*, 165-173.

(7) Leoni, P.; Landi, A.; Pasquali, M. Isolation of the first neutral chromium formyl derivatives. *J. Organomet. Chem.* **1987**, *321*, 365-369.

(8) Harris, R. K.; Becker, E. D.; Menezes, S. M. C. d.; Granger, P.; Hoffman, R. E.; Zilm, K. W. Further conventions for NMR shielding and chemical shifts. *Pure Appl. Chem.* **2008**, *80*, 59-84.

(9) Bettinger, H. F.; Filthaus, M.; Bornemann, H.; Oppel, I. M. Metal-free conversion of methane and cycloalkanes to amines and amides by employing a borylnitrene. *Angew. Chem. Int. Ed.* **2008**, *47*, 4744-4747.

(10) Stoll, S.; Schweiger, A. EasySpin, a comprehensive software package for spectral simulation and analysis in EPR. *J. Magn. Reson.* **2006**, *178*, 42-55.

(11) (a) Neese, F. The ORCA program system. *WIREs Comput Mol Sci* **2012**, *2*, 73-78. (b) Neese, F.; Wennmohs, F.; Becker, U.; Riplinger, C. The ORCA quantum chemistry program package. *J. Chem. Phys.* **2020**, *152*, 224108-224118.

(12) (a) Perdew, J. P. Density-functional approximation for the correlation energy of the inhomogeneous electron gas. *Phys. Rev. B* **1986**, *33*, 8822-8824. (b) Becke, A. D. Density-functional exchange-energy approximation with correct asymptotic behavior. *Phys. Rev. A* **1988**, *38*, 3098-3100. (c) Weigend, F.; Ahlrichs, R. Balanced basis sets of split valence, triple zeta valence and quadruple zeta valence quality for H to Rn: design and assessment of accuracy. *Phys. Chem. Chem. Phys.* **2005**, *7*, 3297-3305. (d) Pantazis, D. A.; Chen, X.-Y.; Landis, C. R.; Neese, F. All-electron scalar relativistic basis sets for third-row transition metal atoms. *J. Chem. Theory Comput.* **2008**, *4*, 908-919. (e) Pantazis, D. A.; Neese, F. All-electron scalar relativistic basis sets for the 6*p* elements. *Theor Chem Acc* **2012**, *131*, 1292-1299.

(13) (a) Eichkorn, K.; Weigend, F.; Treutler, O.; Ahlrichs, R. Auxiliary basis sets for main row atoms and transition metals and their use to approximate Coulomb potentials. *Theor Chem Acc* **1997**, *97*, 119-124. (b)

Eichkorn, K.; Treutler, O.; Öhm, H.; Häser, M.; Ahlrichs, R. Auxiliary basis sets to approximate Coulomb potentials. *Chem. Phys. Lett.* **1995**, *240*, 283-290. (c) Weigend, F. Accurate Coulomb-fitting basis sets for H to Rn. *Phys. Chem. Chem. Phys.* **2006**, *8*, 1057-1065. (d) Neese, F. An improvement of the resolution of the identity approximation for the formation of the Coulomb matrix. *J. Comput. Chem.* **2003**, *24*, 1740-1747.

(14) Grimme, S.; Ehrlich, S.; Goerigk, L. Effect of the damping function in dispersion corrected density functional theory. *J. Comput. Chem.* **2011**, *32*, 1456-1465.

(15) (a) Adamo, C.; Barone, V. Toward reliable density functional methods without adjustable parameters: the PBE0 model. *J. Chem. Phys.* **1999**, *110*, 6158-6170. (b) Ernzerhof, M.; Scuseria, G. E. Assessment of the Perdew–Burke–Ernzerhof exchange-correlation functional. *J. Chem. Phys.* **1999**, *110*, 5029-5036. (c) Marenich, A. V.; Cramer, C. J.; Truhlar, D. G. Universal solvation model based on solute electron density and on a continuum model of the solvent defined by the bulk dielectric constant and atomic surface tensions. *J. Phys. Chem. B* **2009**, *113*, 6378-6396.

(16) (a) Nazemi, A.; Cundari, T. R. Control of C–H bond activation by Mo-oxo complexes: pK_a or bond dissociation free energy (BDFE)? *Inorg. Chem.* **2017**, *56*, 12319-12327. (b) Isegawa, M.; Neese, F.; Pantazis, D. A. Ionization energies and aqueous redox potentials of organic molecules: comparison of DFT, correlated ab initio theory and pair natural orbital approaches. *J. Chem. Theory Comput.* **2016**, *12*, 2272-2284.

(17) (a) Gottschalk, H. C.; Poblotzki, A.; Suhm, M. A.; Al-Mogren, M. M.; Antony, J.; Auer, A. A.; Baptista, L.; Benoit, D. M.; Bistoni, G.; Bohle, F.; et al. The furan microsolvation blind challenge for quantum chemical methods: First steps. *J. Chem. Phys.* **2018**, *148*, 014301-014313. (b) Gottschalk, H. C.; Poblotzki, A.; Fatima, M.; Obenchain, D. A.; Pérez, C.; Antony, J.; Auer, A. A.; Baptista, L.; Benoit, D. M.; Bistoni, G.; et al. The first microsolvation step for furans: new experiments and benchmarking strategies. *J. Chem. Phys.* **2020**, *152*, 164303-164317. (c) Qu, Z.-w.; Hansen, A.; Grimme, S. Co–C bond dissociation energies in cobalamin derivatives and dispersion effects: anomaly or just challenging? *J. Chem. Theory Comput.* **2015**, *11*, 1037-1045. (d) Zhao, Y.; Truhlar, D. G. Density functional theory for reaction energies: test of Meta and Hybrid Meta functionals, range-separated functionals, and other high-performance functionals. *J. Chem. Theory Comput.* **2011**, *7*, 669-676.

(18) (a) Khaskin, E.; Iron, M. A.; Shimon, L. J. W.; Zhang, J.; Milstein, D. N−H activation of amines and ammonia by Ru via metal−ligand cooperation. *J. Am. Chem. Soc.* **2010**, *132*, 8542-8543. (b) Rauch, M.; Kar, S.; Kumar, A.; Avram, L.; Shimon, L. J. W.; Milstein, D. Metal–ligand cooperation facilitates bond activation and catalytic hydrogenation with zinc pincer complexes. *J. Am. Chem. Soc.* **2020**, *142*, 14513-14521.
